# Supplementary material for: Simple and Rapid Site-Specific Integration of Multiple Heterologous DNAs into the Escherichia coli Chromosome
Source: J Bacteriol. 2023 Jan 19;205(2):e00338-22. doi: 10.1128/jb.00338-22 (PMC9945576; doi:10.1128/jb.00338-22)
Supplement: Supplemental file 1 — Concatenated maps of plasmids used in this study. Download jb.00338-22-s0001.pdf, PDF file, 2.6 MB [file jb.00338-22-s0001.pdf]

```
LOCUS       pGSs009                 3009 bp    DNA     circular UNA 29-
APR-2019
DEFINITION  .
ACCESSION   urn.local...g-exuzo53
VERSION     urn.local...g-exuzo53
KEYWORDS    .
SOURCE
  ORGANISM  .
FEATURES             Location/Qualifiers
     misc_feature    1..578
                     /label="Target for MTase"
     misc_feature    2..43
                     /label="C31 attp"
     misc_feature    206..225
                     /label="Plasmid #1 R"
     misc_feature    513..532
                     /label="plasmid#1 F"
     misc_feature    517..561
                     /label="c31 attB"
     misc_feature    886..1278
                     /label="lambda attP"
     misc_feature    1279..1715
                     /label="oriR6K"
     misc_feature    1769..2563
                     /label="Kan"
ORIGIN
        1 cgtgccccaa ctggggtaac ctttgagttc tctcagttgg gggcgtaggg
tctagaattg
       61 tgagcggata acaattacga gcttcatgca cagtgaaatc atgaaaaatt
tatttgcttt
      121 gtgagcggat aacaattata atatgtggaa ttgtgagcgc tcacaattcc
acaacggttt
      181 ccctctagaa ataattttgt ttaactttta taggaggtgt tccatatgac
catgattacg
      241 aatagtagct cggtacccgg ggaccctcta gagtcgacct gccgccatgc
aagcttggca
      301 ctggccgtcg ttttacaacg tcgtgactgg gaaaaccctg gcgttaccca
acttaatcgc
      361 cttgcagcac atccccctttt cgccagctgg cgtaatagcg aagaggcccg
caccgatcgc
      421 ccttcccaac agttgcgcag cctgaatggc gaatggcgag cttggctgtt
ttggcggatg
      481 agagaagatt ttcagcctga tacagattaa ggatcctgcg ggtgccaggg
cgtgcccttg
      541 ggctccccgg gcgcgtactc cacctcactc gagaattctc atgtttgaca
gcttatcact
      601 gatcagtgaa ttaatggcga tgacgcatcc tcacgataat atccgggtag
gcgcaatcac
      661 tttcgtctct actccgttac aaagcgaggc tgggtatttc ccggcctttc
tgttatccga
      721 aatccactga aagcacagcg gctggctgag gagataaata ataaacgagg
ggctgtatgc
      781 acaaagcatc ttctgttgag ttaagaacga gtatcgagat ggcacatagc
cttgctcaaa
```

841 ttggaatcag gtttgtgcca ataccagtag aaacagacga agaagctagc tttgcactgg
901 attgcgaggc tttgtgcttc tctggagtgc gacaggtttg atgacaaaaa attagcgcaa
961 gaagacaaaa atcaccttgc gctaatgctc tgttacaggt cactaatacc atctaagtag
1021 ttgattcata gtgactgcat atatgttgtg ttttacagta ttatgtagtc tgttttttat
1081 gcaaaatcta atttaatata ttgatattta tatcatttta cgtttctcgt tcagcttttt
1141 tatactaagt tggcattata aaaaagcatt gcttatcaat ttgttgcaac gaacaggtca
1201 ctatcagtca aaataaaatc attatttgat ttcaattttg tcccactccc tgcctctgtc
1261 atcacgatac tgtgatgcca tggctaattc ccatgtcagc cgttaagtgt tcctgtgtca
1321 ctcaaaattg ctttgagagg ctctaagggc ttctcagtgc gttacatccc tggcttgttg
1381 tccacaaccg ttaaacctta aaagctttaa aagccttata tattcttttt tttcttataa
1441 aacttaaaac cttagaggct atttaagttg ctgatttata ttaattttat tgttcaaaca
1501 tgagagctta gtacgtgaaa catgagagct tagtacgtta gccatgagag cttagtacgt
1561 tagccatgag ggtttagttc gttaaacatg agagcttagt acgttaaaca tgagagctta
1621 gtacgtgaaa catgagagct tagtacgtac tatcaacagg ttgaactgct gatcttcaga
1681 tcctctacgc cggacgcatc gtggccggat cttgcggccg caaaaattaa aaatgaagtt
1741 ttgacggtat cgaaccccag agtcccgctc agaagaactc gtcaagaagg cgatagaagg
1801 cgatgcgctg cgaatcggga gcggcgatac cgtaaagcac gaggaagcgg tcagcccatt
1861 cgccgccaag ctcttcagca atatcacggg tagccaacgc tatgtcctga tagcggtccg
1921 ccacacccag ccggccacag tcgatgaatc cagaaaagcg gccattttcc accatgatat
1981 tcggcaagca ggcatcgcca tgggtcacga cgagatcctc gccgtcgggc atccgcgcct
2041 tgagcctggc gaacagttcg gctggcgcga gcccctgatg ctcttcgtcc agatcatcct
2101 gatcgacaag accggcttcc atccgagtac gtgctcgctc gatgcgatgt ttcgcttggt
2161 ggtcgaatgg gcaggtagcc ggatcaagcg tatgcagccg ccgcattgca tcagccatga
2221 tggatacttt ctcggcagga gcaaggtgag atgacaggag atcctgcccc ggcacttcgc
2281 ccaatagcag ccagtccctt cccgcttcag tgacaacgtc gagcacagct gcgcaaggaa
2341 cgcccgtcgt ggccagccac gatagccgcg ctgcctcgtc ttggagttca ttcagggcac
2401 cggacaggtc ggtcttgaca aaaagaaccg ggcgcccctg cgctgacagc cggaacacgg

2461 cggcatcaga gcagccgatt gtctgttgtg cccagtcata gccgaatagc
ctctccaccc
2521 aagcggccgg agaacctgcg tgcaatccat cttgttcaat catgcgaaac
gatcctcatc
2581 ctgtctcttg atccactaga ttattgaagc atttatcagg gttattgtct
catgagcgga
2641 tacatatttg aatgtattta gaaaaataaa caaatagggg ttccgcgcac
atttccccga
2701 aaagtgccac ctgcatcgat ggcccccgat ggtagtgtgg ggtctcccca
tgcgagagta
2761 gggaactgcc aggcatcaaa taaaacgaaa ggctcagtcg aaagactggg
cctttcgttt
2821 tatctgttgt ttgtcggtga acgctctcct gagtaggaca aatccgccgg
gagcggattt
2881 gaacgttgcg aagcaacggc ccggagggtg gcgggcagga cgcccgccat
aaactgccag
2941 gcatcaaatt aagcagaagg ccatcctgac ggatggcctt tttgcgtggc
cagtgccaag
3001 cttgcatgc
//

```
LOCUS       pGSs037                 5798 bp    DNA     circular UNA 14-
DEC-2015
DEFINITION  CRIM helper plasmid pINT-ts, complete sequence.
ACCESSION   urn.local...43-exvb5az
VERSION     urn.local...43-exvb5az
KEYWORDS    .
SOURCE      CRIM helper plasmid pINT-ts (CRIM helper plasmid pINT-ts.)
  ORGANISM  CRIM helper plasmid pINT-ts
            other sequences;artificial sequences;vectors.
FEATURES             Location/Qualifiers
     misc_feature    5798
                     /Description="dimer interface [polypeptide binding];
other
                     site"
                     /db_xref="CDD:238415"
                     /gene="int"
                     /label="int"
     misc_feature    5798
                     /Description="active site"
                     /db_xref="CDD:238415"
                     /gene="int"
                     /label="int"
     misc_feature    5798
                     /Description="Int/Topo IB signature motif; other
site"
                     /db_xref="CDD:238415"
                     /gene="int"
                     /label="int"
     CDS             complement(62..775)
                     /db_xref="GI:16209182"
                     /product="cI"
                     /codon_start=1
                     /protein_id="AAL09915.1"
                     /transl_table=11
                     /label="CDS_1"
     misc_feature    complement(74..712)
                     /Description="SOS-response transcriptional
repressors
                     (RecA-mediated autopeptidases) [Transcription /
Signal
                     transduction mechanisms]; Region: LexA; COG1974"
                     /db_xref="CDD:224885"
                     /label="Misc_Feature_3"
     misc_feature    complement(95..352)
                     /Description="Peptidase S24 LexA-like proteins are
                     involved in the SOS response leading to the repair
of
                     single-stranded DNA within the bacterial cell. This
family
                     includes: the lambda repressor CI/C2 family and
related
                     bacterial prophage repressor proteins; LexA (EC...;
                     Region: S24_LexA-like; cd06529"
                     /db_xref="CDD:119397"
```

```
                     /label="Misc_Feature_1"
     misc_feature    complement(197..328)
                     /Description="Catalytic site [active]"
                     /db_xref="CDD:119397"
                     /label="Misc_Feature_4"
     misc_feature    complement(545..730)
                     /Description="Helix-turn-helix XRE-family like
proteins.
                     Prokaryotic DNA binding proteins belonging to the
                     xenobiotic response element family of
transcriptional
                     regulators; Region: HTH_XRE; cd00093"
                     /db_xref="CDD:238045"
                     /label="Misc_Feature_2"
     misc_feature    complement(614..676)
                     /Description="sequence-specific DNA binding site
                     [nucleotide binding]; other site"
                     /db_xref="CDD:238045"
                     /label="Misc_Feature_6"
     misc_feature    complement(617..718)
                     /Description="non-specific DNA binding site
[nucleotide
                     binding]; other site"
                     /db_xref="CDD:238045"
                     /label="Misc_Feature_5"
     misc_feature    complement(620..709)
                     /Description="salt bridge; other site"
                     /db_xref="CDD:238045"
                     /label="Misc_Feature_7"
     CDS             878..2857
                     /label="PhiC31int-attB"
     CDS             complement(2965..3915)
                     /db_xref="GI:16209184"
                     /product="repA101"
                     /codon_start=1
                     /protein_id="AAL09917.1"
                     /transl_table=11
                     /label="CDS_2"
     misc_feature    complement(3253..3912)
                     /Description="Initiator Replication protein; Region:
                     Rep_3; pfam01051"
                     /db_xref="CDD:250324"
                     /label="Misc_Feature_8"
     CDS             complement(4807..5667)
                     /db_xref="GI:16209185"
                     /product="bla"
                     /codon_start=1
                     /protein_id="AAL09918.1"
                     /transl_table=11
                     /label="CDS_3"
     misc_feature    complement(4810..5661)
                     /Description="beta-lactamase TEM; Provisional;
Region:
                     PRK15442"
```

/db_xref="CDD:185339"
/label="Misc_Feature_9"
ORIGIN
1 catcgatgaa gattcttgct caattgttat cagctatgcg ccgaccagaa caccttgccg
61 atcagccaaa cgtctcttca ggccactgac tagcgataac tttccccaca acggaacaac
121 tctcattgca tgggatcatt gggtactgtg ggtttagtgg ttgtaaaaac acctgaccgc
181 tatccctgat cagtttcttg aaggtaaact catcaccccc aagtctggct atgcagaaat
241 cacctggctc aacagcctgc tcagggtcaa cgagaattaa cattccgtca ggaaagcttg
301 gcttggagcc tgttggtgcg gtcatggaat taccttcaac ctcaagccag aatgcagaat
361 cactggcttt tttggttgtg cttacccatc tctccgcatc acctttggta aaggttctaa
421 gcttaggtga gaacatccct gcctgaacat gagaaaaaac agggtactca tactcacttc
481 taagtgacgg ctgcatacta accgcttcat acatctcgta gatttctctg gcgattgaag
541 ggctaaattc ttcaacgcta actttgagaa tttttgtaag caatgcggcg ttataagcat
601 ttaatgcatt gatgccatta aataaagcac caacgcctga ctgccccatc cccatcttgt
661 ctgcgacaga ttcctgggat aagccaagtt catttttctt tttttcataa attgctttaa
721 ggcgacgtgc gtcctcaagc tgctcttgtg ttaatggttt ctttttttgtg ctcatacgtt
781 aaatctatca ccgcaaggga taaatatcta acaccgtgcg tgttgactat tttacctctg
841 gcggtgataa tggttgcatg tactaaggag gttgtatgaa ttcgagctcg gtacccagtt
901 cgtcgatggg ttcgggaatg caggatattc ccacctccgg ttaaggatgg aagagagtat
961 ctgttccacg aatcagcggt aaaggttgac ttaaatcgac cagtaacagg tggccttttg
1021 aagaggactg cagatggaca cctacgcggg cgcgtatgat cgtcagagcc gtgaacgtga
1081 aaacagcagc gcggcgagcc cggcgaccca gcgtagcgcg aacgaagaca aagcggcgga
1141 tctgcaacgt gaggtggaac gtgacggtgg ccgtttccgt tttgtgggtc actttagcga
1201 ggcgccgggt accagcgcgt ttggtaccgc ggaacgtccg gagtttgaac gtatcctgaa
1261 cgagtgccgt gcgggtcgtc tgaacatgat cattgtgtac gacgttagcc gtttcagccg
1321 tctgaaggtg atggatgcga tcccgattgt tagcgaactg ctggcgctgg gtgtgaccat
1381 tgttagcacc caggaaggtg tgtttcgtca gggtaacgtt atggacctga tccacctgat
1441 tatgcgtctg gatgcgagcc acaaagaaag cagcctgaaa agcgcgaaga tcctggacac

1501 caagaacctg caacgtgaac tgggtggcta cgtgggtggc aaagcgccgt atggcttcga
1561 gctggttagc gagaccaagg aaattacccg taacggtcgt atggtgaacg tggttatcaa
1621 caaactggcg cacagcacca ccccgctgac cggtccgttc gagtttgaac cggatgttat
1681 tcgttggtgg tggcgtgaga tcaaaaccca caagcacctg ccgtttaaac cgggtagcca
1741 agcggcgatc cacccgggca gcattaccgg tctgtgcaaa cgtatggatg cggatgcggt
1801 gccgacccgt ggcgaaacca ttggtaagaa aaccgcgagc agcgcgtggg acccggcgac
1861 cgtgatgcgt attctgcgtg atccgcgtat cgcgggtttc gcggcggaag tgatctacaa
1921 gaaaaagccg gacggcaccc cgaccaccaa aatcgaaggt tatcgtattc agcgtgaccc
1981 gatcaccctg cgtccggtgg agctggattg cggtccgatc attgagccgg cggaatggta
2041 tgaactgcaa gcgtggctgg atggtcgtgg tcgtggtaaa ggcctgagcc gtggtcaagc
2101 gattctgagc gcgatggata agctgtactg cgaatgcggc gcggttatga ccagcaaacg
2161 tggtgaggaa agcatcaagg acagctatcg ttgccgtcgt cgtaaggtgg ttgatccgag
2221 cgcgccgggt caacatgagg gtacctgcaa cgtgagcatg gcggcgctgg acaaattcgt
2281 tgcggaacgt atctttaaca agattcgtca cgcggagggt gatgaggaaa ccctggcgct
2341 gctgtgggaa gcggcgcgtc gttttggcaa actgaccgaa gcgccggaga agagcggtga
2401 acgtgcgaac ctggtggcgg agcgtgcgga tgcgctgaac gcgctggagg aactgtacga
2461 agaccgtgcg gcgggtgcgt atgatggtcc ggttggtcgt aaacacttcc gtaagcagca
2521 agcggcgctg accctgcgtc agcagggtgc ggaggaacgt ctggcggagc tggaagcggc
2581 ggaagcgccg aagctgccgc tggaccagtg gtttccggag gatgcggatg cggacccgac
2641 cggtccgaaa tcctggtggg gtcgtgcgag cgtggacgat aagcgtgtgt tcgttggcct
2701 gtttgttgat aaaatcgtgg ttaccaaaag caccaccggt cgtggtcagg gtaccccgat
2761 tgagaaacgt gcgagcatta cctgggcgaa gccgccgacc gatgatgacg aagatgacgc
2821 gcaagacggt accgaggatg ttgcggcgta agctagccca tgggtatgga cagttttccc
2881 tttgatatgt aacggtgaac agttgttcta cttttgtttg ttagtcttga tgcttcactg
2941 atagatacaa gagccataag aacctcagat ccttccgtat ttagccagta tgttctctag
3001 tgtggttcgt tgtttttgcg tgagccatga gaacgaacca ttgagatcat acttactttg
3061 catgtcactc aaaaattttg cctcaaaact ggtgagctga atttttgcag ttaaagcatc

3121 gtgtagtgtt tttcttagtc cgttacgtag gtaggaatct gatgtaatgg ttgttggtat
3181 tttgtcacca ttcattttta tctggttgtt ctcaagttcg gttacgagat ccatttgtct
3241 atctagttca acttggaaaa tcaacgtatc agtcgggcgg cctcgcttat caaccaccaa
3301 tttcatattg ctgtaagtgt ttaaatcttt acttattggt ttcaaaaccc attggttaag
3361 ccttttaaac tcatggtagt tattttcaag cattaacatg aacttaaatt catcaaggct
3421 aatctctata tttgccttgt gagttttctt ttgtgttagt tcttttaata accactcata
3481 aatcctcata gagtatttgt tttcaaaaga cttaacatgt tccagattat attttatgaa
3541 ttttttttaac tggaaaagat aaggcaatat ctcttcacta aaaactaatt ctaattttttc
3601 gcttgagaac ttggcatagt ttgtccactg gaaaatctca aagcctttaa ccaaaggatt
3661 cctgatttcc acagttctcg tcatcagctc tctggttgct ttagctaata caccataagc
3721 attttcccta ctgatgttca tcatctgagc gtattggtta taagtgaacg ataccgtccg
3781 ttctttcctt gtagggtttt caatcgtggg gttgagtagt gccacacagc ataaaattag
3841 cttggtttca tgctccgtta agtcatagcg actaatcgct agttcatttg ctttgaaaac
3901 aactaattca gacatacatc tcaattggtc taggtgattt taatcactat accaattgag
3961 atgggctagt caatgataat tactagtcct tttcctttga gttgtgggta tctgtaaatt
4021 ctgctagacc tttgctggaa aacttgtaaa ttctgctaga ccctctgtaa attccgctag
4081 acctttgtgt gttttttttg tttatattca agtggttata atttatagaa taaagaaaga
4141 ataaaaaaag ataaaaagaa tagatcccag ccctgtgtat aactcactac tttagtcagt
4201 tccgcagtat tacaaaagga tgtcgcaaac gctgtttgct cctctacaaa acagacctta
4261 aaaccctaaa ggcttaagta gcaccctcgc aagctcggtt gcggccgcaa tcgggcaaat
4321 cgctgaatat tccttttgtc tccgaccatc aggcacctga gtcgctgtct ttttcgtgac
4381 attcagttcg ctgcgctcac ggctctggca gtgaatgggg gtaaatggca ctacaggcgc
4441 cttttatgga ttcatgcaag gaaactaccc ataatacaag aaaagcccgt cacgggcttc
4501 tcagggcgtt ttatggcggg tctgctatgt ggtgctatct gacttttttgc tgttcagcag
4561 ttcctgccct ctgattttcc agtctgacca cttcggatta tcccgtgaca ggtcattcag
4621 actggctaat gcacccagta aggcagcggt atcatcaacg ggtctgacg ctcagtggaa
4681 cgaaaactca cgttaaggga ttttggtcat gagattatca aaaaggatct tcacctagat

4741 ccttttaaat taaaaatgaa gttttaaatc aatctaaagt atatatgagt aaacttggtc
4801 tgacagttac caatgcttaa tcagtgaggc acctatctca gcgatctgtc tatttcgttc
4861 atccatagtt gcctgactcc ccgtcgtgta gataactacg atacgggagg gcttaccatc
4921 tggccccagt gctgcaatga taccgcgaga cccacgctca ccggctccag atttatcagc
4981 aataaaccag ccagccggaa gggccgagcg cagaagtggt cctgcaactt tatccgcctc
5041 catccagtct attaattgtt gccgggaagc tagagtaagt agttcgccag ttaatagttt
5101 gcgcaacgtt gttgccattg ctacaggcat cgtggtgtca cgctcgtcgt ttggtatggc
5161 ttcattcagc tccggttccc aacgatcaag gcgagttaca tgatccccca tgttgtgcaa
5221 aaaagcggtt agctccttcg gtcctccgat cgttgtcaga agtaagttgg ccgcagtgtt
5281 atcactcatg gttatggcag cactgcataa ttctcttact gtcatgccat ccgtaagatg
5341 cttttctgtg actggtgagt actcaaccaa gtcattctga gaatagtgta tgcggcgacc
5401 gagttgctct tgcccggcgt caatacggga taataccgcg ccacatagca gaactttaaa
5461 agtgctcatc attggaaaac gttcttcggg gcgaaaactc tcaaggatct taccgctgtt
5521 gagatccagt tcgatgtaac ccactcgtgc acccaactga tcttcagcat cttttacttt
5581 caccagcgtt tctgggtgag caaaaacagg aaggcaaaat gccgcaaaaa agggaataag
5641 ggcgacacgg aaatgttgaa tactcatact cttccttttt caatattatt gaagcattta
5701 tcagggttat tgtctcatga gcggatacat atttgaatgt atttagaaaa ataaacaaat
5761 aggggttccg cgcacatttc cccgaaaagt gccacctg
//

```
LOCUS       pGSs038                 5390 bp    DNA     circular UNA 26-
OCT-2015
DEFINITION  CRIM helper plasmid pINT-ts, complete sequence.
ACCESSION   urn.local...42-exvb5az
VERSION     urn.local...42-exvb5az
KEYWORDS    .
SOURCE      CRIM helper plasmid pINT-ts (CRIM helper plasmid pINT-ts.)
  ORGANISM  CRIM helper plasmid pINT-ts
            other sequences;artificial sequences;vectors.
FEATURES             Location/Qualifiers
     misc_feature    5390
                     /Description="dimer interface [polypeptide binding];
other
                     site"
                     /db_xref="CDD:238415"
                     /gene="int"
                     /label="int"
     misc_feature    5390
                     /Description="active site"
                     /db_xref="CDD:238415"
                     /gene="int"
                     /label="int"
     misc_feature    5390
                     /Description="Int/Topo IB signature motif; other
site"
                     /db_xref="CDD:238415"
                     /gene="int"
                     /label="int"
     CDS             complement(62..775)
                     /db_xref="GI:16209182"
                     /product="cI"
                     /codon_start=1
                     /protein_id="AAL09915.1"
                     /transl_table=11
                     /label="CDS_1"
     misc_feature    complement(74..712)
                     /Description="SOS-response transcriptional
repressors
                     (RecA-mediated autopeptidases) [Transcription /
Signal
                     transduction mechanisms]; Region: LexA; COG1974"
                     /db_xref="CDD:224885"
                     /label="Misc_Feature_3"
     misc_feature    complement(95..352)
                     /Description="Peptidase S24 LexA-like proteins are
                     involved in the SOS response leading to the repair
of
                     single-stranded DNA within the bacterial cell. This
family
                     includes: the lambda repressor CI/C2 family and
related
                     bacterial prophage repressor proteins; LexA (EC...;
                     Region: S24_LexA-like; cd06529"
                     /db_xref="CDD:119397"
```

```
                     /label="Misc_Feature_1"
     misc_feature    complement(197..328)
                     /Description="Catalytic site [active]"
                     /db_xref="CDD:119397"
                     /label="Misc_Feature_4"
     misc_feature    complement(545..730)
                     /Description="Helix-turn-helix XRE-family like
proteins.
                     Prokaryotic DNA binding proteins belonging to the
                     xenobiotic response element family of
transcriptional
                     regulators; Region: HTH_XRE; cd00093"
                     /db_xref="CDD:238045"
                     /label="Misc_Feature_2"
     misc_feature    complement(614..676)
                     /Description="sequence-specific DNA binding site
                     [nucleotide binding]; other site"
                     /db_xref="CDD:238045"
                     /label="Misc_Feature_6"
     misc_feature    complement(617..718)
                     /Description="non-specific DNA binding site
[nucleotide
                     binding]; other site"
                     /db_xref="CDD:238045"
                     /label="Misc_Feature_5"
     misc_feature    complement(620..709)
                     /Description="salt bridge; other site"
                     /db_xref="CDD:238045"
                     /label="Misc_Feature_7"
     misc_feature    878..2449
                     /label="PhiRv1int-attB_pInt-Ts"
     CDS             complement(2557..3507)
                     /db_xref="GI:16209184"
                     /product="repA101"
                     /codon_start=1
                     /protein_id="AAL09917.1"
                     /transl_table=11
                     /label="CDS_2"
     misc_feature    complement(2845..3504)
                     /Description="Initiator Replication protein; Region:
                     Rep_3; pfam01051"
                     /db_xref="CDD:250324"
                     /label="Misc_Feature_8"
     CDS             complement(4399..5259)
                     /db_xref="GI:16209185"
                     /product="bla"
                     /codon_start=1
                     /protein_id="AAL09918.1"
                     /transl_table=11
                     /label="CDS_3"
     misc_feature    complement(4402..5253)
                     /Description="beta-lactamase TEM; Provisional;
Region:
                     PRK15442"
```

```
                     /db_xref="CDD:185339"
                     /label="Misc_Feature_9"
ORIGIN
        1 catcgatgaa gattcttgct caattgttat cagctatgcg ccgaccagaa
caccttgccg
       61 atcagccaaa cgtctcttca ggccactgac tagcgataac tttccccaca
acggaacaac
      121 tctcattgca tgggatcatt gggtactgtg ggtttagtgg ttgtaaaaac
acctgaccgc
      181 tatccctgat cagtttcttg aaggtaaact catcaccccc aagtctggct
atgcagaaat
      241 cacctggctc aacagcctgc tcagggtcaa cgagaattaa cattccgtca
ggaaagcttg
      301 gcttggagcc tgttggtgcg gtcatggaat taccttcaac ctcaagccag
aatgcagaat
      361 cactggcttt tttggttgtg cttacccatc tctccgcatc acctttggta
aaggttctaa
      421 gcttaggtga gaacatccct gcctgaacat gagaaaaaac agggtactca
tactcacttc
      481 taagtgacgg ctgcatacta accgcttcat acatctcgta gatttctctg
gcgattgaag
      541 ggctaaattc ttcaacgcta actttgagaa tttttgtaag caatgcggcg
ttataagcat
      601 ttaatgcatt gatgccatta aataaagcac caacgcctga ctgccccatc
cccatcttgt
      661 ctgcgacaga ttcctgggat aagccaagtt catttttctt tttttcataa
attgctttaa
      721 ggcgacgtgc gtcctcaagc tgctcttgtg ttaatggttt ctttttgtg
ctcatacgtt
      781 aaatctatca ccgcaaggga taaatatcta acaccgtgcg tgttgactat
tttacctctg
      841 gcggtgataa tggttgcatg tactaaggag gttgtatgaa ttcgagctcg
gtacccagtt
      901 cgtcgatggg ttcgggaatg caggatattc ccacctccgg ttaaggatgg
aagagagtat
      961 ctgttccacg aatcagcggt aaaggttgac ttaaatcgac cagtaacagg
tggccttttg
     1021 aagaggactg cagatgcgtt acaccacccc ggttcgtgcg gcggtgtatc
tgcgtatcag
     1081 cgaggaccgt agcggtgaac agctgggcgt tgcgcgtcaa cgtgaggatt
gcctgaagct
     1141 gtgcggtcag cgtaaatggg ttccggtgga atacctggac aacgatgtta
gcgcgagcac
     1201 cggcaagcgt cgtccggcgt atgagcaaat gctggcggac atcaccgcgg
gcaaaattgc
     1261 ggcggtggtt gcgtgggacc tggatcgtct gcaccgtcgt ccgattgagc
tggaagcgtt
     1321 tatgagcctg gcggatgaaa agcgtctggc gctggcgacc gttgcgggtg
atgtggatct
     1381 ggcgaccccg cagggtcgtc tggttgcgcg tctgaaaggc agcgtggcgg
cgcatgagac
     1441 cgaacacaag aaagcgcgtc aacgtcgtgc ggcgcgtcaa aaggcggagc
gtggtcaccc
```

1501 gaactggagc aaagcgttcg gctacctgcc gggtccgaac ggtccggaac cggacccgcg
1561 taccgcgccg ctggttaagc aggcgtatgc ggatatcctg gcgggtgcga gcctgggtga
1621 tgtgtgccgt caatggaacg atgcgggtgc gttcaccatt accggtcgtc cgtggaccac
1681 caccaccctg agcaagtttc tgcgtaaacc gcgtaacgcg ggtctgcgtg cgtacaaggg
1741 tgcgcgttat ggcccggttg accgtgatgc gatcgtgggc aaagcgcaat ggagcccgct
1801 ggttgacgag gcgacctttt gggcggcgca agcggttctg gatgcgccgg gtcgtgcgcc
1861 gggtcgtaag agcgttcgtc gtcacctgct gaccggtctg gcgggttgcg gtaaatgcgg
1921 taaccatctg gcgggcagct accgtaccga cggtcaggtg gtttatgttt gcaaggcgtg
1981 ccatggtgtg gcgatcctgg cggataacat cgagccgatt ctgtaccaca ttgttgcgga
2041 acgtctggcg atgccggatg cggtggatct gctgcgtcgt gagatccatg atgcggcgga
2101 ggcggaaacc attcgtctgg agctggaaac cctgtatggt gaactggatc gtctggcggt
2161 ggagcgtgcg gaaggtctgc tgaccgcgcg tcaggttaag atcagcaccg acattgtgaa
2221 cgcgaagatc accaaactgc aagcgcgtca gcaagaccaa gagcgtctgc gtgttttcga
2281 tggtatcccg ctgggtaccc cgcaagtggc gggtatgatt gcggagctga gcccggaccg
2341 ttttcgtgcg gttctggatg tgctggcgga agtggttgtg cagccggtgg gtaaaagcgg
2401 ccgtattttc aacccggaac gtgttcaagt gaactggcgt taagctagcc catgggtatg
2461 gacagttttc cctttgatat gtaacggtga acagttgttc tacttttgtt tgttagtctt
2521 gatgcttcac tgatagatac aagagccata agaacctcag atccttccgt atttagccag
2581 tatgttctct agtgtggttc gttgttttg cgtgagccat gagaacgaac cattgagatc
2641 atacttactt tgcatgtcac tcaaaaattt tgcctcaaaa ctggtgagct gaatttttgc
2701 agttaaagca tcgtgtagtg tttttcttag tccgttacgt aggtaggaat ctgatgtaat
2761 ggttgttggt attttgtcac cattcatttt tatctggttg ttctcaagtt cggttacgag
2821 atccatttgt ctatctagtt caacttggaa aatcaacgta tcagtcgggc ggcctcgctt
2881 atcaaccacc aatttcatat tgctgtaagt gtttaaatct ttacttattg gtttcaaaac
2941 ccattggtta agccttttaa actcatggta gttattttca agcattaaca tgaacttaaa
3001 ttcatcaagg ctaatctcta tatttgcctt gtgagttttc ttttgtgtta gttcttttaa
3061 taaccactca taaatcctca tagagtattt gttttcaaaa gacttaacat gttccagatt

3121 atattttatg aattttttta actggaaaag ataaggcaat atctcttcac taaaaactaa
3181 ttctaatttt tcgcttgaga acttggcata gtttgtccac tggaaaatct caaagccttt
3241 aaccaaagga ttcctgattt ccacagttct cgtcatcagc tctctggttg ctttagctaa
3301 tacaccataa gcattttccc tactgatgtt catcatctga gcgtattggt tataagtgaa
3361 cgataccgtc cgttctttcc ttgtagggtt ttcaatcgtg gggttgagta gtgccacaca
3421 gcataaaatt agcttggttt catgctccgt taagtcatag cgactaatcg ctagttcatt
3481 tgctttgaaa acaactaatt cagacataca tctcaattgg tctaggtgat tttaatcact
3541 ataccaattg agatgggcta gtcaatgata attactagtc cttttccttt gagttgtggg
3601 tatctgtaaa ttctgctaga cctttgctgg aaaacttgta aattctgcta gaccctctgt
3661 aaattccgct agacctttgt gtgttttttt tgtttatatt caagtggtta taatttatag
3721 aataaagaaa gaataaaaaa agataaaaag aatagatccc agccctgtgt ataactcact
3781 actttagtca gttccgcagt attacaaaag gatgtcgcaa acgctgtttg ctcctctaca
3841 aaacagacct taaaacccta aaggcttaag tagcaccctc gcaagctcgg ttgcggccgc
3901 aatcgggcaa atcgctgaat attccttttg tctccgacca tcaggcacct gagtcgctgt
3961 ctttttcgtg acattcagtt cgctgcgctc acggctctgg cagtgaatgg gggtaaatgg
4021 cactacaggc gccttttatg gattcatgca aggaaactac ccataataca agaaaagccc
4081 gtcacgggct tctcagggcg ttttatggcg ggtctgctat gtggtgctat ctgacttttt
4141 gctgttcagc agttcctgcc ctctgatttt ccagtctgac cacttcggat tatcccgtga
4201 caggtcattc agactggcta atgcacccag taaggcagcg gtatcatcaa cggggtctga
4261 cgctcagtgg aacgaaaact cacgttaagg gattttggtc atgagattat caaaaaggat
4321 cttcacctag atccttttaa attaaaaatg aagttttaaa tcaatctaaa gtatatatga
4381 gtaaacttgg tctgacagtt accaatgctt aatcagtgag gcacctatct cagcgatctg
4441 tctatttcgt tcatccatag ttgcctgact ccccgtcgtg tagataacta cgatacggga
4501 gggcttacca tctggcccca gtgctgcaat gataccgcga gacccacgct caccggctcc
4561 agatttatca gcaataaacc agccagccgg aagggccgag cgcagaagtg gtcctgcaac
4621 tttatccgcc tccatccagt ctattaattg ttgccgggaa gctagagtaa gtagttcgcc
4681 agttaatagt ttgcgcaacg ttgttgccat tgctacaggc atcgtggtgt cacgctcgtc

4741 gtttggtatg gcttcattca gctccggttc ccaacgatca aggcgagtta catgatcccc
4801 catgttgtgc aaaaaagcgg ttagctcctt cggtcctccg atcgttgtca gaagtaagtt
4861 ggccgcagtg ttatcactca tggttatggc agcactgcat aattctctta ctgtcatgcc
4921 atccgtaaga tgcttttctg tgactggtga gtactcaacc aagtcattct gagaatagtg
4981 tatgcggcga ccgagttgct cttgcccggc gtcaatacgg gataataccg cgccacatag
5041 cagaacttta aaagtgctca tcattggaaa acgttcttcg gggcgaaaac tctcaaggat
5101 cttaccgctg ttgagatcca gttcgatgta acccactcgt gcacccaact gatcttcagc
5161 atcttttact ttcaccagcg tttctgggtg agcaaaaaca ggaaggcaaa atgccgcaaa
5221 aaagggaata agggcgacac ggaaatgttg aatactcata ctcttccttt ttcaatatta
5281 ttgaagcatt tatcagggtt attgtctcat gagcggatac atatttgaat gtatttagaa
5341 aaataaacaa ataggggttc cgcgcacatt tccccgaaaa gtgccacctg
//

```
LOCUS       pGSs040                 5840 bp    DNA     circular UNA 26-
OCT-2015
DEFINITION  CRIM helper plasmid pINT-ts, complete sequence.
ACCESSION   urn.local...40-exvb5ay
VERSION     urn.local...40-exvb5ay
KEYWORDS    .
SOURCE      CRIM helper plasmid pINT-ts (CRIM helper plasmid pINT-ts.)
  ORGANISM  CRIM helper plasmid pINT-ts
            other sequences;artificial sequences;vectors.
FEATURES             Location/Qualifiers
     misc_feature    5840
                     /Description="dimer interface [polypeptide binding];
other
                     site"
                     /db_xref="CDD:238415"
                     /gene="int"
                     /label="int"
     misc_feature    5840
                     /Description="active site"
                     /db_xref="CDD:238415"
                     /gene="int"
                     /label="int"
     misc_feature    5840
                     /Description="Int/Topo IB signature motif; other
site"
                     /db_xref="CDD:238415"
                     /gene="int"
                     /label="int"
     CDS             complement(62..775)
                     /db_xref="GI:16209182"
                     /product="cI"
                     /codon_start=1
                     /protein_id="AAL09915.1"
                     /transl_table=11
                     /label="CDS_1"
     misc_feature    complement(74..712)
                     /Description="SOS-response transcriptional
repressors
                     (RecA-mediated autopeptidases) [Transcription /
Signal
                     transduction mechanisms]; Region: LexA; COG1974"
                     /db_xref="CDD:224885"
                     /label="Misc_Feature_3"
     misc_feature    complement(95..352)
                     /Description="Peptidase S24 LexA-like proteins are
                     involved in the SOS response leading to the repair
of
                     single-stranded DNA within the bacterial cell. This
family
                     includes: the lambda repressor CI/C2 family and
related
                     bacterial prophage repressor proteins; LexA (EC...;
                     Region: S24_LexA-like; cd06529"
                     /db_xref="CDD:119397"
```

```
                     /label="Misc_Feature_1"
     misc_feature    complement(197..328)
                     /Description="Catalytic site [active]"
                     /db_xref="CDD:119397"
                     /label="Misc_Feature_4"
     misc_feature    complement(545..730)
                     /Description="Helix-turn-helix XRE-family like
proteins.
                     Prokaryotic DNA binding proteins belonging to the
                     xenobiotic response element family of
transcriptional
                     regulators; Region: HTH_XRE; cd00093"
                     /db_xref="CDD:238045"
                     /label="Misc_Feature_2"
     misc_feature    complement(614..676)
                     /Description="sequence-specific DNA binding site
                     [nucleotide binding]; other site"
                     /db_xref="CDD:238045"
                     /label="Misc_Feature_6"
     misc_feature    complement(617..718)
                     /Description="non-specific DNA binding site
[nucleotide
                     binding]; other site"
                     /db_xref="CDD:238045"
                     /label="Misc_Feature_5"
     misc_feature    complement(620..709)
                     /Description="salt bridge; other site"
                     /db_xref="CDD:238045"
                     /label="Misc_Feature_7"
     misc_feature    878..2899
                     /label="TG1int-attB_pInt-Ts"
     CDS             complement(3007..3957)
                     /db_xref="GI:16209184"
                     /product="repA101"
                     /codon_start=1
                     /protein_id="AAL09917.1"
                     /transl_table=11
                     /label="CDS_2"
     misc_feature    complement(3295..3954)
                     /Description="Initiator Replication protein; Region:
                     Rep_3; pfam01051"
                     /db_xref="CDD:250324"
                     /label="Misc_Feature_8"
     CDS             complement(4849..5709)
                     /db_xref="GI:16209185"
                     /product="bla"
                     /codon_start=1
                     /protein_id="AAL09918.1"
                     /transl_table=11
                     /label="CDS_3"
     misc_feature    complement(4852..5703)
                     /Description="beta-lactamase TEM; Provisional;
Region:
                     PRK15442"
```

/db_xref="CDD:185339"
/label="Misc_Feature_9"
ORIGIN
1 catcgatgaa gattcttgct caattgttat cagctatgcg ccgaccagaa caccttgccg
61 atcagccaaa cgtctcttca ggccactgac tagcgataac tttccccaca acggaacaac
121 tctcattgca tgggatcatt gggtactgtg ggtttagtgg ttgtaaaaac acctgaccgc
181 tatccctgat cagtttcttg aaggtaaact catcaccccc aagtctggct atgcagaaat
241 cacctggctc aacagcctgc tcagggtcaa cgagaattaa cattccgtca ggaaagcttg
301 gcttggagcc tgttggtgcg gtcatggaat taccttcaac ctcaagccag aatgcagaat
361 cactggcttt tttggttgtg cttacccatc tctccgcatc acctttggta aaggttctaa
421 gcttaggtga gaacatccct gcctgaacat gagaaaaaac agggtactca tactcacttc
481 taagtgacgg ctgcatacta accgcttcat acatctcgta gatttctctg gcgattgaag
541 ggctaaattc ttcaacgcta actttgagaa tttttgtaag caatgcggcg ttataagcat
601 ttaatgcatt gatgccatta aataaagcac caacgcctga ctgccccatc cccatcttgt
661 ctgcgacaga ttcctgggat aagccaagtt catttttctt tttttcataa attgctttaa
721 ggcgacgtgc gtcctcaagc tgctcttgtg ttaatggttt ctttttttgtg ctcatacgtt
781 aaatctatca ccgcaaggga taaatatcta acaccgtgcg tgttgactat tttacctctg
841 gcggtgataa tggttgcatg tactaaggag gttgtatgaa ttcgagctcg gtacccagtt
901 cgtcgatggg ttcgggaatg caggatattc ccacctccgg ttaaggatgg aagagagtat
961 ctgttccacg aatcagcggt aaaggttgac ttaaatcgac cagtaacagg tggccttttg
1021 aagaggactg cagatggtga ttctggcggg tggctacgac cgtcagagcg cggagcgtga
1081 aaacagcagc accgcgagcc cggcgaccca acgtgcggcg aaccgtggca aggcggaagc
1141 gctggcgaaa gagtatgcgc gtgatggtgt ggaagttaaa tggctgggtc acttcagcga
1201 ggcgccgggt accagcgcgt tcaccggcgt ggaccgtccg gaatttaacc gtattctgga
1261 tatgtgccgt aaccgtgaga tgaacatgat cattgttcac tacatcagcc gtctgagccg
1321 tgaggaaccg ctggacatca ttccggtggt taccgaactg ctgcgtctgg gtgtgaccat
1381 tgtgagcgtt aacgaaggta ccttccgtcc gggcgagatg atggacctga tccacctgat
1441 tatgcgtctg caagcgagcc acgatgaaag caagaacaaa agcgtggcgg ttagcaacgc

1501 gaaggagctg gcgaaacgtc tgggtggcca caccggtagc accccgtacg gctttgacac
1561 cgtggaggaa atggttccga acccggagga tggtggcaaa ctggtggcga tccgtcgtct
1621 ggttccgagc gcgcacacct gggaaggtgc gcatggtagc gagggtgcgg ttattcgttg
1681 ggcgtggcag gaaatcaaga cccaccgtga caccccgttc aaaggtggcg gtgcgggtag
1741 ctttcatccg ggtagcctga acggtctgtg cgagcgtctg tatcgtgata aggtgccgac
1801 ccgtggtacc ctggttggca agaaacgtgc gggtagcgac tgggacccgg gtgtgctgaa
1861 acgtgttctg agcgacccgc gtattgcggg ttaccaagcg gacatcgcgt ataaagtgcg
1921 tgcggatggc agccgtggcg gtttcagcca ctacaaaatt cgtcgtgatc cggttaccat
1981 ggaaccgctg accctgccgg gttttgagcc gtatatcccg ccggcggagt ggtgggaact
2041 gcaagagtgg ctgcaaggcc gtggtcgtgg caagggccag taccgtggcc aaagcctgct
2101 gagcgcgatg gacgtgctgt actgctatgg cagcggtcag ctggacccgg aaaccggtta
2161 cagcaacggc agcaccatgg cgggtaacgt tcgtgagggc gaccaagcgc acaagagcag
2221 ctatgcgtgc aaatgcccgc gtcgtgtgca cgacggtagc agctgtagca ttaccatgca
2281 caacctggac ccgtacatcg ttggcgcgat tttcgcgcgt attaccgcgt ttgacccggc
2341 ggacccggat gatctggaag gtgataccgc ggcgctgatg tatgaagcgg cgcgtcgttg
2401 gggtgcgacc cacgaacgtc cggagctgaa aggccagcgt agcgaactga tggcgcaacg
2461 tgcggacgcg gttaaggcgc tggaggaact gtacgaggat aaacgtaacg gcggttatcg
2521 tagcgcgatg ggtcgtcgtg cgttcctgga ggaagaggcg gcgctgaccc tgcgtatgga
2581 gggtgcggaa gagcgtctgc gtcaactgga tgcggcggat agcccggtgc tgccgattgg
2641 cgagtggctg ggtgaccgtg gcagcgatcc gaccggtccg ggttcctggt gggcgctggc
2701 gccgctggaa gaccgtcgtg cgttcgtgcg tctgtttgtt gatcgtatcg aggttattaa
2761 gctgccgaaa ggtgtgcaac gtccgggtcg tgttccgccg attgcggacc gtgtgcgtat
2821 ccactgggcg aagccgaaag ttgaagagga aaccgagccg gaaaccctga acggttttac
2881 cgcggcggcg taagctagcc catgggtatg gacagttttc cctttgatat gtaacggtga
2941 acagttgttc tacttttgtt tgttagtctt gatgcttcac tgatagatac aagagccata
3001 agaacctcag atccttccgt atttagccag tatgttctct agtgtggttc gttgtttttg
3061 cgtgagccat gagaacgaac cattgagatc atacttactt tgcatgtcac tcaaaaattt

3121 tgcctcaaaa ctggtgagct gaatttttgc agttaaagca tcgtgtagtg tttttcttag
3181 tccgttacgt aggtaggaat ctgatgtaat ggttgttggt attttgtcac cattcatttt
3241 tatctggttg ttctcaagtt cggttacgag atccatttgt ctatctagtt caacttggaa
3301 aatcaacgta tcagtcgggc ggcctcgctt atcaaccacc aatttcatat tgctgtaagt
3361 gtttaaatct ttacttattg gtttcaaaac ccattggtta agccttttaa actcatggta
3421 gttattttca agcattaaca tgaacttaaa ttcatcaagg ctaatctcta tatttgcctt
3481 gtgagttttc ttttgtgtta gttcttttaa taaccactca taaatcctca tagagtattt
3541 gttttcaaaa gacttaacat gttccagatt atattttatg aattttttta actggaaaag
3601 ataaggcaat atctcttcac taaaaactaa ttctaatttt tcgcttgaga acttggcata
3661 gtttgtccac tggaaaatct caaagccttt aaccaaagga ttcctgattt ccacagttct
3721 cgtcatcagc tctctggttg ctttagctaa tacaccataa gcattttccc tactgatgtt
3781 catcatctga gcgtattggt tataagtgaa cgataccgtc cgttctttcc ttgtagggtt
3841 ttcaatcgtg gggttgagta gtgccacaca gcataaaatt agcttggttt catgctccgt
3901 taagtcatag cgactaatcg ctagttcatt tgctttgaaa acaactaatt cagacataca
3961 tctcaattgg tctaggtgat tttaatcact ataccaattg agatgggcta gtcaatgata
4021 attactagtc cttttccttt gagttgtggg tatctgtaaa ttctgctaga cctttgctgg
4081 aaaacttgta aattctgcta gaccctctgt aaattccgct agacctttgt gtgttttttt
4141 tgtttatatt caagtggtta taatttatag aataaagaaa gaataaaaaa agataaaaag
4201 aatagatccc agccctgtgt ataactcact actttagtca gttccgcagt attacaaaag
4261 gatgtcgcaa acgctgtttg ctcctctaca aaacagacct taaaacccta aaggcttaag
4321 tagcaccctc gcaagctcgg ttgcggccgc aatcgggcaa atcgctgaat attccttttg
4381 tctccgacca tcaggcacct gagtcgctgt ctttttcgtg acattcagtt cgctgcgctc
4441 acggctctgg cagtgaatgg gggtaaatgg cactacaggc gccttttatg gattcatgca
4501 aggaaactac ccataataca agaaaagccc gtcacgggct tctcagggcg ttttatggcg
4561 ggtctgctat gtggtgctat ctgacttttt gctgttcagc agttcctgcc ctctgatttt
4621 ccagtctgac cacttcggat tatcccgtga caggtcattc agactggcta atgcacccag
4681 taaggcagcg gtatcatcaa cggggtctga cgctcagtgg aacgaaaact cacgttaagg

4741 gattttggtc atgagattat caaaaaggat cttcacctag atccttttaa
attaaaaatg
4801 aagttttaaa tcaatctaaa gtatatatga gtaaacttgg tctgacagtt
accaatgctt
4861 aatcagtgag gcacctatct cagcgatctg tctatttcgt tcatccatag
ttgcctgact
4921 ccccgtcgtg tagataacta cgatacggga gggcttacca tctggcccca
gtgctgcaat
4981 gataccgcga gacccacgct caccggctcc agatttatca gcaataaacc
agccagccgg
5041 aagggccgag cgcagaagtg gtcctgcaac tttatccgcc tccatccagt
ctattaattg
5101 ttgccgggaa gctagagtaa gtagttcgcc agttaatagt ttgcgcaacg
ttgttgccat
5161 tgctacaggc atcgtggtgt cacgctcgtc gtttggtatg gcttcattca
gctccggttc
5221 ccaacgatca aggcgagtta catgatcccc catgttgtgc aaaaaagcgg
ttagctcctt
5281 cggtcctccg atcgttgtca gaagtaagtt ggccgcagtg ttatcactca
tggttatggc
5341 agcactgcat aattctctta ctgtcatgcc atccgtaaga tgcttttctg
tgactggtga
5401 gtactcaacc aagtcattct gagaatagtg tatgcggcga ccgagttgct
cttgcccggc
5461 gtcaatacgg gataataccg cgccacatag cagaacttta aaagtgctca
tcattggaaa
5521 acgttcttcg gggcgaaaac tctcaaggat cttaccgctg ttgagatcca
gttcgatgta
5581 acccactcgt gcacccaact gatcttcagc atcttttact ttcaccagcg
tttctgggtg
5641 agcaaaaaca ggaaggcaaa atgccgcaaa aaagggaata agggcgacac
ggaaatgttg
5701 aatactcata ctcttccttt ttcaatatta ttgaagcatt tatcagggtt
attgtctcat
5761 gagcggatac atatttgaat gtatttagaa aaataaacaa ataggggttc
cgcgcacatt
5821 tccccgaaaa gtgccacctg
//

```
LOCUS       pGSs041                 5483 bp    DNA     circular UNA 26-
OCT-2015
DEFINITION  CRIM helper plasmid pINT-ts, complete sequence.
ACCESSION   urn.local...3z-exvb5ay
VERSION     urn.local...3z-exvb5ay
KEYWORDS    .
SOURCE      CRIM helper plasmid pINT-ts (CRIM helper plasmid pINT-ts.)
  ORGANISM  CRIM helper plasmid pINT-ts
            other sequences;artificial sequences;vectors.
FEATURES             Location/Qualifiers
     misc_feature    5483
                     /Description="dimer interface [polypeptide binding];
other
                     site"
                     /db_xref="CDD:238415"
                     /gene="int"
                     /label="int"
     misc_feature    5483
                     /Description="active site"
                     /db_xref="CDD:238415"
                     /gene="int"
                     /label="int"
     misc_feature    5483
                     /Description="Int/Topo IB signature motif; other
site"
                     /db_xref="CDD:238415"
                     /gene="int"
                     /label="int"
     CDS             complement(62..775)
                     /db_xref="GI:16209182"
                     /product="cI"
                     /codon_start=1
                     /protein_id="AAL09915.1"
                     /transl_table=11
                     /label="CDS_1"
     misc_feature    complement(74..712)
                     /Description="SOS-response transcriptional
repressors
                     (RecA-mediated autopeptidases) [Transcription /
Signal
                     transduction mechanisms]; Region: LexA; COG1974"
                     /db_xref="CDD:224885"
                     /label="Misc_Feature_3"
     misc_feature    complement(95..352)
                     /Description="Peptidase S24 LexA-like proteins are
                     involved in the SOS response leading to the repair
of
                     single-stranded DNA within the bacterial cell. This
family
                     includes: the lambda repressor CI/C2 family and
related
                     bacterial prophage repressor proteins; LexA (EC...;
                     Region: S24_LexA-like; cd06529"
                     /db_xref="CDD:119397"
```

```
                     /label="Misc_Feature_1"
     misc_feature    complement(197..328)
                     /Description="Catalytic site [active]"
                     /db_xref="CDD:119397"
                     /label="Misc_Feature_4"
     misc_feature    complement(545..730)
                     /Description="Helix-turn-helix XRE-family like
proteins.
                     Prokaryotic DNA binding proteins belonging to the
                     xenobiotic response element family of
transcriptional
                     regulators; Region: HTH_XRE; cd00093"
                     /db_xref="CDD:238045"
                     /label="Misc_Feature_2"
     misc_feature    complement(614..676)
                     /Description="sequence-specific DNA binding site
                     [nucleotide binding]; other site"
                     /db_xref="CDD:238045"
                     /label="Misc_Feature_6"
     misc_feature    complement(617..718)
                     /Description="non-specific DNA binding site
[nucleotide
                     binding]; other site"
                     /db_xref="CDD:238045"
                     /label="Misc_Feature_5"
     misc_feature    complement(620..709)
                     /Description="salt bridge; other site"
                     /db_xref="CDD:238045"
                     /label="Misc_Feature_7"
     misc_feature    878..2542
                     /label="BxB1int-attB_pInt-Ts"
     CDS             complement(2650..3600)
                     /db_xref="GI:16209184"
                     /product="repA101"
                     /codon_start=1
                     /protein_id="AAL09917.1"
                     /transl_table=11
                     /label="CDS_2"
     misc_feature    complement(2938..3597)
                     /Description="Initiator Replication protein; Region:
                     Rep_3; pfam01051"
                     /db_xref="CDD:250324"
                     /label="Misc_Feature_8"
     CDS             complement(4492..5352)
                     /db_xref="GI:16209185"
                     /product="bla"
                     /codon_start=1
                     /protein_id="AAL09918.1"
                     /transl_table=11
                     /label="CDS_3"
     misc_feature    complement(4495..5346)
                     /Description="beta-lactamase TEM; Provisional;
Region:
                     PRK15442"
```

/db_xref="CDD:185339"
/label="Misc_Feature_9"

ORIGIN

```
        1 catcgatgaa gattcttgct caattgttat cagctatgcg ccgaccagaa
caccttgccg
       61 atcagccaaa cgtctcttca ggccactgac tagcgataac tttccccaca
acggaacaac
      121 tctcattgca tgggatcatt gggtactgtg ggtttagtgg ttgtaaaaac
acctgaccgc
      181 tatccctgat cagtttcttg aaggtaaact catcaccccc aagtctggct
atgcagaaat
      241 cacctggctc aacagcctgc tcagggtcaa cgagaattaa cattccgtca
ggaaagcttg
      301 gcttggagcc tgttggtgcg gtcatggaat taccttcaac ctcaagccag
aatgcagaat
      361 cactggcttt tttggttgtg cttacccatc tctccgcatc acctttggta
aaggttctaa
      421 gcttaggtga gaacatccct gcctgaacat gagaaaaaac agggtactca
tactcacttc
      481 taagtgacgg ctgcatacta accgcttcat acatctcgta gatttctctg
gcgattgaag
      541 ggctaaattc ttcaacgcta actttgagaa tttttgtaag caatgcggcg
ttataagcat
      601 ttaatgcatt gatgccatta aataaagcac caacgcctga ctgccccatc
cccatcttgt
      661 ctgcgacaga ttcctgggat aagccaagtt catttttctt tttttcataa
attgctttaa
      721 ggcgacgtgc gtcctcaagc tgctcttgtg ttaatggttt ctttttttgtg
ctcatacgtt
      781 aaatctatca ccgcaaggga taaatatcta acaccgtgcg tgttgactat
tttacctctg
      841 gcggtgataa tggttgcatg tactaaggag gttgtatgaa ttcgagctcg
gtacccagtt
      901 cgtcgatggg ttcgggaatg caggatattc ccacctccgg ttaaggatgg
aagagagtat
      961 ctgttccacg aatcagcggt aaaggttgac ttaaatcgac cagtaacagg
tggccttttg
     1021 aagaggactg cagatgcgtg cgctggttgt gatccgtctg agccgtgtta
ccgatgcgac
     1081 caccagcccg gagcgtcagc tggaaagctg ccagcaactg tgcgcgcaac
gtggctggga
     1141 tgtggttggt gtggcggagg atctggatgt gagcggtgcg gttgatccgt
tcgaccgtaa
     1201 acgtcgtccg aacctggcgc gttggctggc gttcgaggaa cagccgtttg
atgtgatcgt
     1261 tgcgtaccgt gttgaccgtc tgacccgtag cattcgtcac ctgcaacaac
tggtgcactg
     1321 ggcggaggac cacaagaaac tggtggttag cgcgaccgaa gcgcacttcg
ataccaccac
     1381 cccgtttgcg gcggtggtta tcgcgctgat gggtaccgtt gcgcaaatgg
agctggaagc
     1441 gatcaaggaa cgtaaccgta gcgcggcgca cttcaacatt cgtgcgggca
aataccgtgg
```

1501 tagcctgccg ccgtggggct atctgccgac ccgtgttgat ggtgaatggc gtctggtgcc
1561 ggacccggtt cagcgtgagc gtattctgga agtgtaccac cgtgtggttg ataaccacga
1621 accgctgcac ctggttgcgc acgacctgaa ccgtcgtggc gtgctgagcc cgaaagatta
1681 ttttgcgcag ctgcaaggcc gtgagccgca aggtcgtgaa tggagcgcga ccgcgctgaa
1741 gcgtagcatg atcagcgagg cgatgctggg ctatgcgacc ctgaacggta aaaccgttcg
1801 tgacgatgat ggtgcgccgc tggtgcgtgc ggagccgatt ctgacccgtg agcagctgga
1861 agcgctgcgt gcggaactgg ttaagaccag ccgtgcgaag ccggcggtgt ctaccccgag
1921 cctgctgctg cgtgttctgt tctgcgcggt ttgcggtgaa ccggcgtaca agtttgcggg
1981 tggcggtcgt aaacacccgc gttatcgttg ccgtagcatg ggtttcccga agcactgcgg
2041 caacggtacc gttgcgatgg cggaatggga cgcgttttgc gaggaacaag tgctggatct
2101 gctgggtgat gcggagcgtc tggaaaaagt gtgggttgcg ggtagcgaca gcgcggtgga
2161 gctggcggaa gttaacgcgg agctggttga cctgaccagc ctgattggca gcccggcgta
2221 tcgtgcgggt agcccgcagc gtgaagcgct ggatgcgcgt attgcggcgc tggcggcgcg
2281 tcaagaggaa ctggagggtc tggaagcgcg tccgagcggt tgggagtggc gtgaaaccgg
2341 ccagcgtttt ggtgattggt ggcgtgagca agacaccgcg gcgaagaaca cctggctgcg
2401 tagcatgaac gttcgtctga ccttcgatgt gcgtggcggt ctgacccgta ccattgattt
2461 tggtgacctg caagagtatg aacaacacct gcgtctgggc agcgtggttg aacgtctgca
2521 caccggtatg agctaagcta gcccatgggt atggacagtt ttccctttga tatgtaacgg
2581 tgaacagttg ttctactttt gtttgttagt cttgatgctt cactgataga tacaagagcc
2641 ataagaacct cagatccttc cgtatttagc cagtatgttc tctagtgtgg ttcgttgttt
2701 ttgcgtgagc catgagaacg aaccattgag atcatactta ctttgcatgt cactcaaaaa
2761 ttttgcctca aaactggtga gctgaatttt tgcagttaaa gcatcgtgta gtgtttttct
2821 tagtccgtta cgtaggtagg aatctgatgt aatggttgtt ggtattttgt caccattcat
2881 ttttatctgg ttgttctcaa gttcggttac gagatccatt tgtctatcta gttcaacttg
2941 gaaaatcaac gtatcagtcg ggcggcctcg cttatcaacc accaatttca tattgctgta
3001 agtgtttaaa tctttactta ttggtttcaa aacccattgg ttaagccttt taaactcatg
3061 gtagttattt tcaagcatta acatgaactt aaattcatca aggctaatct ctatatttgc

3121 cttgtgagtt ttcttttgtg ttagttcttt taataaccac tcataaatcc
tcatagagta
3181 tttgttttca aaagacttaa catgttccag attatatttt atgaattttt
ttaactggaa
3241 aagataaggc aatatctctt cactaaaaac taattctaat tttcgcttg
agaacttggc
3301 atagtttgtc cactggaaaa tctcaaagcc tttaaccaaa ggattcctga
tttccacagt
3361 tctcgtcatc agctctctgg ttgctttagc taatacacca taagcatttt
ccctactgat
3421 gttcatcatc tgagcgtatt ggttataagt gaacgatacc gtccgttctt
tccttgtagg
3481 gttttcaatc gtggggttga gtagtgccac acagcataaa attagcttgg
tttcatgctc
3541 cgttaagtca tagcgactaa tcgctagttc atttgctttg aaaacaacta
attcagacat
3601 acatctcaat tggtctaggt gattttaatc actataccaa ttgagatggg
ctagtcaatg
3661 ataattacta gtccttttcc tttgagttgt gggtatctgt aaattctgct
agacctttgc
3721 tggaaaactt gtaaattctg ctagaccctc tgtaaattcc gctagacctt
tgtgtgtttt
3781 ttttgtttat attcaagtgg ttataattta tagaataaag aaagaataaa
aaaagataaa
3841 aagaatagat cccagccctg tgtataactc actactttag tcagttccgc
agtattacaa
3901 aaggatgtcg caaacgctgt ttgctcctct acaaaacaga ccttaaaacc
ctaaaggctt
3961 aagtagcacc ctcgcaagct cggttgcggc cgcaatcggg caaatcgctg
aatattcctt
4021 ttgtctccga ccatcaggca cctgagtcgc tgtctttttc gtgacattca
gttcgctgcg
4081 ctcacggctc tggcagtgaa tgggggtaaa tggcactaca ggcgcctttt
atggattcat
4141 gcaaggaaac tacccataat acaagaaaag cccgtcacgg gcttctcagg
gcgttttatg
4201 gcgggtctgc tatgtggtgc tatctgactt tttgctgttc agcagttcct
gccctctgat
4261 tttccagtct gaccacttcg gattatcccg tgacaggtca ttcagactgg
ctaatgcacc
4321 cagtaaggca gcggtatcat caacggggtc tgacgctcag tggaacgaaa
actcacgtta
4381 agggattttg gtcatgagat tatcaaaaag gatcttcacc tagatccttt
taaattaaaa
4441 atgaagtttt aaatcaatct aaagtatata tgagtaaact tggtctgaca
gttaccaatg
4501 cttaatcagt gaggcaccta tctcagcgat ctgtctattt cgttcatcca
tagttgcctg
4561 actccccgtc gtgtagataa ctacgatacg ggagggctta ccatctggcc
ccagtgctgc
4621 aatgataccg cgagacccac gctcaccggc tccagattta tcagcaataa
accagccagc
4681 cggaagggcc gagcgcagaa gtggtcctgc aactttatcc gcctccatcc
agtctattaa

4741 ttgttgccgg gaagctagag taagtagttc gccagttaat agtttgcgca
acgttgttgc
4801 cattgctaca ggcatcgtgg tgtcacgctc gtcgtttggt atggcttcat
tcagctccgg
4861 ttcccaacga tcaaggcgag ttacatgatc ccccatgttg tgcaaaaaag
cggttagctc
4921 cttcggtcct ccgatcgttg tcagaagtaa gttggccgca gtgttatcac
tcatggttat
4981 ggcagcactg cataattctc ttactgtcat gccatccgta agatgctttt
ctgtgactgg
5041 tgagtactca accaagtcat tctgagaata gtgtatgcgg cgaccgagtt
gctcttgccc
5101 ggcgtcaata cgggataata ccgcgccaca tagcagaact ttaaaagtgc
tcatcattgg
5161 aaaacgttct tcggggcgaa aactctcaag gatcttaccg ctgttgagat
ccagttcgat
5221 gtaacccact cgtgcaccca actgatcttc agcatctttt actttcacca
gcgtttctgg
5281 gtgagcaaaa acaggaaggc aaaatgccgc aaaaaaggga ataagggcga
cacggaaatg
5341 ttgaatactc atactcttcc tttttcaata ttattgaagc atttatcagg
gttattgtct
5401 catgagcgga tacatatttg aatgtattta gaaaaataaa caaatagggg
ttccgcgcac
5461 atttccccga aaagtgccac ctg
//

```
LOCUS       pGSs042                 5339 bp    DNA     circular UNA 14-
DEC-2015
DEFINITION  CRIM helper plasmid pINT-ts, complete sequence.
ACCESSION   urn.local...3y-exvb5ay
VERSION     urn.local...3y-exvb5ay
KEYWORDS    .
SOURCE      CRIM helper plasmid pINT-ts (CRIM helper plasmid pINT-ts.)
  ORGANISM  CRIM helper plasmid pINT-ts
            other sequences;artificial sequences;vectors.
FEATURES             Location/Qualifiers
     misc_feature    5339
                     /Description="dimer interface [polypeptide binding];
other
                     site"
                     /db_xref="CDD:238415"
                     /gene="int"
                     /label="int"
     misc_feature    5339
                     /Description="active site"
                     /db_xref="CDD:238415"
                     /gene="int"
                     /label="int"
     misc_feature    5339
                     /Description="Int/Topo IB signature motif; other
site"
                     /db_xref="CDD:238415"
                     /gene="int"
                     /label="int"
     CDS             complement(62..775)
                     /db_xref="GI:16209182"
                     /product="cI"
                     /codon_start=1
                     /protein_id="AAL09915.1"
                     /transl_table=11
                     /label="CDS_1"
     misc_feature    complement(74..712)
                     /Description="SOS-response transcriptional
repressors
                     (RecA-mediated autopeptidases) [Transcription /
Signal
                     transduction mechanisms]; Region: LexA; COG1974"
                     /db_xref="CDD:224885"
                     /label="Misc_Feature_3"
     misc_feature    complement(95..352)
                     /Description="Peptidase S24 LexA-like proteins are
                     involved in the SOS response leading to the repair
of
                     single-stranded DNA within the bacterial cell. This
family
                     includes: the lambda repressor CI/C2 family and
related
                     bacterial prophage repressor proteins; LexA (EC...;
                     Region: S24_LexA-like; cd06529"
                     /db_xref="CDD:119397"
```

```
                     /label="Misc_Feature_1"
     misc_feature    complement(197..328)
                     /Description="Catalytic site [active]"
                     /db_xref="CDD:119397"
                     /label="Misc_Feature_4"
     misc_feature    complement(545..730)
                     /Description="Helix-turn-helix XRE-family like
proteins.
                     Prokaryotic DNA binding proteins belonging to the
                     xenobiotic response element family of
transcriptional
                     regulators; Region: HTH_XRE; cd00093"
                     /db_xref="CDD:238045"
                     /label="Misc_Feature_2"
     misc_feature    complement(614..676)
                     /Description="sequence-specific DNA binding site
                     [nucleotide binding]; other site"
                     /db_xref="CDD:238045"
                     /label="Misc_Feature_6"
     misc_feature    complement(617..718)
                     /Description="non-specific DNA binding site
[nucleotide
                     binding]; other site"
                     /db_xref="CDD:238045"
                     /label="Misc_Feature_5"
     misc_feature    complement(620..709)
                     /Description="salt bridge; other site"
                     /db_xref="CDD:238045"
                     /label="Misc_Feature_7"
     misc_feature    878..2398
                     /label="PhiA118int-attB_pInt-Ts"
     CDS             complement(2506..3456)
                     /db_xref="GI:16209184"
                     /product="repA101"
                     /codon_start=1
                     /protein_id="AAL09917.1"
                     /transl_table=11
                     /label="CDS_2"
     misc_feature    complement(2794..3453)
                     /Description="Initiator Replication protein; Region:
                     Rep_3; pfam01051"
                     /db_xref="CDD:250324"
                     /label="Misc_Feature_8"
     CDS             complement(4348..5208)
                     /db_xref="GI:16209185"
                     /product="bla"
                     /codon_start=1
                     /protein_id="AAL09918.1"
                     /transl_table=11
                     /label="CDS_3"
     misc_feature    complement(4351..5202)
                     /Description="beta-lactamase TEM; Provisional;
Region:
                     PRK15442"
```

```
                     /db_xref="CDD:185339"
                     /label="Misc_Feature_9"
ORIGIN
        1 catcgatgaa gattcttgct caattgttat cagctatgcg ccgaccagaa
caccttgccg
       61 atcagccaaa cgtctcttca ggccactgac tagcgataac tttccccaca
acggaacaac
      121 tctcattgca tgggatcatt gggtactgtg ggtttagtgg ttgtaaaaac
acctgaccgc
      181 tatccctgat cagtttcttg aaggtaaact catcaccccc aagtctggct
atgcagaaat
      241 cacctggctc aacagcctgc tcagggtcaa cgagaattaa cattccgtca
ggaaagcttg
      301 gcttggagcc tgttggtgcg gtcatggaat taccttcaac ctcaagccag
aatgcagaat
      361 cactggcttt tttggttgtg cttacccatc tctccgcatc acctttggta
aaggttctaa
      421 gcttaggtga gaacatccct gcctgaacat gagaaaaaac agggtactca
tactcacttc
      481 taagtgacgg ctgcatacta accgcttcat acatctcgta gatttctctg
gcgattgaag
      541 ggctaaattc ttcaacgcta actttgagaa tttttgtaag caatgcggcg
ttataagcat
      601 ttaatgcatt gatgccatta aataaagcac caacgcctga ctgccccatc
cccatcttgt
      661 ctgcgacaga ttcctgggat aagccaagtt catttttctt tttttcataa
attgctttaa
      721 ggcgacgtgc gtcctcaagc tgctcttgtg ttaatggttt ctttttgtg
ctcatacgtt
      781 aaatctatca ccgcaaggga taaatatcta acaccgtgcg tgttgactat
tttacctctg
      841 gcggtgataa tggttgcatg tactaaggag gttgtatgaa ttcgagctcg
gtacccagtt
      901 cgtcgatggg ttcgggaatg caggatattc ccacctccgg ttaaggatgg
aagagagtat
      961 ctgttccacg aatcagcggt aaaggttgac ttaaatcgac cagtaacagg
tggccttttg
     1021 aagaggactg cagatgaagg cggcgatcta tattcgtgtt agcacccagg
agcaagtgga
     1081 aaactacagc atccaggcgc aaaccgagaa gctgaccgcg ctgtgccgta
gcaaagactg
     1141 ggatgtttat gatatcttca ttgacggtgg ctacagcggt agcaacatga
accgtccggc
     1201 gctgaacgag atgctgagca agctgcacga aattgatgcg gtggttgtgt
atcgtctgga
     1261 ccgtctgagc cgtagccagc gtgataccat caccctgatt gaggaatact
tcctgaaaaa
     1321 caacgttgag tttgtgagcc tgagcgaaac cctggacacc agcagcccgt
tcggtcgtgc
     1381 gatgatcggc attctgagcg tttttgcgca actggagcgt gaaaccatcc
gtgatcgtat
     1441 ggtgatgggt aaaatcaagc gtattgaggc gggtctgccg ctgaccaccg
cgaaaggtcg
```

1501 taccttcggc tatgacgtga tcgataccaa gctgtacatt aacgaggaag aggcgaaaca
1561 gctgcaactg atctacgaca ttttcgaaga ggaacagagc atcacctttc tgcaaaagcg
1621 tctgaagaaa ctgggtttta aagttcgtac ctacaaccgt tataacaact ggctgaccaa
1681 cgacctgtac tgcggttatg tgagctacaa agataaggtt cacgtgaagg gcatccacga
1741 gccgatcatt agcgaggaac agttctaccg tgttcaagaa atttttaccc gtatgggcaa
1801 aaacccgaac atgaaccgtg acagcgcgag cctgctgaac aacctggttg tgtgcagcaa
1861 gtgcggtctg ggctttgtgc accgtcgtaa agataccatg agccgtggta agaaatacca
1921 ctatcgttac tatagctgca aaacctataa gcacacccac gagctggaaa aatgcggcaa
1981 caagatctgg cgtgcggaca agctggagga actgatcatt aaccgtgtta acaactacag
2041 cttcgcgagc cgtaacgtgg acaaagagga tgaactggac agcctgaacg agaaactgaa
2101 gatcgaacac gcgaagaaaa agcgtctgtt tgacctgtat attaacggta gctacgaagt
2161 tagcgaactg gatagcatga tgaacgacat cgatgcgcag attaactact atgagagcca
2221 aatcgaagcg aacgaggaac tgaaaaagaa caaaaagatt caggagaacc tggcggacct
2281 ggcgaccgtt gacttcgata gcctggagtt tcgtgaaaaa cagctgtatc tgaagagcct
2341 gatcaacaaa atctacattg atggcgagca agtgaccatt gaatggctgt aagctagccc
2401 atgggtatgg acagttttcc ctttgatatg taacggtgaa cagttgttct acttttgttt
2461 gttagtcttg atgcttcact gatagataca agagccataa gaacctcaga tccttccgta
2521 tttagccagt atgttctcta gtgtggttcg ttgtttttgc gtgagccatg agaacgaacc
2581 attgagatca tacttacttt gcatgtcact caaaaatttt gcctcaaaac tggtgagctg
2641 aatttttgca gttaaagcat cgtgtagtgt ttttcttagt ccgttacgta ggtaggaatc
2701 tgatgtaatg gttgttggta ttttgtcacc attcattttt atctggttgt tctcaagttc
2761 ggttacgaga tccatttgtc tatctagttc aacttggaaa atcaacgtat cagtcgggcg
2821 gcctcgctta tcaaccacca atttcatatt gctgtaagtg tttaaatctt tacttattgg
2881 tttcaaaacc cattggttaa gccttttaaa ctcatggtag ttattttcaa gcattaacat
2941 gaacttaaat tcatcaaggc taatctctat atttgccttg tgagttttct tttgtgttag
3001 ttcttttaat aaccactcat aaatcctcat agagtatttg ttttcaaaag acttaacatg
3061 ttccagatta tattttatga attttttaa ctggaaaaga taaggcaata tctcttcact

3121 aaaaactaat tctaattttt cgcttgagaa cttggcatag tttgtccact
ggaaaatctc
3181 aaagccttta accaaaggat tcctgatttc cacagttctc gtcatcagct
ctctggttgc
3241 tttagctaat acaccataag cattttccct actgatgttc atcatctgag
cgtattggtt
3301 ataagtgaac gataccgtcc gttctttcct tgtagggttt tcaatcgtgg
ggttgagtag
3361 tgccacacag cataaaatta gcttggtttc atgctccgtt aagtcatagc
gactaatcgc
3421 tagttcattt gctttgaaaa caactaattc agacatacat ctcaattggt
ctaggtgatt
3481 ttaatcacta taccaattga gatgggctag tcaatgataa ttactagtcc
ttttcctttg
3541 agttgtgggt atctgtaaat tctgctagac ctttgctgga aaacttgtaa
attctgctag
3601 accctctgta aattccgcta gacctttgtg tgtttttttt gtttatattc
aagtggttat
3661 aatttataga ataaagaaag aataaaaaaa gataaaaaga atagatccca
gccctgtgta
3721 taactcacta ctttagtcag ttccgcagta ttacaaaagg atgtcgcaaa
cgctgtttgc
3781 tcctctacaa aacagacctt aaaaccctaa aggcttaagt agcaccctcg
caagctcggt
3841 tgcggccgca atcgggcaaa tcgctgaata ttccttttgt ctccgaccat
caggcacctg
3901 agtcgctgtc tttttcgtga cattcagttc gctgcgctca cggctctggc
agtgaatggg
3961 ggtaaatggc actacaggcg ccttttatgg attcatgcaa ggaaactacc
cataatacaa
4021 gaaaagcccg tcacgggctt ctcagggcgt tttatggcgg gtctgctatg
tggtgctatc
4081 tgactttttg ctgttcagca gttcctgccc tctgattttc cagtctgacc
acttcggatt
4141 atcccgtgac aggtcattca gactggctaa tgcacccagt aaggcagcgg
tatcatcaac
4201 ggggtctgac gctcagtgga acgaaaactc acgttaaggg attttggtca
tgagattatc
4261 aaaaaggatc ttcacctaga tccttttaaa ttaaaaatga agttttaaat
caatctaaag
4321 tatatatgag taaacttggt ctgacagtta ccaatgctta atcagtgagg
cacctatctc
4381 agcgatctgt ctatttcgtt catccatagt tgcctgactc cccgtcgtgt
agataactac
4441 gatacgggag ggcttaccat ctggccccag tgctgcaatg ataccgcgag
acccacgctc
4501 accggctcca gatttatcag caataaacca gccagccgga agggccgagc
gcagaagtgg
4561 tcctgcaact ttatccgcct ccatccagtc tattaattgt tgccgggaag
ctagagtaag
4621 tagttcgcca gttaatagtt tgcgcaacgt tgttgccatt gctacaggca
tcgtggtgtc
4681 acgctcgtcg tttggtatgg cttcattcag ctccggttcc caacgatcaa
ggcgagttac

4741 atgatccccc atgttgtgca aaaaagcggt tagctccttc ggtcctccga
tcgttgtcag
4801 aagtaagttg gccgcagtgt tatcactcat ggttatggca gcactgcata
attctcttac
4861 tgtcatgcca tccgtaagat gcttttctgt gactggtgag tactcaacca
agtcattctg
4921 agaatagtgt atgcggcgac cgagttgctc ttgcccggcg tcaatacggg
ataataccgc
4981 gccacatagc agaactttaa aagtgctcat cattggaaaa cgttcttcgg
ggcgaaaact
5041 ctcaaggatc ttaccgctgt tgagatccag ttcgatgtaa cccactcgtg
cacccaactg
5101 atcttcagca tcttttactt tcaccagcgt ttctgggtga gcaaaaacag
gaaggcaaaa
5161 tgccgcaaaa aagggaataa gggcgacacg gaaatgttga atactcatac
tcttcctttt
5221 tcaatattat tgaagcattt atcagggtta ttgtctcatg agcggataca
tatttgaatg
5281 tatttagaaa aataaacaaa taggggttcc gcgcacattt ccccgaaaag
tgccacctg
//

```
LOCUS       pGSs043                 5375 bp    DNA     circular UNA 26-
OCT-2015
DEFINITION  CRIM helper plasmid pINT-ts, complete sequence.
ACCESSION   urn.local...3x-exvb5ay
VERSION     urn.local...3x-exvb5ay
KEYWORDS    .
SOURCE      CRIM helper plasmid pINT-ts (CRIM helper plasmid pINT-ts.)
  ORGANISM  CRIM helper plasmid pINT-ts
            other sequences;artificial sequences;vectors.
FEATURES             Location/Qualifiers
     misc_feature    5375
                     /Description="dimer interface [polypeptide binding];
other
                     site"
                     /db_xref="CDD:238415"
                     /gene="int"
                     /label="int"
     misc_feature    5375
                     /Description="active site"
                     /db_xref="CDD:238415"
                     /gene="int"
                     /label="int"
     misc_feature    5375
                     /Description="Int/Topo IB signature motif; other
site"
                     /db_xref="CDD:238415"
                     /gene="int"
                     /label="int"
     CDS             complement(62..775)
                     /db_xref="GI:16209182"
                     /product="cI"
                     /codon_start=1
                     /protein_id="AAL09915.1"
                     /transl_table=11
                     /label="CDS_1"
     misc_feature    complement(74..712)
                     /Description="SOS-response transcriptional
repressors
                     (RecA-mediated autopeptidases) [Transcription /
Signal
                     transduction mechanisms]; Region: LexA; COG1974"
                     /db_xref="CDD:224885"
                     /label="Misc_Feature_3"
     misc_feature    complement(95..352)
                     /Description="Peptidase S24 LexA-like proteins are
                     involved in the SOS response leading to the repair
of
                     single-stranded DNA within the bacterial cell. This
family
                     includes: the lambda repressor CI/C2 family and
related
                     bacterial prophage repressor proteins; LexA (EC...;
                     Region: S24_LexA-like; cd06529"
                     /db_xref="CDD:119397"
```

```
                     /label="Misc_Feature_1"
     misc_feature    complement(197..328)
                     /Description="Catalytic site [active]"
                     /db_xref="CDD:119397"
                     /label="Misc_Feature_4"
     misc_feature    complement(545..730)
                     /Description="Helix-turn-helix XRE-family like
proteins.
                     Prokaryotic DNA binding proteins belonging to the
                     xenobiotic response element family of
transcriptional
                     regulators; Region: HTH_XRE; cd00093"
                     /db_xref="CDD:238045"
                     /label="Misc_Feature_2"
     misc_feature    complement(614..676)
                     /Description="sequence-specific DNA binding site
                     [nucleotide binding]; other site"
                     /db_xref="CDD:238045"
                     /label="Misc_Feature_6"
     misc_feature    complement(617..718)
                     /Description="non-specific DNA binding site
[nucleotide
                     binding]; other site"
                     /db_xref="CDD:238045"
                     /label="Misc_Feature_5"
     misc_feature    complement(620..709)
                     /Description="salt bridge; other site"
                     /db_xref="CDD:238045"
                     /label="Misc_Feature_7"
     misc_feature    878..2434
                     /label="PhiFC1int-attB_pInt-Ts"
     CDS             complement(2542..3492)
                     /db_xref="GI:16209184"
                     /product="repA101"
                     /codon_start=1
                     /protein_id="AAL09917.1"
                     /transl_table=11
                     /label="CDS_2"
     misc_feature    complement(2830..3489)
                     /Description="Initiator Replication protein; Region:
                     Rep_3; pfam01051"
                     /db_xref="CDD:250324"
                     /label="Misc_Feature_8"
     CDS             complement(4384..5244)
                     /db_xref="GI:16209185"
                     /product="bla"
                     /codon_start=1
                     /protein_id="AAL09918.1"
                     /transl_table=11
                     /label="CDS_3"
     misc_feature    complement(4387..5238)
                     /Description="beta-lactamase TEM; Provisional;
Region:
                     PRK15442"
```

/db_xref="CDD:185339"
/label="Misc_Feature_9"

ORIGIN

```
        1 catcgatgaa gattcttgct caattgttat cagctatgcg ccgaccagaa
caccttgccg
       61 atcagccaaa cgtctcttca ggccactgac tagcgataac tttccccaca
acggaacaac
      121 tctcattgca tgggatcatt gggtactgtg ggtttagtgg ttgtaaaaac
acctgaccgc
      181 tatccctgat cagtttcttg aaggtaaact catcaccccc aagtctggct
atgcagaaat
      241 cacctggctc aacagcctgc tcagggtcaa cgagaattaa cattccgtca
ggaaagcttg
      301 gcttggagcc tgttggtgcg gtcatggaat taccttcaac ctcaagccag
aatgcagaat
      361 cactggcttt tttggttgtg cttacccatc tctccgcatc acctttggta
aaggttctaa
      421 gcttaggtga gaacatccct gcctgaacat gagaaaaaac agggtactca
tactcacttc
      481 taagtgacgg ctgcatacta accgcttcat acatctcgta gatttctctg
gcgattgaag
      541 ggctaaattc ttcaacgcta actttgagaa tttttgtaag caatgcggcg
ttataagcat
      601 ttaatgcatt gatgccatta aataaagcac caacgcctga ctgccccatc
cccatcttgt
      661 ctgcgacaga ttcctgggat aagccaagtt catttttctt tttttcataa
attgctttaa
      721 ggcgacgtgc gtcctcaagc tgctcttgtg ttaatggttt ctttttttgtg
ctcatacgtt
      781 aaatctatca ccgcaaggga taaatatcta acaccgtgcg tgttgactat
tttacctctg
      841 gcggtgataa tggttgcatg tactaaggag gttgtatgaa ttcgagctcg
gtacccagtt
      901 cgtcgatggg ttcgggaatg caggatattc ccacctccgg ttaaggatgg
aagagagtat
      961 ctgttccacg aatcagcggt aaaggttgac ttaaatcgac cagtaacagg
tggccttttg
     1021 aagaggactg cagatgaaac gtgcggcgct gtacatccgt gtgagcacca
tggaacaggc
     1081 gaaagagggt tatagcattc cggcgcaaac cgacaagctg aaagcgttcg
cgaaggcgaa
     1141 agacatggcg gtggcgaaag tttacaccga tccgggtttt agcggtgcga
agatggaacg
     1201 tccggcgctg caagagatga tcagcgacat tcaaaacaag aaaatcgatg
tggttctggt
     1261 gtacaaactg gatcgtctga gccgtagcca gaagaacacc ctgtatctga
tcgaagacgt
     1321 gttcctgaag aacaacgttg atttcatcag catgcaggag agcttcgaca
ccagcacccc
     1381 gtttggccgt gcgaccatcg gtatgctgag cgttttcgcg cagctggaac
gtgataccat
     1441 taccgagcgt atgcacatgg gccgtaccga acgtgcgaag caaggttact
atcacggtag
```

1501 cggcatcgtg ccgctgggct acgactatgt tcacggcgag ctgatcatta acgattacga
1561 agcgcagatc attcaagaga tctacgacct gtatgtgaac cagggtaaag gccagcaata
1621 cattaccaaa cgtatggttg cgaagtatcc ggataaggtg aaaaccctga ccatcgttaa
1681 gtacgcgctg accaacccgc tgtatatcgg taaaattagc tgggacggca aggtgtacga
1741 tggtcaccac agcccgatca ttgacaaaag catgtatgat aaggcgcagg aaatcattgc
1801 gcgtatggcg caaaaaggtg gcgagcagca cggtaaccaa ctgggcctgc tgctgggtat
1861 cacctactgc ggcaagtgcg gcgcggaagt gtttcgttat gttagcggtg gcaagaaata
1921 ccgttataac tactatatgt gccgtagcgt gaagaaaatg ctgccgagcc tggttaaaga
1981 ctggaactgc aagcagccga gcctgcgtca agaggtggtt gaaaagaaag tgatcgacag
2041 cctgaaaagc ctggatttca agaaaattga gcgtgaactg aagcaggttg aaaacaagac
2101 caaaagcaag atcaccacca ttaacaacca aatcagcaag aaacacaacg agaaacagaa
2161 gattctggac ctgtaccaat atggcacctt tgatgttacc atgctgaacg aacgtatgaa
2221 gaaaatcgac aacgagatta acgcgctgac cgcgaacatc gcgaacctgg aaggtaccaa
2281 aagcgagagc ctgattaaca aactggaaac cctgaagacc ttcaactggg agaccgaaac
2341 caccgagaac aaaatcctga tcattaagga gttcgtggaa cgtattgagc tgtttgacga
2401 tgaggttatc attaaataca agttttaagc tagcccatgg gtatggacag ttttcccttt
2461 gatatgtaac ggtgaacagt tgttctactt ttgtttgtta gtcttgatgc ttcactgata
2521 gatacaagag ccataagaac ctcagatcct tccgtattta gccagtatgt tctctagtgt
2581 ggttcgttgt ttttgcgtga gccatgagaa cgaaccattg agatcatact tactttgcat
2641 gtcactcaaa aattttgcct caaaactggt gagctgaatt tttgcagtta aagcatcgtg
2701 tagtgttttt cttagtccgt tacgtaggta ggaatctgat gtaatggttg ttggtatttt
2761 gtcaccattc atttttatct ggttgttctc aagttcggtt acgagatcca tttgtctatc
2821 tagttcaact tggaaaatca acgtatcagt cgggcggcct cgcttatcaa ccaccaattt
2881 catattgctg taagtgttta aatctttact tattggtttc aaaacccatt ggttaagcct
2941 tttaaactca tggtagttat tttcaagcat taacatgaac ttaaattcat caaggctaat
3001 ctctatattt gccttgtgag ttttcttttg tgttagttct tttaataacc actcataaat
3061 cctcatagag tatttgtttt caaaagactt aacatgttcc agattatatt ttatgaattt

3121 ttttaactgg aaaagataag gcaatatctc ttcactaaaa actaattcta atttttcgct
3181 tgagaacttg gcatagtttg tccactggaa aatctcaaag cctttaacca aaggattcct
3241 gatttccaca gttctcgtca tcagctctct ggttgcttta gctaatacac cataagcatt
3301 ttccctactg atgttcatca tctgagcgta ttggttataa gtgaacgata ccgtccgttc
3361 tttccttgta gggttttcaa tcgtggggtt gagtagtgcc acacagcata aaattagctt
3421 ggtttcatgc tccgttaagt catagcgact aatcgctagt tcatttgctt tgaaaacaac
3481 taattcagac atacatctca attggtctag gtgattttaa tcactatacc aattgagatg
3541 ggctagtcaa tgataattac tagtcctttt cctttgagtt gtgggtatct gtaaattctg
3601 ctagaccttt gctggaaaac ttgtaaattc tgctagaccc tctgtaaatt ccgctagacc
3661 tttgtgtgtt ttttttgttt atattcaagt ggttataatt tatagaataa agaaagaata
3721 aaaaaagata aaaagaatag atcccagccc tgtgtataac tcactacttt agtcagttcc
3781 gcagtattac aaaaggatgt cgcaaacgct gtttgctcct ctacaaaaca gaccttaaaa
3841 ccctaaaggc ttaagtagca ccctcgcaag ctcggttgcg gccgcaatcg ggcaaatcgc
3901 tgaatattcc ttttgtctcc gaccatcagg cacctgagtc gctgtctttt tcgtgacatt
3961 cagttcgctg cgctcacggc tctggcagtg aatgggggta aatggcacta caggcgcctt
4021 ttatggattc atgcaaggaa actacccata atacaagaaa agcccgtcac gggcttctca
4081 gggcgtttta tggcgggtct gctatgtggt gctatctgac tttttgctgt tcagcagttc
4141 ctgccctctg attttccagt ctgaccactt cggattatcc cgtgacaggt cattcagact
4201 ggctaatgca cccagtaagg cagcggtatc atcaacgggg tctgacgctc agtggaacga
4261 aaactcacgt taagggattt tggtcatgag attatcaaaa aggatcttca cctagatcct
4321 tttaaattaa aaatgaagtt ttaaatcaat ctaaagtata tatgagtaaa cttggtctga
4381 cagttaccaa tgcttaatca gtgaggcacc tatctcagcg atctgtctat ttcgttcatc
4441 catagttgcc tgactccccg tcgtgtagat aactacgata cgggagggct taccatctgg
4501 ccccagtgct gcaatgatac cgcgagaccc acgctcaccg gctccagatt tatcagcaat
4561 aaaccagcca gccggaaggg ccgagcgcag aagtggtcct gcaactttat ccgcctccat
4621 ccagtctatt aattgttgcc gggaagctag agtaagtagt tcgccagtta atagtttgcg
4681 caacgttgtt gccattgcta caggcatcgt ggtgtcacgc tcgtcgtttg gtatggcttc

4741 attcagctcc ggttcccaac gatcaaggcg agttacatga tcccccatgt
tgtgcaaaaa
4801 agcggttagc tccttcggtc ctccgatcgt tgtcagaagt aagttggccg
cagtgttatc
4861 actcatggtt atggcagcac tgcataattc tcttactgtc atgccatccg
taagatgctt
4921 ttctgtgact ggtgagtact caaccaagtc attctgagaa tagtgtatgc
ggcgaccgag
4981 ttgctcttgc ccggcgtcaa tacgggataa taccgcgcca catagcagaa
ctttaaaagt
5041 gctcatcatt ggaaaacgtt cttcggggcg aaaactctca aggatcttac
cgctgttgag
5101 atccagttcg atgtaaccca ctcgtgcacc caactgatct tcagcatctt
ttactttcac
5161 cagcgtttct gggtgagcaa aaacaggaag gcaaaatgcc gcaaaaaagg
gaataagggc
5221 gacacggaaa tgttgaatac tcatactctt cctttttcaa tattattgaa
gcatttatca
5281 gggttattgt ctcatgagcg gatacatatt tgaatgtatt tagaaaaata
aacaaatagg
5341 ggttccgcgc acatttcccc gaaaagtgcc acctg
//

```
LOCUS       pGSs044                 5639 bp    DNA     circular UNA 26-
OCT-2015
DEFINITION  CRIM helper plasmid pINT-ts, complete sequence.
ACCESSION   urn.local...3w-exvb5ay
VERSION     urn.local...3w-exvb5ay
KEYWORDS    .
SOURCE      CRIM helper plasmid pINT-ts (CRIM helper plasmid pINT-ts.)
  ORGANISM  CRIM helper plasmid pINT-ts
            other sequences;artificial sequences;vectors.
FEATURES             Location/Qualifiers
     misc_feature    5639
                     /Description="dimer interface [polypeptide binding];
other
                     site"
                     /db_xref="CDD:238415"
                     /gene="int"
                     /label="int"
     misc_feature    5639
                     /Description="active site"
                     /db_xref="CDD:238415"
                     /gene="int"
                     /label="int"
     misc_feature    5639
                     /Description="Int/Topo IB signature motif; other
site"
                     /db_xref="CDD:238415"
                     /gene="int"
                     /label="int"
     CDS             complement(62..775)
                     /db_xref="GI:16209182"
                     /product="cI"
                     /codon_start=1
                     /protein_id="AAL09915.1"
                     /transl_table=11
                     /label="CDS_1"
     misc_feature    complement(74..712)
                     /Description="SOS-response transcriptional
repressors
                     (RecA-mediated autopeptidases) [Transcription /
Signal
                     transduction mechanisms]; Region: LexA; COG1974"
                     /db_xref="CDD:224885"
                     /label="Misc_Feature_3"
     misc_feature    complement(95..352)
                     /Description="Peptidase S24 LexA-like proteins are
                     involved in the SOS response leading to the repair
of
                     single-stranded DNA within the bacterial cell. This
family
                     includes: the lambda repressor CI/C2 family and
related
                     bacterial prophage repressor proteins; LexA (EC...;
                     Region: S24_LexA-like; cd06529"
                     /db_xref="CDD:119397"
```

```
                     /label="Misc_Feature_1"
     misc_feature    complement(197..328)
                     /Description="Catalytic site [active]"
                     /db_xref="CDD:119397"
                     /label="Misc_Feature_4"
     misc_feature    complement(545..730)
                     /Description="Helix-turn-helix XRE-family like
proteins.
                     Prokaryotic DNA binding proteins belonging to the
                     xenobiotic response element family of
transcriptional
                     regulators; Region: HTH_XRE; cd00093"
                     /db_xref="CDD:238045"
                     /label="Misc_Feature_2"
     misc_feature    complement(614..676)
                     /Description="sequence-specific DNA binding site
                     [nucleotide binding]; other site"
                     /db_xref="CDD:238045"
                     /label="Misc_Feature_6"
     misc_feature    complement(617..718)
                     /Description="non-specific DNA binding site
[nucleotide
                     binding]; other site"
                     /db_xref="CDD:238045"
                     /label="Misc_Feature_5"
     misc_feature    complement(620..709)
                     /Description="salt bridge; other site"
                     /db_xref="CDD:238045"
                     /label="Misc_Feature_7"
     misc_feature    878..2698
                     /label="PhiK38int-attB_pInt-Ts"
     CDS             complement(2806..3756)
                     /db_xref="GI:16209184"
                     /product="repA101"
                     /codon_start=1
                     /protein_id="AAL09917.1"
                     /transl_table=11
                     /label="CDS_2"
     misc_feature    complement(3094..3753)
                     /Description="Initiator Replication protein; Region:
                     Rep_3; pfam01051"
                     /db_xref="CDD:250324"
                     /label="Misc_Feature_8"
     CDS             complement(4648..5508)
                     /db_xref="GI:16209185"
                     /product="bla"
                     /codon_start=1
                     /protein_id="AAL09918.1"
                     /transl_table=11
                     /label="CDS_3"
     misc_feature    complement(4651..5502)
                     /Description="beta-lactamase TEM; Provisional;
Region:
                     PRK15442"
```

```
                     /db_xref="CDD:185339"
                     /label="Misc_Feature_9"
ORIGIN
        1 catcgatgaa gattcttgct caattgttat cagctatgcg ccgaccagaa
caccttgccg
       61 atcagccaaa cgtctcttca ggccactgac tagcgataac tttccccaca
acggaacaac
      121 tctcattgca tgggatcatt gggtactgtg ggtttagtgg ttgtaaaaac
acctgaccgc
      181 tatccctgat cagtttcttg aaggtaaact catcaccccc aagtctggct
atgcagaaat
      241 cacctggctc aacagcctgc tcagggtcaa cgagaattaa cattccgtca
ggaaagcttg
      301 gcttggagcc tgttggtgcg gtcatggaat taccttcaac ctcaagccag
aatgcagaat
      361 cactggcttt tttggttgtg cttacccatc tctccgcatc acctttggta
aaggttctaa
      421 gcttaggtga gaacatccct gcctgaacat gagaaaaaac agggtactca
tactcacttc
      481 taagtgacgg ctgcatacta accgcttcat acatctcgta gatttctctg
gcgattgaag
      541 ggctaaattc ttcaacgcta actttgagaa tttttgtaag caatgcggcg
ttataagcat
      601 ttaatgcatt gatgccatta aataaagcac caacgcctga ctgccccatc
cccatcttgt
      661 ctgcgacaga ttcctgggat aagccaagtt catttttctt tttttcataa
attgctttaa
      721 ggcgacgtgc gtcctcaagc tgctcttgtg ttaatggttt ctttttttgtg
ctcatacgtt
      781 aaatctatca ccgcaaggga taaatatcta acaccgtgcg tgttgactat
tttacctctg
      841 gcggtgataa tggttgcatg tactaaggag gttgtatgaa ttcgagctcg
gtacccagtt
      901 cgtcgatggg ttcgggaatg caggatattc ccacctccgg ttaaggatgg
aagagagtat
      961 ctgttccacg aatcagcggt aaaggttgac ttaaatcgac cagtaacagg
tggccttttg
     1021 aagaggactg cagatgccgg gtatgaccac cgagaccggt ccggacccgg
cgggcctgat
     1081 tgacctgttc tgccgtaaga gcaaagcggt taaaagccgt gcgaacggtg
cgggtcagcg
     1141 tcgtaaacaa gagatcagca ttgcggcgca agaaaccctg ggtcgtaaag
tggcggcgct
     1201 gctgggtatg caagtgcgtc acgtttggaa agaagttggt agcgcgagcc
gttttcgtaa
     1261 gggcaaagcg cgtgacgatc aaagcaaggc gctgaaagcg ctggagagcg
gtgaagtggg
     1321 tgcgctgtgg tgctaccgtc tggaccgttg ggatcgtggt ggcgcgggtg
cgatcctgaa
     1381 gatcattgag ccggaagacg gcatgccgcg tcgtctgctg ttcggttggg
acgaggatac
     1441 cggccgtccg gttctggaca gcaccaacaa acgtgatcgt ggtgaactga
ttcgtcgtgc
```

1501 ggaggaagcg cgtgaggaag cggagaagct gagcgaacgt gtgcgtgaca ccaaagcgca
1561 ccagcgtgag aacggtgaat gggttaacgc gcgtgcgccg tatggtctgc gtgtggttct
1621 ggtgaccgtt agcgatgagg aaggtgacga gtatgatgag cgtaagctgg cggcggacga
1681 tgaagatgcg ggtggcccgg atggtctgac caaagcggaa gcggcgcgtc tggtgttcac
1741 cctgccggtt accgaccgtc tgagctatgc gggtaccgcg cacgcgatga acacccgtga
1801 gattccgagc ccgaccggtg gcccgtggat tgcggtgacc gttcgtgaca tgattcaaaa
1861 cccggcgtat gcgggttggc agaccaccgg tcgtcaggat ggcaagcaac gtcgtctgac
1921 cttttataac ggtgaaggca aacgtgtgag cgttatgcat ggcccgccgc tggtgaccga
1981 tgaggaacaa gaggcggcga aagcggcggt taaaggtgaa gacggtgtgg gcgttccgct
2041 ggatggcagc gaccacgata cccgtcgtaa gcacctgctg agcggtcgta tgcgttgccc
2101 gggttgcggt ggtagctgta gctacagcgg taacggctat cgttgctggc gtagcagcgt
2161 gaagggtggc tgcccggcgc cgacctatgt tgcgcgtaaa agcgtggagg aatatgttgc
2221 gtttcgttgg gcggcgaaac tggcggcgag cgagccggac gatccgtttg tgattgcggt
2281 tgcggaccgt tgggcggcgc tgacccaccc gcaagcgagc gaggatgaaa agtacgcgaa
2341 agcggcggtg cgtgaggcgg aaaagaacct gggtcgtctg ctgcgcgacc gtcagaacgg
2401 tgtttacgat ggtccggcgg agcagttctt tgcgccggcg tatcaagaag cgctgagcac
2461 cctgcaagcg gcgaaagatg ctgtgagcga aagcagcgcg agcgcggcgg tggatgttag
2521 ctggatcgtt gacagcagcg attatgagga actgtggctg cgtgcgaccc cgaccatgcg
2581 taacgcgatc attgacacct gcatcgatga gatttgggtg gcgaagggtc agcgtggtcg
2641 tccgtttgac ggcgatgaac gtgttaagat taaatgggcg gcgcgtacct aagctagccc
2701 atgggtatgg acagttttcc ctttgatatg taacggtgaa cagttgttct acttttgttt
2761 gttagtcttg atgcttcact gatagataca agagccataa gaacctcaga tccttccgta
2821 tttagccagt atgttctcta gtgtggttcg ttgtttttgc gtgagccatg agaacgaacc
2881 attgagatca tacttacttt gcatgtcact caaaaatttt gcctcaaaac tggtgagctg
2941 aatttttgca gttaaagcat cgtgtagtgt ttttcttagt ccgttacgta ggtaggaatc
3001 tgatgtaatg gttgttggta ttttgtcacc attcattttt atctggttgt tctcaagttc
3061 ggttacgaga tccatttgtc tatctagttc aacttggaaa atcaacgtat cagtcgggcg

3121 gcctcgctta tcaaccacca atttcatatt gctgtaagtg tttaaatctt tacttattgg
3181 tttcaaaacc cattggttaa gccttttaaa ctcatggtag ttattttcaa gcattaacat
3241 gaacttaaat tcatcaaggc taatctctat atttgccttg tgagttttct tttgtgttag
3301 ttcttttaat aaccactcat aaatcctcat agagtatttg ttttcaaaag acttaacatg
3361 ttccagatta tattttatga atttttttaa ctggaaaaga taaggcaata tctcttcact
3421 aaaaactaat tctaattttt cgcttgagaa cttggcatag tttgtccact ggaaaatctc
3481 aaagccttta accaaaggat tcctgatttc cacagttctc gtcatcagct ctctggttgc
3541 tttagctaat acaccataag cattttccct actgatgttc atcatctgag cgtattggtt
3601 ataagtgaac gataccgtcc gttctttcct tgtagggttt tcaatcgtgg ggttgagtag
3661 tgccacacag cataaaatta gcttggtttc atgctccgtt aagtcatagc gactaatcgc
3721 tagttcattt gctttgaaaa caactaattc agacatacat ctcaattggt ctaggtgatt
3781 ttaatcacta taccaattga gatgggctag tcaatgataa ttactagtcc ttttcctttg
3841 agttgtgggt atctgtaaat tctgctagac ctttgctgga aaacttgtaa attctgctag
3901 accctctgta aattccgcta gacctttgtg tgtttttttt gtttatattc aagtggttat
3961 aatttataga ataaagaaag aataaaaaaa gataaaaaga atagatccca gccctgtgta
4021 taactcacta ctttagtcag ttccgcagta ttacaaaagg atgtcgcaaa cgctgtttgc
4081 tcctctacaa aacagacctt aaaaccctaa aggcttaagt agcaccctcg caagctcggt
4141 tgcggccgca atcgggcaaa tcgctgaata ttccttttgt ctccgaccat caggcacctg
4201 agtcgctgtc tttttcgtga cattcagttc gctgcgctca cggctctggc agtgaatggg
4261 ggtaaatggc actacaggcg ccttttatgg attcatgcaa ggaaactacc cataatacaa
4321 gaaaagcccg tcacgggctt ctcagggcgt tttatggcgg gtctgctatg tggtgctatc
4381 tgactttttg ctgttcagca gttcctgccc tctgattttc cagtctgacc acttcggatt
4441 atcccgtgac aggtcattca gactggctaa tgcacccagt aaggcagcgg tatcatcaac
4501 ggggtctgac gctcagtgga acgaaaactc acgttaaggg attttggtca tgagattatc
4561 aaaaaggatc ttcacctaga tccttttaaa ttaaaaatga agttttaaat caatctaaag
4621 tatatatgag taaacttggt ctgacagtta ccaatgctta atcagtgagg cacctatctc
4681 agcgatctgt ctatttcgtt catccatagt tgcctgactc cccgtcgtgt agataactac

4741 gatacgggag ggcttaccat ctggccccag tgctgcaatg ataccgcgag acccacgctc
4801 accggctcca gatttatcag caataaacca gccagccgga agggccgagc gcagaagtgg
4861 tcctgcaact ttatccgcct ccatccagtc tattaattgt tgccgggaag ctagagtaag
4921 tagttcgcca gttaatagtt tgcgcaacgt tgttgccatt gctacaggca tcgtggtgtc
4981 acgctcgtcg tttggtatgg cttcattcag ctccggttcc caacgatcaa ggcgagttac
5041 atgatccccc atgttgtgca aaaaagcggt tagctccttc ggtcctccga tcgttgtcag
5101 aagtaagttg gccgcagtgt tatcactcat ggttatggca gcactgcata attctcttac
5161 tgtcatgcca tccgtaagat gcttttctgt gactggtgag tactcaacca agtcattctg
5221 agaatagtgt atgcggcgac cgagttgctc ttgcccggcg tcaatacggg ataataccgc
5281 gccacatagc agaactttaa aagtgctcat cattggaaaa cgttcttcgg ggcgaaaact
5341 ctcaaggatc ttaccgctgt tgagatccag ttcgatgtaa cccactcgtg cacccaactg
5401 atcttcagca tcttttactt tcaccagcgt ttctgggtga gcaaaaacag gaaggcaaaa
5461 tgccgcaaaa aagggaataa gggcgacacg gaaatgttga atactcatac tcttcctttt
5521 tcaatattat tgaagcattt atcagggtta ttgtctcatg agcggataca tatttgaatg
5581 tatttagaaa aataaacaaa taggggttcc gcgcacattt ccccgaaaag tgccacctg
//

```
LOCUS       pGSs045                 5357 bp    DNA     circular UNA 26-
OCT-2015
DEFINITION  CRIM helper plasmid pINT-ts, complete sequence.
ACCESSION   urn.local...3v-exvb5ay
VERSION     urn.local...3v-exvb5ay
KEYWORDS    .
SOURCE      CRIM helper plasmid pINT-ts (CRIM helper plasmid pINT-ts.)
  ORGANISM  CRIM helper plasmid pINT-ts
            other sequences;artificial sequences;vectors.
FEATURES             Location/Qualifiers
     misc_feature    5357
                     /Description="dimer interface [polypeptide binding];
other
                     site"
                     /db_xref="CDD:238415"
                     /gene="int"
                     /label="int"
     misc_feature    5357
                     /Description="active site"
                     /db_xref="CDD:238415"
                     /gene="int"
                     /label="int"
     misc_feature    5357
                     /Description="Int/Topo IB signature motif; other
site"
                     /db_xref="CDD:238415"
                     /gene="int"
                     /label="int"
     CDS             complement(62..775)
                     /db_xref="GI:16209182"
                     /product="cI"
                     /codon_start=1
                     /protein_id="AAL09915.1"
                     /transl_table=11
                     /label="CDS_1"
     misc_feature    complement(74..712)
                     /Description="SOS-response transcriptional
repressors
                     (RecA-mediated autopeptidases) [Transcription /
Signal
                     transduction mechanisms]; Region: LexA; COG1974"
                     /db_xref="CDD:224885"
                     /label="Misc_Feature_3"
     misc_feature    complement(95..352)
                     /Description="Peptidase S24 LexA-like proteins are
                     involved in the SOS response leading to the repair
of
                     single-stranded DNA within the bacterial cell. This
family
                     includes: the lambda repressor CI/C2 family and
related
                     bacterial prophage repressor proteins; LexA (EC...;
                     Region: S24_LexA-like; cd06529"
                     /db_xref="CDD:119397"
```

```
                     /label="Misc_Feature_1"
     misc_feature    complement(197..328)
                     /Description="Catalytic site [active]"
                     /db_xref="CDD:119397"
                     /label="Misc_Feature_4"
     misc_feature    complement(545..730)
                     /Description="Helix-turn-helix XRE-family like
proteins.
                     Prokaryotic DNA binding proteins belonging to the
                     xenobiotic response element family of
transcriptional
                     regulators; Region: HTH_XRE; cd00093"
                     /db_xref="CDD:238045"
                     /label="Misc_Feature_2"
     misc_feature    complement(614..676)
                     /Description="sequence-specific DNA binding site
                     [nucleotide binding]; other site"
                     /db_xref="CDD:238045"
                     /label="Misc_Feature_6"
     misc_feature    complement(617..718)
                     /Description="non-specific DNA binding site
[nucleotide
                     binding]; other site"
                     /db_xref="CDD:238045"
                     /label="Misc_Feature_5"
     misc_feature    complement(620..709)
                     /Description="salt bridge; other site"
                     /db_xref="CDD:238045"
                     /label="Misc_Feature_7"
     misc_feature    878..2416
                     /label="phiMR11int-attB_pInt-Ts"
     CDS             complement(2524..3474)
                     /db_xref="GI:16209184"
                     /product="repA101"
                     /codon_start=1
                     /protein_id="AAL09917.1"
                     /transl_table=11
                     /label="CDS_2"
     misc_feature    complement(2812..3471)
                     /Description="Initiator Replication protein; Region:
                     Rep_3; pfam01051"
                     /db_xref="CDD:250324"
                     /label="Misc_Feature_8"
     CDS             complement(4366..5226)
                     /db_xref="GI:16209185"
                     /product="bla"
                     /codon_start=1
                     /protein_id="AAL09918.1"
                     /transl_table=11
                     /label="CDS_3"
     misc_feature    complement(4369..5220)
                     /Description="beta-lactamase TEM; Provisional;
Region:
                     PRK15442"
```

```
                     /db_xref="CDD:185339"
                     /label="Misc_Feature_9"
ORIGIN
        1 catcgatgaa gattcttgct caattgttat cagctatgcg ccgaccagaa
caccttgccg
       61 atcagccaaa cgtctcttca ggccactgac tagcgataac tttccccaca
acggaacaac
      121 tctcattgca tgggatcatt gggtactgtg ggtttagtgg ttgtaaaaac
acctgaccgc
      181 tatccctgat cagtttcttg aaggtaaact catcaccccc aagtctggct
atgcagaaat
      241 cacctggctc aacagcctgc tcagggtcaa cgagaattaa cattccgtca
ggaaagcttg
      301 gcttggagcc tgttggtgcg gtcatggaat taccttcaac ctcaagccag
aatgcagaat
      361 cactggcttt tttggttgtg cttacccatc tctccgcatc acctttggta
aaggttctaa
      421 gcttaggtga gaacatccct gcctgaacat gagaaaaaac agggtactca
tactcacttc
      481 taagtgacgg ctgcatacta accgcttcat acatctcgta gatttctctg
gcgattgaag
      541 ggctaaattc ttcaacgcta actttgagaa tttttgtaag caatgcggcg
ttataagcat
      601 ttaatgcatt gatgccatta aataaagcac caacgcctga ctgccccatc
cccatcttgt
      661 ctgcgacaga ttcctgggat aagccaagtt cattttcttt tttttcataa
attgctttaa
      721 ggcgacgtgc gtcctcaagc tgctcttgtg ttaatggttt ctttttgtg
ctcatacgtt
      781 aaatctatca ccgcaaggga taaatatcta acaccgtgcg tgttgactat
tttacctctg
      841 gcggtgataa tggttgcatg tactaaggag gttgtatgaa ttcgagctcg
gtacccagtt
      901 cgtcgatggg ttcgggaatg caggatattc ccacctccgg ttaaggatgg
aagagagtat
      961 ctgttccacg aatcagcggt aaaggttgac ttaaatcgac cagtaacagg
tggccttttg
     1021 aagaggactg cagatgaagg ttgcgatcta cacccgtgtg agcaccctgg
agcagaagga
     1081 aaaaggtcac agcattgagg aacaagagcg taaactgcgt gcgtatagcg
acatcaacga
     1141 ttggaccatt cagggcgttt acgtggacgc gggttatagc ggcgcgaaga
ccgatcgtcc
     1201 ggagctgaac cgtctgaagg aaaacctgag caaaatcgac ctggttctgg
tgtacaagct
     1261 ggatcgtctg acccgtaacg ttaaagacct gctggatctg ctggagattt
tcgaacgtga
     1321 gaacgttagc tttcgtagcg cgaccgaagt gtatgacacc agcaccgcga
tgggtcgtct
     1381 gttcgttacc ctggtgggcg cgatggcgga atgggagcgt gaaaccatcc
gtgagcgtgc
     1441 gatgatgggc aagcaggcgg cgatccgtaa aggcatgatt ctgaccccgc
cgccgtttta
```

1501 ctatgaccgt gttgataaca agtacatccc gaacaagtac aaagatgtgg
ttgtgtgggc
1561 gtacgaggaa gtgaagaaag gtaacagcgc gaagggcatc gcgcgtaaac
tgaacgcgag
1621 cgacatcccg ccgccgaacg gtattcaatg ggaagatcgt accattaccc
gtgcgctgcg
1681 tagcccgctg agcaagggtc actacttctg gggcgacatc tttattgaga
acagccacga
1741 accgatcatt accgatgaga tgtataacga gatcaaggaa cgtctgaacg
aacgtgttaa
1801 cgcgaaaacc atcacccaca ccagcgtgtt ccgtggtaaa ctgatttgcc
cgaactgcaa
1861 cggccgtctg tgcctgaaca ccagctaccg taagctgaaa cgtggtgacg
ttatccacaa
1921 gaactactat tgcaacaact gcaaggtgaa caaaagcggc gcgttcagct
ttaccgagaa
1981 ggaagcgctg aaagtgttct acgactatct gagcaagctg gatctgagca
agtataaagc
2041 gaaggagaaa gaagacaaga aaatcgttac catcgatatc aacaaggtga
tggaacagcg
2101 taagcgttac cacaaactgt atgcgaacgg tatgatgcaa gaggaagagc
tgttcgagct
2161 gatcaaggag accgacgaaa aaattagcga gtacgaaaag caaaaagaac
gtgttccgaa
2221 gaaacgtctg gacgtgagca agatcaaaaa ctttaagaac attctgctgg
atagctggaa
2281 cgcgttcacc ctggaggaca aagaagattt catcaagatg gcgatcaaga
gcatcgagat
2341 tgaatacatc cacgtgaaac gtggcaagac caagcacagc atcaagatca
agaacatcga
2401 tttctattaa gctagcccat gggtatggac agttttccct ttgatatgta
acggtgaaca
2461 gttgttctac ttttgtttgt tagtcttgat gcttcactga tagatacaag
agccataaga
2521 acctcagatc cttccgtatt tagccagtat gttctctagt gtggttcgtt
gtttttgcgt
2581 gagccatgag aacgaaccat tgagatcata cttactttgc atgtcactca
aaaattttgc
2641 ctcaaaactg gtgagctgaa tttttgcagt taaagcatcg tgtagtgttt
ttcttagtcc
2701 gttacgtagg taggaatctg atgtaatggt tgttggtatt ttgtcaccat
tcatttttat
2761 ctggttgttc tcaagttcgg ttacgagatc catttgtcta tctagttcaa
cttggaaaat
2821 caacgtatca gtcgggcggc ctcgcttatc aaccaccaat ttcatattgc
tgtaagtgtt
2881 taaatcttta cttattggtt tcaaaaccca ttggttaagc cttttaaact
catggtagtt
2941 attttcaagc attaacatga acttaaattc atcaaggcta atctctatat
ttgccttgtg
3001 agttttcttt tgtgttagtt cttttaataa ccactcataa atcctcatag
agtatttgtt
3061 ttcaaaagac ttaacatgtt ccagattata ttttatgaat ttttttaact
ggaaaagata

3121 aggcaatatc tcttcactaa aaactaattc taatttttcg cttgagaact tggcatagtt
3181 tgtccactgg aaaatctcaa agcctttaac caaaggattc ctgatttcca cagttctcgt
3241 catcagctct ctggttgctt tagctaatac accataagca ttttccctac tgatgttcat
3301 catctgagcg tattggttat aagtgaacga taccgtccgt tctttccttg tagggttttc
3361 aatcgtgggg ttgagtagtg ccacacagca taaaattagc ttggtttcat gctccgttaa
3421 gtcatagcga ctaatcgcta gttcatttgc tttgaaaaca actaattcag acatacatct
3481 caattggtct aggtgatttt aatcactata ccaattgaga tgggctagtc aatgataatt
3541 actagtcctt ttcctttgag ttgtgggtat ctgtaaattc tgctagacct ttgctggaaa
3601 acttgtaaat tctgctagac cctctgtaaa ttccgctaga cctttgtgtg ttttttttgt
3661 ttatattcaa gtggttataa tttatagaat aaagaaagaa taaaaaaaga taaaaagaat
3721 agatcccagc cctgtgtata actcactact ttagtcagtt ccgcagtatt acaaaaggat
3781 gtcgcaaacg ctgtttgctc ctctacaaaa cagaccttaa aaccctaaag gcttaagtag
3841 caccctcgca agctcggttg cggccgcaat cgggcaaatc gctgaatatt ccttttgtct
3901 ccgaccatca ggcacctgag tcgctgtctt tttcgtgaca ttcagttcgc tgcgctcacg
3961 gctctggcag tgaatggggg taaatggcac tacaggcgcc ttttatggat tcatgcaagg
4021 aaactaccca taatacaaga aaagcccgtc acgggcttct cagggcgttt tatggcgggt
4081 ctgctatgtg gtgctatctg actttttgct gttcagcagt tcctgccctc tgattttcca
4141 gtctgaccac ttcggattat cccgtgacag gtcattcaga ctggctaatg cacccagtaa
4201 ggcagcggta tcatcaacgg ggtctgacgc tcagtggaac gaaaactcac gttaagggat
4261 tttggtcatg agattatcaa aaaggatctt cacctagatc cttttaaatt aaaaatgaag
4321 ttttaaatca atctaaagta tatatgagta aacttggtct gacagttacc aatgcttaat
4381 cagtgaggca cctatctcag cgatctgtct atttcgttca tccatagttg cctgactccc
4441 cgtcgtgtag ataactacga tacgggaggg cttaccatct ggccccagtg ctgcaatgat
4501 accgcgagac ccacgctcac cggctccaga tttatcagca ataaaccagc cagccggaag
4561 ggccgagcgc agaagtggtc ctgcaacttt atccgcctcc atccagtcta ttaattgttg
4621 ccgggaagct agagtaagta gttcgccagt taatagtttg cgcaacgttg ttgccattgc
4681 tacaggcatc gtggtgtcac gctcgtcgtt tggtatggct tcattcagct ccggttccca

4741 acgatcaagg cgagttacat gatcccccat gttgtgcaaa aaagcggtta gctccttcgg
4801 tcctccgatc gttgtcagaa gtaagttggc cgcagtgtta tcactcatgg ttatggcagc
4861 actgcataat tctcttactg tcatgccatc cgtaagatgc ttttctgtga ctggtgagta
4921 ctcaaccaag tcattctgag aatagtgtat gcggcgaccg agttgctctt gcccggcgtc
4981 aatacgggat aataccgcgc cacatagcag aactttaaaa gtgctcatca ttggaaaacg
5041 ttcttcgggg cgaaaactct caaggatctt accgctgttg agatccagtt cgatgtaacc
5101 cactcgtgca cccaactgat cttcagcatc ttttactttc accagcgttt ctgggtgagc
5161 aaaaacagga aggcaaaatg ccgcaaaaaa gggaataagg gcgacacgga aatgttgaat
5221 actcatactc ttcctttttc aatattattg aagcatttat cagggttatt gtctcatgag
5281 cggatacata tttgaatgta tttagaaaaa taaacaaata ggggttccgc gcacatttcc
5341 ccgaaaagtg ccacctg
//

```
LOCUS       pGSs046                 5390 bp    DNA     circular UNA 26-
OCT-2015
DEFINITION  CRIM helper plasmid pINT-ts, complete sequence.
ACCESSION   urn.local...3u-exvb5ay
VERSION     urn.local...3u-exvb5ay
KEYWORDS    .
SOURCE      CRIM helper plasmid pINT-ts (CRIM helper plasmid pINT-ts.)
  ORGANISM  CRIM helper plasmid pINT-ts
            other sequences;artificial sequences;vectors.
FEATURES             Location/Qualifiers
     misc_feature    5390
                     /Description="dimer interface [polypeptide binding];
other
                     site"
                     /db_xref="CDD:238415"
                     /gene="int"
                     /label="int"
     misc_feature    5390
                     /Description="active site"
                     /db_xref="CDD:238415"
                     /gene="int"
                     /label="int"
     misc_feature    5390
                     /Description="Int/Topo IB signature motif; other
site"
                     /db_xref="CDD:238415"
                     /gene="int"
                     /label="int"
     source          1..5390
                     /Description="lambda Int"
                     /mol_type="genomic DNA"
                     /db_xref="taxon:172366"
                     /organism="CRIM helper plasmid pINT-ts"
                     /label="Source_1"
     CDS             complement(62..775)
                     /db_xref="GI:16209182"
                     /product="cI"
                     /codon_start=1
                     /protein_id="AAL09915.1"
                     /transl_table=11
                     /label="CDS_1"
     misc_feature    complement(74..712)
                     /Description="SOS-response transcriptional
repressors
                     (RecA-mediated autopeptidases) [Transcription /
Signal
                     transduction mechanisms]; Region: LexA; COG1974"
                     /db_xref="CDD:224885"
                     /label="Misc_Feature_3"
     misc_feature    complement(95..352)
                     /Description="Peptidase S24 LexA-like proteins are
                     involved in the SOS response leading to the repair
of
```

```
                     single-stranded DNA within the bacterial cell. This
family
                     includes: the lambda repressor CI/C2 family and
related
                     bacterial prophage repressor proteins; LexA (EC...;
                     Region: S24_LexA-like; cd06529"
                     /db_xref="CDD:119397"
                     /label="Misc_Feature_1"
     misc_feature    complement(197..328)
                     /Description="Catalytic site [active]"
                     /db_xref="CDD:119397"
                     /label="Misc_Feature_4"
     misc_feature    complement(545..730)
                     /Description="Helix-turn-helix XRE-family like
proteins.
                     Prokaryotic DNA binding proteins belonging to the
                     xenobiotic response element family of
transcriptional
                     regulators; Region: HTH_XRE; cd00093"
                     /db_xref="CDD:238045"
                     /label="Misc_Feature_2"
     misc_feature    complement(614..676)
                     /Description="sequence-specific DNA binding site
                     [nucleotide binding]; other site"
                     /db_xref="CDD:238045"
                     /label="Misc_Feature_6"
     misc_feature    complement(617..718)
                     /Description="non-specific DNA binding site
[nucleotide
                     binding]; other site"
                     /db_xref="CDD:238045"
                     /label="Misc_Feature_5"
     misc_feature    complement(620..709)
                     /Description="salt bridge; other site"
                     /db_xref="CDD:238045"
                     /label="Misc_Feature_7"
     misc_feature    878..2449
                     /label="R4int-attB_pInt-Ts"
     CDS             complement(2557..3507)
                     /db_xref="GI:16209184"
                     /product="repA101"
                     /codon_start=1
                     /protein_id="AAL09917.1"
                     /transl_table=11
                     /label="CDS_2"
     misc_feature    complement(2845..3504)
                     /Description="Initiator Replication protein; Region:
                     Rep_3; pfam01051"
                     /db_xref="CDD:250324"
                     /label="Misc_Feature_8"
     CDS             complement(4399..5259)
                     /db_xref="GI:16209185"
                     /product="bla"
                     /codon_start=1
```

```
                     /protein_id="AAL09918.1"
                     /transl_table=11
                     /label="CDS_3"
     misc_feature    complement(4402..5253)
                     /Description="beta-lactamase TEM; Provisional;
Region:
                     PRK15442"
                     /db_xref="CDD:185339"
                     /label="Misc_Feature_9"
ORIGIN
        1 catcgatgaa gattcttgct caattgttat cagctatgcg ccgaccagaa
caccttgccg
       61 atcagccaaa cgtctcttca ggccactgac tagcgataac tttccccaca
acggaacaac
      121 tctcattgca tgggatcatt gggtactgtg ggtttagtgg ttgtaaaaac
acctgaccgc
      181 tatccctgat cagtttcttg aaggtaaact catcaccccc aagtctggct
atgcagaaat
      241 cacctggctc aacagcctgc tcagggtcaa cgagaattaa cattccgtca
ggaaagcttg
      301 gcttggagcc tgttggtgcg gtcatggaat taccttcaac ctcaagccag
aatgcagaat
      361 cactggcttt tttggttgtg cttacccatc tctccgcatc acctttggta
aaggttctaa
      421 gcttaggtga gaacatccct gcctgaacat gagaaaaaac agggtactca
tactcacttc
      481 taagtgacgg ctgcatacta accgcttcat acatctcgta gatttctctg
gcgattgaag
      541 ggctaaattc ttcaacgcta actttgagaa tttttgtaag caatgcggcg
ttataagcat
      601 ttaatgcatt gatgccatta aataaagcac caacgcctga ctgccccatc
cccatcttgt
      661 ctgcgacaga ttcctgggat aagccaagtt cattttcctt tttttcataa
attgctttaa
      721 ggcgacgtgc gtcctcaagc tgctcttgtg ttaatggttt ctttttgtg
ctcatacgtt
      781 aaatctatca ccgcaaggga taaatatcta acaccgtgcg tgttgactat
tttacctctg
      841 gcggtgataa tggttgcatg tactaaggag gttgtatgaa ttcgagctcg
gtacccagtt
      901 cgtcgatggg ttcgggaatg caggatattc ccacctccgg ttaaggatgg
aagagagtat
      961 ctgttccacg aatcagcggt aaaggttgac ttaaatcgac cagtaacagg
tggccttttg
     1021 aagaggactg cagatgaacc gtggtggccc gaccgtgcgt gcggacatct
acgttcgtat
     1081 tagcctggat cgtaccggcg aggaactggg cgtggagcgt caggaagaga
gctgccgtga
     1141 actgtgcaag agcctgggta tggaagtggg ccaagtgtgg gttgacaacg
atctgagcgc
     1201 gaccaagaaa aacgtggttc gtccggactt cgaagcgatg atcgcgagca
acccgcaggc
     1261 gattgtgtgc tggcacaccg atcgtctgat ccgtgtgacc cgtgacctgg
agcgtgttat
```

1321 tgatctgggc gtgaacgttc acgcggttat ggcgggtcac ctggacctga gcaccccggc
1381 gggccgtgcg gtggcgcgta ccgttaccgc gtgggcgacc tacgagggtg aacagaaggc
1441 ggaacgtcaa aaactggcga acatccaaaa cgcgcgtgcg ggcaagccgt acaccccggg
1501 tatccgtccg tttggttatg gcgacgatca catgaccatt gtgaccgcgg aggcggatgc
1561 gatccgtgat ggtgcgaaaa tgattctgga tggttggagc ctgagcgcgg ttgcgcgtta
1621 ttgggaggaa ctgaaactgc aaagcccgcg tagcatggcg gcgggtggca aaggttggag
1681 cctgcgtggc gtgaagaaag ttctgaccag cccgcgttac gtgggtcgta gcagctatct
1741 gggtgaagtg gttggcgacg cgcaatggcc gccaatcctg gacccggatg tttactatgg
1801 cgtggttgcg attctgaaca acccggatcg tttcagcggt ggcccgcgta ccggtcgtac
1861 cccgggtacc ctgctggcgg gtattgcgct gtgcggcgag tgcggcaaga ccgtgagcgg
1921 tcgtggctac cgtggtgtgc tggtttatgg ctgcaaagac acccacaccc gtaccccgcg
1981 tagcattgcg gatggtcgtg cgagcagcag caccctggcg cgtctgatgt tcccggactt
2041 tctgccgggt ctgctggcga gcggtcaggc ggaagatggt caaagcgcgg cgagcaagca
2101 cagcgaggcg cagaccctgc gtgaacgtct ggatggcctg gcgaccgcgt atgcggaagg
2161 tgcgatcagc ctgagccaaa tgaccgcggg tagcgaggcg ctgcgtaaga aactggaagt
2221 gatcgaagcg gacctggttg gtagcgcggg cattccgccg tttgatccgg tggcgggtgt
2281 tgcgggcctg attagcggtt ggccgaccac cccgctgccg acccgtcgtg cgtgggtgga
2341 cttttgcctg gtggttaccc tgaacaccca gaaaggccgt cacgcgagca gcatgaccgt
2401 ggacgatcac gttaccatcg agtggcgtga tgttgcggaa taagctagcc catgggtatg
2461 gacagttttc cctttgatat gtaacggtga acagttgttc tacttttgtt tgttagtctt
2521 gatgcttcac tgatagatac aagagccata agaacctcag atccttccgt atttagccag
2581 tatgttctct agtgtggttc gttgtttttg cgtgagccat gagaacgaac cattgagatc
2641 atacttactt tgcatgtcac tcaaaaattt tgcctcaaaa ctggtgagct gaatttttgc
2701 agttaaagca tcgtgtagtg tttttcttag tccgttacgt aggtaggaat ctgatgtaat
2761 ggttgttggt attttgtcac cattcatttt tatctggttg ttctcaagtt cggttacgag
2821 atccatttgt ctatctagtt caacttggaa aatcaacgta tcagtcgggc ggcctcgctt
2881 atcaaccacc aatttcatat tgctgtaagt gtttaaatct ttacttattg gtttcaaaac

2941 ccattggtta agccttttaa actcatggta gttattttca agcattaaca tgaacttaaa
3001 ttcatcaagg ctaatctcta tatttgcctt gtgagttttc ttttgtgtta gttcttttaa
3061 taaccactca taaatcctca tagagtattt gttttcaaaa gacttaacat gttccagatt
3121 atattttatg aatttttta actggaaaag ataaggcaat atctcttcac taaaaactaa
3181 ttctaatttt tcgcttgaga acttggcata gtttgtccac tggaaaatct caaagccttt
3241 aaccaaagga ttcctgattt ccacagttct cgtcatcagc tctctggttg ctttagctaa
3301 tacaccataa gcattttccc tactgatgtt catcatctga gcgtattggt tataagtgaa
3361 cgataccgtc cgttctttcc ttgtagggtt ttcaatcgtg gggttgagta gtgccacaca
3421 gcataaaatt agcttggttt catgctccgt taagtcatag cgactaatcg ctagttcatt
3481 tgctttgaaa acaactaatt cagacataca tctcaattgg tctaggtgat tttaatcact
3541 ataccaattg agatgggcta gtcaatgata attactagtc cttttccttt gagttgtggg
3601 tatctgtaaa ttctgctaga cctttgctgg aaaacttgta aattctgcta gaccctctgt
3661 aaattccgct agacctttgt gtgttttttt tgtttatatt caagtggtta taatttatag
3721 aataaagaaa gaataaaaaa agataaaaag aatagatccc agccctgtgt ataactcact
3781 actttagtca gttccgcagt attacaaaag gatgtcgcaa acgctgtttg ctcctctaca
3841 aaacagacct taaaacccta aaggcttaag tagcaccctc gcaagctcgg ttgcggccgc
3901 aatcgggcaa atcgctgaat attccttttg tctccgacca tcaggcacct gagtcgctgt
3961 ctttttcgtg acattcagtt cgctgcgctc acggctctgg cagtgaatgg gggtaaatgg
4021 cactacaggc gccttttatg gattcatgca aggaaactac ccataataca agaaaagccc
4081 gtcacgggct tctcagggcg ttttatggcg ggtctgctat gtggtgctat ctgacttttt
4141 gctgttcagc agttcctgcc ctctgatttt ccagtctgac cacttcggat tatcccgtga
4201 caggtcattc agactggcta atgcacccag taaggcagcg gtatcatcaa cggggtctga
4261 cgctcagtgg aacgaaaact cacgttaagg gattttggtc atgagattat caaaaaggat
4321 cttcacctag atccttttaa attaaaaatg aagttttaaa tcaatctaaa gtatatatga
4381 gtaaacttgg tctgacagtt accaatgctt aatcagtgag gcacctatct cagcgatctg
4441 tctatttcgt tcatccatag ttgcctgact ccccgtcgtg tagataacta cgatacggga
4501 gggcttacca tctggcccca gtgctgcaat gataccgcga gacccacgct caccggctcc

4561 agatttatca gcaataaacc agccagccgg aagggccgag cgcagaagtg gtcctgcaac
4621 tttatccgcc tccatccagt ctattaattg ttgccgggaa gctagagtaa gtagttcgcc
4681 agttaatagt ttgcgcaacg ttgttgccat tgctacaggc atcgtggtgt cacgctcgtc
4741 gtttggtatg gcttcattca gctccggttc ccaacgatca aggcgagtta catgatcccc
4801 catgttgtgc aaaaaagcgg ttagctcctt cggtcctccg atcgttgtca gaagtaagtt
4861 ggccgcagtg ttatcactca tggttatggc agcactgcat aattctctta ctgtcatgcc
4921 atccgtaaga tgcttttctg tgactggtga gtactcaacc aagtcattct gagaatagtg
4981 tatgcggcga ccgagttgct cttgcccggc gtcaatacgg gataataccg cgccacatag
5041 cagaacttta aaagtgctca tcattggaaa acgttcttcg gggcgaaaac tctcaaggat
5101 cttaccgctg ttgagatcca gttcgatgta acccactcgt gcacccaact gatcttcagc
5161 atcttttact ttcaccagcg tttctgggtg agcaaaaaca ggaaggcaaa atgccgcaaa
5221 aaagggaata agggcgacac ggaaatgttg aatactcata ctcttccttt ttcaatatta
5281 ttgaagcatt tatcagggtt attgtctcat gagcggatac atatttgaat gtatttagaa
5341 aaataaacaa ataggggttc cgcgcacatt tccccgaaaa gtgccacctg
//

```
LOCUS       pGSs047                 5618 bp    DNA     circular UNA 26-
OCT-2015
DEFINITION  CRIM helper plasmid pINT-ts, complete sequence.
ACCESSION   urn.local...3t-exvb5ay
VERSION     urn.local...3t-exvb5ay
KEYWORDS    .
SOURCE      CRIM helper plasmid pINT-ts (CRIM helper plasmid pINT-ts.)
  ORGANISM  CRIM helper plasmid pINT-ts
            other sequences;artificial sequences;vectors.
FEATURES             Location/Qualifiers
     misc_feature    5618
                     /Description="dimer interface [polypeptide binding];
other
                     site"
                     /db_xref="CDD:238415"
                     /gene="int"
                     /label="int"
     misc_feature    5618
                     /Description="active site"
                     /db_xref="CDD:238415"
                     /gene="int"
                     /label="int"
     misc_feature    5618
                     /Description="Int/Topo IB signature motif; other
site"
                     /db_xref="CDD:238415"
                     /gene="int"
                     /label="int"
     CDS             complement(62..775)
                     /db_xref="GI:16209182"
                     /product="cI"
                     /codon_start=1
                     /protein_id="AAL09915.1"
                     /transl_table=11
                     /label="CDS_1"
     misc_feature    complement(74..712)
                     /Description="SOS-response transcriptional
repressors
                     (RecA-mediated autopeptidases) [Transcription /
Signal
                     transduction mechanisms]; Region: LexA; COG1974"
                     /db_xref="CDD:224885"
                     /label="Misc_Feature_3"
     misc_feature    complement(95..352)
                     /Description="Peptidase S24 LexA-like proteins are
                     involved in the SOS response leading to the repair
of
                     single-stranded DNA within the bacterial cell. This
family
                     includes: the lambda repressor CI/C2 family and
related
                     bacterial prophage repressor proteins; LexA (EC...;
                     Region: S24_LexA-like; cd06529"
                     /db_xref="CDD:119397"
```

```
                     /label="Misc_Feature_1"
     misc_feature    complement(197..328)
                     /Description="Catalytic site [active]"
                     /db_xref="CDD:119397"
                     /label="Misc_Feature_4"
     misc_feature    complement(545..730)
                     /Description="Helix-turn-helix XRE-family like
proteins.
                     Prokaryotic DNA binding proteins belonging to the
                     xenobiotic response element family of
transcriptional
                     regulators; Region: HTH_XRE; cd00093"
                     /db_xref="CDD:238045"
                     /label="Misc_Feature_2"
     misc_feature    complement(614..676)
                     /Description="sequence-specific DNA binding site
                     [nucleotide binding]; other site"
                     /db_xref="CDD:238045"
                     /label="Misc_Feature_6"
     misc_feature    complement(617..718)
                     /Description="non-specific DNA binding site
[nucleotide
                     binding]; other site"
                     /db_xref="CDD:238045"
                     /label="Misc_Feature_5"
     misc_feature    complement(620..709)
                     /Description="salt bridge; other site"
                     /db_xref="CDD:238045"
                     /label="Misc_Feature_7"
     misc_feature    878..2677
                     /label="SPBeta_int-attB_pInt-Ts"
     CDS             complement(2785..3735)
                     /db_xref="GI:16209184"
                     /product="repA101"
                     /codon_start=1
                     /protein_id="AAL09917.1"
                     /transl_table=11
                     /label="CDS_2"
     misc_feature    complement(3073..3732)
                     /Description="Initiator Replication protein; Region:
                     Rep_3; pfam01051"
                     /db_xref="CDD:250324"
                     /label="Misc_Feature_8"
     CDS             complement(4627..5487)
                     /db_xref="GI:16209185"
                     /product="bla"
                     /codon_start=1
                     /protein_id="AAL09918.1"
                     /transl_table=11
                     /label="CDS_3"
     misc_feature    complement(4630..5481)
                     /Description="beta-lactamase TEM; Provisional;
Region:
                     PRK15442"
```

```
                     /db_xref="CDD:185339"
                     /label="Misc_Feature_9"
ORIGIN
        1 catcgatgaa gattcttgct caattgttat cagctatgcg ccgaccagaa
caccttgccg
       61 atcagccaaa cgtctcttca ggccactgac tagcgataac tttccccaca
acggaacaac
      121 tctcattgca tgggatcatt gggtactgtg ggtttagtgg ttgtaaaaac
acctgaccgc
      181 tatccctgat cagtttcttg aaggtaaact catcaccccc aagtctggct
atgcagaaat
      241 cacctggctc aacagcctgc tcagggtcaa cgagaattaa cattccgtca
ggaaagcttg
      301 gcttggagcc tgttggtgcg gtcatggaat taccttcaac ctcaagccag
aatgcagaat
      361 cactggcttt tttggttgtg cttacccatc tctccgcatc acctttggta
aaggttctaa
      421 gcttaggtga gaacatccct gcctgaacat gagaaaaaac agggtactca
tactcacttc
      481 taagtgacgg ctgcatacta accgcttcat acatctcgta gatttctctg
gcgattgaag
      541 ggctaaattc ttcaacgcta actttgagaa tttttgtaag caatgcggcg
ttataagcat
      601 ttaatgcatt gatgccatta aataaagcac caacgcctga ctgccccatc
cccatcttgt
      661 ctgcgacaga ttcctgggat aagccaagtt catttttctt tttttcataa
attgctttaa
      721 ggcgacgtgc gtcctcaagc tgctcttgtg ttaatggttt ctttttttgtg
ctcatacgtt
      781 aaatctatca ccgcaaggga taaatatcta acaccgtgcg tgttgactat
tttacctctg
      841 gcggtgataa tggttgcatg tactaaggag gttgtatgaa ttcgagctcg
gtacccagtt
      901 cgtcgatggg ttcgggaatg caggatattc ccacctccgg ttaaggatgg
aagagagtat
      961 ctgttccacg aatcagcggt aaaggttgac ttaaatcgac cagtaacagg
tggccttttg
     1021 aagaggactg cagatggagc tgaagaacat cgttaacagc tacaacatta
ccaacatcct
     1081 gggctatctg cgtcgtagcc gtcaggacat ggagcgtgaa aaacgtaccg
gtgaagatac
     1141 cctgaccgag caaaaggaac tgatgaacaa aatcctgacc gcgattgaga
tcccgtacga
     1201 actgaagatg gagattggta gcggtgaaag cattgatggt cgtccggtgt
ttaaggaatg
     1261 cctgaaagat ctggaggaag gtaaatacca ggcgattgcg gttaaagaga
tcacccgtct
     1321 gagccgtggc agctatagcg acgcgggtca gatcgtgaac ctgctgcaaa
gcaagcgtct
     1381 gatcattatc accccgtaca aagtgtatga cccgcgtaac ccggttgata
tgcgtcagat
     1441 tcgttttgaa ctgttcatgg cgcgtgagga gttcgaaatg acccgtgagc
gtatgaccgg
```

1501 cgcgaagtac acctatgcgg cgcagggtaa atggatcagc ggcctggcgc cgtacggtta
1561 tcaactgaac aagaaaacca gcaagctgga cccggttgag gatgaagcga aagtggttca
1621 gctgattttt aacatcttcc tgaacggtct gaacggcaag gactacagct ataccgcgat
1681 tgcgagccac ctgaccaacc tgcaaatccc gaccccgagc ggtaagaaac gttggaacca
1741 gtacaccatt aaagcgatcc tgcaaaacga ggtgtacatc ggcaccgtga agtataaagt
1801 tcgtgaaaag accaaagatg gcaagcgtac cattcgtccg gagaaagaac agatcgtggt
1861 tcaagacgcg cacgcgccga ttatcgataa agagcagttt cagcaaagcc aagtgaagat
1921 cgcgaacaaa gttccgctgc tgccgaacaa ggacgagttc gaactgagcg aactggcggg
1981 cgtgtgcacc tgtagcaaat gcggcgagcc gctgagcaag tacgaaagca aacgtattcg
2041 taagaacaaa gatggcaccg agagcgtgta tcacgttaag agcctgacct gcaagaaaaa
2101 caaatgcacc tacgtgcgtt ataacgacgt tgaaaacgcg atcctggatt acctgagcag
2161 cctgaacgac ctgaacgata gcaccctgac caagcacatt aacagcatgc tgagcaaata
2221 tgaggacgat aacagcaaca tgaagaccaa gaaacagatg agcgagcacc tgagccaaaa
2281 ggagaaggaa ctgaagaaca aggaaaactt catcttcgac aagtacgaga gcggcatcta
2341 tagcgatgaa ctgtttctga agcgtaaagc ggcgctggac gaggagttca aggagctgca
2401 aaacgcgaaa aacgaactga acggtctgca agacacccaa agcgagattg atagcaacac
2461 cgttcgtaac aacatcaaca agattatcga ccaataccac attgaaagca gcagcgagaa
2521 gaaaaacgaa ctgctgcgta tggtgctgaa agatgtgatc gttaacatga cccagaagcg
2581 taaaggtccg attccggcgc aatttgaaat taccccgatc ctgcgtttca actttatctt
2641 cgatctgacc gcgaccaaca gcttccacta agctagccca tgggtatgga cagttttccc
2701 tttgatatgt aacggtgaac agttgttcta cttttgtttg ttagtcttga tgcttcactg
2761 atagatacaa gagccataag aacctcagat ccttccgtat ttagccagta tgttctctag
2821 tgtggttcgt tgtttttgcg tgagccatga gaacgaacca ttgagatcat acttactttg
2881 catgtcactc aaaaattttg cctcaaaact ggtgagctga atttttgcag ttaaagcatc
2941 gtgtagtgtt tttcttagtc cgttacgtag gtaggaatct gatgtaatgg ttgttggtat
3001 tttgtcacca ttcattttta tctggttgtt ctcaagttcg gttacgagat ccatttgtct
3061 atctagttca acttggaaaa tcaacgtatc agtcgggcgg cctcgcttat caaccaccaa

3121 tttcatattg ctgtaagtgt ttaaatcttt acttattggt ttcaaaaccc
attggttaag
3181 ccttttaaac tcatggtagt tattttcaag cattaacatg aacttaaatt
catcaaggct
3241 aatctctata tttgccttgt gagttttctt ttgtgttagt tcttttaata
accactcata
3301 aatcctcata gagtatttgt tttcaaaaga cttaacatgt tccagattat
attttatgaa
3361 ttttttaac tggaaaagat aaggcaatat ctcttcacta aaaactaatt
ctaatttttc
3421 gcttgagaac ttggcatagt ttgtccactg gaaaatctca aagcctttaa
ccaaaggatt
3481 cctgatttcc acagttctcg tcatcagctc tctggttgct ttagctaata
caccataagc
3541 attttcccta ctgatgttca tcatctgagc gtattggtta taagtgaacg
ataccgtccg
3601 ttctttcctt gtagggtttt caatcgtggg gttgagtagt gccacacagc
ataaaattag
3661 cttggtttca tgctccgtta agtcatagcg actaatcgct agttcatttg
ctttgaaaac
3721 aactaattca gacatacatc tcaattggtc taggtgattt taatcactat
accaattgag
3781 atgggctagt caatgataat tactagtcct tttcctttga gttgtgggta
tctgtaaatt
3841 ctgctagacc tttgctggaa aacttgtaaa ttctgctaga ccctctgtaa
attccgctag
3901 acctttgtgt gttttttttg tttatattca agtggttata atttatagaa
taaagaaaga
3961 ataaaaaaag ataaaaagaa tagatcccag ccctgtgtat aactcactac
tttagtcagt
4021 tccgcagtat tacaaaagga tgtcgcaaac gctgtttgct cctctacaaa
acagacctta
4081 aaaccctaaa ggcttaagta gcaccctcgc aagctcggtt gcggccgcaa
tcgggcaaat
4141 cgctgaatat tccttttgtc tccgaccatc aggcacctga gtcgctgtct
ttttcgtgac
4201 attcagttcg ctgcgctcac ggctctggca gtgaatgggg gtaaatggca
ctacaggcgc
4261 cttttatgga ttcatgcaag gaaactaccc ataatacaag aaaagcccgt
cacgggcttc
4321 tcagggcgtt ttatggcggg tctgctatgt ggtgctatct gactttttgc
tgttcagcag
4381 ttcctgccct ctgattttcc agtctgacca cttcggatta tcccgtgaca
ggtcattcag
4441 actggctaat gcacccagta aggcagcggt atcatcaacg gggtctgacg
ctcagtggaa
4501 cgaaaactca cgttaaggga ttttggtcat gagattatca aaaaggatct
tcacctagat
4561 ccttttaaat taaaaatgaa gttttaaatc aatctaaagt atatatgagt
aaacttggtc
4621 tgacagttac caatgcttaa tcagtgaggc acctatctca gcgatctgtc
tatttcgttc
4681 atccatagtt gcctgactcc ccgtcgtgta gataactacg atacgggagg
gcttaccatc

4741 tggccccagt gctgcaatga taccgcgaga cccacgctca ccggctccag
atttatcagc
4801 aataaaccag ccagccggaa gggccgagcg cagaagtggt cctgcaactt
tatccgcctc
4861 catccagtct attaattgtt gccgggaagc tagagtaagt agttcgccag
ttaatagttt
4921 gcgcaacgtt gttgccattg ctacaggcat cgtggtgtca cgctcgtcgt
ttggtatggc
4981 ttcattcagc tccggttccc aacgatcaag gcgagttaca tgatccccca
tgttgtgcaa
5041 aaaagcggtt agctccttcg gtcctccgat cgttgtcaga agtaagttgg
ccgcagtgtt
5101 atcactcatg gttatggcag cactgcataa ttctcttact gtcatgccat
ccgtaagatg
5161 cttttctgtg actggtgagt actcaaccaa gtcattctga gaatagtgta
tgcggcgacc
5221 gagttgctct tgcccggcgt caatacggga taataccgcg ccacatagca
gaactttaaa
5281 agtgctcatc attggaaaac gttcttcggg gcgaaaactc tcaaggatct
taccgctgtt
5341 gagatccagt tcgatgtaac ccactcgtgc acccaactga tcttcagcat
cttttacttt
5401 caccagcgtt tctgggtgag caaaaacagg aaggcaaaat gccgcaaaaa
agggaataag
5461 ggcgacacgg aaatgttgaa tactcatact cttccttttt caatattatt
gaagcattta
5521 tcagggttat tgtctcatga gcggatacat atttgaatgt atttagaaaa
ataaacaaat
5581 aggggttccg cgcacatttc cccgaaaagt gccacctg
//

```
LOCUS       pGSs048                 5438 bp    DNA     circular UNA 26-
OCT-2015
DEFINITION  CRIM helper plasmid pINT-ts, complete sequence.
ACCESSION   urn.local...3s-exvb5ay
VERSION     urn.local...3s-exvb5ay
KEYWORDS    .
SOURCE      CRIM helper plasmid pINT-ts (CRIM helper plasmid pINT-ts.)
  ORGANISM  CRIM helper plasmid pINT-ts
            other sequences;artificial sequences;vectors.
FEATURES             Location/Qualifiers
     misc_feature    5438
                     /Description="dimer interface [polypeptide binding];
other
                     site"
                     /db_xref="CDD:238415"
                     /gene="int"
                     /label="int"
     misc_feature    5438
                     /Description="active site"
                     /db_xref="CDD:238415"
                     /gene="int"
                     /label="int"
     misc_feature    5438
                     /Description="Int/Topo IB signature motif; other
site"
                     /db_xref="CDD:238415"
                     /gene="int"
                     /label="int"
     CDS             complement(62..775)
                     /db_xref="GI:16209182"
                     /product="cI"
                     /codon_start=1
                     /protein_id="AAL09915.1"
                     /transl_table=11
                     /label="CDS_1"
     misc_feature    complement(74..712)
                     /Description="SOS-response transcriptional
repressors
                     (RecA-mediated autopeptidases) [Transcription /
Signal
                     transduction mechanisms]; Region: LexA; COG1974"
                     /db_xref="CDD:224885"
                     /label="Misc_Feature_3"
     misc_feature    complement(95..352)
                     /Description="Peptidase S24 LexA-like proteins are
                     involved in the SOS response leading to the repair
of
                     single-stranded DNA within the bacterial cell. This
family
                     includes: the lambda repressor CI/C2 family and
related
                     bacterial prophage repressor proteins; LexA (EC...;
                     Region: S24_LexA-like; cd06529"
                     /db_xref="CDD:119397"
```

```
                     /label="Misc_Feature_1"
     misc_feature    complement(197..328)
                     /Description="Catalytic site [active]"
                     /db_xref="CDD:119397"
                     /label="Misc_Feature_4"
     misc_feature    complement(545..730)
                     /Description="Helix-turn-helix XRE-family like
proteins.
                     Prokaryotic DNA binding proteins belonging to the
                     xenobiotic response element family of
transcriptional
                     regulators; Region: HTH_XRE; cd00093"
                     /db_xref="CDD:238045"
                     /label="Misc_Feature_2"
     misc_feature    complement(614..676)
                     /Description="sequence-specific DNA binding site
                     [nucleotide binding]; other site"
                     /db_xref="CDD:238045"
                     /label="Misc_Feature_6"
     misc_feature    complement(617..718)
                     /Description="non-specific DNA binding site
[nucleotide
                     binding]; other site"
                     /db_xref="CDD:238045"
                     /label="Misc_Feature_5"
     misc_feature    complement(620..709)
                     /Description="salt bridge; other site"
                     /db_xref="CDD:238045"
                     /label="Misc_Feature_7"
     misc_feature    878..2497
                     /label="TP901-1int-attB_pInt-Ts"
     CDS             complement(2605..3555)
                     /db_xref="GI:16209184"
                     /product="repA101"
                     /codon_start=1
                     /protein_id="AAL09917.1"
                     /transl_table=11
                     /label="CDS_2"
     misc_feature    complement(2893..3552)
                     /Description="Initiator Replication protein; Region:
                     Rep_3; pfam01051"
                     /db_xref="CDD:250324"
                     /label="Misc_Feature_8"
     CDS             complement(4447..5307)
                     /db_xref="GI:16209185"
                     /product="bla"
                     /codon_start=1
                     /protein_id="AAL09918.1"
                     /transl_table=11
                     /label="CDS_3"
     misc_feature    complement(4450..5301)
                     /Description="beta-lactamase TEM; Provisional;
Region:
                     PRK15442"
```

/db_xref="CDD:185339"
/label="Misc_Feature_9"

ORIGIN

```
        1 catcgatgaa gattcttgct caattgttat cagctatgcg ccgaccagaa
caccttgccg
       61 atcagccaaa cgtctcttca ggccactgac tagcgataac tttccccaca
acggaacaac
      121 tctcattgca tgggatcatt gggtactgtg ggtttagtgg ttgtaaaaac
acctgaccgc
      181 tatccctgat cagtttcttg aaggtaaact catcaccccc aagtctggct
atgcagaaat
      241 cacctggctc aacagcctgc tcagggtcaa cgagaattaa cattccgtca
ggaaagcttg
      301 gcttggagcc tgttggtgcg gtcatggaat taccttcaac ctcaagccag
aatgcagaat
      361 cactggcttt tttggttgtg cttacccatc tctccgcatc acctttggta
aaggttctaa
      421 gcttaggtga gaacatccct gcctgaacat gagaaaaaac agggtactca
tactcacttc
      481 taagtgacgg ctgcatacta accgcttcat acatctcgta gatttctctg
gcgattgaag
      541 ggctaaattc ttcaacgcta actttgagaa tttttgtaag caatgcggcg
ttataagcat
      601 ttaatgcatt gatgccatta aataaagcac caacgcctga ctgccccatc
cccatcttgt
      661 ctgcgacaga ttcctgggat aagccaagtt catttttctt tttttcataa
attgctttaa
      721 ggcgacgtgc gtcctcaagc tgctcttgtg ttaatggttt ctttttttgtg
ctcatacgtt
      781 aaatctatca ccgcaaggga taaatatcta acaccgtgcg tgttgactat
tttacctctg
      841 gcggtgataa tggttgcatg tactaaggag gttgtatgaa ttcgagctcg
gtacccagtt
      901 cgtcgatggg ttcgggaatg caggatattc ccacctccgg ttaaggatgg
aagagagtat
      961 ctgttccacg aatcagcggt aaaggttgac ttaaatcgac cagtaacagg
tggccttttg
     1021 aagaggactg cagatgacca agaaagtggc gatctacacc cgtgttagca
ccaccaacca
     1081 agcggaggaa ggtttcagca tcgacgaaca gattgatcgt ctgaccaaat
acgcggaggc
     1141 gatgggctgg caagtgagcg acacctatac cgatgcgggt ttcagcggtg
cgaagctgga
     1201 acgtccggcg atgcagcgtc tgatcaacga cattgagaac aaagcgtttg
ataccgtgct
     1261 ggtttacaag ctggaccgtc tgagccgtag cgttcgtgac accctgtatc
tggtgaaaga
     1321 tgttttcacc aagaacaaga tcgacttcat cagcctgaac gaaagcatcg
ataccagcag
     1381 cgcgatgggt agcctgttcc tgaccatcct gagcgcgatt aacgagtttg
aacgtgagaa
     1441 cattaaagag cgtatgacca tgggcaagct gggccgtgcg aaaagcggca
agagcatgat
```

1501 gtggaccaaa accgcgttcg gttactatca caaccgtaag accggcatcc tggaaattgt
1561 gccgctgcaa gcgaccatcg ttgagcagat ttttaccgat tacctgagcg gtatcagcct
1621 gaccaaactg cgtgacaagc tgaacgaaag cggtcacatc ggcaaagata ttccgtggag
1681 ctatcgtacc ctgcgtcaga ccctggacaa cccggtgtac tgcggttata tcaaattcaa
1741 ggatagcctg tttgaaggca tgcacaagcc gatcattccg tacgaaacct atctgaaagt
1801 tcaaaaggag ctggaggaac gtcagcaaca gacctacgag cgtaacaaca acccgcgtcc
1861 gttccaggcg aaatacatgc tgagcggcat ggcgcgttgc ggttattgcg gtgcgccgct
1921 gaaaatcgtg ctgggtcaca agcgtaagga cggcagccgt accatgaagt atcactgcgc
1981 gaaccgtttc ccgcgtaaaa ccaagggtat taccgtgtac aacgacaaca agaaatgcga
2041 tagcggcacc tatgacctga gcaacctgga aaacaccgtt atcgacaacc tgattggttt
2101 tcaagagaac aacgatagcc tgctgaagat cattaacggc aacaaccagc cgatcctgga
2161 caccagcagc ttcaagaaac aaatcagcca gattgataag aaaatccaga agaacagcga
2221 tctgtacctg aacgacttta ttaccatgga tgagctgaag gaccgtaccg atagcctgca
2281 agcggaaaag aaactgctga aagcgaagat cagcgagaac aaattcaacg acagcaccga
2341 tgtgtttgaa ctggttaaga cccaactggg tagcatcccg attaacgagc tgagctatga
2401 caacaagaaa aagattgtga acaacctggt tagcaaagtg gatgttaccg cggacaacgt
2461 tgatatcatt ttcaagtttc agctggcgta agctagccca tgggtatgga cagttttccc
2521 tttgatatgt aacggtgaac agttgttcta cttttgtttg ttagtcttga tgcttcactg
2581 atagatacaa gagccataag aacctcagat ccttccgtat ttagccagta tgttctctag
2641 tgtggttcgt tgtttttgcg tgagccatga gaacgaacca ttgagatcat acttactttg
2701 catgtcactc aaaaattttg cctcaaaact ggtgagctga atttttgcag ttaaagcatc
2761 gtgtagtgtt tttcttagtc cgttacgtag gtaggaatct gatgtaatgg ttgttggtat
2821 tttgtcacca ttcattttta tctggttgtt ctcaagttcg gttacgagat ccatttgtct
2881 atctagttca acttggaaaa tcaacgtatc agtcgggcgg cctcgcttat caaccaccaa
2941 tttcatattg ctgtaagtgt ttaaatcttt acttattggt ttcaaaaccc attggttaag
3001 ccttttaaac tcatggtagt tattttcaag cattaacatg aacttaaatt catcaaggct
3061 aatctctata tttgccttgt gagttttctt ttgtgttagt tcttttaata accactcata

3121 aatcctcata gagtatttgt tttcaaaaga cttaacatgt tccagattat attttatgaa
3181 tttttttaac tggaaaagat aaggcaatat ctcttcacta aaaactaatt ctaatttttc
3241 gcttgagaac ttggcatagt ttgtccactg gaaaatctca aagcctttaa ccaaaggatt
3301 cctgatttcc acagttctcg tcatcagctc tctggttgct ttagctaata caccataagc
3361 attttcccta ctgatgttca tcatctgagc gtattggtta taagtgaacg ataccgtccg
3421 ttctttcctt gtagggtttt caatcgtggg gttgagtagt gccacacagc ataaaattag
3481 cttggtttca tgctccgtta agtcatagcg actaatcgct agttcatttg ctttgaaaac
3541 aactaattca gacatacatc tcaattggtc taggtgattt taatcactat accaattgag
3601 atgggctagt caatgataat tactagtcct tttcctttga gttgtgggta tctgtaaatt
3661 ctgctagacc tttgctggaa aacttgtaaa ttctgctaga ccctctgtaa attccgctag
3721 acctttgtgt gttttttttg tttatattca agtggttata atttatagaa taaagaaaga
3781 ataaaaaaag ataaaaagaa tagatcccag ccctgtgtat aactcactac tttagtcagt
3841 tccgcagtat tacaaaagga tgtcgcaaac gctgtttgct cctctacaaa acagacctta
3901 aaaccctaaa ggcttaagta gcaccctcgc aagctcggtt gcggccgcaa tcgggcaaat
3961 cgctgaatat tccttttgtc tccgaccatc aggcacctga gtcgctgtct ttttcgtgac
4021 attcagttcg ctgcgctcac ggctctggca gtgaatgggg gtaaatggca ctacaggcgc
4081 cttttatgga ttcatgcaag gaaactaccc ataatacaag aaaagcccgt cacgggcttc
4141 tcagggcgtt ttatggcggg tctgctatgt ggtgctatct gactttttgc tgttcagcag
4201 ttcctgccct ctgattttcc agtctgacca cttcggatta tcccgtgaca ggtcattcag
4261 actggctaat gcacccagta aggcagcggt atcatcaacg ggtctgacg ctcagtggaa
4321 cgaaaactca cgttaaggga ttttggtcat gagattatca aaaaggatct tcacctagat
4381 ccttttaaat taaaaatgaa gttttaaatc aatctaaagt atatatgagt aaacttggtc
4441 tgacagttac caatgcttaa tcagtgaggc acctatctca gcgatctgtc tatttcgttc
4501 atccatagtt gcctgactcc ccgtcgtgta gataactacg atacgggagg gcttaccatc
4561 tggccccagt gctgcaatga taccgcgaga cccacgctca ccggctccag atttatcagc
4621 aataaaccag ccagccggaa gggccgagcg cagaagtggt cctgcaactt tatccgcctc
4681 catccagtct attaattgtt gccgggaagc tagagtaagt agttcgccag ttaatagttt

4741 gcgcaacgtt gttgccattg ctacaggcat cgtggtgtca cgctcgtcgt
ttggtatggc
4801 ttcattcagc tccggttccc aacgatcaag gcgagttaca tgatccccca
tgttgtgcaa
4861 aaaagcggtt agctccttcg gtcctccgat cgttgtcaga agtaagttgg
ccgcagtgtt
4921 atcactcatg gttatggcag cactgcataa ttctcttact gtcatgccat
ccgtaagatg
4981 cttttctgtg actggtgagt actcaaccaa gtcattctga gaatagtgta
tgcggcgacc
5041 gagttgctct tgcccggcgt caatacggga taataccgcg ccacatagca
gaactttaaa
5101 agtgctcatc attggaaaac gttcttcggg gcgaaaactc tcaaggatct
taccgctgtt
5161 gagatccagt tcgatgtaac ccactcgtgc acccaactga tcttcagcat
cttttacttt
5221 caccagcgtt tctgggtgag caaaaacagg aaggcaaaat gccgcaaaaa
agggaataag
5281 ggcgacacgg aaatgttgaa tactcatact cttccttttt caatattatt
gaagcattta
5341 tcagggttat tgtctcatga gcggatacat atttgaatgt atttagaaaa
ataaacaaat
5401 aggggttccg cgcacatttc cccgaaaagt gccacctg
//

```
LOCUS       pGSs050                 5396 bp    DNA     circular UNA 26-
OCT-2015
DEFINITION  CRIM helper plasmid pINT-ts, complete sequence.
ACCESSION   urn.local...50-exvbdzh
VERSION     urn.local...50-exvbdzh
KEYWORDS    .
SOURCE      CRIM helper plasmid pINT-ts (CRIM helper plasmid pINT-ts.)
  ORGANISM  CRIM helper plasmid pINT-ts
            other sequences;artificial sequences;vectors.
FEATURES             Location/Qualifiers
     misc_feature    5396
                     /Description="dimer interface [polypeptide binding];
other
                     site"
                     /db_xref="CDD:238415"
                     /gene="int"
                     /label="int"
     misc_feature    5396
                     /Description="active site"
                     /db_xref="CDD:238415"
                     /gene="int"
                     /label="int"
     misc_feature    5396
                     /Description="Int/Topo IB signature motif; other
site"
                     /db_xref="CDD:238415"
                     /gene="int"
                     /label="int"
     CDS             complement(62..775)
                     /db_xref="GI:16209182"
                     /product="cI"
                     /codon_start=1
                     /protein_id="AAL09915.1"
                     /transl_table=11
                     /label="CDS_1"
     misc_feature    complement(74..712)
                     /Description="SOS-response transcriptional
repressors
                     (RecA-mediated autopeptidases) [Transcription /
Signal
                     transduction mechanisms]; Region: LexA; COG1974"
                     /db_xref="CDD:224885"
                     /label="Misc_Feature_3"
     misc_feature    complement(95..352)
                     /Description="Peptidase S24 LexA-like proteins are
                     involved in the SOS response leading to the repair
of
                     single-stranded DNA within the bacterial cell. This
family
                     includes: the lambda repressor CI/C2 family and
related
                     bacterial prophage repressor proteins; LexA (EC...;
                     Region: S24_LexA-like; cd06529"
                     /db_xref="CDD:119397"
```

```
                     /label="Misc_Feature_1"
     misc_feature    complement(197..328)
                     /Description="Catalytic site [active]"
                     /db_xref="CDD:119397"
                     /label="Misc_Feature_4"
     misc_feature    complement(545..730)
                     /Description="Helix-turn-helix XRE-family like
proteins.
                     Prokaryotic DNA binding proteins belonging to the
                     xenobiotic response element family of
transcriptional
                     regulators; Region: HTH_XRE; cd00093"
                     /db_xref="CDD:238045"
                     /label="Misc_Feature_2"
     misc_feature    complement(614..676)
                     /Description="sequence-specific DNA binding site
                     [nucleotide binding]; other site"
                     /db_xref="CDD:238045"
                     /label="Misc_Feature_6"
     misc_feature    complement(617..718)
                     /Description="non-specific DNA binding site
[nucleotide
                     binding]; other site"
                     /db_xref="CDD:238045"
                     /label="Misc_Feature_5"
     misc_feature    complement(620..709)
                     /Description="salt bridge; other site"
                     /db_xref="CDD:238045"
                     /label="Misc_Feature_7"
     misc_feature    878..2455
                     /label="phi370int-attB_pInt-Ts"
     CDS             complement(2563..3513)
                     /db_xref="GI:16209184"
                     /product="repA101"
                     /codon_start=1
                     /protein_id="AAL09917.1"
                     /transl_table=11
                     /label="CDS_2"
     misc_feature    complement(2851..3510)
                     /Description="Initiator Replication protein; Region:
                     Rep_3; pfam01051"
                     /db_xref="CDD:250324"
                     /label="Misc_Feature_8"
     CDS             complement(4405..5265)
                     /db_xref="GI:16209185"
                     /product="bla"
                     /codon_start=1
                     /protein_id="AAL09918.1"
                     /transl_table=11
                     /label="CDS_3"
     misc_feature    complement(4408..5259)
                     /Description="beta-lactamase TEM; Provisional;
Region:
                     PRK15442"
```

```
                     /db_xref="CDD:185339"
                     /label="Misc_Feature_9"
ORIGIN
        1 catcgatgaa gattcttgct caattgttat cagctatgcg ccgaccagaa
caccttgccg
       61 atcagccaaa cgtctcttca ggccactgac tagcgataac tttccccaca
acggaacaac
      121 tctcattgca tgggatcatt gggtactgtg ggtttagtgg ttgtaaaaac
acctgaccgc
      181 tatccctgat cagtttcttg aaggtaaact catcaccccc aagtctggct
atgcagaaat
      241 cacctggctc aacagcctgc tcagggtcaa cgagaattaa cattccgtca
ggaaagcttg
      301 gcttggagcc tgttggtgcg gtcatggaat taccttcaac ctcaagccag
aatgcagaat
      361 cactggcttt tttggttgtg cttacccatc tctccgcatc acctttggta
aaggttctaa
      421 gcttaggtga gaacatccct gcctgaacat gagaaaaaac agggtactca
tactcacttc
      481 taagtgacgg ctgcatacta accgcttcat acatctcgta gatttctctg
gcgattgaag
      541 ggctaaattc ttcaacgcta actttgagaa tttttgtaag caatgcggcg
ttataagcat
      601 ttaatgcatt gatgccatta aataaagcac caacgcctga ctgccccatc
cccatcttgt
      661 ctgcgacaga ttcctgggat aagccaagtt catttttctt tttttcataa
attgctttaa
      721 ggcgacgtgc gtcctcaagc tgctcttgtg ttaatggttt ctttttttgtg
ctcatacgtt
      781 aaatctatca ccgcaaggga taaatatcta acaccgtgcg tgttgactat
tttacctctg
      841 gcggtgataa tggttgcatg tactaaggag gttgtatgaa ttcgagctcg
gtacccagtt
      901 cgtcgatggg ttcgggaatg caggatattc ccacctccgg ttaaggatgg
aagagagtat
      961 ctgttccacg aatcagcggt aaaggttgac ttaaatcgac cagtaacagg
tggccttttg
     1021 aagaggactg cagatgcgta aggtggcgat ctacagccgt gttagcacca
ttaaccaggc
     1081 ggaggaaggt tatagcatcc agggccaaat tgaagcgctg accaagtact
gcgaagcgat
     1141 ggagtggaag atctataaaa actacagcga cgcgggtttc agcggtggca
aactggagcg
     1201 tccggcgatc accgaactga ttgaggacgg caagaacaac aaatttgata
ccatcctggt
     1261 gtataagctg gaccgtctga gccgtaacgt taaggacacc ctgtacctgg
tgaaagatgt
     1321 tttcaccgcg aacaacattc actttgtgag cctgaaagaa aacatcgata
ccagcagcgc
     1381 gatgggtaac ctgttcctga ccctgctgag cgcgattgcg gagtttgaac
gtgagcagat
     1441 caaggagcgt atgcaattcg gtgttatgaa ccgtgcgaag agcggcaaaa
ccaccgcgtg
```

1501 gaaaaccccg ccgtacggct atcgttacaa caaggacgaa aaaaccctga gcgtgaacga
1561 actggaggcg gcgaacgttc gtcagatgtt cgacatgatc attagcggtt gcagcatcat
1621 gagcattacc aactatgcgc gtgataactt tgtgggcaac acctggaccc acgtgaaggt
1681 taaacgtatc ctggaaaacg agacctataa gggtctggtt aaataccgtg aacagacctt
1741 cagcggcgac caccaagcga tcattgatga gaagacctac aacaaagcgc agattgcgct
1801 ggcgcaccgt accgacacca agaccaacac ccgtccgttt cagggtaagt atatgctgag
1861 ccacatcgcg aaatgcggtt attgcggtgc gccgctgaag gtgtgcaccg gtcgtgcgaa
1921 aaacgatggc acccgtcgtc agacctatgt gtgcgttaac aaaaccgaaa gcctggcgcg
1981 tcgtagcgtt aacaactaca acaaccaaaa gatctgcaac accggccgtt acgaaaagaa
2041 acacatcgag aaatatgtga ttgacgttct gtacaagctg caacacgata aggagtacct
2101 gaagaaaatc aagaaagacg ataacatcat cgacatcacc ccgctgaaga aagaaatcga
2161 gatcatcgac aagaaaatca accgtctgaa cgatctgtac atcaacgacc tgattgatct
2221 gccgaagctg aagaaagata ttgaggaact gaaccacctg aaagacgatt ataacaaggc
2281 gatcaaactg aactacctgg acaagaaaaa cgaagatagc ctgggtatgc tgatggacaa
2341 cctggatatt cgtaagagca gctacgacgt gcagagccgt atcgttaaac aactgattga
2401 tcgtgttgag gtgaccatgg acaacatcga tatcattttc aaattttaag ctagcccatg
2461 ggtatggaca gttttccctt tgatatgtaa cggtgaacag ttgttctact tttgtttgtt
2521 agtcttgatg cttcactgat agatacaaga gccataagaa cctcagatcc ttccgtattt
2581 agccagtatg ttctctagtg tggttcgttg tttttgcgtg agccatgaga acgaaccatt
2641 gagatcatac ttactttgca tgtcactcaa aaattttgcc tcaaaactgg tgagctgaat
2701 ttttgcagtt aaagcatcgt gtagtgtttt tcttagtccg ttacgtaggt aggaatctga
2761 tgtaatggtt gttggtattt tgtcaccatt catttttatc tggttgttct caagttcggt
2821 tacgagatcc atttgtctat ctagttcaac ttggaaaatc aacgtatcag tcgggcggcc
2881 tcgcttatca accaccaatt tcatattgct gtaagtgttt aaatctttac ttattggttt
2941 caaaacccat tggttaagcc ttttaaactc atggtagtta ttttcaagca ttaacatgaa
3001 cttaaattca tcaaggctaa tctctatatt tgccttgtga gttttctttt gtgttagttc
3061 ttttaataac cactcataaa tcctcataga gtatttgttt tcaaaagact taacatgttc

3121 cagattatat tttatgaatt tttttaactg gaaaagataa ggcaatatct cttcactaaa
3181 aactaattct aatttttcgc ttgagaactt ggcatagttt gtccactgga aaatctcaaa
3241 gcctttaacc aaaggattcc tgatttccac agttctcgtc atcagctctc tggttgcttt
3301 agctaataca ccataagcat tttccctact gatgttcatc atctgagcgt attggttata
3361 agtgaacgat accgtccgtt ctttccttgt agggttttca atcgtggggt tgagtagtgc
3421 cacacagcat aaaattagct tggtttcatg ctccgttaag tcatagcgac taatcgctag
3481 ttcatttgct ttgaaaacaa ctaattcaga catacatctc aattggtcta ggtgatttta
3541 atcactatac caattgagat gggctagtca atgataatta ctagtccttt tcctttgagt
3601 tgtgggtatc tgtaaattct gctagacctt tgctggaaaa cttgtaaatt ctgctagacc
3661 ctctgtaaat tccgctagac ctttgtgtgt ttttttgtt tatattcaag tggttataat
3721 ttatagaata aagaaagaat aaaaaaagat aaaaagaata gatcccagcc ctgtgtataa
3781 ctcactactt tagtcagttc cgcagtatta caaaaggatg tcgcaaacgc tgtttgctcc
3841 tctacaaaac agaccttaaa accctaaagg cttaagtagc accctcgcaa gctcggttgc
3901 ggccgcaatc gggcaaatcg ctgaatattc cttttgtctc cgaccatcag gcacctgagt
3961 cgctgtcttt ttcgtgacat tcagttcgct gcgctcacgg ctctggcagt gaatgggggt
4021 aaatggcact acaggcgcct tttatggatt catgcaagga aactacccat aatacaagaa
4081 aagcccgtca cgggcttctc agggcgtttt atggcgggtc tgctatgtgg tgctatctga
4141 ctttttgctg ttcagcagtt cctgccctct gattttccag tctgaccact tcggattatc
4201 ccgtgacagg tcattcagac tggctaatgc acccagtaag gcagcggtat catcaacggg
4261 gtctgacgct cagtggaacg aaaactcacg ttaagggatt ttggtcatga gattatcaaa
4321 aaggatcttc acctagatcc ttttaaatta aaaatgaagt tttaaatcaa tctaaagtat
4381 atatgagtaa acttggtctg acagttacca atgcttaatc agtgaggcac ctatctcagc
4441 gatctgtcta tttcgttcat ccatagttgc ctgactcccc gtcgtgtaga taactacgat
4501 acgggagggc ttaccatctg gccccagtgc tgcaatgata ccgcgagacc cacgctcacc
4561 ggctccagat ttatcagcaa taaaccagcc agccggaagg gccgagcgca gaagtggtcc
4621 tgcaacttta tccgcctcca tccagtctat taattgttgc cgggaagcta gagtaagtag
4681 ttcgccagtt aatagtttgc gcaacgttgt tgccattgct acaggcatcg tggtgtcacg

4741 ctcgtcgttt ggtatggctt cattcagctc cggttcccaa cgatcaaggc
gagttacatg
4801 atcccccatg ttgtgcaaaa aagcggttag ctccttcggt cctccgatcg
ttgtcagaag
4861 taagttggcc gcagtgttat cactcatggt tatggcagca ctgcataatt
ctcttactgt
4921 catgccatcc gtaagatgct tttctgtgac tggtgagtac tcaaccaagt
cattctgaga
4981 atagtgtatg cggcgaccga gttgctcttg cccggcgtca atacgggata
ataccgcgcc
5041 acatagcaga actttaaaag tgctcatcat tggaaaacgt tcttcggggc
gaaaactctc
5101 aaggatctta ccgctgttga gatccagttc gatgtaaccc actcgtgcac
ccaactgatc
5161 ttcagcatct tttactttca ccagcgtttc tgggtgagca aaaacaggaa
ggcaaaatgc
5221 cgcaaaaaag ggaataaggg cgacacggaa atgttgaata ctcatactct
tcctttttca
5281 atattattga agcatttatc agggttattg tctcatgagc ggatacatat
ttgaatgtat
5341 ttagaaaaat aaacaaatag gggttccgcg cacatttccc cgaaaagtgc cacctg
//

```
LOCUS       pGSs053                 5378 bp    DNA     circular UNA 19-
NOV-2015
DEFINITION  CRIM helper plasmid pINT-ts, complete sequence.
ACCESSION   urn.local...4z-exvbdzh
VERSION     urn.local...4z-exvbdzh
KEYWORDS    .
SOURCE      CRIM helper plasmid pINT-ts (CRIM helper plasmid pINT-ts.)
  ORGANISM  CRIM helper plasmid pINT-ts
            other sequences;artificial sequences;vectors.
FEATURES             Location/Qualifiers
     misc_feature    5378
                     /Description="dimer interface [polypeptide binding];
other
                     site"
                     /db_xref="CDD:238415"
                     /gene="int"
                     /label="int"
     misc_feature    5378
                     /Description="active site"
                     /db_xref="CDD:238415"
                     /gene="int"
                     /label="int"
     misc_feature    5378
                     /Description="Int/Topo IB signature motif; other
site"
                     /db_xref="CDD:238415"
                     /gene="int"
                     /label="int"
     CDS             complement(62..775)
                     /db_xref="GI:16209182"
                     /product="cI"
                     /codon_start=1
                     /protein_id="AAL09915.1"
                     /transl_table=11
                     /label="CDS_1"
     misc_feature    complement(74..712)
                     /Description="SOS-response transcriptional
repressors
                     (RecA-mediated autopeptidases) [Transcription /
Signal
                     transduction mechanisms]; Region: LexA; COG1974"
                     /db_xref="CDD:224885"
                     /label="Misc_Feature_3"
     misc_feature    complement(95..352)
                     /Description="Peptidase S24 LexA-like proteins are
                     involved in the SOS response leading to the repair
of
                     single-stranded DNA within the bacterial cell. This
family
                     includes: the lambda repressor CI/C2 family and
related
                     bacterial prophage repressor proteins; LexA (EC...;
                     Region: S24_LexA-like; cd06529"
                     /db_xref="CDD:119397"
```

```
                     /label="Misc_Feature_1"
     misc_feature    complement(197..328)
                     /Description="Catalytic site [active]"
                     /db_xref="CDD:119397"
                     /label="Misc_Feature_4"
     misc_feature    complement(545..730)
                     /Description="Helix-turn-helix XRE-family like
proteins.
                     Prokaryotic DNA binding proteins belonging to the
                     xenobiotic response element family of
transcriptional
                     regulators; Region: HTH_XRE; cd00093"
                     /db_xref="CDD:238045"
                     /label="Misc_Feature_2"
     misc_feature    complement(614..676)
                     /Description="sequence-specific DNA binding site
                     [nucleotide binding]; other site"
                     /db_xref="CDD:238045"
                     /label="Misc_Feature_6"
     misc_feature    complement(617..718)
                     /Description="non-specific DNA binding site
[nucleotide
                     binding]; other site"
                     /db_xref="CDD:238045"
                     /label="Misc_Feature_5"
     misc_feature    complement(620..709)
                     /Description="salt bridge; other site"
                     /db_xref="CDD:238045"
                     /label="Misc_Feature_7"
     misc_feature    878..2437
                     /label="BL3-attB_pInt-Ts"
     CDS             complement(2545..3495)
                     /db_xref="GI:16209184"
                     /product="repA101"
                     /codon_start=1
                     /protein_id="AAL09917.1"
                     /transl_table=11
                     /label="CDS_2"
     misc_feature    complement(2833..3492)
                     /Description="Initiator Replication protein; Region:
                     Rep_3; pfam01051"
                     /db_xref="CDD:250324"
                     /label="Misc_Feature_8"
     CDS             complement(4387..5247)
                     /db_xref="GI:16209185"
                     /product="bla"
                     /codon_start=1
                     /protein_id="AAL09918.1"
                     /transl_table=11
                     /label="CDS_3"
     misc_feature    complement(4390..5241)
                     /Description="beta-lactamase TEM; Provisional;
Region:
                     PRK15442"
```

/db_xref="CDD:185339"
/label="Misc_Feature_9"
ORIGIN
1 catcgatgaa gattcttgct caattgttat cagctatgcg ccgaccagaa caccttgccg
61 atcagccaaa cgtctcttca ggccactgac tagcgataac tttccccaca acggaacaac
121 tctcattgca tgggatcatt gggtactgtg ggtttagtgg ttgtaaaaac acctgaccgc
181 tatccctgat cagtttcttg aaggtaaact catcaccccc aagtctggct atgcagaaat
241 cacctggctc aacagcctgc tcagggtcaa cgagaattaa cattccgtca ggaaagcttg
301 gcttggagcc tgttggtgcg gtcatggaat taccttcaac ctcaagccag aatgcagaat
361 cactggcttt tttggttgtg cttacccatc tctccgcatc acctttggta aaggttctaa
421 gcttaggtga gaacatccct gcctgaacat gagaaaaaac agggtactca tactcacttc
481 taagtgacgg ctgcatacta accgcttcat acatctcgta gatttctctg gcgattgaag
541 ggctaaattc ttcaacgcta actttgagaa tttttgtaag caatgcggcg ttataagcat
601 ttaatgcatt gatgccatta aataaagcac caacgcctga ctgccccatc cccatcttgt
661 ctgcgacaga ttcctgggat aagccaagtt cattttttctt tttttcataa attgctttaa
721 ggcgacgtgc gtcctcaagc tgctcttgtg ttaatggttt ctttttttgtg ctcatacgtt
781 aaatctatca ccgcaaggga taaatatcta acaccgtgcg tgttgactat tttacctctg
841 gcggtgataa tggttgcatg tactaaggag gttgtatgaa ttcgagctcg gtacccagtt
901 cgtcgatggg ttcgggaatg caggatattc ccacctccgg ttaaggatgg aagagagtat
961 ctgttccacg aatcagcggt aaaggttgac ttaaatcgac cagtaacagg tggccttttg
1021 aagaggactg cagatgaaac tgcgtgcggc gatttacgtg cgtgttagca ccatggagca
1081 ggcggaggaa ggttatagca tcagcgcgca aaccgaaaaa ctgaagagct acgcgaacgc
1141 gaaagactat caggtggtta aggtgttcac cgatccgggt tacagcggtg cgaaactgga
1201 gcgtccgggt ctgcaaaaca tgatcaaaag cattgagagc aaggaaattg acgtggttct
1261 ggtttataaa ctggatcgtc tgagccgtag ccagaagaac accctgttcc tgatcgagga
1321 cgtgtttctg aagaaccacg ttcagttcac cagcatgcaa gaaagcttcg ataccagcac
1381 cagctttggt cgtgcgatga tcggcattct gagcgtgttt gcgcagctgg agcgtgacgc
1441 gatcaccgaa cgtatgcaaa tgggtgcgaa agagcgtgcg aaagcgggca tgtggcgtgg

1501 tggcccgcag agccgtctgc cgttcggtta ccgttatatt gatggcgtgc tgctggttga
1561 cgattacgag gcgatgatcg ttaaatacat gtataccgag ttcattaagg gtaccccgct
1621 gaccaaaatc cagagcaagg tggcggcgaa atttccggtt aaggaaaccc tgatctaccc
1681 gagcattatg aagaacatcc tgcaaaacaa catctacatc ggcaagatca agtacgcggg
1741 cgagacctat gaaggcctgc acgagcacat cctggacacc gaaacctacg ataaagcgca
1801 gcaactgtgg gagcaccgta acaccaacaa gaaaaagtac ttcgaaagca agtatctgct
1861 gagcggtatt ctgtattgcg gccactgcgg tggcaaaatg gcgagcaccg gtgcgggtct
1921 gctgaagagc ggcgagcgtg tgaccgacta catctgctat agcaaaaagg gcaccccgag
1981 ccacatggtg gttgaccgta actgcccgag caagcgtcac cgtgtgaacc gtctggaccc
2041 gaaaattgtt gaactgctga agaccatcac cttcgaggaa atgcagaaag acaacagctt
2101 taccgataac accaccacca ttaagagcga gatcgaaagc ctggacacca aaatcagcaa
2161 gctgctggac ctgtaccaag atggtctggt gccgattgac gttctgaacg atcgtatcag
2221 caaactgaac gacgataagg agctgctgca agaaaccctg attagccaga aaaagcaaat
2281 ccacccggag gaaattgcga aaaacatcca aaccgcgaag gactttgatt gggcgaacag
2341 cgacagcgcg gcgaagcgtg cgatggtgcg tgcgctgatc aacaaagttg agctgaccaa
2401 cgaagatatg aagattgaat ggaacatcta agctagccca tgggtatgga cagttttccc
2461 tttgatatgt aacggtgaac agttgttcta cttttgtttg ttagtcttga tgcttcactg
2521 atagatacaa gagccataag aacctcagat ccttccgtat ttagccagta tgttctctag
2581 tgtggttcgt tgtttttgcg tgagccatga gaacgaacca ttgagatcat acttactttg
2641 catgtcactc aaaaattttg cctcaaaact ggtgagctga atttttgcag ttaaagcatc
2701 gtgtagtgtt tttcttagtc cgttacgtag gtaggaatct gatgtaatgg ttgttggtat
2761 tttgtcacca ttcattttta tctggttgtt ctcaagttcg gttacgagat ccatttgtct
2821 atctagttca acttggaaaa tcaacgtatc agtcgggcgg cctcgcttat caaccaccaa
2881 tttcatattg ctgtaagtgt ttaaatcttt acttattggt ttcaaaaccc attggttaag
2941 ccttttaaac tcatggtagt tattttcaag cattaacatg aacttaaatt catcaaggct
3001 aatctctata tttgccttgt gagttttctt ttgtgttagt tcttttaata accactcata
3061 aatcctcata gagtatttgt tttcaaaaga cttaacatgt tccagattat attttatgaa

3121 ttttttaac tggaaaagat aaggcaatat ctcttcacta aaaactaatt
ctaattttc
3181 gcttgagaac ttggcatagt ttgtccactg gaaaatctca aagcctttaa
ccaaaggatt
3241 cctgatttcc acagttctcg tcatcagctc tctggttgct ttagctaata
caccataagc
3301 attttcccta ctgatgttca tcatctgagc gtattggtta taagtgaacg
ataccgtccg
3361 ttctttcctt gtagggtttt caatcgtggg gttgagtagt gccacacagc
ataaaattag
3421 cttggtttca tgctccgtta agtcatagcg actaatcgct agttcatttg
ctttgaaaac
3481 aactaattca gacatacatc tcaattggtc taggtgattt taatcactat
accaattgag
3541 atgggctagt caatgataat tactagtcct tttcctttga gttgtgggta
tctgtaaatt
3601 ctgctagacc tttgctggaa aacttgtaaa ttctgctaga ccctctgtaa
attccgctag
3661 acctttgtgt gttttttttg tttatattca agtggttata atttatagaa
taaagaaaga
3721 ataaaaaaag ataaaaagaa tagatcccag ccctgtgtat aactcactac
tttagtcagt
3781 tccgcagtat tacaaaagga tgtcgcaaac gctgtttgct cctctacaaa
acagacctta
3841 aaaccctaaa ggcttaagta gcaccctcgc aagctcggtt gcggccgcaa
tcgggcaaat
3901 cgctgaatat tccttttgtc tccgaccatc aggcacctga gtcgctgtct
ttttcgtgac
3961 attcagttcg ctgcgctcac ggctctggca gtgaatgggg gtaaatggca
ctacaggcgc
4021 cttttatgga ttcatgcaag gaaactaccc ataatacaag aaaagcccgt
cacgggcttc
4081 tcagggcgtt ttatggcggg tctgctatgt ggtgctatct gactttttgc
tgttcagcag
4141 ttcctgccct ctgattttcc agtctgacca cttcggatta tcccgtgaca
ggtcattcag
4201 actggctaat gcacccagta aggcagcggt atcatcaacg ggtctgacg
ctcagtggaa
4261 cgaaaactca cgttaaggga ttttggtcat gagattatca aaaaggatct
tcacctagat
4321 ccttttaaat taaaaatgaa gttttaaatc aatctaaagt atatatgagt
aaacttggtc
4381 tgacagttac caatgcttaa tcagtgaggc acctatctca gcgatctgtc
tatttcgttc
4441 atccatagtt gcctgactcc ccgtcgtgta gataactacg atacgggagg
gcttaccatc
4501 tggccccagt gctgcaatga taccgcgaga cccacgctca ccggctccag
atttatcagc
4561 aataaaccag ccagccggaa gggccgagcg cagaagtggt cctgcaactt
tatccgcctc
4621 catccagtct attaattgtt gccgggaagc tagagtaagt agttcgccag
ttaatagttt
4681 gcgcaacgtt gttgccattg ctacaggcat cgtggtgtca cgctcgtcgt
ttggtatggc

4741 ttcattcagc tccggttccc aacgatcaag gcgagttaca tgatccccca tgttgtgcaa
4801 aaaagcggtt agctccttcg gtcctccgat cgttgtcaga agtaagttgg ccgcagtgtt
4861 atcactcatg gttatggcag cactgcataa ttctcttact gtcatgccat ccgtaagatg
4921 cttttctgtg actggtgagt actcaaccaa gtcattctga gaatagtgta tgcggcgacc
4981 gagttgctct tgcccggcgt caatacggga taataccgcg ccacatagca gaactttaaa
5041 agtgctcatc attggaaaac gttcttcggg gcgaaaactc tcaaggatct taccgctgtt
5101 gagatccagt tcgatgtaac ccactcgtgc acccaactga tcttcagcat cttttacttt
5161 caccagcgtt tctgggtgag caaaaacagg aaggcaaaat gccgcaaaaa agggaataag
5221 ggcgacacgg aaatgttgaa tactcatact cttccttttt caatattatt gaagcattta
5281 tcagggttat tgtctcatga gcggatacat atttgaatgt atttagaaaa ataaacaaat
5341 aggggttccg cgcacatttc cccgaaaagt gccacctg
//

```
LOCUS       pGSs054                 5765 bp    DNA     circular UNA 08-
DEC-2015
DEFINITION  CRIM helper plasmid pINT-ts, complete sequence.
ACCESSION   urn.local...4y-exvbdzh
VERSION     urn.local...4y-exvbdzh
KEYWORDS    .
SOURCE      CRIM helper plasmid pINT-ts (CRIM helper plasmid pINT-ts.)
  ORGANISM  CRIM helper plasmid pINT-ts
            other sequences;artificial sequences;vectors.
FEATURES             Location/Qualifiers
     misc_feature    5765
                     /Description="dimer interface [polypeptide binding];
other
                     site"
                     /db_xref="CDD:238415"
                     /gene="int"
                     /label="int"
     misc_feature    5765
                     /Description="active site"
                     /db_xref="CDD:238415"
                     /gene="int"
                     /label="int"
     misc_feature    5765
                     /Description="Int/Topo IB signature motif; other
site"
                     /db_xref="CDD:238415"
                     /gene="int"
                     /label="int"
     CDS             complement(62..775)
                     /db_xref="GI:16209182"
                     /product="cI"
                     /codon_start=1
                     /protein_id="AAL09915.1"
                     /transl_table=11
                     /label="CDS_1"
     misc_feature    complement(74..712)
                     /Description="SOS-response transcriptional
repressors
                     (RecA-mediated autopeptidases) [Transcription /
Signal
                     transduction mechanisms]; Region: LexA; COG1974"
                     /db_xref="CDD:224885"
                     /label="Misc_Feature_3"
     misc_feature    complement(95..352)
                     /Description="Peptidase S24 LexA-like proteins are
                     involved in the SOS response leading to the repair
of
                     single-stranded DNA within the bacterial cell. This
family
                     includes: the lambda repressor CI/C2 family and
related
                     bacterial prophage repressor proteins; LexA (EC...;
                     Region: S24_LexA-like; cd06529"
                     /db_xref="CDD:119397"
```

```
                     /label="Misc_Feature_1"
     misc_feature    complement(197..328)
                     /Description="Catalytic site [active]"
                     /db_xref="CDD:119397"
                     /label="Misc_Feature_4"
     misc_feature    complement(545..730)
                     /Description="Helix-turn-helix XRE-family like
proteins.
                     Prokaryotic DNA binding proteins belonging to the
                     xenobiotic response element family of
transcriptional
                     regulators; Region: HTH_XRE; cd00093"
                     /db_xref="CDD:238045"
                     /label="Misc_Feature_2"
     misc_feature    complement(614..676)
                     /Description="sequence-specific DNA binding site
                     [nucleotide binding]; other site"
                     /db_xref="CDD:238045"
                     /label="Misc_Feature_6"
     misc_feature    complement(617..718)
                     /Description="non-specific DNA binding site
[nucleotide
                     binding]; other site"
                     /db_xref="CDD:238045"
                     /label="Misc_Feature_5"
     misc_feature    complement(620..709)
                     /Description="salt bridge; other site"
                     /db_xref="CDD:238045"
                     /label="Misc_Feature_7"
     misc_feature    878..2824
                     /label="phiBTint-attB_pInt-Ts"
     CDS             complement(2932..3882)
                     /db_xref="GI:16209184"
                     /product="repA101"
                     /codon_start=1
                     /protein_id="AAL09917.1"
                     /transl_table=11
                     /label="CDS_2"
     misc_feature    complement(3220..3879)
                     /Description="Initiator Replication protein; Region:
                     Rep_3; pfam01051"
                     /db_xref="CDD:250324"
                     /label="Misc_Feature_8"
     CDS             complement(4774..5634)
                     /db_xref="GI:16209185"
                     /product="bla"
                     /codon_start=1
                     /protein_id="AAL09918.1"
                     /transl_table=11
                     /label="CDS_3"
     misc_feature    complement(4777..5628)
                     /Description="beta-lactamase TEM; Provisional;
Region:
                     PRK15442"
```

```
                     /db_xref="CDD:185339"
                     /label="Misc_Feature_9"
ORIGIN
        1 catcgatgaa gattcttgct caattgttat cagctatgcg ccgaccagaa
caccttgccg
       61 atcagccaaa cgtctcttca ggccactgac tagcgataac tttccccaca
acggaacaac
      121 tctcattgca tgggatcatt gggtactgtg ggtttagtgg ttgtaaaaac
acctgaccgc
      181 tatccctgat cagtttcttg aaggtaaact catcaccccc aagtctggct
atgcagaaat
      241 cacctggctc aacagcctgc tcagggtcaa cgagaattaa cattccgtca
ggaaagcttg
      301 gcttggagcc tgttggtgcg gtcatggaat taccttcaac ctcaagccag
aatgcagaat
      361 cactggcttt tttggttgtg cttacccatc tctccgcatc acctttggta
aaggttctaa
      421 gcttaggtga gaacatccct gcctgaacat gagaaaaaac agggtactca
tactcacttc
      481 taagtgacgg ctgcatacta accgcttcat acatctcgta gatttctctg
gcgattgaag
      541 ggctaaattc ttcaacgcta actttgagaa tttttgtaag caatgcggcg
ttataagcat
      601 ttaatgcatt gatgccatta aataaagcac caacgcctga ctgccccatc
cccatcttgt
      661 ctgcgacaga ttcctgggat aagccaagtt catttttctt tttttcataa
attgctttaa
      721 ggcgacgtgc gtcctcaagc tgctcttgtg ttaatggttt ctttttttgtg
ctcatacgtt
      781 aaatctatca ccgcaaggga taaatatcta acaccgtgcg tgttgactat
tttacctctg
      841 gcggtgataa tggttgcatg tactaaggag gttgtatgaa ttcgagctcg
gtacccagtt
      901 cgtcgatggg ttcgggaatg caggatattc ccacctccgg ttaaggatgg
aagagagtat
      961 ctgttccacg aatcagcggt aaaggttgac ttaaatcgac cagtaacagg
tggccttttg
     1021 aagaggactg cagatgagcc cgttcattgc gccggatgtg ccggagcacc
tgctggacac
     1081 cgtgcgtgtt tttctgtacg cgcgtcagag caaaggtcgt agcgatggca
gcgatgtgag
     1141 caccgaggcg cagctggcgg cgggtcgtgc gctggttgcg agccgtaacg
cgcaaggtgg
     1201 cgcgcgttgg gtggttgcgg gtgagttcgt ggatgttggt cgtagcggtt
gggacccgaa
     1261 cgtgacccgt gcggattttg agcgtatgat gggtgaagtt cgtgcgggcg
agggcgacgt
     1321 ggttgtggtt aacgagctga gccgtctgac ccgtaagggt gcgcacgatg
cgctggagat
     1381 cgacaacgaa ctgaagaaac acggcgtgcg tttcatgagc gttctggaac
cgtttctgga
     1441 caccagcacc ccgatcggtg tggcgatttt cgcgctgatc gcggcgctgg
cgaagcaaga
```

1501 cagcgatctg aaagcggagc gtctgaaggg tgcgaaagat gaaattgcgg cgctgggtgg
1561 cgttcacagc agcagcgcgc cgtttggtat gcgtgcggtg cgtaagaaag ttgacaacct
1621 ggtgatcagc gttctggagc cggacgaaga taacccggat cacgtggagc tggttgaacg
1681 tatggcgaaa atgagcttcg agggtgttag cgacaacgcg attgcgacca cctttgagaa
1741 ggaaaaaatt ccgagcccgg gtatggcgga acgtcgtgcg accgagaagc gtctggcgag
1801 cgtgaaagcg cgtcgtctga acggtgcgga gaagccgatc atgtggcgtg cgcagaccgt
1861 tcgttggatt ctgaaccacc cggcgatcgg tggcttcgcg tttgaacgtg tgaagcacgg
1921 caaagcgcac atcaacgtta ttcgtcgtga cccgggtggc aaaccgctga ccccgcacac
1981 cggtattctg agcggcagca agtggctgga gctgcaagaa aagcgtagcg gtaaaaacct
2041 gagcgatcgt aaaccgggtg cggaagtgga accgaccctg ctgagcggtt ggcgtttcct
2101 gggctgccgt atctgcggtg gcagcatggg tcagagccaa ggtggccgta agcgtaacgg
2161 tgacctggcg gaaggcaact acatgtgcgc gaacccgaaa ggccacggtg gcctgagcgt
2221 taagcgtagc gagctggatg agttcgtggc gagcaaagtt tgggcgcgtc tgcgtaccgc
2281 ggacatggag gatgaacacg atcaagcgtg gatcgcggcg gcggcggagc gttttgcgct
2341 gcaacatgat ctggcgggtg tggcggatga gcgtcgtgaa cagcaagcgc acctggacaa
2401 cgttcgtcgt agcatcaaag acctgcaagc ggaccgtaag ccgggtctgt atgtgggccg
2461 tgaggaactg gaaacctggc gtagcaccgt tctgcaatac cgtagctatg aggcggaatg
2521 caccacccgt ctggcggagc tggatgaaaa gatgaacggt agcacccgtg tgccgagcga
2581 atggtttagc ggtgaggacc cgaccgcgga aggtggcatt tgggcgagct gggatgttta
2641 cgagcgtcgt gaatttctga gcttctttct ggatagcgtg atggttgacc gtggtcgtca
2701 cccggagacc aagaaatata tcccgctgaa ggaccgtgtg accctgaaat gggcggaact
2761 gctgaaggaa gaggacgagg cgagcgaagc gaccgagcgt gaactggcgg cgctgtaagc
2821 tagcccatgg gtatggacag ttttcccttt gatatgtaac ggtgaacagt tgttctactt
2881 ttgtttgtta gtcttgatgc ttcactgata gatacaagag ccataagaac ctcagatcct
2941 tccgtattta gccagtatgt tctctagtgt ggttcgttgt ttttgcgtga gccatgagaa
3001 cgaaccattg agatcatact tactttgcat gtcactcaaa aattttgcct caaaactggt
3061 gagctgaatt tttgcagtta aagcatcgtg tagtgttttt cttagtccgt tacgtaggta

3121 ggaatctgat gtaatggttg ttggtatttt gtcaccattc atttttatct
ggttgttctc
3181 aagttcggtt acgagatcca tttgtctatc tagttcaact tggaaaatca
acgtatcagt
3241 cgggcggcct cgcttatcaa ccaccaattt catattgctg taagtgttta
aatctttact
3301 tattggtttc aaaacccatt ggttaagcct tttaaactca tggtagttat
tttcaagcat
3361 taacatgaac ttaaattcat caaggctaat ctctatattt gccttgtgag
ttttcttttg
3421 tgttagttct tttaataacc actcataaat cctcatagag tatttgtttt
caaaagactt
3481 aacatgttcc agattatatt ttatgaattt ttttaactgg aaaagataag
gcaatatctc
3541 ttcactaaaa actaattcta atttttcgct tgagaacttg gcatagtttg
tccactggaa
3601 aatctcaaag cctttaacca aaggattcct gatttccaca gttctcgtca
tcagctctct
3661 ggttgcttta gctaatacac cataagcatt ttccctactg atgttcatca
tctgagcgta
3721 ttggttataa gtgaacgata ccgtccgttc tttccttgta gggttttcaa
tcgtggggtt
3781 gagtagtgcc acacagcata aaattagctt ggtttcatgc tccgttaagt
catagcgact
3841 aatcgctagt tcatttgctt tgaaaacaac taattcagac atacatctca
attggtctag
3901 gtgattttaa tcactatacc aattgagatg ggctagtcaa tgataattac
tagtcctttt
3961 cctttgagtt gtgggtatct gtaaattctg ctagaccttt gctggaaaac
ttgtaaattc
4021 tgctagaccc tctgtaaatt ccgctagacc tttgtgtgtt ttttttgttt
atattcaagt
4081 ggttataatt tatagaataa agaaagaata aaaaaagata aaaagaatag
atcccagccc
4141 tgtgtataac tcactacttt agtcagttcc gcagtattac aaaaggatgt
cgcaaacgct
4201 gtttgctcct ctacaaaaca gaccttaaaa ccctaaaggc ttaagtagca
ccctcgcaag
4261 ctcggttgcg gccgcaatcg ggcaaatcgc tgaatattcc ttttgtctcc
gaccatcagg
4321 cacctgagtc gctgtctttt tcgtgacatt cagttcgctg cgctcacggc
tctggcagtg
4381 aatgggggta aatggcacta caggcgcctt ttatggattc atgcaaggaa
actacccata
4441 atacaagaaa agcccgtcac gggcttctca gggcgtttta tggcgggtct
gctatgtggt
4501 gctatctgac tttttgctgt tcagcagttc ctgccctctg attttccagt
ctgaccactt
4561 cggattatcc cgtgacaggt cattcagact ggctaatgca cccagtaagg
cagcggtatc
4621 atcaacgggg tctgacgctc agtggaacga aaactcacgt taagggattt
tggtcatgag
4681 attatcaaaa aggatcttca cctagatcct tttaaattaa aaatgaagtt
ttaaatcaat

4741 ctaaagtata tatgagtaaa cttggtctga cagttaccaa tgcttaatca
gtgaggcacc
4801 tatctcagcg atctgtctat ttcgttcatc catagttgcc tgactccccg
tcgtgtagat
4861 aactacgata cgggagggct taccatctgg ccccagtgct gcaatgatac
cgcgagaccc
4921 acgctcaccg gctccagatt tatcagcaat aaaccagcca gccggaaggg
ccgagcgcag
4981 aagtggtcct gcaactttat ccgcctccat ccagtctatt aattgttgcc
gggaagctag
5041 agtaagtagt tcgccagtta atagtttgcg caacgttgtt gccattgcta
caggcatcgt
5101 ggtgtcacgc tcgtcgtttg gtatggcttc attcagctcc ggttcccaac
gatcaaggcg
5161 agttacatga tcccccatgt tgtgcaaaaa agcggttagc tccttcggtc
ctccgatcgt
5221 tgtcagaagt aagttggccg cagtgttatc actcatggtt atggcagcac
tgcataattc
5281 tcttactgtc atgccatccg taagatgctt ttctgtgact ggtgagtact
caaccaagtc
5341 attctgagaa tagtgtatgc ggcgaccgag ttgctcttgc ccggcgtcaa
tacgggataa
5401 taccgcgcca catagcagaa ctttaaaagt gctcatcatt ggaaaacgtt
cttcggggcg
5461 aaaactctca aggatcttac cgctgttgag atccagttcg atgtaaccca
ctcgtgcacc
5521 caactgatct tcagcatctt ttactttcac cagcgtttct gggtgagcaa
aaacaggaag
5581 gcaaaatgcc gcaaaaaagg gaataagggc gacacggaaa tgttgaatac
tcatactctt
5641 cctttttcaa tattattgaa gcatttatca gggttattgt ctcatgagcg
gatacatatt
5701 tgaatgtatt tagaaaaata aacaaatagg ggttccgcgc acatttcccc
gaaaagtgcc
5761 acctg
//

```
LOCUS       pGSs082                 5426 bp    DNA     circular UNA 08-
DEC-2015
DEFINITION  CRIM helper plasmid pINT-ts, complete sequence.
ACCESSION   urn.local...4x-exvbdzh
VERSION     urn.local...4x-exvbdzh
KEYWORDS    .
SOURCE      CRIM helper plasmid pINT-ts (CRIM helper plasmid pINT-ts.)
  ORGANISM  CRIM helper plasmid pINT-ts
            other sequences;artificial sequences;vectors.
FEATURES             Location/Qualifiers
     misc_feature    5426
                     /Description="dimer interface [polypeptide binding];
other
                     site"
                     /db_xref="CDD:238415"
                     /gene="int"
                     /label="int"
     misc_feature    5426
                     /Description="active site"
                     /db_xref="CDD:238415"
                     /gene="int"
                     /label="int"
     misc_feature    5426
                     /Description="Int/Topo IB signature motif; other
site"
                     /db_xref="CDD:238415"
                     /gene="int"
                     /label="int"
     CDS             complement(62..775)
                     /db_xref="GI:16209182"
                     /product="cI"
                     /codon_start=1
                     /protein_id="AAL09915.1"
                     /transl_table=11
                     /label="CDS_1"
     misc_feature    complement(74..712)
                     /Description="SOS-response transcriptional
repressors
                     (RecA-mediated autopeptidases) [Transcription /
Signal
                     transduction mechanisms]; Region: LexA; COG1974"
                     /db_xref="CDD:224885"
                     /label="Misc_Feature_3"
     misc_feature    complement(95..352)
                     /Description="Peptidase S24 LexA-like proteins are
                     involved in the SOS response leading to the repair
of
                     single-stranded DNA within the bacterial cell. This
family
                     includes: the lambda repressor CI/C2 family and
related
                     bacterial prophage repressor proteins; LexA (EC...;
                     Region: S24_LexA-like; cd06529"
                     /db_xref="CDD:119397"
```

```
                     /label="Misc_Feature_1"
     misc_feature    complement(197..328)
                     /Description="Catalytic site [active]"
                     /db_xref="CDD:119397"
                     /label="Misc_Feature_4"
     misc_feature    complement(545..730)
                     /Description="Helix-turn-helix XRE-family like
proteins.
                     Prokaryotic DNA binding proteins belonging to the
                     xenobiotic response element family of
transcriptional
                     regulators; Region: HTH_XRE; cd00093"
                     /db_xref="CDD:238045"
                     /label="Misc_Feature_2"
     misc_feature    complement(614..676)
                     /Description="sequence-specific DNA binding site
                     [nucleotide binding]; other site"
                     /db_xref="CDD:238045"
                     /label="Misc_Feature_6"
     misc_feature    complement(617..718)
                     /Description="non-specific DNA binding site
[nucleotide
                     binding]; other site"
                     /db_xref="CDD:238045"
                     /label="Misc_Feature_5"
     misc_feature    complement(620..709)
                     /Description="salt bridge; other site"
                     /db_xref="CDD:238045"
                     /label="Misc_Feature_7"
     misc_feature    878..2485
                     /label="Wbetaint-attB_pInt-Ts"
     CDS             complement(2593..3543)
                     /db_xref="GI:16209184"
                     /product="repA101"
                     /codon_start=1
                     /protein_id="AAL09917.1"
                     /transl_table=11
                     /label="CDS_2"
     misc_feature    complement(2881..3540)
                     /Description="Initiator Replication protein; Region:
                     Rep_3; pfam01051"
                     /db_xref="CDD:250324"
                     /label="Misc_Feature_8"
     CDS             complement(4435..5295)
                     /db_xref="GI:16209185"
                     /product="bla"
                     /codon_start=1
                     /protein_id="AAL09918.1"
                     /transl_table=11
                     /label="CDS_3"
     misc_feature    complement(4438..5289)
                     /Description="beta-lactamase TEM; Provisional;
Region:
                     PRK15442"
```

```
                     /db_xref="CDD:185339"
                     /label="Misc_Feature_9"
ORIGIN
        1 catcgatgaa gattcttgct caattgttat cagctatgcg ccgaccagaa
caccttgccg
       61 atcagccaaa cgtctcttca ggccactgac tagcgataac tttccccaca
acggaacaac
      121 tctcattgca tgggatcatt gggtactgtg ggtttagtgg ttgtaaaaac
acctgaccgc
      181 tatccctgat cagtttcttg aaggtaaact catcaccccc aagtctggct
atgcagaaat
      241 cacctggctc aacagcctgc tcagggtcaa cgagaattaa cattccgtca
ggaaagcttg
      301 gcttggagcc tgttggtgcg gtcatggaat taccttcaac ctcaagccag
aatgcagaat
      361 cactggcttt tttggttgtg cttacccatc tctccgcatc acctttggta
aaggttctaa
      421 gcttaggtga gaacatccct gcctgaacat gagaaaaaac agggtactca
tactcacttc
      481 taagtgacgg ctgcatacta accgcttcat acatctcgta gatttctctg
gcgattgaag
      541 ggctaaattc ttcaacgcta actttgagaa tttttgtaag caatgcggcg
ttataagcat
      601 ttaatgcatt gatgccatta aataaagcac caacgcctga ctgccccatc
cccatcttgt
      661 ctgcgacaga ttcctgggat aagccaagtt cattttctt tttttcataa
attgctttaa
      721 ggcgacgtgc gtcctcaagc tgctcttgtg ttaatggttt ctttttgtg
ctcatacgtt
      781 aaatctatca ccgcaaggga taaatatcta acaccgtgcg tgttgactat
tttacctctg
      841 gcggtgataa tggttgcatg tactaaggag gttgtatgaa ttcgagctcg
gtacccagtt
      901 cgtcgatggg ttcgggaatg caggatattc ccacctccgg ttaaggatgg
aagagagtat
      961 ctgttccacg aatcagcggt aaaggttgac ttaaatcgac cagtaacagg
tggcctttg
     1021 aagaggactg cagatgaagt atgcggttta cgtgcgtgtg agcaccgacc
gtgacgaaca
     1081 agtgagcagc gttgagaatc agattgacat ttgccgttac tggctggaga
aaaacggtta
     1141 cgaatgggac ccgaacgcgg tgtattttga cgatggtatt agcggtaccg
cgtggctgga
     1201 gcgtcatgcg atgcagctga ttctggagaa agcgcgtcgt aacgaactgg
acaccgtggt
     1261 tttcaagagc atccaccgtc tggcgcgtga cctgcgtgat gcgctggaga
ttaaggaaat
     1321 cctgattggt cacggcatcc gtctggttac cattgaggaa aactacgaca
gcctgtatga
     1381 gggtggcaac gatatcaaat tcgaaatgtt tgcgatgttc gcggcgcagc
tgccgaagac
     1441 cattagcgtg agcgttagcg cggcgatgca agcgaaagcg cgtcgtggtg
agtttatcgg
```

1501 caagccgggt ctgggctacg acgtgatcga taagaaactg gttattaacg agaaagaagc
1561 ggagatcgtg cgtgaaattt ttgatctgag ctacaagggt tatggcttca agaaaatcgc
1621 gaacattctg aacgacaaag gtacctatac caagtttggt cagctgtgga gccacaccac
1681 cgtgggcaag atcctgaaaa accaaaccta caaaggcaac ctggttctga acagctataa
1741 gaccgtgaaa gttgatggta agaaaaagcg tgtttacacc ccgaaagagc gtctgaccat
1801 cattgaagac cactatccga ccatcgtgag caaggaactg tggaacgcgg ttaacagcga
1861 tcgtgcgagc aaaaagaaaa ccaaacaaga cacccgtaac gagtttcgtg gtatgatgtt
1921 ctgcaagcac tgcggcgaac cgattaccgc gaaatacagc ggtcgttatg cgaagggcag
1981 caagaaagag tgggtgtaca tgaagtgcag caactatatc cgttttaacc gttgcgttaa
2041 cttcgatccg gcgcactacg acgatattcg tgaagcgatc atttatggtc tgaagcagca
2101 agaaaaagag ctggaaatcc actttaaccc gaagatgcac cagaaacgta acgacaagag
2161 caccgagatc aagaaacaaa ttaagctgct gaaagtgaag aaagaaaaac tgatcgacct
2221 gtacgttgag ggcctgattg ataaagaaat gtttagcaag cgtgacctga acttcgagaa
2281 cgaaattaaa gagcaggaac tggcgctgct gaagctgacc gaccaaaaca agcgtaacaa
2341 agaggaaaag aaaatcaaag aggcgtttag catgctggac gaggaaaaag atatgcacga
2401 agtgttcaag accctgatta agaaaatcac cctgagcaag gacaaataca ttgacattga
2461 atacaccttt agcctgtaag ctagcccatg ggtatggaca gttttccctt tgatatgtaa
2521 cggtgaacag ttgttctact tttgtttgtt agtcttgatg cttcactgat agatacaaga
2581 gccataagaa cctcagatcc ttccgtattt agccagtatg ttctctagtg tggttcgttg
2641 tttttgcgtg agccatgaga acgaaccatt gagatcatac ttactttgca tgtcactcaa
2701 aaattttgcc tcaaaactgg tgagctgaat ttttgcagtt aaagcatcgt gtagtgtttt
2761 tcttagtccg ttacgtaggt aggaatctga tgtaatggtt gttggtattt tgtcaccatt
2821 catttttatc tggttgttct caagttcggt tacgagatcc atttgtctat ctagttcaac
2881 ttggaaaatc aacgtatcag tcgggcggcc tcgcttatca accaccaatt tcatattgct
2941 gtaagtgttt aaatctttac ttattggttt caaaacccat tggttaagcc ttttaaactc
3001 atggtagtta ttttcaagca ttaacatgaa cttaaattca tcaaggctaa tctctatatt
3061 tgccttgtga gttttctttt gtgttagttc ttttaataac cactcataaa tcctcataga

3121 gtatttgttt tcaaaagact taacatgttc cagattatat tttatgaatt tttttaactg
3181 gaaaagataa ggcaatatct cttcactaaa aactaattct aatttttcgc ttgagaactt
3241 ggcatagttt gtccactgga aaatctcaaa gcctttaacc aaaggattcc tgatttccac
3301 agttctcgtc atcagctctc tggttgcttt agctaataca ccataagcat tttccctact
3361 gatgttcatc atctgagcgt attggttata agtgaacgat accgtccgtt ctttccttgt
3421 agggttttca atcgtggggt tgagtagtgc cacacagcat aaaattagct tggtttcatg
3481 ctccgttaag tcatagcgac taatcgctag ttcatttgct ttgaaaacaa ctaattcaga
3541 catacatctc aattggtcta ggtgatttta atcactatac caattgagat gggctagtca
3601 atgataatta ctagtccttt tcctttgagt tgtgggtatc tgtaaattct gctagacctt
3661 tgctggaaaa cttgtaaatt ctgctagacc ctctgtaaat tccgctagac ctttgtgtgt
3721 ttttttgtt tatattcaag tggttataat ttatagaata aagaaagaat aaaaaaagat
3781 aaaaagaata gatcccagcc ctgtgtataa ctcactactt tagtcagttc cgcagtatta
3841 caaaaggatg tcgcaaacgc tgtttgctcc tctacaaaac agaccttaaa accctaaagg
3901 cttaagtagc accctcgcaa gctcggttgc ggccgcaatc gggcaaatcg ctgaatattc
3961 cttttgtctc cgaccatcag gcacctgagt cgctgtcttt ttcgtgacat tcagttcgct
4021 gcgctcacgg ctctggcagt gaatgggggt aaatggcact acaggcgcct tttatggatt
4081 catgcaagga aactacccat aatacaagaa aagcccgtca cgggcttctc agggcgtttt
4141 atggcgggtc tgctatgtgg tgctatctga ctttttgctg ttcagcagtt cctgccctct
4201 gattttccag tctgaccact tcggattatc ccgtgacagg tcattcagac tggctaatgc
4261 acccagtaag gcagcggtat catcaacggg gtctgacgct cagtggaacg aaaactcacg
4321 ttaagggatt ttggtcatga gattatcaaa aaggatcttc acctagatcc ttttaaatta
4381 aaaatgaagt tttaaatcaa tctaaagtat atatgagtaa acttggtctg acagttacca
4441 atgcttaatc agtgaggcac ctatctcagc gatctgtcta tttcgttcat ccatagttgc
4501 ctgactcccc gtcgtgtaga taactacgat acgggagggc ttaccatctg gccccagtgc
4561 tgcaatgata ccgcgagacc cacgctcacc ggctccagat ttatcagcaa taaaccagcc
4621 agccggaagg gccgagcgca gaagtggtcc tgcaacttta tccgcctcca tccagtctat
4681 taattgttgc cgggaagcta gagtaagtag ttcgccagtt aatagtttgc gcaacgttgt

4741 tgccattgct acaggcatcg tggtgtcacg ctcgtcgttt ggtatggctt
cattcagctc
4801 cggttcccaa cgatcaaggc gagttacatg atcccccatg ttgtgcaaaa
aagcggttag
4861 ctccttcggt cctccgatcg ttgtcagaag taagttggcc gcagtgttat
cactcatggt
4921 tatggcagca ctgcataatt ctcttactgt catgccatcc gtaagatgct
tttctgtgac
4981 tggtgagtac tcaaccaagt cattctgaga atagtgtatg cggcgaccga
gttgctcttg
5041 cccggcgtca atacgggata ataccgcgcc acatagcaga actttaaaag
tgctcatcat
5101 tggaaaacgt tcttcggggc gaaaactctc aaggatctta ccgctgttga
gatccagttc
5161 gatgtaaccc actcgtgcac ccaactgatc ttcagcatct tttactttca
ccagcgtttc
5221 tgggtgagca aaaacaggaa ggcaaaatgc cgcaaaaaag ggaataaggg
cgacacggaa
5281 atgttgaata ctcatactct tcctttttca atattattga agcatttatc
agggttattg
5341 tctcatgagc ggatacatat ttgaatgtat ttagaaaaat aaacaaatag
gggttccgcg
5401 cacatttccc cgaaaagtgc cacctg
//

```
LOCUS       pLAR031                 3426 bp    DNA     circular     14-
JUN-2022
DEFINITION  attB cassette in pGSs009.
ACCESSION   urn.local...o-exuzoig
KEYWORDS    .
SOURCE
  ORGANISM  .
FEATURES             Location/Qualifiers
     attP            42..96
                     /modified_by="lrf"
                     /label="spbc"
     misc_feature    265..282
                     /Sequence="GAACGTTGCGAAGCAACG"
                     /Hairpin_Tm="83.7"
                     /Tm="58.2"
                     /Self_Dimer_Tm="40.2"
                     /%GC="55.6"
                     /created_by="primer3"
                     /label="SpBeta-P2"
                     /note="Geneious type: primer_bind_reverse"
     misc_feature    378..425
                     /label="pT-T7"
     primer_bind     397..415
                     /Sequence="CCTCTAAACGGGTCTTGAG"
                     /Hairpin_Tm="None"
                     /Tm="54.6"
                     /Self_Dimer_Tm="None"
                     /%GC="52.6"
                     /created_by="primer3"
                     /label="R4 P1"
     misc_feature    426..433
                     /label="NotI"
     misc_feature    434..496
                     /label="PhiBT1attP"
     primer_bind     495..515
                     /Sequence="CCAGGCATGTTCCCCAAAGCG"
                     /Hairpin_Tm="None"
                     /Tm="64.4"
                     /Self_Dimer_Tm="None"
                     /%GC="61.9"
                     /created_by="primer3"
                     /label="BxB-P1"
     misc_feature    497..566
                     /label="R4attP"
     primer_bind     556..575
                     /Sequence="GCGGGTGATGAGTCGTGGTT"
                     /Hairpin_Tm="None"
                     /Tm="62.8"
                     /Self_Dimer_Tm="None"
                     /%GC="60.0"
                     /created_by="primer3"
                     /label="TP901-P1"
     misc_feature    567..624
                     /label="BxB1attP"
```

```
misc_feature    625..690
                /label="TP901-1attP"
misc_feature    691..759
                /label="RVattP"
primer_bind     704..723
                /Sequence="GTATCTCACAGGTCCACGGT"
                /Hairpin_Tm="None"
                /Tm="58.8"
                /Self_Dimer_Tm="None"
                /%GC="55.0"
                /created_by="primer3"
                /label="tg1-p1"
misc_feature    734..753
                /Sequence="CTGCTGAAGAACATTCCACG"
                /Hairpin_Tm="None"
                /Tm="57.1"
                /Self_Dimer_Tm="None"
                /%GC="50.0"
                /created_by="primer3"
                /label="R4-P2"
                /note="Geneious type: primer_bind_reverse"
misc_feature    737..757
                /Sequence="CTGAAGAACATTCCACGCCAG"
                /Hairpin_Tm="None"
                /Tm="59.5"
                /Self_Dimer_Tm="None"
                /%GC="52.4"
                /created_by="primer3"
                /label="FC1-P1"
                /note="Geneious type: primer_bind_reverse"
misc_feature    760..826
                /label="TG1attP"
misc_feature    794..811
                /Sequence="GCTCTTACCCAGTTGGGC"
                /Hairpin_Tm="60.1"
                /Tm="57.7"
                /Self_Dimer_Tm="17.2"
                /%GC="61.1"
                /created_by="primer3"
                /label="bxb-p2"
                /note="Geneious type: primer_bind_reverse"
misc_feature    812..829
                /Sequence="GGGATAGCCTGCCCGATT"
                /Hairpin_Tm="34.0"
                /Tm="58.8"
                /Self_Dimer_Tm="None"
                /%GC="61.1"
                /created_by="primer3"
                /label="tp901-p2"
                /note="Geneious type: primer_bind_reverse"
primer_bind     813..831
                /Sequence="GGATAGCCTGCCCGATTAA"
                /Hairpin_Tm="None"
                /Tm="56.7"
```

```
                     /Self_Dimer_Tm="None"
                     /%GC="52.6"
                     /created_by="primer3"
                     /label="MR11-P1"
     primer_bind     814..833
                     /Sequence="GATAGCCTGCCCGATTAAAT"
                     /Hairpin_Tm="None"
                     /Tm="55.4"
                     /Self_Dimer_Tm="None"
                     /%GC="45.0"
                     /created_by="primer3"
                     /label="K38 P1"
     misc_feature    827..898
                     /label="PhiFC1attP"
     misc_feature    899..1018
                     /label="MR11attP"
     primer_bind     937..955
                     /Sequence="GCGGAACTACGAACAGTTC"
                     /Hairpin_Tm="49.1"
                     /Tm="56.4"
                     /Self_Dimer_Tm="16.0"
                     /%GC="52.6"
                     /created_by="primer3"
                     /label="370-P1"
     misc_feature    993..1012
                     /Sequence="CCACGACAATTAAGACGTGG"
                     /Hairpin_Tm="57.2"
                     /Tm="56.8"
                     /Self_Dimer_Tm="19.5"
                     /%GC="50.0"
                     /created_by="primer3"
                     /label="tg1-p2"
                     /note="Geneious type: primer_bind_reverse"
     misc_feature    1019..1070
                     /label="Phi370attP"
     primer_bind     1062..1081
                     /Sequence="GTGCCTAAACCCTAATACGC"
                     /Hairpin_Tm="None"
                     /Tm="56.3"
                     /Self_Dimer_Tm="None"
                     /%GC="50.0"
                     /created_by="primer3"
                     /label="A118-P1"
     misc_feature    1064..1084
                     /Sequence="GCCTAAACCCTAATACGCAAG"
                     /Hairpin_Tm="48.5"
                     /Tm="56.8"
                     /Self_Dimer_Tm="None"
                     /%GC="47.6"
                     /created_by="primer3"
                     /label="FC1-P2"
                     /note="Geneious type: primer_bind_reverse"
     misc_feature    1071..1126
                     /label="PhiK38attP"
```

```
primer_bind     1108..1129
                /Sequence="GACAACTTGCGCACCCTGATTG"
                /Hairpin_Tm="None"
                /Tm="62.8"
                /Self_Dimer_Tm="11.1"
                /%GC="54.5"
                /created_by="primer3"
                /label="Wbeta-P1"
primer_bind     1118..1138
                /Sequence="GCACCCTGATTGTTTAGTTCC"
                /Hairpin_Tm="None"
                /Tm="57.2"
                /Self_Dimer_Tm="None"
                /%GC="47.6"
                /created_by="primer3"
                /label="BL3-P1"
misc_feature    1127..1183
                /label="A118attP"
misc_feature    1184..1251
                /label="WBeta-attP"
misc_feature    1222..1242
                /Sequence="CACGGTACCCAATAACCAATG"
                /Hairpin_Tm="35.4"
                /Tm="57.0"
                /Self_Dimer_Tm="6.3"
                /%GC="47.6"
                /modified_by="lrf"
                /label="MR11-P2 and 370-P2"
                /note="Geneious type: primer_bind_reverse"
misc_feature    1224..1244
                /Sequence="CGGTACCCAATAACCAATGAA"
                /Hairpin_Tm="35.4"
                /Tm="55.9"
                /Self_Dimer_Tm="6.3"
                /%GC="42.9"
                /created_by="primer3"
                /label="K38 P2"
                /note="Geneious type: primer_bind_reverse"
misc_feature    1252..1312
                /label="BL3attP"
misc_feature    1313..1320
                /label="NotI"
misc_feature    1373..1395
                /Sequence="TCGTCTCTACTCCGTTACAAAGC"
                /Hairpin_Tm="None"
                /Tm="60.1"
                /Self_Dimer_Tm="None"
                /%GC="47.8"
                /created_by="primer3"
                /label="A118-P2"
                /note="Geneious type: primer_bind_reverse"
misc_feature    1413..1435
                /Sequence="GGCCTTTCTGTTATCCGAAATCC"
                /Hairpin_Tm="None"
```

```
                     /Tm="59.7"
                     /Self_Dimer_Tm="None"
                     /%GC="47.8"
                     /created_by="primer3"
                     /label="Wbeta-P2"
                     /note="Geneious type: primer_bind_reverse"
     misc_feature    1482..1503
                     /Sequence="GCTGTATGCACAAAGCATCTTC"
                     /Hairpin_Tm="62.9"
                     /Tm="58.6"
                     /Self_Dimer_Tm="1.2"
                     /%GC="45.5"
                     /created_by="primer3"
                     /label="BL3-P2"
                     /note="Geneious type: primer_bind_reverse"
     misc_feature    1596..1988
                     /label="lambda attP"
     misc_feature    1989..2425
                     /label="oriR6K"
     misc_feature    2479..3273
                     /label="Kan"
     primer_bind     3396..3416
                     /Sequence="CGCACATTTCCCCGAAAAGTG"
                     /Hairpin_Tm="46.9"
                     /Tm="60.7"
                     /Self_Dimer_Tm="14.8"
                     /%GC="52.4"
                     /created_by="primer3"
                     /label="Spbeta-P1"
ORIGIN
        1 ttttgagttc tctcagttgg gggcgtaggg tcaggcgcgc caaagtagta
agtatcttaa
       61 aaaacagata aagctgtata ttaagatact tactacatcg cgatggcccc
cgatggtagt
      121 gtggggtctc cccatgcgag agtagggaac tgccaggcat caaataaaac
gaaaggctca
      181 gtcgaaagac tgggcctttc gttttatctg ttgtttgtcg gtgaacgctc
tcctgagtag
      241 gacaaatccg ccgggagcgg atttgaacgt tgcgaagcaa cggcccggag
ggtggcgggc
      301 aggacgcccg ccataaactg ccaggcatca aattaagcag aaggccatcc
tgacggatgg
      361 cctttttgct tttataacta gcataacccc ttggggcctc taaacgggtc
ttgaggggtt
      421 ttttggcggc cgcggtgctg ggttgttgtc tctggacagt gatccatggg
aaactactca
      481 gcaccaccaa tgttccaggc atgttcccca aagcgatacc acttgaagca
gtggtactgc
      541 ttgtgggtac actctgcggg tgatgagtcg tggtttgtct ggtcaaccac
cgcggtctca
      601 gtggtgtacg gtacaaaccc cgactccaac tcgcttaatt gcgagttttt
atttcgttta
      661 tttcaattaa ggtaactaaa aaactccttt gcacaggtgt agtgtatctc
acaggtccac
```

721 ggttggccgt ggactgctga agaacattcc acgccaggat caaccccgtt ccagcccaac
781 agtgttagtc tttgctctta cccagttggg cgggatagcc tgcccgatta aatataaatt
841 ttagtacata gtgttatata cactaataaa caaaatcata tacctaaaat attacattca
901 aaataaaaaa cattgatttt tattaacttc ttttgtgcgg aactacgaac agttcattaa
961 tacgaagtgt acaaacttcc atacaaaaat aaccacgaca attaagacgt ggtttctata
1021 aaaaaataca gcgtttttca tgtacaacta tactagttgt agtgcctaaa ccctaatacg
1081 caagtcgata actctcctgg gagcgttgac aacttgcgca ccctgattgt ttagttcctc
1141 gttttctctc gttggaagaa gaagaaacga gaaactaaaa ttatagtttt aaagttggtt
1201 attagttact gtgatattta tcacggtacc caataaccaa tgaatatttg agagaatact
1261 gttgaacaat gaaaaactag gcatgtagaa gttgtttgtg cactaacttt aagcggccgc
1321 ttaatggcga tgacgcatcc tcacgataat atccgggtag gcgcaatcac tttcgtctct
1381 actccgttac aaagcgaggc tgggtatttc ccggcctttc tgttatccga aatccactga
1441 aagcacagcg gctggctgag gagataaata ataaacgagg ggctgtatgc acaaagcatc
1501 ttctgttgag ttaagaacga gtatcgagat ggcacatagc cttgctcaaa ttggaatcag
1561 gtttgtgcca ataccagtag aaacagacga agaagctagc tttgcactgg attgcgaggc
1621 tttgtgcttc tctggagtgc gacaggtttg atgacaaaaa attagcgcaa gaagacaaaa
1681 atcaccttgc gctaatgctc tgttacaggt cactaatacc atctaagtag ttgattcata
1741 gtgactgcat atatgttgtg ttttacagta ttatgtagtc tgttttttat gcaaaatcta
1801 atttaatata ttgatattta tatcatttta cgtttctcgt tcagcttttt tatactaagt
1861 tggcattata aaaaagcatt gcttatcaat ttgttgcaac gaacaggtca ctatcagtca
1921 aaataaaatc attatttgat ttcaattttg tcccactccc tgcctctgtc atcacgatac
1981 tgtgatgcca tggctaattc ccatgtcagc cgttaagtgt tcctgtgtca ctcaaaattg
2041 ctttgagagg ctctaagggc ttctcagtgc gttacatccc tggcttgttg tccacaaccg
2101 ttaaacctta aaagctttaa aagccttata tattcttttt tttcttataa aacttaaaac
2161 cttagaggct atttaagttg ctgatttata ttaattttat tgttcaaaca tgagagctta
2221 gtacgtgaaa catgagagct tagtacgtta gccatgagag cttagtacgt tagccatgag
2281 ggtttagttc gttaaacatg agagcttagt acgttaaaca tgagagctta gtacgtgaaa

2341 catgagagct tagtacgtac tatcaacagg ttgaactgct gatcttcaga
tcctctacgc
2401 cggacgcatc gtggccggat cttgcggccg caaaaattaa aaatgaagtt
ttgacggtat
2461 cgaaccccag agtcccgctc agaagaactc gtcaagaagg cgatagaagg
cgatgcgctg
2521 cgaatcggga gcggcgatac cgtaaagcac gaggaagcgg tcagcccatt
cgccgccaag
2581 ctcttcagca atatcacggg tagccaacgc tatgtcctga tagcggtccg
ccacacccag
2641 ccggccacag tcgatgaatc cagaaaagcg gccattttcc accatgatat
tcggcaagca
2701 ggcatcgcca tgggtcacga cgagatcctc gccgtcgggc atccgcgcct
tgagcctggc
2761 gaacagttcg gctggcgcga gcccctgatg ctcttcgtcc agatcatcct
gatcgacaag
2821 accggcttcc atccgagtac gtgctcgctc gatgcgatgt ttcgcttggt
ggtcgaatgg
2881 gcaggtagcc ggatcaagcg tatgcagccg ccgcattgca tcagccatga
tggatacttt
2941 ctcggcagga gcaaggtgag atgacaggag atcctgcccc ggcacttcgc
ccaatagcag
3001 ccagtccctt cccgcttcag tgacaacgtc gagcacagct gcgcaaggaa
cgcccgtcgt
3061 ggccagccac gatagccgcg ctgcctcgtc ttggagttca ttcagggcac
cggacaggtc
3121 ggtcttgaca aaaagaaccg ggcgcccctg cgctgacagc cggaacacgg
cggcatcaga
3181 gcagccgatt gtctgttgtg cccagtcata gccgaatagc ctctccaccc
aagcggccgg
3241 agaacctgcg tgcaatccat cttgttcaat catgcgaaac gatcctcatc
ctgtctcttg
3301 atccactaga ttattgaagc atttatcagg gttattgtct catgagcgga
tacatatttg
3361 aatgtattta gaaaaataaa caaatagggg ttccgcgcac atttccccga
aaagtgccac
3421 ctgcat
//

```
LOCUS       pLAR047                 5773 bp    DNA     circular UNA 12-
NOV-2019
DEFINITION  .
ACCESSION   urn.local...1k-exuztbs
VERSION     urn.local...1k-exuztbs
KEYWORDS    .
SOURCE
  ORGANISM  .
FEATURES             Location/Qualifiers
     CDS             complement(86..1036)
                     /label="repA101"
     CDS             complement(1928..2788)
                     /label="bla"
     misc_feature    2937..2958
                     /label="RP-2"
     CDS             complement(2981..3694)
                     /label="cI"
     misc_feature    3810..3949
                     /created_by="lrf"
                     /label="pEno"
     CDS             3950..5773
                     /label="PhiC31int-attB"
ORIGIN
        1 atcggatccc gggcccgtcg aatcggatcc cgggcccgtc gactgcagag
gcctgtcact
       61 gatagataca agagccataa gaacctcaga tccttccgta tttagccagt
atgttctcta
      121 gtgtggttcg ttgtttttgc gtgagccatg agaacgaacc attgagatca
tacttacttt
      181 gcatgtcact caaaaatttt gcctcaaaac tggtgagctg aatttttgca
gttaaagcat
      241 cgtgtagtgt ttttcttagt ccgttacgta ggtaggaatc tgatgtaatg
gttgttggta
      301 ttttgtcacc attcattttt atctggttgt tctcaagttc ggttacgaga
tccatttgtc
      361 tatctagttc aacttggaaa atcaacgtat cagtcgggcg gcctcgctta
tcaaccacca
      421 atttcatatt gctgtaagtg tttaaatctt tacttattgg tttcaaaacc
cattggttaa
      481 gccttttaaa ctcatggtag ttattttcaa gcattaacat gaacttaaat
tcatcaaggc
      541 taatctctat atttgccttg tgagttttct tttgtgttag ttcttttaat
aaccactcat
      601 aaatcctcat agagtatttg ttttcaaaag acttaacatg ttccagatta
tattttatga
      661 attttttttaa ctggaaaaga taaggcaata tctcttcact aaaaactaat
tctaattttt
      721 cgcttgagaa cttggcatag tttgtccact ggaaaatctc aaagccttta
accaaaggat
      781 tcctgatttc cacagttctc gtcatcagct ctctggttgc tttagctaat
acaccataag
      841 cattttccct actgatgttc atcatctgag cgtattggtt ataagtgaac
gataccgtcc
```

901 gttctttcct tgtagggttt tcaatcgtgg ggttgagtag tgccacacag cataaaatta
961 gcttggtttc atgctccgtt aagtcatagc gactaatcgc tagttcattt gctttgaaaa
1021 caactaattc agacatacat ctcaattggt ctaggtgatt ttaatcacta taccaattga
1081 gatgggctag tcaatgataa ttactagtcc ttttcctttg agttgtgggt atctgtaaat
1141 tctgctagac ctttgctgga aaacttgtaa attctgctag accctctgta aattccgcta
1201 gacctttgtg tgtttttttt gtttatattc aagtggttat aatttataga ataaagaaag
1261 aataaaaaaa gataaaaaga atagatccca gccctgtgta taactcacta ctttagtcag
1321 ttccgcagta ttacaaaagg atgtcgcaaa cgctgtttgc tcctctacaa aacagacctt
1381 aaaaccctaa aggcttaagt agcaccctcg caagctcggt tgcggccgca atcgggcaaa
1441 tcgctgaata ttccttttgt ctccgaccat caggcacctg agtcgctgtc tttttcgtga
1501 cattcagttc gctgcgctca cggctctggc agtgaatggg ggtaaatggc actacaggcg
1561 ccttttatgg attcatgcaa ggaaactacc cataatacaa gaaaagcccg tcacgggctt
1621 ctcagggcgt tttatggcgg gtctgctatg tggtgctatc tgactttttg ctgttcagca
1681 gttcctgccc tctgattttc cagtctgacc acttcggatt atcccgtgac aggtcattca
1741 gactggctaa tgcacccagt aaggcagcgg tatcatcaac ggggtctgac gctcagtgga
1801 acgaaaactc acgttaaggg attttggtca tgagattatc aaaaaggatc ttcacctaga
1861 tccttttaaa ttaaaaatga agttttaaat caatctaaag tatatatgag taaacttggt
1921 ctgacagtta ccaatgctta atcagtgagg cacctatctc agcgatctgt ctatttcgtt
1981 catccatagt tgcctgactc cccgtcgtgt agataactac gatacgggag ggcttaccat
2041 ctggccccag tgctgcaatg ataccgcgag acccacgctc accggctcca gatttatcag
2101 caataaacca gccagccgga agggccgagc gcagaagtgg tcctgcaact ttatccgcct
2161 ccatccagtc tattaattgt tgccgggaag ctagagtaag tagttcgcca gttaatagtt
2221 tgcgcaacgt tgttgccatt gctacaggca tcgtggtgtc acgctcgtcg tttggtatgg
2281 cttcattcag ctccggttcc caacgatcaa ggcgagttac atgatccccc atgttgtgca
2341 aaaaagcggt tagctccttc ggtcctccga tcgttgtcag aagtaagttg gccgcagtgt
2401 tatcactcat ggttatggca gcactgcata attctcttac tgtcatgcca tccgtaagat
2461 gcttttctgt gactggtgag tactcaacca agtcattctg agaatagtgt atgcggcgac

2521 cgagttgctc ttgcccggcg tcaatacggg ataataccgc gccacatagc agaactttaa
2581 aagtgctcat cattggaaaa cgttcttcgg ggcgaaaact ctcaaggatc ttaccgctgt
2641 tgagatccag ttcgatgtaa cccactcgtg cacccaactg atcttcagca tcttttactt
2701 tcaccagcgt ttctgggtga gcaaaaacag gaaggcaaaa tgccgcaaaa aagggaataa
2761 gggcgacacg gaaatgttga atactcatac tcttcctttt tcaatattat tgaagcattt
2821 atcagggtta ttgtctcatg agcggataca tatttgaatg tatttagaaa aataaacaaa
2881 taggggttcc gcgcacattt ccccgaaaag tgccacctgc atcgatgaag attcttgctc
2941 aattgttatc agctatgcgc cgaccagaac accttgccga tcagccaaac gtctcttcag
3001 gccactgact agcgataact ttccccacaa cggaacaact ctcattgcat gggatcattg
3061 ggtactgtgg gtttagtggt tgtaaaaaca cctgaccgct atccctgatc agtttcttga
3121 aggtaaactc atcaccccca agtctggcta tgcagaaatc acctggctca acagcctgct
3181 cagggtcaac gagaattaac attccgtcag gaaagcttgg cttggagcct gttggtgcgg
3241 tcatggaatt accttcaacc tcaagccaga atgcagaatc actggctttt ttggttgtgc
3301 ttacccatct ctccgcatca cctttggtaa aggttctaag cttaggtgag aacatccctg
3361 cctgaacatg agaaaaaaca gggtactcat actcacttct aagtgacggc tgcatactaa
3421 ccgcttcata catctcgtag atttctctgg cgattgaagg gctaaattct tcaacgctaa
3481 ctttgagaat tttgtaagc aatgcggcgt tataagcatt taatgcattg atgccattaa
3541 ataaagcacc aacgcctgac tgccccatcc ccatcttgtc tgcgacagat tcctgggata
3601 agccaagttc atttttcttt ttttcataaa ttgctttaag gcgacgtgcg tcctcaagct
3661 gctcttgtgt taatggtttc ttttttgtgc tcatacgtta aatctatcac cgcaagggat
3721 aaatatctag ttgggtaacg ccagggtttt cccagtcacg acgttgtaaa acgacggcca
3781 gtgaattgta atacgactca ctatagggcg gatccacagt ttgattacag tttagtcaga
3841 gctattgact attaaaaaac cgcttgataa aattttagct gtaagtgatg aggctataaa
3901 aaatagtata acctcatcac taaaaaatca tacaaggagg tttacctgca tggacaccta
3961 cgcgggcgcg tatgatcgtc agagccgtga acgtgaaaac agcagcgcgg cgagcccggc
4021 gacccagcgt agcgcgaacg aagacaaagc ggcggatctg caacgtgagg tggaacgtga
4081 cggtggccgt ttccgttttg tgggtcactt tagcgaggcg ccgggtacca gcgcgtttgg

4141 taccgcggaa cgtccggagt ttgaacgtat cctgaacgag tgccgtgcgg gtcgtctgaa
4201 catgatcatt gtgtacgacg ttagccgttt cagccgtctg aaggtgatgg atgcgatccc
4261 gattgttagc gaactgctgg cgctgggtgt gaccattgtt agcacccagg aaggtgtgtt
4321 tcgtcagggt aacgttatgg acctgatcca cctgattatg cgtctggatg cgagccacaa
4381 agaaagcagc ctgaaaagcg cgaagatcct ggacaccaag aacctgcaac gtgaactggg
4441 tggctacgtg ggtggcaaag cgccgtatgg cttcgagctg gttagcgaga ccaaggaaat
4501 tacccgtaac ggtcgtatgg tgaacgtggt tatcaacaaa ctggcgcaca gcaccacccc
4561 gctgaccggt ccgttcgagt ttgaaccgga tgttattcgt tggtggtggc gtgagatcaa
4621 aacccacaag cacctgccgt ttaaaccggg tagccaagcg gcgatccacc cgggcagcat
4681 taccggtctg tgcaaacgta tggatgcgga tgcggtgccg acccgtggcg aaaccattgg
4741 taagaaaacc gcgagcagcg cgtgggaccc ggcgaccgtg atgcgtattc tgcgtgatcc
4801 gcgtatcgcg ggtttcgcgg cggaagtgat ctacaagaaa aagccggacg gcaccccgac
4861 caccaaaatc gaaggttatc gtattcagcg tgacccgatc accctgcgtc cggtggagct
4921 ggattgcggt ccgatcattg agccggcgga atggtatgaa ctgcaagcgt ggctggatgg
4981 tcgtggtcgt ggtaaaggcc tgagccgtgg tcaagcgatt ctgagcgcga tggataagct
5041 gtactgcgaa tgcggcgcgg ttatgaccag caaacgtggt gaggaaagca tcaaggacag
5101 ctatcgttgc cgtcgtcgta aggtggttga tccgagcgcg ccgggtcaac atgagggtac
5161 ctgcaacgtg agcatggcgg cgctggacaa attcgttgcg gaacgtatct ttaacaagat
5221 tcgtcacgcg gagggtgatg aggaaaccct ggcgctgctg tgggaagcgg cgcgtcgttt
5281 tggcaaactg accgaagcgc cggagaagag cggtgaacgt gcgaacctgg tggcggagcg
5341 tgcggatgcg ctgaacgcgc tggaggaact gtacgaagac cgtgcggcgg gtgcgtatga
5401 tggtccggtt ggtcgtaaac acttccgtaa gcagcaagcg gcgctgaccc tgcgtcagca
5461 gggtgcggag gaacgtctgg cggagctgga agcggcggaa gcgccgaagc tgccgctgga
5521 ccagtggttt ccggaggatg cggatgcgga cccgaccggt ccgaaatcct ggtggggtcg
5581 tgcgagcgtg gacgataagc gtgtgttcgt tggcctgttt gttgataaaa tcgtggttac
5641 caaaagcacc accggtcgtg gtcagggtac cccgattgag aaacgtgcga gcattacctg
5701 ggcgaagccg ccgaccgatg atgacgaaga tgacgcgcaa gacggtaccg aggatgttgc

```
     5761 ggcgtaagct agc
//
```

```
LOCUS       pLAR052                 5386 bp    DNA     circular UNA 07-
NOV-2019
DEFINITION  .
ACCESSION   urn.local...1x-exuztbt
VERSION     urn.local...1x-exuztbt
KEYWORDS    .
SOURCE
  ORGANISM  .
FEATURES             Location/Qualifiers
     CDS             complement(86..1036)
                     /label="repA101"
     CDS             complement(1928..2788)
                     /label="bla"
     misc_feature    2937..2958
                     /label="RP-2"
     CDS             complement(2981..3694)
                     /label="cI"
     misc_feature    3810..3949
                     /created_by="lrf"
                     /label="pEno"
     misc_feature    3950..5386
                     /label="phi370"
ORIGIN
        1 atcggatccc gggcccgtcg aatcggatcc cgggcccgtc gactgcagag
gcctgtcact
       61 gatagataca agagccataa gaacctcaga tccttccgta tttagccagt
atgttctcta
      121 gtgtggttcg ttgtttttgc gtgagccatg agaacgaacc attgagatca
tacttacttt
      181 gcatgtcact caaaaatttt gcctcaaaac tggtgagctg aatttttgca
gttaaagcat
      241 cgtgtagtgt ttttcttagt ccgttacgta ggtaggaatc tgatgtaatg
gttgttggta
      301 ttttgtcacc attcattttt atctggttgt tctcaagttc ggttacgaga
tccatttgtc
      361 tatctagttc aacttggaaa atcaacgtat cagtcgggcg gcctcgctta
tcaaccacca
      421 atttcatatt gctgtaagtg tttaaatctt tacttattgg tttcaaaacc
cattggttaa
      481 gccttttaaa ctcatggtag ttattttcaa gcattaacat gaacttaaat
tcatcaaggc
      541 taatctctat atttgccttg tgagttttct tttgtgttag ttcttttaat
aaccactcat
      601 aaatcctcat agagtatttg ttttcaaaag acttaacatg ttccagatta
tattttatga
      661 atttttttaa ctggaaaaga taaggcaata tctcttcact aaaaactaat
tctaattttt
      721 cgcttgagaa cttggcatag tttgtccact ggaaaatctc aaagccttta
accaaaggat
      781 tcctgatttc cacagttctc gtcatcagct ctctggttgc tttagctaat
acaccataag
      841 cattttccct actgatgttc atcatctgag cgtattggtt ataagtgaac
gataccgtcc
```

901 gttctttcct tgtagggttt tcaatcgtgg ggttgagtag tgccacacag cataaaatta
961 gcttggtttc atgctccgtt aagtcatagc gactaatcgc tagttcattt gctttgaaaa
1021 caactaattc agacatacat ctcaattggt ctaggtgatt ttaatcacta taccaattga
1081 gatgggctag tcaatgataa ttactagtcc ttttcctttg agttgtgggt atctgtaaat
1141 tctgctagac ctttgctgga aaacttgtaa attctgctag accctctgta aattccgcta
1201 gacctttgtg tgtttttttt gtttatattc aagtggttat aatttataga ataaagaaag
1261 aataaaaaaa gataaaaaga atagatccca gccctgtgta taactcacta ctttagtcag
1321 ttccgcagta ttacaaaagg atgtcgcaaa cgctgtttgc tcctctacaa aacagacctt
1381 aaaaccctaa aggcttaagt agcaccctcg caagctcggt tgcggccgca atcgggcaaa
1441 tcgctgaata ttccttttgt ctccgaccat caggcacctg agtcgctgtc tttttcgtga
1501 cattcagttc gctgcgctca cggctctggc agtgaatggg ggtaaatggc actacaggcg
1561 ccttttatgg attcatgcaa ggaaactacc cataatacaa gaaaagcccg tcacgggctt
1621 ctcagggcgt tttatggcgg gtctgctatg tggtgctatc tgactttttg ctgttcagca
1681 gttcctgccc tctgattttc cagtctgacc acttcggatt atcccgtgac aggtcattca
1741 gactggctaa tgcacccagt aaggcagcgg tatcatcaac ggggtctgac gctcagtgga
1801 acgaaaactc acgttaaggg attttggtca tgagattatc aaaaaggatc ttcacctaga
1861 tccttttaaa ttaaaaatga agttttaaat caatctaaag tatatatgag taaacttggt
1921 ctgacagtta ccaatgctta atcagtgagg cacctatctc agcgatctgt ctatttcgtt
1981 catccatagt tgcctgactc cccgtcgtgt agataactac gatacgggag ggcttaccat
2041 ctggccccag tgctgcaatg ataccgcgag acccacgctc accggctcca gatttatcag
2101 caataaacca gccagccgga agggccgagc gcagaagtgg tcctgcaact ttatccgcct
2161 ccatccagtc tattaattgt tgccgggaag ctagagtaag tagttcgcca gttaatagtt
2221 tgcgcaacgt tgttgccatt gctacaggca tcgtggtgtc acgctcgtcg tttggtatgg
2281 cttcattcag ctccggttcc caacgatcaa ggcgagttac atgatccccc atgttgtgca
2341 aaaaagcggt tagctccttc ggtcctccga tcgttgtcag aagtaagttg gccgcagtgt
2401 tatcactcat ggttatggca gcactgcata attctcttac tgtcatgcca tccgtaagat
2461 gcttttctgt gactggtgag tactcaacca agtcattctg agaatagtgt atgcggcgac

2521 cgagttgctc ttgcccggcg tcaatacggg ataataccgc gccacatagc agaactttaa
2581 aagtgctcat cattggaaaa cgttcttcgg ggcgaaaact ctcaaggatc ttaccgctgt
2641 tgagatccag ttcgatgtaa cccactcgtg cacccaactg atcttcagca tcttttactt
2701 tcaccagcgt ttctgggtga gcaaaaacag gaaggcaaaa tgccgcaaaa aagggaataa
2761 gggcgacacg gaaatgttga atactcatac tcttcctttt tcaatattat tgaagcattt
2821 atcagggtta ttgtctcatg agcggataca tatttgaatg tatttagaaa aataaacaaa
2881 taggggttcc gcgcacattt ccccgaaaag tgccacctgc atcgatgaag attcttgctc
2941 aattgttatc agctatgcgc cgaccagaac accttgccga tcagccaaac gtctcttcag
3001 gccactgact agcgataact ttccccacaa cggaacaact ctcattgcat gggatcattg
3061 ggtactgtgg gtttagtggt tgtaaaaaca cctgaccgct atccctgatc agtttcttga
3121 aggtaaactc atcaccccca agtctggcta tgcagaaatc acctggctca acagcctgct
3181 cagggtcaac gagaattaac attccgtcag gaaagcttgg cttggagcct gttggtgcgg
3241 tcatggaatt accttcaacc tcaagccaga atgcagaatc actggctttt ttggttgtgc
3301 ttacccatct ctccgcatca cctttggtaa aggttctaag cttaggtgag aacatccctg
3361 cctgaacatg agaaaaaaca gggtactcat actcacttct aagtgacggc tgcatactaa
3421 ccgcttcata catctcgtag atttctctgg cgattgaagg gctaaattct tcaacgctaa
3481 ctttgagaat ttttgtaagc aatgcggcgt tataagcatt taatgcattg atgccattaa
3541 ataaagcacc aacgcctgac tgccccatcc ccatcttgtc tgcgacagat tcctgggata
3601 agccaagttc atttttcttt ttttcataaa ttgctttaag gcgacgtgcg tcctcaagct
3661 gctcttgtgt taatggtttc ttttttgtgc tcatacgtta aatctatcac cgcaagggat
3721 aaatatctag ttgggtaacg ccagggtttt cccagtcacg acgttgtaaa acgacggcca
3781 gtgaattgta atacgactca ctatagggcg gatccacagt ttgattacag tttagtcaga
3841 gctattgact attaaaaaac cgcttgataa aattttagct gtaagtgatg aggctataaa
3901 aaatagtata acctcatcac taaaaaatca tacaaggagg tttacctgca tgcgtaaggt
3961 ggcgatctac agccgtgtta gcaccattaa ccaggcggag gaaggttata gcatccaggg
4021 ccaaattgaa gcgctgacca agtactgcga agcgatggag tggaagatct ataaaaacta
4081 cagcgacgcg ggtttcagcg gtggcaaact ggagcgtccg gcgatcaccg aactgattga

4141 ggacggcaag aacaacaaat ttgataccat cctggtgtat aagctggacc gtctgagccg
4201 taacgttaag gacaccctgt acctggtgaa agatgttttc accgcgaaca acattcactt
4261 tgtgagcctg aaagaaaaca tcgataccag cagcgcgatg ggtaacctgt tcctgaccct
4321 gctgagcgcg attgcggagt ttgaacgtga gcagatcaag gagcgtatgc aattcggtgt
4381 tatgaaccgt gcgaagagcg gcaaaaccac cgcgtggaaa accccgccgt acggctatcg
4441 ttacaacaag gacgaaaaaa ccctgagcgt gaacgaactg gaggcggcga acgttcgtca
4501 gatgttcgac atgatcatta gcggttgcag catcatgagc attaccaact atgcgcgtga
4561 taactttgtg ggcaacacct ggacccacgt gaaggttaaa cgtatcctgg aaaacgagac
4621 ctataagggt ctggttaaat accgtgaaca gaccttcagc ggcgaccacc aagcgatcat
4681 tgatgagaag acctacaaca aagcgcagat tgcgctggcg caccgtaccg acaccaagac
4741 caacacccgt ccgtttcagg gtaagtatat gctgagccac atcgcgaaat gcggttattg
4801 cggtgcgccg ctgaaggtgt gcaccggtcg tgcgaaaaac gatggcaccc gtcgtcagac
4861 ctatgtgtgc gttaacaaaa ccgaaagcct ggcgcgtcgt agcgttaaca actacaacaa
4921 ccaaaagatc tgcaacaccg gccgttacga aaagaaacac atcgagaaat atgtgattga
4981 cgttctgtac aagctgcaac acgataagga gtacctgaag aaaatcaaga aagacgataa
5041 catcatcgac atcaccccgc tgaagaaaga aatcgagatc atcgacaaga aaatcaaccg
5101 tctgaacgat ctgtacatca acgacctgat tgatctgccg aagctgaaga aagatattga
5161 ggaactgaac cacctgaaag acgattataa caaggcgatc aaactgaact acctggacaa
5221 gaaaaacgaa gatagcctgg gtatgctgat ggacaacctg gatattcgta agagcagcta
5281 cgacgtgcag agccgtatcg ttaaacaact gattgatcgt gttgaggtga ccatggacaa
5341 catcgatatc attttcaaat tttaagctag ctgtaaagga gactga
//

```
LOCUS       pLAR053                 5258 bp    DNA     circular UNA 14-
JUN-2022
DEFINITION  .
ACCESSION   urn.local...1y-exuztbt
VERSION     urn.local...1y-exuztbt
KEYWORDS    .
SOURCE
  ORGANISM  .
FEATURES             Location/Qualifiers
     misc_feature    1..1359
                     /label="A118"
     CDS             complement(1395..2345)
                     /label="repA101"
     CDS             complement(3237..4097)
                     /label="bla"
     misc_feature    4246..4267
                     /label="RP-2"
     CDS             complement(4290..5003)
                     /label="cI"
     misc_feature    5119..5258
                     /created_by="lrf"
                     /label="pEno"
ORIGIN
        1 atgaaggcgg cgatctatat tcgtgttagc acccaggagc aagtggaaaa
ctacagcatc
       61 caggcgcaaa ccgagaagct gaccgcgctg tgccgtagca aagactggga
tgtttatgat
      121 atcttcattg acggtggcta cagcggtagc aacatgaacc gtccggcgct
gaacgagatg
      181 ctgagcaagc tgcacgaaat tgatgcggtg gttgtgtatc gtctggaccg
tctgagccgt
      241 agccagcgtg ataccatcac cctgattgag gaatacttcc tgaaaaacaa
cgttgagttt
      301 gtgagcctga gcgaaaccct ggacaccagc agcccgttcg gtcgtgcgat
gatcggcatt
      361 ctgagcgttt ttgcgcaact ggagcgtgaa accatccgtg atcgtatggt
gatgggtaaa
      421 atcaagcgta ttgaggcggg tctgccgctg accaccgcga aggtcgtac
cttcggctat
      481 gacgtgatcg ataccaagct gtacattaac gaggaagagg cgaaacagct
gcaactgatc
      541 tacgacattt tcgaagagga acagagcatc acctttctgc aaaagcgtct
gaagaaactg
      601 ggttttaaag ttcgtaccta caaccgttat aacaactggc tgaccaacga
cctgtactgc
      661 ggttatgtga gctacaaaga taaggttcac gtgaagggca tccacgagcc
gatcattagc
      721 gaggaacagt tctaccgtgt tcaagaaatt tttacccgta tgggcaaaaa
cccgaacatg
      781 aaccgtgaca gcgcgagcct gctgaacaac ctggttgtgt gcagcaagtg
cggtctgggc
      841 tttgtgcacc gtcgtaaaga taccatgagc cgtggtaaga aataccacta
tcgttactat
```

901 agctgcaaaa cctataagca cacccacgag ctggaaaaat gcggcaacaa gatctggcgt
961 gcggacaagc tggaggaact gatcattaac cgtgttaaca actacagctt cgcgagccgt
1021 aacgtggaca aagaggatga actggacagc ctgaacgaga aactgaagat cgaacacgcg
1081 aagaaaaagc gtctgtttga cctgtatatt aacggtagct acgaagttag cgaactggat
1141 agcatgatga acgacatcga tgcgcagatt aactactatg agagccaaat cgaagcgaac
1201 gaggaactga aaaagaacaa aaagattcag gagaacctgg cggacctggc gaccgttgac
1261 ttcgatagcc tggagtttcg tgaaaaacag ctgtatctga agagcctgat caacaaaatc
1321 tacattgatg gcgagcaagt gaccattgaa tggctgtaag cctgtcactg atagatacaa
1381 gagccataag aacctcagat ccttccgtat ttagccagta tgttctctag tgtggttcgt
1441 tgtttttgcg tgagccatga gaacgaacca ttgagatcat acttactttg catgtcactc
1501 aaaaattttg cctcaaaact ggtgagctga atttttgcag ttaaagcatc gtgtagtgtt
1561 tttcttagtc cgttacgtag gtaggaatct gatgtaatgg ttgttggtat tttgtcacca
1621 ttcattttta tctggttgtt ctcaagttcg gttacgagat ccatttgtct atctagttca
1681 acttggaaaa tcaacgtatc agtcgggcgg cctcgcttat caaccaccaa tttcatattg
1741 ctgtaagtgt ttaaatcttt acttattggt ttcaaaaccc attggttaag ccttttaaac
1801 tcatggtagt tattttcaag cattaacatg aacttaaatt catcaaggct aatctctata
1861 tttgccttgt gagttttctt ttgtgttagt tcttttaata accactcata aatcctcata
1921 gagtatttgt tttcaaaaga cttaacatgt tccagattat attttatgaa ttttttttaac
1981 tggaaaagat aaggcaatat ctcttcacta aaaactaatt ctaatttttc gcttgagaac
2041 ttggcatagt ttgtccactg gaaaatctca aagcctttaa ccaaaggatt cctgatttcc
2101 acagttctcg tcatcagctc tctggttgct ttagctaata caccataagc attttcccta
2161 ctgatgttca tcatctgagc gtattggtta taagtgaacg ataccgtccg ttctttcctt
2221 gtagggtttt caatcgtggg gttgagtagt gccacacagc ataaaattag cttggtttca
2281 tgctccgtta agtcatagcg actaatcgct agttcatttg ctttgaaaac aactaattca
2341 gacatacatc tcaattggtc taggtgattt taatcactat accaattgag atgggctagt
2401 caatgataat tactagtcct tttcctttga gttgtgggta tctgtaaatt ctgctagacc
2461 tttgctggaa aacttgtaaa ttctgctaga ccctctgtaa attccgctag acctttgtgt

2521 gtttttttg tttatattca agtggttata atttatagaa taaagaaaga ataaaaaaag
2581 ataaaaagaa tagatcccag ccctgtgtat aactcactac tttagtcagt tccgcagtat
2641 tacaaaagga tgtcgcaaac gctgtttgct cctctacaaa acagacctta aaaccctaaa
2701 ggcttaagta gcaccctcgc aagctcggtt gcggccgcaa tcgggcaaat cgctgaatat
2761 tccttttgtc tccgaccatc aggcacctga gtcgctgtct ttttcgtgac attcagttcg
2821 ctgcgctcac ggctctggca gtgaatgggg gtaaatggca ctacaggcgc cttttatgga
2881 ttcatgcaag gaaactaccc ataatacaag aaaagcccgt cacgggcttc tcagggcgtt
2941 ttatggcggg tctgctatgt ggtgctatct gactttttgc tgttcagcag ttcctgccct
3001 ctgattttcc agtctgacca cttcggatta tcccgtgaca ggtcattcag actggctaat
3061 gcacccagta aggcagcggt atcatcaacg gggtctgacg ctcagtggaa cgaaaactca
3121 cgttaaggga ttttggtcat gagattatca aaaaggatct tcacctagat ccttttaaat
3181 taaaaatgaa gttttaaatc aatctaaagt atatatgagt aaacttggtc tgacagttac
3241 caatgcttaa tcagtgaggc acctatctca gcgatctgtc tatttcgttc atccatagtt
3301 gcctgactcc ccgtcgtgta gataactacg atacgggagg gcttaccatc tggccccagt
3361 gctgcaatga taccgcgaga cccacgctca ccggctccag atttatcagc aataaaccag
3421 ccagccggaa gggccgagcg cagaagtggt cctgcaactt tatccgcctc catccagtct
3481 attaattgtt gccgggaagc tagagtaagt agttcgccag ttaatagttt gcgcaacgtt
3541 gttgccattg ctacaggcat cgtggtgtca cgctcgtcgt ttggtatggc ttcattcagc
3601 tccggttccc aacgatcaag gcgagttaca tgatccccca tgttgtgcaa aaaagcggtt
3661 agctccttcg gtcctccgat cgttgtcaga agtaagttgg ccgcagtgtt atcactcatg
3721 gttatggcag cactgcataa ttctcttact gtcatgccat ccgtaagatg cttttctgtg
3781 actggtgagt actcaaccaa gtcattctga gaatagtgta tgcggcgacc gagttgctct
3841 tgcccggcgt caatacggga taataccgcg ccacatagca gaactttaaa agtgctcatc
3901 attggaaaac gttcttcggg gcgaaaactc tcaaggatct taccgctgtt gagatccagt
3961 tcgatgtaac ccactcgtgc acccaactga tcttcagcat cttttacttt caccagcgtt
4021 tctgggtgag caaaaacagg aaggcaaaat gccgcaaaaa agggaataag ggcgacacgg
4081 aaatgttgaa tactcatact cttccttttt caatattatt gaagcattta tcagggttat

4141 tgtctcatga gcggatacat atttgaatgt atttagaaaa ataaacaaat aggggttccg
4201 cgcacatttc cccgaaaagt gccacctgca tcgatgaaga ttcttgctca attgttatca
4261 gctatgcgcc gaccagaaca ccttgccgat cagccaaacg tctcttcagg ccactgacta
4321 gcgataactt tccccacaac ggaacaactc tcattgcatg ggatcattgg gtactgtggg
4381 tttagtggtt gtaaaaacac ctgaccgcta tccctgatca gtttcttgaa ggtaaactca
4441 tcacccccaa gtctggctat gcagaaatca cctggctcaa cagcctgctc agggtcaacg
4501 agaattaaca ttccgtcagg aaagcttggc ttggagcctg ttggtgcggt catggaatta
4561 ccttcaacct caagccagaa tgcagaatca ctggcttttt tggttgtgct tacccatctc
4621 tccgcatcac ctttggtaaa ggttctaagc ttaggtgaga acatccctgc ctgaacatga
4681 gaaaaaacag ggtactcata ctcacttcta agtgacggct gcatactaac cgcttcatac
4741 atctcgtaga tttctctggc gattgaaggg ctaaattctt caacgctaac tttgagaatt
4801 tttgtaagca atgcggcgtt ataagcattt aatgcattga tgccattaaa taaagcacca
4861 acgcctgact gccccatccc catcttgtct gcgacagatt cctgggataa gccaagttca
4921 tttttctttt tttcataaat tgctttaagg cgacgtgcgt cctcaagctg ctcttgtgtt
4981 aatggtttct tttttgtgct catacgttaa atctatcacc gcaagggata aatatctagt
5041 tgggtaacgc cagggttttc ccagtcacga cgttgtaaaa cgacggccag tgaattgtaa
5101 tacgactcac tatagggcgg atccacagtt tgattacagt ttagtcagag ctattgacta
5161 ttaaaaaacc gcttgataaa attttagctg taagtgatga ggctataaaa aatagtataa
5221 cctcatcact aaaaaatcat acaaggaggt ttacctgc
//

```
LOCUS       pLAR055                 5344 bp    DNA     circular UNA 11-
OCT-2017
DEFINITION  .
ACCESSION   urn.local...20-exuztbt
VERSION     urn.local...20-exuztbt
KEYWORDS    .
SOURCE
  ORGANISM  .
FEATURES             Location/Qualifiers
     CDS             complement(86..1036)
                     /label="repA101"
     CDS             complement(1928..2788)
                     /label="bla"
     misc_feature    2937..2958
                     /label="RP-2"
     CDS             complement(2981..3694)
                     /label="cI"
     misc_feature    3950..5344
                     /label="FC1"
ORIGIN
        1 atcggatccc gggcccgtcg aatcggatcc cgggcccgtc gactgcagag
gcctgtcact
       61 gatagataca agagccataa gaacctcaga tccttccgta tttagccagt
atgttctcta
      121 gtgtggttcg ttgtttttgc gtgagccatg agaacgaacc attgagatca
tacttacttt
      181 gcatgtcact caaaaatttt gcctcaaaac tggtgagctg aatttttgca
gttaaagcat
      241 cgtgtagtgt ttttcttagt ccgttacgta ggtaggaatc tgatgtaatg
gttgttggta
      301 ttttgtcacc attcattttt atctggttgt tctcaagttc ggttacgaga
tccatttgtc
      361 tatctagttc aacttggaaa atcaacgtat cagtcgggcg gcctcgctta
tcaaccacca
      421 atttcatatt gctgtaagtg tttaaatctt tacttattgg tttcaaaacc
cattggttaa
      481 gccttttaaa ctcatggtag ttattttcaa gcattaacat gaacttaaat
tcatcaaggc
      541 taatctctat atttgccttg tgagttttct tttgtgttag ttcttttaat
aaccactcat
      601 aaatcctcat agagtatttg ttttcaaaag acttaacatg ttccagatta
tattttatga
      661 atttttttaa ctggaaaaga taaggcaata tctcttcact aaaaactaat
tctaattttt
      721 cgcttgagaa cttggcatag tttgtccact ggaaaatctc aaagccttta
accaaaggat
      781 tcctgatttc cacagttctc gtcatcagct ctctggttgc tttagctaat
acaccataag
      841 cattttccct actgatgttc atcatctgag cgtattggtt ataagtgaac
gataccgtcc
      901 gttctttcct tgtagggttt tcaatcgtgg ggttgagtag tgccacacag
cataaaatta
      961 gcttggtttc atgctccgtt aagtcatagc gactaatcgc tagttcattt
gctttgaaaa
```

1021 caactaattc agacatacat ctcaattggt ctaggtgatt ttaatcacta
taccaattga
1081 gatgggctag tcaatgataa ttactagtcc ttttcctttg agttgtgggt
atctgtaaat
1141 tctgctagac ctttgctgga aaacttgtaa attctgctag accctctgta
aattccgcta
1201 gacctttgtg tgtttttttt gtttatattc aagtggttat aatttataga
ataaagaaag
1261 aataaaaaaa gataaaaaga atagatccca gccctgtgta taactcacta
ctttagtcag
1321 ttccgcagta ttacaaaagg atgtcgcaaa cgctgtttgc tcctctacaa
aacagacctt
1381 aaaaccctaa aggcttaagt agcaccctcg caagctcggt tgcggccgca
atcgggcaaa
1441 tcgctgaata ttccttttgt ctccgaccat caggcacctg agtcgctgtc
tttttcgtga
1501 cattcagttc gctgcgctca cggctctggc agtgaatggg ggtaaatggc
actacaggcg
1561 ccttttatgg attcatgcaa ggaaactacc cataatacaa gaaaagcccg
tcacgggctt
1621 ctcagggcgt tttatggcgg gtctgctatg tggtgctatc tgactttttg
ctgttcagca
1681 gttcctgccc tctgattttc cagtctgacc acttcggatt atcccgtgac
aggtcattca
1741 gactggctaa tgcacccagt aaggcagcgg tatcatcaac ggggtctgac
gctcagtgga
1801 acgaaaactc acgttaaggg attttggtca tgagattatc aaaaaggatc
ttcacctaga
1861 tccttttaaa ttaaaaatga agttttaaat caatctaaag tatatatgag
taaacttggt
1921 ctgacagtta ccaatgctta atcagtgagg cacctatctc agcgatctgt
ctatttcgtt
1981 catccatagt tgcctgactc cccgtcgtgt agataactac gatacgggag
ggcttaccat
2041 ctggccccag tgctgcaatg ataccgcgag acccacgctc accggctcca
gatttatcag
2101 caataaacca gccagccgga agggccgagc gcagaagtgg tcctgcaact
ttatccgcct
2161 ccatccagtc tattaattgt tgccgggaag ctagagtaag tagttcgcca
gttaatagtt
2221 tgcgcaacgt tgttgccatt gctacaggca tcgtggtgtc acgctcgtcg
tttggtatgg
2281 cttcattcag ctccggttcc caacgatcaa ggcgagttac atgatccccc
atgttgtgca
2341 aaaaagcggt tagctccttc ggtcctccga tcgttgtcag aagtaagttg
gccgcagtgt
2401 tatcactcat ggttatggca gcactgcata attctcttac tgtcatgcca
tccgtaagat
2461 gcttttctgt gactggtgag tactcaacca agtcattctg agaatagtgt
atgcggcgac
2521 cgagttgctc ttgcccggcg tcaatacggg ataataccgc gccacatagc
agaactttaa
2581 aagtgctcat cattggaaaa cgttcttcgg ggcgaaaact ctcaaggatc
ttaccgctgt

2641 tgagatccag ttcgatgtaa cccactcgtg cacccaactg atcttcagca tcttttactt
2701 tcaccagcgt ttctgggtga gcaaaaacag gaaggcaaaa tgccgcaaaa aagggaataa
2761 gggcgacacg gaaatgttga atactcatac tcttcctttt tcaatattat tgaagcattt
2821 atcagggtta ttgtctcatg agcggataca tatttgaatg tatttagaaa aataaacaaa
2881 taggggttcc gcgcacattt ccccgaaaag tgccacctgc atcgatgaag attcttgctc
2941 aattgttatc agctatgcgc cgaccagaac accttgccga tcagccaaac gtctcttcag
3001 gccactgact agcgataact ttccccacaa cggaacaact ctcattgcat gggatcattg
3061 ggtactgtgg gtttagtggt tgtaaaaaca cctgaccgct atccctgatc agtttcttga
3121 aggtaaactc atcaccccca agtctggcta tgcagaaatc acctggctca acagcctgct
3181 cagggtcaac gagaattaac attccgtcag gaaagcttgg cttggagcct gttggtgcgg
3241 tcatggaatt accttcaacc tcaagccaga atgcagaatc actggctttt ttggttgtgc
3301 ttacccatct ctccgcatca cctttggtaa aggttctaag cttaggtgag aacatccctg
3361 cctgaacatg agaaaaaaca gggtactcat actcacttct aagtgacggc tgcatactaa
3421 ccgcttcata catctcgtag atttctctgg cgattgaagg gctaaattct tcaacgctaa
3481 ctttgagaat ttttgtaagc aatgcggcgt tataagcatt taatgcattg atgccattaa
3541 ataaagcacc aacgcctgac tgccccatcc ccatcttgtc tgcgacagat tcctgggata
3601 agccaagttc atttttcttt ttttcataaa ttgctttaag gcgacgtgcg tcctcaagct
3661 gctcttgtgt taatggtttc ttttttgtgc tcatacgtta aatctatcac cgcaagggat
3721 aaatatctag ttgggtaacg ccagggtttt cccagtcacg acgttgtaaa acgacggcca
3781 gtgaattgta atacgactca ctatagggcg gatccacagt ttgattacag tttagtcaga
3841 gctattgact attaaaaaac cgcttgataa aattttagct gtaagtgatg aggctataaa
3901 aaatagtata acctcatcac taaaaaatca tacaaggagg tttacctgca tgaaacgtgc
3961 ggcgctgtac atccgtgtga gcaccatgga acaggcgaaa gagggttata gcattccggc
4021 gcaaaccgac aagctgaaag cgttcgcgaa ggcgaaagac atggcggtgg cgaaagttta
4081 caccgatccg ggttttagcg gtgcgaagat ggaacgtccg gcgctgcaag agatgatcag
4141 cgacattcaa aacaagaaaa tcgatgtggt tctggtgtac aaactggatc gtctgagccg
4201 tagccagaag aacaccctgt atctgatcga agacgtgttc ctgaagaaca acgttgattt

4261 catcagcatg caggagagct tcgacaccag caccccgttt ggccgtgcga
ccatcggtat
4321 gctgagcgtt ttcgcgcagc tggaacgtga taccattacc gagcgtatgc
acatgggccg
4381 taccgaacgt gcgaagcaag gttactatca cggtagcggc atcgtgccgc
tgggctacga
4441 ctatgttcac ggcgagctga tcattaacga ttacgaagcg cagatcattc
aagagatcta
4501 cgacctgtat gtgaaccagg gtaaaggcca gcaatacatt accaaacgta
tggttgcgaa
4561 gtatccggat aaggtgaaaa ccctgaccat cgttaagtac gcgctgacca
acccgctgta
4621 tatcggtaaa attagctggg acggcaaggt gtacgatggt caccacagcc
cgatcattga
4681 caaaagcatg tatgataagg cgcaggaaat cattgcgcgt atggcgcaaa
aaggtggcga
4741 gcagcacggt aaccaactgg gcctgctgct gggtatcacc tactgcggca
agtgcggcgc
4801 ggaagtgttt cgttatgtta gcggtggcaa gaaataccgt tataactact
atatgtgccg
4861 tagcgtgaag aaaatgctgc cgagcctggt taaagactgg aactgcaagc
agccgagcct
4921 gcgtcaagag gtggttgaaa agaaagtgat cgacagcctg aaaagcctgg
atttcaagaa
4981 aattgagcgt gaactgaagc aggttgaaaa caagaccaaa agcaagatca
ccaccattaa
5041 caaccaaatc agcaagaaac acaacgagaa acagaagatt ctggacctgt
accaatatgg
5101 cacctttgat gttaccatgc tgaacgaacg tatgaagaaa atcgacaacg
agattaacgc
5161 gctgaccgcg aacatcgcga acctggaagg taccaaaagc gagagcctga
ttaacaaact
5221 ggaaaccctg aagaccttca actgggagac cgaaaccacc gagaacaaaa
tcctgatcat
5281 taaggagttc gtggaacgta ttgagctgtt tgacgatgag gttatcatta
aatacaagtt
5341 ttaa
//

```
LOCUS       pLAR056                 5523 bp    DNA     circular UNA 14-
JUN-2022
DEFINITION  .
ACCESSION   urn.local...21-exuztbt
VERSION     urn.local...21-exuztbt
KEYWORDS    .
SOURCE
  ORGANISM  .
FEATURES             Location/Qualifiers
     CDS             complement(1..951)
                     /label="repA101"
     CDS             complement(1843..2703)
                     /label="bla"
     misc_feature    2852..2873
                     /label="RP-2"
     CDS             complement(2896..3609)
                     /label="cI"
     misc_feature    3865..5523
                     /label="phik38"
ORIGIN
        1 tcagatcctt ccgtatttag ccagtatgtt ctctagtgtg gttcgttgtt
tttgcgtgag
       61 ccatgagaac gaaccattga gatcatactt actttgcatg tcactcaaaa
attttgcctc
      121 aaaactggtg agctgaattt ttgcagttaa agcatcgtgt agtgtttttc
ttagtccgtt
      181 acgtaggtag gaatctgatg taatggttgt tggtattttg tcaccattca
tttttatctg
      241 gttgttctca agttcggtta cgagatccat ttgtctatct agttcaactt
ggaaaatcaa
      301 cgtatcagtc gggcggcctc gcttatcaac caccaatttc atattgctgt
aagtgtttaa
      361 atctttactt attggtttca aaacccattg gttaagcctt ttaaactcat
ggtagttatt
      421 ttcaagcatt aacatgaact taaattcatc aaggctaatc tctatatttg
ccttgtgagt
      481 tttcttttgt gttagttctt ttaataacca ctcataaatc ctcatagagt
atttgttttc
      541 aaaagactta acatgttcca gattatattt tatgaatttt tttaactgga
aaagataagg
      601 caatatctct tcactaaaaa ctaattctaa tttttcgctt gagaacttgg
catagtttgt
      661 ccactggaaa atctcaaagc ctttaaccaa aggattcctg atttccacag
ttctcgtcat
      721 cagctctctg gttgctttag ctaatacacc ataagcattt tccctactga
tgttcatcat
      781 ctgagcgtat tggttataag tgaacgatac cgtccgttct ttccttgtag
ggttttcaat
      841 cgtggggttg agtagtgcca cacagcataa aattagcttg gtttcatgct
ccgttaagtc
      901 atagcgacta atcgctagtt catttgcttt gaaaacaact aattcagaca
tacatctcaa
      961 ttggtctagg tgattttaat cactatacca attgagatgg gctagtcaat
gataattact
```

1021 agtccttttc ctttgagttg tgggtatctg taaattctgc tagacctttg ctggaaaact
1081 tgtaaattct gctagaccct ctgtaaattc cgctagacct ttgtgtgttt tttttgttta
1141 tattcaagtg gttataattt atagaataaa gaaagaataa aaaaagataa aaagaataga
1201 tcccagccct gtgtataact cactacttta gtcagttccg cagtattaca aaaggatgtc
1261 gcaaacgctg tttgctcctc tacaaaacag accttaaaac cctaaaggct taagtagcac
1321 cctcgcaagc tcggttgcgg ccgcaatcgg gcaaatcgct gaatattcct tttgtctccg
1381 accatcaggc acctgagtcg ctgtcttttt cgtgacattc agttcgctgc gctcacggct
1441 ctggcagtga atgggggtaa atggcactac aggcgccttt tatggattca tgcaaggaaa
1501 ctacccataa tacaagaaaa gcccgtcacg ggcttctcag ggcgttttat ggcgggtctg
1561 ctatgtggtg ctatctgact ttttgctgtt cagcagttcc tgccctctga ttttccagtc
1621 tgaccacttc ggattatccc gtgacaggtc attcagactg gctaatgcac ccagtaaggc
1681 agcggtatca tcaacggggt ctgacgctca gtggaacgaa aactcacgtt aagggatttt
1741 ggtcatgaga ttatcaaaaa ggatcttcac ctagatcctt ttaaattaaa aatgaagttt
1801 taaatcaatc taaagtatat atgagtaaac ttggtctgac agttaccaat gcttaatcag
1861 tgaggcacct atctcagcga tctgtctatt tcgttcatcc atagttgcct gactccccgt
1921 cgtgtagata actacgatac gggagggctt accatctggc cccagtgctg caatgatacc
1981 gcgagaccca cgctcaccgg ctccagattt atcagcaata aaccagccag ccggaagggc
2041 cgagcgcaga agtggtcctg caactttatc cgcctccatc cagtctatta attgttgccg
2101 ggaagctaga gtaagtagtt cgccagttaa tagtttgcgc aacgttgttg ccattgctac
2161 aggcatcgtg gtgtcacgct cgtcgtttgg tatggcttca ttcagctccg gttcccaacg
2221 atcaaggcga gttacatgat cccccatgtt gtgcaaaaaa gcggttagct ccttcggtcc
2281 tccgatcgtt gtcagaagta agttggccgc agtgttatca ctcatggtta tggcagcact
2341 gcataattct cttactgtca tgccatccgt aagatgcttt tctgtgactg gtgagtactc
2401 aaccaagtca ttctgagaat agtgtatgcg gcgaccgagt tgctcttgcc cggcgtcaat
2461 acgggataat accgcgccac atagcagaac tttaaaagtg ctcatcattg gaaaacgttc
2521 ttcggggcga aaactctcaa ggatcttacc gctgttgaga tccagttcga tgtaacccac
2581 tcgtgcaccc aactgatctt cagcatcttt tactttcacc agcgtttctg ggtgagcaaa

2641 aacaggaagg caaaatgccg caaaaaaggg aataagggcg acacggaaat
gttgaatact
2701 catactcttc ctttttcaat attattgaag catttatcag ggttattgtc
tcatgagcgg
2761 atacatattt gaatgtattt agaaaaataa acaaataggg gttccgcgca
catttccccg
2821 aaaagtgcca cctgcatcga tgaagattct tgctcaattg ttatcagcta
tgcgccgacc
2881 agaacacctt gccgatcagc caaacgtctc ttcaggccac tgactagcga
taactttccc
2941 cacaacggaa caactctcat tgcatgggat cattgggtac tgtgggttta
gtggttgtaa
3001 aaacacctga ccgctatccc tgatcagttt cttgaaggta aactcatcac
ccccaagtct
3061 ggctatgcag aaatcacctg gctcaacagc ctgctcaggg tcaacgagaa
ttaacattcc
3121 gtcaggaaag cttggcttgg agcctgttgg tgcggtcatg gaattacctt
caacctcaag
3181 ccagaatgca gaatcactgg ctttttggt tgtgcttacc catctctccg
catcaccttt
3241 ggtaaaggtt ctaagcttag gtgagaacat ccctgcctga acatgagaaa
aaacagggta
3301 ctcatactca cttctaagtg acggctgcat actaaccgct tcatacatct
cgtagatttc
3361 tctggcgatt gaagggctaa attcttcaac gctaactttg agaatttttg
taagcaatgc
3421 ggcgttataa gcatttaatg cattgatgcc attaaataaa gcaccaacgc
ctgactgccc
3481 catccccatc ttgtctgcga cagattcctg ggataagcca agttcatttt
tcttttttc
3541 ataaattgct ttaaggcgac gtgcgtcctc aagctgctct tgtgttaatg
gtttcttttt
3601 tgtgctcata cgttaaatct atcaccgcaa gggataaata tctagttggg
taacgccagg
3661 gttttcccag tcacgacgtt gtaaaacgac ggccagtgaa ttgtaatacg
actcactata
3721 gggcggatcc acagtttgat tacagtttag tcagagctat tgactattaa
aaaaccgctt
3781 gataaaattt tagctgtaag tgatgaggct ataaaaaata gtataacctc
atcactaaaa
3841 aatcatacaa ggaggtttac ctgcatgccg ggtatgacca ccgagaccgg
tccggacccg
3901 gcgggcctga ttgacctgtt ctgccgtaag agcaaagcgg ttaaaagccg
tgcgaacggt
3961 gcgggtcagc gtcgtaaaca agagatcagc attgcggcgc aagaaaccct
gggtcgtaaa
4021 gtggcggcgc tgctgggtat gcaagtgcgt cacgtttgga aagaagttgg
tagcgcgagc
4081 cgttttcgta agggcaaagc gcgtgacgat caaagcaagg cgctgaaagc
gctggagagc
4141 ggtgaagtgg gtgcgctgtg gtgctaccgt ctggaccgtt gggatcgtgg
tggcgcgggt
4201 gcgatcctga agatcattga gccggaagac ggcatgccgc gtcgtctgct
gttcggttgg

4261 gacgaggata ccggccgtcc ggttctggac agcaccaaca aacgtgatcg
tggtgaactg
4321 attcgtcgtg cggaggaagc gcgtgaggaa gcggagaagc tgagcgaacg
tgtgcgtgac
4381 accaaagcgc accagcgtga gaacggtgaa tgggttaacg cgcgtgcgcc
gtatggtctg
4441 cgtgtggttc tggtgaccgt tagcgatgag gaaggtgacg agtatgatga
gcgtaagctg
4501 gcggcggacg atgaagatgc gggtggcccg gatggtctga ccaaagcgga
agcggcgcgt
4561 ctggtgttca ccctgccggt taccgaccgt ctgagctatg cgggtaccgc
gcacgcgatg
4621 aacacccgtg agattccgag cccgaccggt ggcccgtgga ttgcggtgac
cgttcgtgac
4681 atgattcaaa acccggcgta tgcgggttgg cagaccaccg gtcgtcagga
tggcaagcaa
4741 cgtcgtctga ccttttataa cggtgaaggc aaacgtgtga gcgttatgca
tggcccgccg
4801 ctggtgaccg atgaggaaca agaggcggcg aaagcggcgg ttaaaggtga
agacggtgtg
4861 ggcgttccgc tggatggcag cgaccacgat acccgtcgta agcacctgct
gagcggtcgt
4921 atgcgttgcc cgggttgcgg tggtagctgt agctacagcg gtaacggcta
tcgttgctgg
4981 cgtagcagcg tgaagggtgg ctgcccggcg ccgacctatg ttgcgcgtaa
aagcgtggag
5041 gaatatgttg cgtttcgttg ggcggcgaaa ctggcggcga gcgagccgga
cgatccgttt
5101 gtgattgcgg ttgcggaccg ttgggcggcg ctgacccacc cgcaagcgag
cgaggatgaa
5161 aagtacgcga aagcggcggt gcgtgaggcg gaaaagaacc tgggtcgtct
gctgcgcgac
5221 cgtcagaacg gtgtttacga tggtccggcg gagcagttct ttgcgccggc
gtatcaagaa
5281 gcgctgagca ccctgcaagc ggcgaaagat gctgtgagcg aaagcagcgc
gagcgcggcg
5341 gtggatgtta gctggatcgt tgacagcagc gattatgagg aactgtggct
gcgtgcgacc
5401 ccgaccatgc gtaacgcgat cattgacacc tgcatcgatg agatttgggt
ggcgaagggt
5461 cagcgtggtc gtccgtttga cggcgatgaa cgtgttaaga ttaaatgggc
ggcgcgtacc
5521 taa
//

```
LOCUS       pLAR057                 5326 bp    DNA     circular UNA 06-
NOV-2017
DEFINITION  .
ACCESSION   urn.local...22-exuztbt
VERSION     urn.local...22-exuztbt
KEYWORDS    .
SOURCE
  ORGANISM  .
FEATURES             Location/Qualifiers
     misc_feature    45..65
                     /label="Reverse Seq"
     CDS             complement(86..1036)
                     /label="repA101"
     CDS             complement(1928..2788)
                     /label="bla"
     misc_feature    2937..2958
                     /label="RP-2"
     CDS             complement(2981..3694)
                     /label="cI"
     misc_feature    3691..3713
                     /label="forward seq"
     misc_feature    3950..5326
                     /label="MRII"
ORIGIN
        1 atcggatccc gggcccgtcg aatcggatcc cgggcccgtc gactgcagag
gcctgtcact
       61 gatagataca agagccataa gaacctcaga tccttccgta tttagccagt
atgttctcta
      121 gtgtggttcg ttgtttttgc gtgagccatg agaacgaacc attgagatca
tacttacttt
      181 gcatgtcact caaaaatttt gcctcaaaac tggtgagctg aatttttgca
gttaaagcat
      241 cgtgtagtgt ttttcttagt ccgttacgta ggtaggaatc tgatgtaatg
gttgttggta
      301 ttttgtcacc attcattttt atctggttgt tctcaagttc ggttacgaga
tccatttgtc
      361 tatctagttc aacttggaaa atcaacgtat cagtcgggcg gcctcgctta
tcaaccacca
      421 atttcatatt gctgtaagtg tttaaatctt tacttattgg tttcaaaacc
cattggttaa
      481 gccttttaaa ctcatggtag ttattttcaa gcattaacat gaacttaaat
tcatcaaggc
      541 taatctctat atttgccttg tgagttttct tttgtgttag ttcttttaat
aaccactcat
      601 aaatcctcat agagtatttg ttttcaaaag acttaacatg ttccagatta
tattttatga
      661 atttttttaa ctggaaaaga taaggcaata tctcttcact aaaaactaat
tctaattttt
      721 cgcttgagaa cttggcatag tttgtccact ggaaaatctc aaagccttta
accaaaggat
      781 tcctgatttc cacagttctc gtcatcagct ctctggttgc tttagctaat
acaccataag
      841 cattttccct actgatgttc atcatctgag cgtattggtt ataagtgaac
gataccgtcc
```

901 gttctttcct tgtagggttt tcaatcgtgg ggttgagtag tgccacacag cataaaatta
961 gcttggtttc atgctccgtt aagtcatagc gactaatcgc tagttcattt gctttgaaaa
1021 caactaattc agacatacat ctcaattggt ctaggtgatt ttaatcacta taccaattga
1081 gatgggctag tcaatgataa ttactagtcc ttttcctttg agttgtgggt atctgtaaat
1141 tctgctagac ctttgctgga aaacttgtaa attctgctag accctctgta aattccgcta
1201 gacctttgtg tgtttttttt gtttatattc aagtggttat aatttataga ataaagaaag
1261 aataaaaaaa gataaaaaga atagatccca gccctgtgta taactcacta ctttagtcag
1321 ttccgcagta ttacaaaagg atgtcgcaaa cgctgtttgc tcctctacaa aacagacctt
1381 aaaaccctaa aggcttaagt agcaccctcg caagctcggt tgcggccgca atcgggcaaa
1441 tcgctgaata ttccttttgt ctccgaccat caggcacctg agtcgctgtc tttttcgtga
1501 cattcagttc gctgcgctca cggctctggc agtgaatggg ggtaaatggc actacaggcg
1561 ccttttatgg attcatgcaa ggaaactacc cataatacaa gaaaagcccg tcacgggctt
1621 ctcagggcgt tttatggcgg gtctgctatg tggtgctatc tgactttttg ctgttcagca
1681 gttcctgccc tctgattttc cagtctgacc acttcggatt atcccgtgac aggtcattca
1741 gactggctaa tgcacccagt aaggcagcgg tatcatcaac ggggtctgac gctcagtgga
1801 acgaaaactc acgttaaggg attttggtca tgagattatc aaaaaggatc ttcacctaga
1861 tccttttaaa ttaaaaatga agttttaaat caatctaaag tatatatgag taaacttggt
1921 ctgacagtta ccaatgctta atcagtgagg cacctatctc agcgatctgt ctatttcgtt
1981 catccatagt tgcctgactc cccgtcgtgt agataactac gatacgggag ggcttaccat
2041 ctggccccag tgctgcaatg ataccgcgag acccacgctc accggctcca gatttatcag
2101 caataaacca gccagccgga agggccgagc gcagaagtgg tcctgcaact ttatccgcct
2161 ccatccagtc tattaattgt tgccgggaag ctagagtaag tagttcgcca gttaatagtt
2221 tgcgcaacgt tgttgccatt gctacaggca tcgtggtgtc acgctcgtcg tttggtatgg
2281 cttcattcag ctccggttcc caacgatcaa ggcgagttac atgatccccc atgttgtgca
2341 aaaaagcggt tagctccttc ggtcctccga tcgttgtcag aagtaagttg gccgcagtgt
2401 tatcactcat ggttatggca gcactgcata attctcttac tgtcatgcca tccgtaagat
2461 gcttttctgt gactggtgag tactcaacca agtcattctg agaatagtgt atgcggcgac

2521 cgagttgctc ttgcccggcg tcaatacggg ataataccgc gccacatagc agaactttaa
2581 aagtgctcat cattggaaaa cgttcttcgg ggcgaaaact ctcaaggatc ttaccgctgt
2641 tgagatccag ttcgatgtaa cccactcgtg cacccaactg atcttcagca tcttttactt
2701 tcaccagcgt ttctgggtga gcaaaaacag gaaggcaaaa tgccgcaaaa aagggaataa
2761 gggcgacacg gaaatgttga atactcatac tcttcctttt tcaatattat tgaagcattt
2821 atcagggtta ttgtctcatg agcggataca tatttgaatg tatttagaaa aataaacaaa
2881 taggggttcc gcgcacattt ccccgaaaag tgccacctgc atcgatgaag attcttgctc
2941 aattgttatc agctatgcgc cgaccagaac accttgccga tcagccaaac gtctcttcag
3001 gccactgact agcgataact ttccccacaa cggaacaact ctcattgcat gggatcattg
3061 ggtactgtgg gtttagtggt tgtaaaaaca cctgaccgct atccctgatc agtttcttga
3121 aggtaaactc atcacccccca agtctggcta tgcagaaatc acctggctca acagcctgct
3181 cagggtcaac gagaattaac attccgtcag gaaagcttgg cttggagcct gttggtgcgg
3241 tcatggaatt accttcaacc tcaagccaga atgcagaatc actggctttt ttggttgtgc
3301 ttacccatct ctccgcatca cctttggtaa aggttctaag cttaggtgag aacatccctg
3361 cctgaacatg agaaaaaaca gggtactcat actcacttct aagtgacggc tgcatactaa
3421 ccgcttcata catctcgtag atttctctgg cgattgaagg gctaaattct tcaacgctaa
3481 ctttgagaat ttttgtaagc aatgcggcgt tataagcatt taatgcattg atgccattaa
3541 ataaagcacc aacgcctgac tgccccatcc ccatcttgtc tgcgacagat tcctgggata
3601 agccaagttc atttttcttt ttttcataaa ttgctttaag gcgacgtgcg tcctcaagct
3661 gctcttgtgt taatggtttc ttttttgtgc tcatacgtta aatctatcac cgcaagggat
3721 aaatatctag ttgggtaacg ccagggtttt cccagtcacg acgttgtaaa acgacggcca
3781 gtgaattgta atacgactca ctatagggcg gatccacagt ttgattacag tttagtcaga
3841 gctattgact attaaaaaac cgcttgataa aattttagct gtaagtgatg aggctataaa
3901 aaatagtata acctcatcac taaaaaatca tacaaggagg tttacctgca tgaaggttgc
3961 gatctacacc cgtgtgagca ccctggagca gaaggaaaaa ggtcacagca ttgaggaaca
4021 agagcgtaaa ctgcgtgcgt atagcgacat caacgattgg accattcagg gcgtttacgt
4081 ggacgcgggt tatagcggcg cgaagaccga tcgtccggag ctgaaccgtc tgaaggaaaa

4141 cctgagcaaa atcgacctgg ttctggtgta caagctggat cgtctgaccc gtaacgttaa
4201 agacctgctg gatctgctgg agattttcga acgtgagaac gttagctttc gtagcgcgac
4261 cgaagtgtat gacaccagca ccgcgatggg tcgtctgttc gttaccctgg tgggcgcgat
4321 ggcggaatgg gagcgtgaaa ccatccgtga gcgtgcgatg atgggcaagc aggcggcgat
4381 ccgtaaaggc atgattctga ccccgccgcc gttttactat gaccgtgttg ataacaagta
4441 catcccgaac aagtacaaag atgtggttgt gtgggcgtac gaggaagtga agaaaggtaa
4501 cagcgcgaag ggcatcgcgc gtaaactgaa cgcgagcgac atcccgccgc cgaacggtat
4561 tcaatgggaa gatcgtacca ttacccgtgc gctgcgtagc ccgctgagca agggtcacta
4621 cttctggggc gacatcttta ttgagaacag ccacgaaccg atcattaccg atgagatgta
4681 taacgagatc aaggaacgtc tgaacgaacg tgttaacgcg aaaaccatca cccacaccag
4741 cgtgttccgt ggtaaactga tttgcccgaa ctgcaacggc cgtctgtgcc tgaacaccag
4801 ctaccgtaag ctgaaacgtg gtgacgttat ccacaagaac tactattgca acaactgcaa
4861 ggtgaacaaa agcggcgcgt tcagctttac cgagaaggaa gcgctgaaag tgttctacga
4921 ctatctgagc aagctggatc tgagcaagta taaagcgaag gagaaagaag acaagaaaat
4981 cgttaccatc gatatcaaca aggtgatgga acagcgtaag cgttaccaca aactgtatgc
5041 gaacggtatg atgcaagagg aagagctgtt cgagctgatc aaggagaccg acgaaaaaat
5101 tagcgagtac gaaaagcaaa aagaacgtgt tccgaagaaa cgtctggacg tgagcaagat
5161 caaaaacttt aagaacattc tgctggatag ctggaacgcg ttcaccctgg aggacaaaga
5221 agatttcatc aagatggcga tcaagagcat cgagattgaa tacatccacg tgaaacgtgg
5281 caagaccaag cacagcatca agatcaagaa catcgatttc tattaa
//

```
LOCUS       pLAR058                 5359 bp    DNA     circular UNA 12-
OCT-2017
DEFINITION  .
ACCESSION   urn.local...23-exuztbt
VERSION     urn.local...23-exuztbt
KEYWORDS    .
SOURCE
  ORGANISM  .
FEATURES             Location/Qualifiers
     CDS             complement(86..1036)
                     /label="repA101"
     CDS             complement(1928..2788)
                     /label="bla"
     misc_feature    2937..2958
                     /label="RP-2"
     CDS             complement(2981..3694)
                     /label="cI"
     misc_feature    3950..5359
                     /label="R4"
ORIGIN
        1 atcggatccc gggcccgtcg aatcggatcc cgggcccgtc gactgcagag
gcctgtcact
       61 gatagataca agagccataa gaacctcaga tccttccgta tttagccagt
atgttctcta
      121 gtgtggttcg ttgtttttgc gtgagccatg agaacgaacc attgagatca
tacttacttt
      181 gcatgtcact caaaaatttt gcctcaaaac tggtgagctg aatttttgca
gttaaagcat
      241 cgtgtagtgt ttttcttagt ccgttacgta ggtaggaatc tgatgtaatg
gttgttggta
      301 ttttgtcacc attcattttt atctggttgt tctcaagttc ggttacgaga
tccatttgtc
      361 tatctagttc aacttggaaa atcaacgtat cagtcgggcg gcctcgctta
tcaaccacca
      421 atttcatatt gctgtaagtg tttaaatctt tacttattgg tttcaaaacc
cattggttaa
      481 gccttttaaa ctcatggtag ttattttcaa gcattaacat gaacttaaat
tcatcaaggc
      541 taatctctat atttgccttg tgagttttct tttgtgttag ttcttttaat
aaccactcat
      601 aaatcctcat agagtatttg ttttcaaaag acttaacatg ttccagatta
tattttatga
      661 atttttttaa ctggaaaaga taaggcaata tctcttcact aaaaactaat
tctaattttt
      721 cgcttgagaa cttggcatag tttgtccact ggaaaatctc aaagccttta
accaaaggat
      781 tcctgatttc cacagttctc gtcatcagct ctctggttgc tttagctaat
acaccataag
      841 cattttccct actgatgttc atcatctgag cgtattggtt ataagtgaac
gataccgtcc
      901 gttctttcct tgtagggttt tcaatcgtgg ggttgagtag tgccacacag
cataaaatta
      961 gcttggtttc atgctccgtt aagtcatagc gactaatcgc tagttcattt
gctttgaaaa
```

1021 caactaattc agacatacat ctcaattggt ctaggtgatt ttaatcacta taccaattga
1081 gatgggctag tcaatgataa ttactagtcc ttttcctttg agttgtgggt atctgtaaat
1141 tctgctagac ctttgctgga aaacttgtaa attctgctag accctctgta aattccgcta
1201 gacctttgtg tgtttttttt gtttatattc aagtggttat aatttataga ataaagaaag
1261 aataaaaaaa gataaaaaga atagatccca gccctgtgta taactcacta ctttagtcag
1321 ttccgcagta ttacaaaagg atgtcgcaaa cgctgtttgc tcctctacaa aacagacctt
1381 aaaaccctaa aggcttaagt agcaccctcg caagctcggt tgcggccgca atcgggcaaa
1441 tcgctgaata ttccttttgt ctccgaccat caggcacctg agtcgctgtc tttttcgtga
1501 cattcagttc gctgcgctca cggctctggc agtgaatggg ggtaaatggc actacaggcg
1561 ccttttatgg attcatgcaa ggaaactacc cataatacaa gaaaagcccg tcacgggctt
1621 ctcagggcgt tttatggcgg gtctgctatg tggtgctatc tgactttttg ctgttcagca
1681 gttcctgccc tctgattttc cagtctgacc acttcggatt atcccgtgac aggtcattca
1741 gactggctaa tgcacccagt aaggcagcgg tatcatcaac ggggtctgac gctcagtgga
1801 acgaaaactc acgttaaggg attttggtca tgagattatc aaaaaggatc ttcacctaga
1861 tccttttaaa ttaaaaatga agttttaaat caatctaaag tatatatgag taaacttggt
1921 ctgacagtta ccaatgctta atcagtgagg cacctatctc agcgatctgt ctatttcgtt
1981 catccatagt tgcctgactc cccgtcgtgt agataactac gatacgggag ggcttaccat
2041 ctggccccag tgctgcaatg ataccgcgag acccacgctc accggctcca gatttatcag
2101 caataaacca gccagccgga agggccgagc gcagaagtgg tcctgcaact ttatccgcct
2161 ccatccagtc tattaattgt tgccgggaag ctagagtaag tagttcgcca gttaatagtt
2221 tgcgcaacgt tgttgccatt gctacaggca tcgtggtgtc acgctcgtcg tttggtatgg
2281 cttcattcag ctccggttcc caacgatcaa ggcgagttac atgatccccc atgttgtgca
2341 aaaaagcggt tagctccttc ggtcctccga tcgttgtcag aagtaagttg gccgcagtgt
2401 tatcactcat ggttatggca gcactgcata attctcttac tgtcatgcca tccgtaagat
2461 gcttttctgt gactggtgag tactcaacca agtcattctg agaatagtgt atgcggcgac
2521 cgagttgctc ttgcccggcg tcaatacggg ataataccgc gccacatagc agaactttaa
2581 aagtgctcat cattggaaaa cgttcttcgg ggcgaaaact ctcaaggatc ttaccgctgt

2641 tgagatccag ttcgatgtaa cccactcgtg cacccaactg atcttcagca tcttttactt
2701 tcaccagcgt ttctgggtga gcaaaaacag gaaggcaaaa tgccgcaaaa aagggaataa
2761 gggcgacacg gaaatgttga atactcatac tcttcctttt tcaatattat tgaagcattt
2821 atcagggtta ttgtctcatg agcggataca tatttgaatg tatttagaaa aataaacaaa
2881 taggggttcc gcgcacattt ccccgaaaag tgccacctgc atcgatgaag attcttgctc
2941 aattgttatc agctatgcgc cgaccagaac accttgccga tcagccaaac gtctcttcag
3001 gccactgact agcgataact ttccccacaa cggaacaact ctcattgcat gggatcattg
3061 ggtactgtgg gtttagtggt tgtaaaaaca cctgaccgct atccctgatc agtttcttga
3121 aggtaaactc atcaccccca agtctggcta tgcagaaatc acctggctca acagcctgct
3181 cagggtcaac gagaattaac attccgtcag gaaagcttgg cttggagcct gttggtgcgg
3241 tcatggaatt accttcaacc tcaagccaga atgcagaatc actggctttt ttggttgtgc
3301 ttacccatct ctccgcatca cctttggtaa aggttctaag cttaggtgag aacatccctg
3361 cctgaacatg agaaaaaaca gggtactcat actcacttct aagtgacggc tgcatactaa
3421 ccgcttcata catctcgtag atttctctgg cgattgaagg gctaaattct tcaacgctaa
3481 ctttgagaat ttttgtaagc aatgcggcgt tataagcatt taatgcattg atgccattaa
3541 ataaagcacc aacgcctgac tgccccatcc ccatcttgtc tgcgacagat tcctgggata
3601 agccaagttc atttttcttt ttttcataaa ttgctttaag gcgacgtgcg tcctcaagct
3661 gctcttgtgt taatggtttc ttttttgtgc tcatacgtta aatctatcac cgcaagggat
3721 aaatatctag ttgggtaacg ccagggtttt cccagtcacg acgttgtaaa acgacggcca
3781 gtgaattgta atacgactca ctatagggcg gatccacagt ttgattacag tttagtcaga
3841 gctattgact attaaaaaac cgcttgataa aattttagct gtaagtgatg aggctataaa
3901 aaatagtata acctcatcac taaaaaatca tacaaggagg tttacctgca tgaaccgtgg
3961 tggcccgacc gtgcgtgcgg acatctacgt tcgtattagc ctggatcgta ccggcgagga
4021 actgggcgtg gagcgtcagg aagagagctg ccgtgaactg tgcaagagcc tgggtatgga
4081 agtgggccaa gtgtgggttg acaacgatct gagcgcgacc aagaaaaacg tggttcgtcc
4141 ggacttcgaa gcgatgatcg cgagcaaccc gcaggcgatt gtgtgctggc acaccgatcg
4201 tctgatccgt gtgacccgtg acctggagcg tgttattgat ctgggcgtga acgttcacgc

4261 ggttatggcg ggtcacctgg acctgagcac cccggcgggc cgtgcggtgg cgcgtaccgt
4321 taccgcgtgg gcgacctacg agggtgaaca gaaggcggaa cgtcaaaaac tggcgaacat
4381 ccaaaacgcg cgtgcgggca agccgtacac cccgggtatc cgtccgtttg gttatggcga
4441 cgatcacatg accattgtga ccgcggaggc ggatgcgatc cgtgatggtg cgaaaatgat
4501 tctggatggt tggagcctga gcgcggttgc gcgttattgg gaggaactga aactgcaaag
4561 cccgcgtagc atggcggcgg gtggcaaagg ttggagcctg cgtggcgtga agaaagttct
4621 gaccagcccg cgttacgtgg gtcgtagcag ctatctgggt gaagtggttg gcgacgcgca
4681 atggccgcca atcctggacc cggatgttta ctatggcgtg gttgcgattc tgaacaaccc
4741 ggatcgtttc agcggtggcc cgcgtaccgg tcgtaccccg ggtaccctgc tggcgggtat
4801 tgcgctgtgc ggcgagtgcg gcaagaccgt gagcggtcgt ggctaccgtg gtgtgctggt
4861 ttatggctgc aaagacaccc acacccgtac cccgcgtagc attgcggatg gtcgtgcgag
4921 cagcagcacc ctggcgcgtc tgatgttccc ggactttctg ccgggtctgc tggcgagcgg
4981 tcaggcggaa gatggtcaaa gcgcggcgag caagcacagc gaggcgcaga ccctgcgtga
5041 acgtctggat ggcctggcga ccgcgtatgc ggaaggtgcg atcagcctga gccaaatgac
5101 cgcgggtagc gaggcgctgc gtaagaaact ggaagtgatc gaagcggacc tggttggtag
5161 cgcgggcatt ccgccgtttg atccggtggc gggtgttgcg ggcctgatta gcggttggcc
5221 gaccaccccg ctgccgaccc gtcgtgcgtg ggtggacttt tgcctggtgg ttaccctgaa
5281 cacccagaaa ggccgtcacg cgagcagcat gaccgtggac gatcacgtta ccatcgagtg
5341 gcgtgatgtt gcggaataa
//

```
LOCUS       pLAR060                 5809 bp    DNA     circular UNA 12-
OCT-2017
DEFINITION  .
ACCESSION   urn.local...25-exuztbt
VERSION     urn.local...25-exuztbt
KEYWORDS    .
SOURCE
  ORGANISM  .
FEATURES             Location/Qualifiers
     CDS             complement(86..1036)
                     /label="repA101"
     CDS             complement(1928..2788)
                     /label="bla"
     misc_feature    2937..2958
                     /label="RP-2"
     CDS             complement(2981..3694)
                     /label="cI"
     misc_feature    3950..5809
                     /label="TG1"
ORIGIN
        1 atcggatccc gggcccgtcg aatcggatcc cgggcccgtc gactgcagag
gcctgtcact
       61 gatagataca agagccataa gaacctcaga tccttccgta tttagccagt
atgttctcta
      121 gtgtggttcg ttgtttttgc gtgagccatg agaacgaacc attgagatca
tacttacttt
      181 gcatgtcact caaaaatttt gcctcaaaac tggtgagctg aatttttgca
gttaaagcat
      241 cgtgtagtgt ttttcttagt ccgttacgta ggtaggaatc tgatgtaatg
gttgttggta
      301 ttttgtcacc attcattttt atctggttgt tctcaagttc ggttacgaga
tccatttgtc
      361 tatctagttc aacttggaaa atcaacgtat cagtcgggcg gcctcgctta
tcaaccacca
      421 atttcatatt gctgtaagtg tttaaatctt tacttattgg tttcaaaacc
cattggttaa
      481 gccttttaaa ctcatggtag ttattttcaa gcattaacat gaacttaaat
tcatcaaggc
      541 taatctctat atttgccttg tgagttttct tttgtgttag ttcttttaat
aaccactcat
      601 aaatcctcat agagtatttg ttttcaaaag acttaacatg ttccagatta
tattttatga
      661 atttttttaa ctggaaaaga taaggcaata tctcttcact aaaaactaat
tctaattttt
      721 cgcttgagaa cttggcatag tttgtccact ggaaaatctc aaagccttta
accaaaggat
      781 tcctgatttc cacagttctc gtcatcagct ctctggttgc tttagctaat
acaccataag
      841 cattttccct actgatgttc atcatctgag cgtattggtt ataagtgaac
gataccgtcc
      901 gttctttcct tgtagggttt tcaatcgtgg ggttgagtag tgccacacag
cataaaatta
      961 gcttggtttc atgctccgtt aagtcatagc gactaatcgc tagttcattt
gctttgaaaa
```

1021 caactaattc agacatacat ctcaattggt ctaggtgatt ttaatcacta taccaattga
1081 gatgggctag tcaatgataa ttactagtcc ttttcctttg agttgtgggt atctgtaaat
1141 tctgctagac ctttgctgga aaacttgtaa attctgctag accctctgta aattccgcta
1201 gacctttgtg tgtttttttt gtttatattc aagtggttat aatttataga ataaagaaag
1261 aataaaaaaa gataaaaaga atagatccca gccctgtgta taactcacta ctttagtcag
1321 ttccgcagta ttacaaaagg atgtcgcaaa cgctgtttgc tcctctacaa aacagacctt
1381 aaaaccctaa aggcttaagt agcaccctcg caagctcggt tgcggccgca atcgggcaaa
1441 tcgctgaata ttcctttgt ctccgaccat caggcacctg agtcgctgtc tttttcgtga
1501 cattcagttc gctgcgctca cggctctggc agtgaatggg ggtaaatggc actacaggcg
1561 ccttttatgg attcatgcaa ggaaactacc cataatacaa gaaaagcccg tcacgggctt
1621 ctcagggcgt tttatggcgg gtctgctatg tggtgctatc tgactttttg ctgttcagca
1681 gttcctgccc tctgattttc cagtctgacc acttcggatt atcccgtgac aggtcattca
1741 gactggctaa tgcacccagt aaggcagcgg tatcatcaac ggggtctgac gctcagtgga
1801 acgaaaactc acgttaaggg attttggtca tgagattatc aaaaaggatc ttcacctaga
1861 tccttttaaa ttaaaaatga agttttaaat caatctaaag tatatatgag taaacttggt
1921 ctgacagtta ccaatgctta atcagtgagg cacctatctc agcgatctgt ctatttcgtt
1981 catccatagt tgcctgactc cccgtcgtgt agataactac gatacgggag ggcttaccat
2041 ctggccccag tgctgcaatg ataccgcgag acccacgctc accggctcca gatttatcag
2101 caataaacca gccagccgga agggccgagc gcagaagtgg tcctgcaact ttatccgcct
2161 ccatccagtc tattaattgt tgccgggaag ctagagtaag tagttcgcca gttaatagtt
2221 tgcgcaacgt tgttgccatt gctacaggca tcgtggtgtc acgctcgtcg tttggtatgg
2281 cttcattcag ctccggttcc caacgatcaa ggcgagttac atgatccccc atgttgtgca
2341 aaaaagcggt tagctccttc ggtcctccga tcgttgtcag aagtaagttg gccgcagtgt
2401 tatcactcat ggttatggca gcactgcata attctcttac tgtcatgcca tccgtaagat
2461 gcttttctgt gactggtgag tactcaacca agtcattctg agaatagtgt atgcggcgac
2521 cgagttgctc ttgcccggcg tcaatacggg ataataccgc gccacatagc agaactttaa
2581 aagtgctcat cattggaaaa cgttcttcgg ggcgaaaact ctcaaggatc ttaccgctgt

2641 tgagatccag ttcgatgtaa cccactcgtg cacccaactg atcttcagca
tcttttactt
2701 tcaccagcgt ttctgggtga gcaaaaacag gaaggcaaaa tgccgcaaaa
aagggaataa
2761 gggcgacacg gaaatgttga atactcatac tcttcctttt tcaatattat
tgaagcattt
2821 atcagggtta ttgtctcatg agcggataca tatttgaatg tatttagaaa
aataaacaaa
2881 taggggttcc gcgcacattt ccccgaaaag tgccacctgc atcgatgaag
attcttgctc
2941 aattgttatc agctatgcgc cgaccagaac accttgccga tcagccaaac
gtctcttcag
3001 gccactgact agcgataact ttccccacaa cggaacaact ctcattgcat
gggatcattg
3061 ggtactgtgg gtttagtggt tgtaaaaaca cctgaccgct atccctgatc
agtttcttga
3121 aggtaaactc atcaccccca agtctggcta tgcagaaatc acctggctca
acagcctgct
3181 cagggtcaac gagaattaac attccgtcag gaaagcttgg cttggagcct
gttggtgcgg
3241 tcatggaatt accttcaacc tcaagccaga atgcagaatc actggctttt
ttggttgtgc
3301 ttacccatct ctccgcatca cctttggtaa aggttctaag cttaggtgag
aacatccctg
3361 cctgaacatg agaaaaaaca gggtactcat actcacttct aagtgacggc
tgcatactaa
3421 ccgcttcata catctcgtag atttctctgg cgattgaagg gctaaattct
tcaacgctaa
3481 ctttgagaat ttttgtaagc aatgcggcgt tataagcatt taatgcattg
atgccattaa
3541 ataaagcacc aacgcctgac tgccccatcc ccatcttgtc tgcgacagat
tcctgggata
3601 agccaagttc atttttcttt ttttcataaa ttgctttaag gcgacgtgcg
tcctcaagct
3661 gctcttgtgt taatggtttc ttttttgtgc tcatacgtta aatctatcac
cgcaagggat
3721 aaatatctag ttgggtaacg ccagggtttt cccagtcacg acgttgtaaa
acgacggcca
3781 gtgaattgta atacgactca ctatagggcg gatccacagt ttgattacag
tttagtcaga
3841 gctattgact attaaaaaac cgcttgataa aattttagct gtaagtgatg
aggctataaa
3901 aaatagtata acctcatcac taaaaaatca tacaaggagg tttacctgca
tggtgattct
3961 ggcgggtggc tacgaccgtc agagcgcgga gcgtgaaaac agcagcaccg
cgagcccggc
4021 gacccaacgt gcggcgaacc gtggcaaggc ggaagcgctg gcgaaagagt
atgcgcgtga
4081 tggtgtggaa gttaaatggc tgggtcactt cagcgaggcg ccgggtacca
gcgcgttcac
4141 cggcgtggac cgtccggaat ttaaccgtat tctggatatg tgccgtaacc
gtgagatgaa
4201 catgatcatt gttcactaca tcagccgtct gagccgtgag gaaccgctgg
acatcattcc

4261 ggtggttacc gaactgctgc gtctgggtgt gaccattgtg agcgttaacg aaggtacctt
4321 ccgtccgggc gagatgatgg acctgatcca cctgattatg cgtctgcaag cgagccacga
4381 tgaaagcaag aacaaaagcg tggcggttag caacgcgaag gagctggcga aacgtctggg
4441 tggccacacc ggtagcaccc cgtacggctt tgacaccgtg gaggaaatgg ttccgaaccc
4501 ggaggatggt ggcaaactgg tggcgatccg tcgtctggtt ccgagcgcgc acacctggga
4561 aggtgcgcat ggtagcgagg gtgcggttat tcgttgggcg tggcaggaaa tcaagaccca
4621 ccgtgacacc ccgttcaaag gtggcggtgc gggtagcttt catccgggta gcctgaacgg
4681 tctgtgcgag cgtctgtatc gtgataaggt gccgacccgt ggtaccctgg ttggcaagaa
4741 acgtgcgggt agcgactggg acccgggtgt gctgaaacgt gttctgagcg acccgcgtat
4801 tgcgggttac caagcggaca tcgcgtataa agtgcgtgcg gatggcagcc gtggcggttt
4861 cagccactac aaaattcgtc gtgatccggt taccatggaa ccgctgaccc tgccgggttt
4921 tgagccgtat atcccgccgg cggagtggtg ggaactgcaa gagtggctgc aaggccgtgg
4981 tcgtggcaag ggccagtacc gtggccaaag cctgctgagc gcgatggacg tgctgtactg
5041 ctatggcagc ggtcagctgg acccggaaac cggttacagc aacggcagca ccatggcggg
5101 taacgttcgt gagggcgacc aagcgcacaa gagcagctat gcgtgcaaat gcccgcgtcg
5161 tgtgcacgac ggtagcagct gtagcattac catgcacaac ctggacccgt acatcgttgg
5221 cgcgattttc gcgcgtatta ccgcgtttga cccggcggac ccggatgatc tggaaggtga
5281 taccgcggcg ctgatgtatg aagcggcgcg tcgttggggt gcgacccacg aacgtccgga
5341 gctgaaaggc cagcgtagcg aactgatggc gcaacgtgcg gacgcggtta aggcgctgga
5401 ggaactgtac gaggataaac gtaacggcgg ttatcgtagc gcgatgggtc gtcgtgcgtt
5461 cctggaggaa gaggcggcgc tgaccctgcg tatggagggt gcggaagagc gtctgcgtca
5521 actggatgcg gcggatagcc cggtgctgcc gattggcgag tggctgggtg accgtggcag
5581 cgatccgacc ggtccgggtt cctggtgggc gctggcgccg ctggaagacc gtcgtgcgtt
5641 cgtgcgtctg tttgttgatc gtatcgaggt tattaagctg ccgaaaggtg tgcaacgtcc
5701 gggtcgtgtt ccgccgattg cggaccgtgt gcgtatccac tgggcgaagc cgaaagttga
5761 agaggaaacc gagccggaaa ccctgaacgg ttttaccgcg gcggcgtaa
//

```
LOCUS       pLAR061                  5322 bp    DNA     circular UNA 14-
JUN-2022
DEFINITION  .
ACCESSION   urn.local...26-exuztbt
VERSION     urn.local...26-exuztbt
KEYWORDS    .
SOURCE
  ORGANISM  .
FEATURES             Location/Qualifiers
     CDS             complement(1..951)
                     /label="repA101"
     CDS             complement(1843..2703)
                     /label="bla"
     misc_feature    2852..2873
                     /label="RP-2"
     CDS             complement(2896..3609)
                     /label="cI"
     misc_feature    3865..5322
                     /label="TP901"
ORIGIN
        1 tcagatcctt ccgtatttag ccagtatgtt ctctagtgtg gttcgttgtt
tttgcgtgag
       61 ccatgagaac gaaccattga gatcatactt actttgcatg tcactcaaaa
attttgcctc
      121 aaaactggtg agctgaattt ttgcagttaa agcatcgtgt agtgtttttc
ttagtccgtt
      181 acgtaggtag gaatctgatg taatggttgt tggtattttg tcaccattca
tttttatctg
      241 gttgttctca agttcggtta cgagatccat ttgtctatct agttcaactt
ggaaaatcaa
      301 cgtatcagtc gggcggcctc gcttatcaac caccaatttc atattgctgt
aagtgtttaa
      361 atctttactt attggtttca aaacccattg gttaagcctt ttaaactcat
ggtagttatt
      421 ttcaagcatt aacatgaact taaattcatc aaggctaatc tctatatttg
ccttgtgagt
      481 tttcttttgt gttagttctt ttaataacca ctcataaatc ctcatagagt
atttgttttc
      541 aaaagactta acatgttcca gattatattt tatgaatttt tttaactgga
aaagataagg
      601 caatatctct tcactaaaaa ctaattctaa tttttcgctt gagaacttgg
catagtttgt
      661 ccactggaaa atctcaaagc ctttaaccaa aggattcctg atttccacag
ttctcgtcat
      721 cagctctctg gttgctttag ctaatacacc ataagcattt tccctactga
tgttcatcat
      781 ctgagcgtat tggttataag tgaacgatac cgtccgttct ttccttgtag
ggttttcaat
      841 cgtggggttg agtagtgcca cacagcataa aattagcttg gtttcatgct
ccgttaagtc
      901 atagcgacta atcgctagtt catttgcttt gaaaacaact aattcagaca
tacatctcaa
      961 ttggtctagg tgattttaat cactatacca attgagatgg gctagtcaat
gataattact
```

1021 agtccttttc ctttgagttg tgggtatctg taaattctgc tagacctttg ctggaaaact
1081 tgtaaattct gctagaccct ctgtaaattc cgctagacct ttgtgtgttt tttttgttta
1141 tattcaagtg gttataattt atagaataaa gaaagaataa aaaaagataa aaagaataga
1201 tcccagccct gtgtataact cactacttta gtcagttccg cagtattaca aaaggatgtc
1261 gcaaacgctg tttgctcctc tacaaaacag accttaaaac cctaaaggct taagtagcac
1321 cctcgcaagc tcggttgcgg ccgcaatcgg gcaaatcgct gaatattcct tttgtctccg
1381 accatcaggc acctgagtcg ctgtcttttt cgtgacattc agttcgctgc gctcacggct
1441 ctggcagtga atgggggtaa atggcactac aggcgccttt tatggattca tgcaaggaaa
1501 ctacccataa tacaagaaaa gcccgtcacg ggcttctcag ggcgttttat ggcgggtctg
1561 ctatgtggtg ctatctgact ttttgctgtt cagcagttcc tgccctctga ttttccagtc
1621 tgaccacttc ggattatccc gtgacaggtc attcagactg gctaatgcac ccagtaaggc
1681 agcggtatca tcaacggggt ctgacgctca gtggaacgaa aactcacgtt aagggatttt
1741 ggtcatgaga ttatcaaaaa ggatcttcac ctagatcctt ttaaattaaa aatgaagttt
1801 taaatcaatc taaagtatat atgagtaaac ttggtctgac agttaccaat gcttaatcag
1861 tgaggcacct atctcagcga tctgtctatt tcgttcatcc atagttgcct gactccccgt
1921 cgtgtagata actacgatac gggagggctt accatctggc cccagtgctg caatgatacc
1981 gcgagaccca cgctcaccgg ctccagattt atcagcaata aaccagccag ccggaagggc
2041 cgagcgcaga agtggtcctg caactttatc cgcctccatc cagtctatta attgttgccg
2101 ggaagctaga gtaagtagtt cgccagttaa tagtttgcgc aacgttgttg ccattgctac
2161 aggcatcgtg gtgtcacgct cgtcgtttgg tatggcttca ttcagctccg gttcccaacg
2221 atcaaggcga gttacatgat cccccatgtt gtgcaaaaaa gcggttagct ccttcggtcc
2281 tccgatcgtt gtcagaagta agttggccgc agtgttatca ctcatggtta tggcagcact
2341 gcataattct cttactgtca tgccatccgt aagatgcttt tctgtgactg gtgagtactc
2401 aaccaagtca ttctgagaat agtgtatgcg gcgaccgagt tgctcttgcc cggcgtcaat
2461 acgggataat accgcgccac atagcagaac tttaaaagtg ctcatcattg gaaaacgttc
2521 ttcggggcga aaactctcaa ggatcttacc gctgttgaga tccagttcga tgtaacccac
2581 tcgtgcaccc aactgatctt cagcatcttt tactttcacc agcgtttctg ggtgagcaaa

2641 aacaggaagg caaaatgccg caaaaaaggg aataagggcg acacggaaat
gttgaatact
2701 catactcttc ctttttcaat attattgaag catttatcag ggttattgtc
tcatgagcgg
2761 atacatattt gaatgtattt agaaaaataa acaaataggg gttccgcgca
catttccccg
2821 aaaagtgcca cctgcatcga tgaagattct tgctcaattg ttatcagcta
tgcgccgacc
2881 agaacacctt gccgatcagc caaacgtctc ttcaggccac tgactagcga
taactttccc
2941 cacaacggaa caactctcat tgcatgggat cattgggtac tgtgggttta
gtggttgtaa
3001 aaacacctga ccgctatccc tgatcagttt cttgaaggta aactcatcac
ccccaagtct
3061 ggctatgcag aaatcacctg gctcaacagc ctgctcaggg tcaacgagaa
ttaacattcc
3121 gtcaggaaag cttggcttgg agcctgttgg tgcggtcatg gaattacctt
caacctcaag
3181 ccagaatgca gaatcactgg ctttttggt tgtgcttacc catctctccg
catcaccttt
3241 ggtaaaggtt ctaagcttag gtgagaacat ccctgcctga acatgagaaa
aaacagggta
3301 ctcatactca cttctaagtg acggctgcat actaaccgct tcatacatct
cgtagatttc
3361 tctggcgatt gaagggctaa attcttcaac gctaactttg agaatttttg
taagcaatgc
3421 ggcgttataa gcatttaatg cattgatgcc attaaataaa gcaccaacgc
ctgactgccc
3481 catccccatc ttgtctgcga cagattcctg ggataagcca agttcatttt
tcttttttc
3541 ataaattgct ttaaggcgac gtgcgtcctc aagctgctct tgtgttaatg
gtttcttttt
3601 tgtgctcata cgttaaatct atcaccgcaa gggataaata tctagttggg
taacgccagg
3661 gttttcccag tcacgacgtt gtaaaacgac ggccagtgaa ttgtaatacg
actcactata
3721 gggcggatcc acagtttgat tacagtttag tcagagctat tgactattaa
aaaaccgctt
3781 gataaaattt tagctgtaag tgatgaggct ataaaaaata gtataacctc
atcactaaaa
3841 aatcatacaa ggaggtttac ctgcatgacc aagaaagtgg cgatctacac
ccgtgttagc
3901 accaccaacc aagcggagga aggtttcagc atcgacgaac agattgatcg
tctgaccaaa
3961 tacgcggagg cgatgggctg gcaagtgagc gacacctata ccgatgcggg
tttcagcggt
4021 gcgaagctgg aacgtccggc gatgcagcgt ctgatcaacg acattgagaa
caaagcgttt
4081 gataccgtgc tggtttacaa gctggaccgt ctgagccgta gcgttcgtga
caccctgtat
4141 ctggtgaaag atgttttcac caagaacaag atcgacttca tcagcctgaa
cgaaagcatc
4201 gataccagca gcgcgatggg tagcctgttc ctgaccatcc tgagcgcgat
taacgagttt

4261 gaacgtgaga acattaaaga gcgtatgacc atgggcaagc tgggccgtgc gaaaagcggc
4321 aagagcatga tgtggaccaa aaccgcgttc ggttactatc acaaccgtaa gaccggcatc
4381 ctggaaattg tgccgctgca agcgaccatc gttgagcaga tttttaccga ttacctgagc
4441 ggtatcagcc tgaccaaact gcgtgacaag ctgaacgaaa gcggtcacat cggcaaagat
4501 attccgtgga gctatcgtac cctgcgtcag accctggaca acccggtgta ctgcggttat
4561 atcaaattca aggatagcct gtttgaaggc atgcacaagc cgatcattcc gtacgaaacc
4621 tatctgaaag ttcaaaagga gctggaggaa cgtcagcaac agacctacga gcgtaacaac
4681 aacccgcgtc cgttccaggc gaaatacatg ctgagcggca tggcgcgttg cggttattgc
4741 ggtgcgccgc tgaaaatcgt gctgggtcac aagcgtaagg acggcagccg taccatgaag
4801 tatcactgcg cgaaccgttt cccgcgtaaa accaagggta ttaccgtgta caacgacaac
4861 aagaaatgcg atagcggcac ctatgacctg agcaacctgg aaaacaccgt tatcgacaac
4921 ctgattggtt ttcaagagaa caacgatagc ctgctgaaga tcattaacgg caacaaccag
4981 ccgatcctgg acaccagcag cttcaagaaa caaatcagcc agattgataa gaaaatccag
5041 aagaacagcg atctgtacct gaacgacttt attaccatgg atgagctgaa ggaccgtacc
5101 gatagcctgc aagcggaaaa gaaactgctg aaagcgaaga tcagcgagaa caaattcaac
5161 gacagcaccg atgtgtttga actggttaag acccaactgg gtagcatccc gattaacgag
5221 ctgagctatg acaacaagaa aaagattgtg aacaacctgg ttagcaaagt ggatgttacc
5281 gcggacaacg ttgatatcat tttcaagttt cagctggcgt aa
//

```
LOCUS       pLAR062                 5347 bp    DNA     circular UNA 07-
OCT-2020
DEFINITION  .
ACCESSION   urn.local...27-exuztbt
VERSION     urn.local...27-exuztbt
KEYWORDS    .
SOURCE
  ORGANISM  .
FEATURES             Location/Qualifiers
     CDS             complement(86..1036)
                     /label="repA101"
     CDS             complement(1928..2788)
                     /label="bla"
     misc_feature    2937..2958
                     /label="RP-2"
     CDS             complement(2981..3694)
                     /label="cI"
     misc_feature    3810..3949
                     /created_by="lrf"
                     /label="pEno"
     misc_feature    3950..5347
                     /label="BL3"
ORIGIN
        1 atcggatccc gggcccgtcg aatcggatcc cgggcccgtc gactgcagag
gcctgtcact
       61 gatagataca agagccataa gaacctcaga tccttccgta tttagccagt
atgttctcta
      121 gtgtggttcg ttgtttttgc gtgagccatg agaacgaacc attgagatca
tacttacttt
      181 gcatgtcact caaaaatttt gcctcaaaac tggtgagctg aatttttgca
gttaaagcat
      241 cgtgtagtgt ttttcttagt ccgttacgta ggtaggaatc tgatgtaatg
gttgttggta
      301 ttttgtcacc attcattttt atctggttgt tctcaagttc ggttacgaga
tccatttgtc
      361 tatctagttc aacttggaaa atcaacgtat cagtcgggcg gcctcgctta
tcaaccacca
      421 atttcatatt gctgtaagtg tttaaatctt tacttattgg tttcaaaacc
cattggttaa
      481 gccttttaaa ctcatggtag ttattttcaa gcattaacat gaacttaaat
tcatcaaggc
      541 taatctctat atttgccttg tgagttttct tttgtgttag ttcttttaat
aaccactcat
      601 aaatcctcat agagtatttg ttttcaaaag acttaacatg ttccagatta
tattttatga
      661 attttttaa ctggaaaaga taaggcaata tctcttcact aaaaactaat
tctaattttt
      721 cgcttgagaa cttggcatag tttgtccact ggaaaatctc aaagccttta
accaaaggat
      781 tcctgatttc cacagttctc gtcatcagct ctctggttgc tttagctaat
acaccataag
      841 cattttccct actgatgttc atcatctgag cgtattggtt ataagtgaac
gataccgtcc
```

901 gttctttcct tgtagggttt tcaatcgtgg ggttgagtag tgccacacag
cataaaatta
961 gcttggtttc atgctccgtt aagtcatagc gactaatcgc tagttcattt
gctttgaaaa
1021 caactaattc agacatacat ctcaattggt ctaggtgatt ttaatcacta
taccaattga
1081 gatgggctag tcaatgataa ttactagtcc ttttcctttg agttgtgggt
atctgtaaat
1141 tctgctagac ctttgctgga aaacttgtaa attctgctag accctctgta
aattccgcta
1201 gacctttgtg tgtttttttt gtttatattc aagtggttat aatttataga
ataaagaaag
1261 aataaaaaaa gataaaaaga atagatccca gccctgtgta taactcacta
ctttagtcag
1321 ttccgcagta ttacaaaagg atgtcgcaaa cgctgtttgc tcctctacaa
aacagacctt
1381 aaaaccctaa aggcttaagt agcaccctcg caagctcggt tgcggccgca
atcgggcaaa
1441 tcgctgaata ttccttttgt ctccgaccat caggcacctg agtcgctgtc
tttttcgtga
1501 cattcagttc gctgcgctca cggctctggc agtgaatggg ggtaaatggc
actacaggcg
1561 ccttttatgg attcatgcaa ggaaactacc cataatacaa gaaaagcccg
tcacgggctt
1621 ctcagggcgt tttatggcgg gtctgctatg tggtgctatc tgactttttg
ctgttcagca
1681 gttcctgccc tctgattttc cagtctgacc acttcggatt atcccgtgac
aggtcattca
1741 gactggctaa tgcacccagt aaggcagcgg tatcatcaac ggggtctgac
gctcagtgga
1801 acgaaaactc acgttaaggg attttggtca tgagattatc aaaaaggatc
ttcacctaga
1861 tccttttaaa ttaaaaatga agttttaaat caatctaaag tatatatgag
taaacttggt
1921 ctgacagtta ccaatgctta atcagtgagg cacctatctc agcgatctgt
ctatttcgtt
1981 catccatagt tgcctgactc cccgtcgtgt agataactac gatacgggag
ggcttaccat
2041 ctggccccag tgctgcaatg ataccgcgag acccacgctc accggctcca
gatttatcag
2101 caataaacca gccagccgga agggccgagc gcagaagtgg tcctgcaact
ttatccgcct
2161 ccatccagtc tattaattgt tgccgggaag ctagagtaag tagttcgcca
gttaatagtt
2221 tgcgcaacgt tgttgccatt gctacaggca tcgtggtgtc acgctcgtcg
tttggtatgg
2281 cttcattcag ctccggttcc caacgatcaa ggcgagttac atgatccccc
atgttgtgca
2341 aaaaagcggt tagctccttc ggtcctccga tcgttgtcag aagtaagttg
gccgcagtgt
2401 tatcactcat ggttatggca gcactgcata attctcttac tgtcatgcca
tccgtaagat
2461 gcttttctgt gactggtgag tactcaacca agtcattctg agaatagtgt
atgcggcgac

2521 cgagttgctc ttgcccggcg tcaatacggg ataataccgc gccacatagc agaactttaa
2581 aagtgctcat cattggaaaa cgttcttcgg ggcgaaaact ctcaaggatc ttaccgctgt
2641 tgagatccag ttcgatgtaa cccactcgtg cacccaactg atcttcagca tcttttactt
2701 tcaccagcgt ttctgggtga gcaaaaacag gaaggcaaaa tgccgcaaaa aagggaataa
2761 gggcgacacg gaaatgttga atactcatac tcttcctttt tcaatattat tgaagcattt
2821 atcagggtta ttgtctcatg agcggataca tatttgaatg tatttagaaa aataaacaaa
2881 taggggttcc gcgcacattt ccccgaaaag tgccacctgc atcgatgaag attcttgctc
2941 aattgttatc agctatgcgc cgaccagaac accttgccga tcagccaaac gtctcttcag
3001 gccactgact agcgataact ttccccacaa cggaacaact ctcattgcat gggatcattg
3061 ggtactgtgg gtttagtggt tgtaaaaaca cctgaccgct atccctgatc agtttcttga
3121 aggtaaactc atcaccccca agtctggcta tgcagaaatc acctggctca acagcctgct
3181 cagggtcaac gagaattaac attccgtcag gaaagcttgg cttggagcct gttggtgcgg
3241 tcatggaatt accttcaacc tcaagccaga atgcagaatc actggctttt ttggttgtgc
3301 ttacccatct ctccgcatca cctttggtaa aggttctaag cttaggtgag aacatccctg
3361 cctgaacatg agaaaaaaca gggtactcat actcacttct aagtgacggc tgcatactaa
3421 ccgcttcata catctcgtag atttctctgg cgattgaagg gctaaattct tcaacgctaa
3481 ctttgagaat ttttgtaagc aatgcggcgt tataagcatt taatgcattg atgccattaa
3541 ataaagcacc aacgcctgac tgccccatcc ccatcttgtc tgcgacagat tcctgggata
3601 agccaagttc atttttcttt ttttcataaa ttgctttaag gcgacgtgcg tcctcaagct
3661 gctcttgtgt taatggtttc tttttgtgc tcatacgtta aatctatcac cgcaagggat
3721 aaatatctag ttgggtaacg ccagggtttt cccagtcacg acgttgtaaa acgacggcca
3781 gtgaattgta atacgactca ctatagggcg gatccacagt ttgattacag tttagtcaga
3841 gctattgact attaaaaaac cgcttgataa aattttagct gtaagtgatg aggctataaa
3901 aaatagtata acctcatcac taaaaaatca tacaaggagg tttacctgca tgaaactgcg
3961 tgcggcgatt tacgtgcgtg ttagcaccat ggagcaggcg gaggaaggtt atagcatcag
4021 cgcgcaaacc gaaaaactga agagctacgc gaacgcgaaa gactatcagg tggttaaggt
4081 gttcaccgat ccgggttaca gcggtgcgaa actggagcgt ccgggtctgc aaaacatgat

4141 caaaagcatt gagagcaagg aaattgacgt ggttctggtt tataaactgg
atcgtctgag
4201 ccgtagccag aagaacaccc tgttcctgat cgaggacgtg tttctgaaga
accacgttca
4261 gttcaccagc atgcaagaaa gcttcgatac cagcaccagc tttggtcgtg
cgatgatcgg
4321 cattctgagc gtgtttgcgc agctggagcg tgacgcgatc accgaacgta
tgcaaatggg
4381 tgcgaaagag cgtgcgaaag cgggcatgtg gcgtggtggc ccgcagagcc
gtctgccgtt
4441 cggttaccgt tatattgatg gcgtgctgct ggttgacgat tacgaggcga
tgatcgttaa
4501 atacatgtat accgagttca ttaagggtac cccgctgacc aaaatccaga
gcaaggtggc
4561 ggcgaaattt ccggttaagg aaaccctgat ctacccgagc attatgaaga
acatcctgca
4621 aaacaacatc tacatcggca agatcaagta cgcgggcgag acctatgaag
gcctgcacga
4681 gcacatcctg gacaccgaaa cctacgataa agcgcagcaa ctgtgggagc
accgtaacac
4741 caacaagaaa aagtacttcg aaagcaagta tctgctgagc ggtattctgt
attgcggcca
4801 ctgcggtggc aaaatggcga gcaccggtgc gggtctgctg aagagcggcg
agcgtgtgac
4861 cgactacatc tgctatagca aaaagggcac cccgagccac atggtggttg
accgtaactg
4921 cccgagcaag cgtcaccgtg tgaaccgtct ggacccgaaa attgttgaac
tgctgaagac
4981 catcaccttc gaggaaatgc agaaagacaa cagctttacc gataacacca
ccaccattaa
5041 gagcgagatc gaaagcctgg acaccaaaat cagcaagctg ctggacctgt
accaagatgg
5101 tctggtgccg attgacgttc tgaacgatcg tatcagcaaa ctgaacgacg
ataaggagct
5161 gctgcaagaa accctgatta gccagaaaaa gcaaatccac ccggaggaaa
ttgcgaaaaa
5221 catccaaacc gcgaaggact ttgattgggc gaacagcgac agcgcggcga
agcgtgcgat
5281 ggtgcgtgcg ctgatcaaca aagttgagct gaccaacgaa gatatgaaga
ttgaatggaa
5341 catctaa
//

```
LOCUS       pLAR067                 3165 bp    DNA     circular     26-
FEB-2019
DEFINITION  .
ACCESSION   urn.local...5e-exvbmdg
KEYWORDS    .
SOURCE
  ORGANISM  .
FEATURES             Location/Qualifiers
     misc_feature    1..285
                     /label="laczalpha"
     misc_feature    61..78
                     /label="seq5_R"
     misc_feature    101..118
                     /label="seq4_R"
     misc_feature    218..236
                     /label="seq3_F"
     misc_feature    288..307
                     /label="plasmid#1 F"
     misc_feature    292..336
                     /label="c31"
     misc_feature    661..1053
                     /label="lambda attP"
     misc_feature    1054..1490
                     /label="oriR6K"
     misc_feature    1544..2338
                     /label="Kan"
     misc_feature    2232..2249
                     /label="seq1_F"
     misc_feature    2464..2481
                     /label="seq6_F"
     misc_feature    2726..2743
                     /label="seq2_F"
     misc_feature    2786..2827
                     /label=31
     misc_feature    2840..3157
                     /label="Promoter_P_1"
                     /note="Geneious type: promoter prokaryotic"
ORIGIN
        1 atgaccatga ttacgaatag tagctcggta cccggggacc ctctagagtc
gacctgccgc
       61 catgcaagct tggcactggc cgtcgtttta caacgtcgtg actgggaaaa
ccctggcgtt
      121 acccaactta atcgccttgc agcacatccc cctttcgcca gctggcgtaa
tagcgaagag
      181 gcccgcaccg atcgcccttc ccaacagttg cgcagcctga atggcgaatg
gcgagcttgg
      241 ctgttttggc ggatgagaga agattttcag cctgatacag attaaggatc
ctgcgggtgc
      301 cagggcgtgc ccttgggctc cccgggcgcg tactccacct cactcgagaa
ttctcatgtt
      361 tgacagctta tcactgatca gtgaattaat ggcgatgacg catcctcacg
ataatatccg
      421 ggtaggcgca atcactttcg tctctactcc gttacaaagc gaggctgggt
atttcccggc
```

481 ctttctgtta tccgaaatcc actgaaagca cagcggctgg ctgaggagat aaataataaa
541 cgaggggctg tatgcacaaa gcatcttctg ttgagttaag aacgagtatc gagatggcac
601 atagccttgc tcaaattgga atcaggtttg tgccaatacc agtagaaaca gacgaagaag
661 ctagctttgc actggattgc gaggctttgt gcttctctgg agtgcgacag gtttgatgac
721 aaaaaattag cgcaagaaga caaaaatcac cttgcgctaa tgctctgtta caggtcacta
781 ataccatcta agtagttgat tcatagtgac tgcatatatg ttgtgtttta cagtattatg
841 tagtctgttt tttatgcaaa atctaattta atatattgat atttatatca ttttacgttt
901 ctcgttcagc ttttttatac taagttggca ttataaaaaa gcattgctta tcaatttgtt
961 gcaacgaaca ggtcactatc agtcaaaata aaatcattat ttgatttcaa ttttgtccca
1021 ctccctgcct ctgtcatcac gatactgtga tgccatggct aattcccatg tcagccgtta
1081 agtgttcctg tgtcactcaa aattgctttg agaggctcta agggcttctc agtgcgttac
1141 atccctggct tgttgtccac aaccgttaaa ccttaaaagc tttaaaagcc ttatatattc
1201 tttttttct tataaaactt aaaaccttag aggctattta agttgctgat ttatattaat
1261 tttattgttc aaacatgaga gcttagtacg tgaaacatga gagcttagta cgttagccat
1321 gagagcttag tacgttagcc atgagggttt agttcgttaa acatgagagc ttagtacgtt
1381 aaacatgaga gcttagtacg tgaaacatga gagcttagta cgtactatca acaggttgaa
1441 ctgctgatct tcagatcctc tacgccggac gcatcgtggc cggatcttgc ggccgcaaaa
1501 attaaaaatg aagttttgac ggtatcgaac cccagagtcc cgctcagaag aactcgtcaa
1561 gaaggcgata gaaggcgatg cgctgcgaat cgggagcggc gataccgtaa agcacgagga
1621 agcggtcagc ccattcgccg ccaagctctt cagcaatatc acgggtagcc aacgctatgt
1681 cctgatagcg gtccgccaca cccagccggc cacagtcgat gaatccagaa aagcggccat
1741 tttccaccat gatattcggc aagcaggcat cgccatgggt cacgacgaga tcctcgccgt
1801 cgggcatccg cgccttgagc ctggcgaaca gttcggctgg cgcgagcccc tgatgctctt
1861 cgtccagatc atcctgatcg acaagaccgg cttccatccg agtacgtgct cgctcgatgc
1921 gatgtttcgc ttggtggtcg aatgggcagg tagccggatc aagcgtatgc agccgccgca
1981 ttgcatcagc catgatggat actttctcgg caggagcaag gtgagatgac aggagatcct
2041 gccccggcac ttcgcccaat agcagccagt cccttcccgc ttcagtgaca acgtcgagca

2101 cagctgcgca aggaacgccc gtcgtggcca gccacgatag ccgcgctgcc
tcgtcttgga
2161 gttcattcag ggcaccggac aggtcggtct tgacaaaaag aaccgggcgc
ccctgcgctg
2221 acagccggaa cacggcggca tcagagcagc cgattgtctg ttgtgcccag
tcatagccga
2281 atagcctctc cacccaagcg gccggagaac ctgcgtgcaa tccatcttgt
tcaatcatgc
2341 gaaacgatcc tcatcctgtc tcttgatcca ctagattatt gaagcattta
tcagggttat
2401 tgtctcatga gcggatacat atttgaatgt atttagaaaa ataaacaaat
aggggttccg
2461 cgcacatttc cccgaaaagt gccacctgca tcgatggccc ccgatggtag
tgtggggtct
2521 ccccatgcga gagtagggaa ctgccaggca tcaaataaaa cgaaaggctc
agtcgaaaga
2581 ctgggccttt cgttttatct gttgtttgtc ggtgaacgct ctcctgagta
ggacaaatcc
2641 gccgggagcg gatttgaacg ttgcgaagca acggcccgga gggtggcggg
caggacgccc
2701 gccataaact gccaggcatc aaattaagca gaaggccatc ctgacggatg
gcctttttgc
2761 gtggccagtg ccaagcttgc atgccgtgcc ccaactgggg taacctttga
gttctctcag
2821 ttgggggcgt agggtctagc cgccattcag agaagaaacc aattgtccat
attgcatcag
2881 acattgccgt cactgcgtct tttactggct cttctcgcta accaaaccgg
taaccccgct
2941 tattaaaagc attctgtaac aaagcgggac caaggccatg acaaaaacgc
gtagcaaaag
3001 tgtctataat cacggcagaa aagtccacat tgattatttg cacggcgtca
cactttgcta
3061 tgccatagca tttttatcca taagattagc ggatcctacc tgacgctttt
tatcgcaact
3121 ctctactgtt tctccatacc cgtttttttg gatggaggaa ttcat
//

```
LOCUS       pLAR074                 5565 bp    DNA     circular UNA 24-
OCT-2019
DEFINITION  .
ACCESSION   urn.local...28-exuztbt
VERSION     urn.local...28-exuztbt
KEYWORDS    .
SOURCE
  ORGANISM  .
FEATURES             Location/Qualifiers
     misc_feature    85..1831
                     /label="CtBxb_v2"
     misc_feature    85..224
                     /created_by="lrf"
                     /label="promoter"
     CDS             complement(1923..2873)
                     /label="repA101"
     CDS             complement(3765..4625)
                     /label="bla"
     misc_feature    4774..4795
                     /label="RP-2"
     CDS             complement(4818..5531)
                     /label="cI"
ORIGIN
        1 ggaaacagct atgaccatga ttacgccaag cttgcatgca ggcctctgca
gtcgacgggc
       61 ccgggatccg atatacatct agatggatcc acagtttgat tacagtttag
tcagagctat
      121 tgactattaa aaaaccgctt gataaaattt tagctgtaag tgatgaggct
ataaaaaata
      181 gtataacctc atcactaaaa aatcatacaa ggaggtttac ctgcatgaga
gcgttagttg
      241 ttataaggct tagtagagtg acggacgcta caacaagtcc ggagaggcaa
cttgaatcgt
      301 gccagcagct ttgtgcacag agaggttggg acgtagttgg cgtagccgaa
gatctggatg
      361 tgagtggagc cgtagatccc tttgatagga aaagaaggcc gaatcttgct
agatggcttg
      421 cgtttgaaga acaaccattt gatgttatag tggcgtatag ggtggataga
cttaccagat
      481 cgattagaca tttgcaacag ttagtacatt gggccgagga tcacaaaaaa
ttggtggtat
      541 ccgctacaga agcacatttt gatactacca cacctttgc cgcagtggta
attgccttga
      601 tgggtactgt ggcacagatg gaattagaag ctataaagga gagaaacaga
agtgcagcac
      661 attttaatat aagggctgga aaatatagag gatctctgcc tccgtgggga
tatttaccga
      721 cgagagtgga tggtgaatgg agattagttc ctgaccccgt tcagagggag
agaatactgg
      781 aagtatatca tagagtagta gacaatcatg agccgcttca tctggttgca
cacgatctta
      841 acaggagggg tgttctttca cctaaagatt attttgcgca gcttcaaggt
agagaaccgc
```

901 aaggcaggga atggagtgca accgcactta aaagatcaat gattagcgag gcaatgctgg
961 gttatgcgac tttgaatgga aaaactgtga gagatgatga cggcgcgcct ctggtgagag
1021 cggaacccat attgacaagg gaacagttag aggcacttag ggcagaactt gtaaaaacat
1081 ccagagccaa accggcagtt agcactccga gtttgttgct tagggtattg ttctgcgccg
1141 tttgcggaga acctgcctat aaattcgcag gcggaggaag aaaacatcct aggtacagat
1201 gcagaagtat gggatttccg aaacattgcg gaaatggaac cgttgcaatg gcagaatggg
1261 acgcgttttg tgaggaacag gttctggatc ttttaggaga tgcagaaaga ttggaaaaag
1321 tttgggttgc aggaagtgac agtgcagttg aattagcaga agtgaatgca gagctggttg
1381 atttgactag tttaatagga agcccagctt acagggctgg ctctccccaa agagaagcac
1441 ttgacgcaag gattgctgca ctggctgcaa gacaagaaga actggaagga ttggaagcca
1501 gaccctcagg atgggaatgg agagagaccg gccagagatt tggcgactgg tggagggagc
1561 aggacacagc agcaaaaaat acatggttga gaagcatgaa tgtgagactt acctttgatg
1621 taaggggtgg actgacaaga actattgatt tcggtgatct tcaagaatat gaacagcatc
1681 ttagacttgg ctccgtagtg gaaaggctgc acaccggaat gtcttgacta gcataacccc
1741 ttggggcctc taaacgggtc ttgaggggtt ttttgtcggc cggcttgtcg acgacggcgg
1801 tctccgtcgt caggatcatc cgggcggatc catcggatcc cgggcccgtc gaatctagat
1861 gcattcgcga ggtaccgagc tcgaattcac tgtcactgat agatacaaga gccataagaa
1921 cctcagatcc ttccgtattt agccagtatg ttctctagtg tggttcgttg tttttgcgtg
1981 agccatgaga acgaaccatt gagatcatac ttactttgca tgtcactcaa aaattttgcc
2041 tcaaaactgg tgagctgaat ttttgcagtt aaagcatcgt gtagtgtttt tcttagtccg
2101 ttacgtaggt aggaatctga tgtaatggtt gttggtattt tgtcaccatt catttttatc
2161 tggttgttct caagttcggt tacgagatcc atttgtctat ctagttcaac ttggaaaatc
2221 aacgtatcag tcgggcggcc tcgcttatca accaccaatt tcatattgct gtaagtgttt
2281 aaatctttac ttattggttt caaaacccat tggttaagcc ttttaaactc atggtagtta
2341 ttttcaagca ttaacatgaa cttaaattca tcaaggctaa tctctatatt tgccttgtga
2401 gttttctttt gtgttagttc ttttaataac cactcataaa tcctcataga gtatttgttt
2461 tcaaaagact taacatgttc cagattatat tttatgaatt tttttaactg gaaaagataa

2521 ggcaatatct cttcactaaa aactaattct aatttttcgc ttgagaactt ggcatagttt
2581 gtccactgga aaatctcaaa gcctttaacc aaaggattcc tgatttccac agttctcgtc
2641 atcagctctc tggttgcttt agctaataca ccataagcat tttccctact gatgttcatc
2701 atctgagcgt attggttata agtgaacgat accgtccgtt ctttccttgt agggttttca
2761 atcgtggggt tgagtagtgc cacacagcat aaaattagct tggtttcatg ctccgttaag
2821 tcatagcgac taatcgctag ttcatttgct ttgaaaacaa ctaattcaga catacatctc
2881 aattggtcta ggtgatttta atcactatac caattgagat gggctagtca atgataatta
2941 ctagtccttt tcctttgagt tgtgggtatc tgtaaattct gctagacctt tgctggaaaa
3001 cttgtaaatt ctgctagacc ctctgtaaat tccgctagac ctttgtgtgt tttttttgtt
3061 tatattcaag tggttataat ttatagaata aagaaagaat aaaaaaagat aaaaagaata
3121 gatcccagcc ctgtgtataa ctcactactt tagtcagttc cgcagtatta caaaaggatg
3181 tcgcaaacgc tgtttgctcc tctacaaaac agaccttaaa accctaaagg cttaagtagc
3241 accctcgcaa gctcggttgc ggccgcaatc gggcaaatcg ctgaatattc cttttgtctc
3301 cgaccatcag gcacctgagt cgctgtcttt ttcgtgacat tcagttcgct gcgctcacgg
3361 ctctggcagt gaatgggggt aaatggcact acaggcgcct tttatggatt catgcaagga
3421 aactacccat aatacaagaa aagcccgtca cgggcttctc agggcgtttt atggcgggtc
3481 tgctatgtgg tgctatctga ctttttgctg ttcagcagtt cctgccctct gattttccag
3541 tctgaccact tcggattatc ccgtgacagg tcattcagac tggctaatgc acccagtaag
3601 gcagcggtat catcaacggg gtctgacgct cagtggaacg aaaactcacg ttaagggatt
3661 ttggtcatga gattatcaaa aaggatcttc acctagatcc ttttaaatta aaaatgaagt
3721 tttaaatcaa tctaaagtat atatgagtaa acttggtctg acagttacca atgcttaatc
3781 agtgaggcac ctatctcagc gatctgtcta tttcgttcat ccatagttgc ctgactcccc
3841 gtcgtgtaga taactacgat acgggagggc ttaccatctg gccccagtgc tgcaatgata
3901 ccgcgagacc cacgctcacc ggctccagat ttatcagcaa taaaccagcc agccggaagg
3961 gccgagcgca gaagtggtcc tgcaacttta tccgcctcca tccagtctat taattgttgc
4021 cgggaagcta gagtaagtag ttcgccagtt aatagtttgc gcaacgttgt tgccattgct
4081 acaggcatcg tggtgtcacg ctcgtcgttt ggtatggctt cattcagctc cggttcccaa

4141 cgatcaaggc gagttacatg atcccccatg ttgtgcaaaa aagcggttag ctccttcggt
4201 cctccgatcg ttgtcagaag taagttggcc gcagtgttat cactcatggt tatggcagca
4261 ctgcataatt ctcttactgt catgccatcc gtaagatgct tttctgtgac tggtgagtac
4321 tcaaccaagt cattctgaga atagtgtatg cggcgaccga gttgctcttg cccggcgtca
4381 atacgggata ataccgcgcc acatagcaga actttaaaag tgctcatcat tggaaaacgt
4441 tcttcggggc gaaaactctc aaggatctta ccgctgttga gatccagttc gatgtaaccc
4501 actcgtgcac ccaactgatc ttcagcatct tttactttca ccagcgtttc tgggtgagca
4561 aaaacaggaa ggcaaaatgc cgcaaaaaag ggaataaggg cgacacggaa atgttgaata
4621 ctcatactct tcctttttca atattattga agcatttatc agggttattg tctcatgagc
4681 ggatacatat ttgaatgtat ttagaaaaat aaacaaatag gggttccgcg cacatttccc
4741 cgaaaagtgc cacctgcatc gatgaagatt cttgctcaat tgttatcagc tatgcgccga
4801 ccagaacacc ttgccgatca gccaaacgtc tcttcaggcc actgactagc gataactttc
4861 cccacaacgg aacaactctc attgcatggg atcattgggt actgtgggtt tagtggttgt
4921 aaaaacacct gaccgctatc cctgatcagt ttcttgaagg taaactcatc acccccaagt
4981 ctggctatgc agaaatcacc tggctcaaca gcctgctcag ggtcaacgag aattaacatt
5041 ccgtcaggaa agcttggctt ggagcctgtt ggtgcggtca tggaattacc ttcaacctca
5101 agccagaatg cagaatcact ggcttttttg gttgtgctta cccatctctc cgcatcacct
5161 ttggtaaagg ttctaagctt aggtgagaac atccctgcct gaacatgaga aaaaacaggg
5221 tactcatact cacttctaag tgacggctgc atactaaccg cttcatacat ctcgtagatt
5281 tctctggcga ttgaagggct aaattcttca acgctaactt tgagaatttt tgtaagcaat
5341 gcggcgttat aagcatttaa tgcattgatg ccattaaata aagcaccaac gcctgactgc
5401 cccatcccca tcttgtctgc gacagattcc tgggataagc caagttcatt tttctttttt
5461 tcataaattg ctttaaggcg acgtgcgtcc tcaagctgct cttgtgttaa tggtttcttt
5521 tttgtgctca tacgttaaat ctatcaccgc aagggataaa tatct
//

```
LOCUS       pLAR080                 3454 bp    DNA     circular     14-
JUN-2022
DEFINITION  .
ACCESSION   urn.local...1c-exuzs7s
KEYWORDS    .
SOURCE
  ORGANISM  .
FEATURES             Location/Qualifiers
     misc_feature    1..31
                     /label="FP"
     misc_recomb     10..55
                     /label="FRT"
     primer_bind     36..76
                     /label="CLH5"
     protein_binding 74..76
                     /label="SmaI"
     misc_feature    77..84
                     /label="NotI"
     primer_bind     83..106
                     /Sequence="GCTGTAAAGGAGACTGATAATGGC"
                     /Hairpin_Tm="42.7"
                     /Tm="59.5"
                     /Self_Dimer_Tm="None"
                     /%GC="45.8"
                     /created_by="primer3"
                     /label="bxb-p3"
     misc_feature    85..137
                     /label="Phi370attB"
     misc_feature    138..197
                     /label="PhiBT1attB"
     misc_feature    165..187
                     /Sequence="GATCCAGATGATCCAGCTCCACA"
                     /Hairpin_Tm="43.5"
                     /Tm="61.8"
                     /Self_Dimer_Tm="None"
                     /%GC="52.2"
                     /created_by="primer3"
                     /label="370-P4"
                     /note="Geneious type: primer_bind_reverse"
     misc_feature    198..254
                     /label="R4attB"
     primer_bind     complement(212..232)
                     /Sequence="CACTGCTTCGGCATGGTCATG"
                     /Hairpin_Tm="50.7"
                     /Tm="62.2"
                     /Self_Dimer_Tm="6.6"
                     /%GC="57.1"
                     /created_by="primer3"
                     /label="SpBeta-P3"
     primer_bind     212..231
                     /Sequence="CATGACCATGCCGAAGCAGT"
                     /Hairpin_Tm="56.0"
                     /Tm="61.0"
                     /Self_Dimer_Tm="None"
```

```
                     /%GC="55.0"
                     /created_by="primer3"
                     /label="370-P4-V2"
     misc_feature    255..304
                     /label="BxB1attB"
     primer_bind     283..298
                     /Sequence="CCGTCGTCAGGATCAT"
                     /Hairpin_Tm="None"
                     /Tm="52.2"
                     /Self_Dimer_Tm="None"
                     /%GC="56.3"
                     /created_by="primer3"
                     /label="tp901-p3"
     misc_feature    302..322
                     /Sequence="GGCCTGATAATTGCCAACACA"
                     /Hairpin_Tm="44.7"
                     /Tm="59.2"
                     /Self_Dimer_Tm="None"
                     /%GC="47.6"
                     /created_by="primer3"
                     /label="R4 P4"
                     /note="Geneious type: primer_bind_reverse"
     primer_bind     303..321
                     /Sequence="GCCTGATAATTGCCAACAC"
                     /Hairpin_Tm="None"
                     /Tm="54.7"
                     /Self_Dimer_Tm="None"
                     /%GC="47.4"
                     /created_by="primer3"
                     /label="tg1-p3"
     misc_feature    305..359
                     /label="TP901-1attB"
     misc_feature    360..427
                     /label="RVattB"
     primer_bind     360..376
                     /Sequence="TCTCGTGGTGGTGGAAG"
                     /Hairpin_Tm="None"
                     /Tm="55.6"
                     /Self_Dimer_Tm="None"
                     /%GC="58.8"
                     /created_by="primer3"
                     /label="FC1-P3"
     misc_feature    369..388
                     /Sequence="GGTGGAAGGTGTTGGTGCGG"
                     /Hairpin_Tm="None"
                     /Tm="63.9"
                     /Self_Dimer_Tm="None"
                     /%GC="65.0"
                     /created_by="primer3"
                     /label="bxb-p4"
                     /note="Geneious type: primer_bind_reverse"
     misc_feature    428..492
                     /label="SPBcattB"
     primer_bind     474..492
```

```
                     /Sequence="GTGAACACAATGGGTGCCA"
                     /Hairpin_Tm="52.2"
                     /Tm="58.6"
                     /Self_Dimer_Tm="None"
                     /%GC="52.6"
                     /created_by="primer3"
                     /label="MR11-P3"
     misc_feature    493..538
                     /label="TG1attB"
     misc_feature    complement(508..527)
                     /Sequence="CCGTGAAGGAGAAGGTCTTG"
                     /Hairpin_Tm="45.0"
                     /Tm="57.9"
                     /Self_Dimer_Tm="None"
                     /%GC="55.0"
                     /created_by="primer3"
                     /label="tp901-p4"
                     /note="Geneious type: primer_bind_reverse"
     misc_feature    536..556
                     /Sequence="GTCATATTTAACCGCTTCCCG"
                     /Hairpin_Tm="None"
                     /Tm="57.1"
                     /Self_Dimer_Tm="None"
                     /%GC="47.6"
                     /created_by="primer3"
                     /label="SpBeta-P4"
                     /note="Geneious type: primer_bind_reverse"
     misc_feature    539..624
                     /label="PhiFC1attB"
     primer_bind     567..582
                     /Sequence="CGTGGATGAGCAATAC"
                     /Hairpin_Tm="None"
                     /Tm="48.7"
                     /Self_Dimer_Tm="None"
                     /%GC="50.0"
                     /created_by="primer3"
                     /label="A118_P3_V2"
     primer_bind     619..641
                     /Sequence="GGATAAACAGGTCAACACATCGC"
                     /Hairpin_Tm="None"
                     /Tm="60.2"
                     /Self_Dimer_Tm="None"
                     /%GC="47.8"
                     /modified_by="lrf"
                     /label="A118-P3 and K38"
     misc_feature    625..744
                     /label="MR11attB"
     primer_bind     626..643
                     /Sequence="CAGGTCAACACATCGCAG"
                     /Hairpin_Tm="None"
                     /Tm="56.1"
                     /Self_Dimer_Tm="None"
                     /%GC="55.6"
                     /created_by="primer3"
```

```
                     /label="BL3-P3-V5"
     primer_bind     629..648
                     /Sequence="GTCAACACATCGCAGTTATC"
                     /Hairpin_Tm="None"
                     /Tm="54.8"
                     /Self_Dimer_Tm="None"
                     /%GC="45.0"
                     /created_by="primer3"
                     /label="WB-P3-V2"
     misc_feature    634..652
                     /Sequence="CACATCGCAGTTATCGAAC"
                     /Hairpin_Tm="39.5"
                     /Tm="54.4"
                     /Self_Dimer_Tm="None"
                     /%GC="47.4"
                     /created_by="primer3"
                     /label="FC1-P4"
                     /note="Geneious type: primer_bind_reverse"
     misc_feature    639..656
                     /Sequence="CGCAGTTATCGAACAATC"
                     /Hairpin_Tm="38.1"
                     /Tm="51.5"
                     /Self_Dimer_Tm="None"
                     /%GC="44.4"
                     /created_by="primer3"
                     /label="tg1-p4"
                     /note="Geneious type: primer_bind_reverse"
     primer_bind     665..684
                     /Sequence="TGTATGGAGGCACTTGTATC"
                     /Hairpin_Tm="41.6"
                     /Tm="54.5"
                     /Self_Dimer_Tm="None"
                     /%GC="45.0"
                     /created_by="primer3"
                     /label="Wbeta-P3"
     primer_bind     691..710
                     /Sequence="GGATGTATACCTTCGAAGAC"
                     /Hairpin_Tm="34.3"
                     /Tm="52.7"
                     /Self_Dimer_Tm="26.6"
                     /%GC="45.0"
                     /created_by="primer3"
                     /label="FC1-P4-V2"
     primer_bind     704..725
                     /Sequence="CGAAGACACTTGTACATGATGG"
                     /Hairpin_Tm="None"
                     /Tm="57.2"
                     /Self_Dimer_Tm="15.1"
                     /%GC="45.5"
                     /created_by="primer3"
                     /label="BL3-P3-V2"
     primer_bind     724..747
                     /Sequence="GGATTAGAAGGCAAATCCTTTGAG"
                     /Hairpin_Tm="53.5"
```

```
                     /Tm="57.9"
                     /Self_Dimer_Tm="6.8"
                     /%GC="41.7"
                     /created_by="primer3"
                     /label="BL3-P3"
     misc_feature    745..800
                     /label="PhiK38attB"
     misc_feature    789..806
                     /label="RV P4"
     misc_feature    793..814
                     /Sequence="CTGCGGTCTGTAACTTTTTCGG"
                     /Hairpin_Tm="None"
                     /Tm="60.1"
                     /Self_Dimer_Tm="None"
                     /%GC="50.0"
                     /created_by="primer3"
                     /label="MR11-P4"
                     /note="Geneious type: primer_bind_reverse"
     misc_feature    801..857
                     /label="A118attB"
     misc_feature    858..925
                     /label="WBeta_attB"
     misc_feature    876..898
                     /label="TG1 R"
     primer_bind     876..900
                     /Sequence="GGTGTAACTGTCGTGTTTGTAACGG"
                     /Hairpin_Tm="38.2"
                     /Tm="62.5"
                     /Self_Dimer_Tm="None"
                     /%GC="48.0"
                     /created_by="primer3"
                     /label="BL3-P3-V3"
     misc_feature    899..922
                     /Sequence="GGTACTTCCAACAGCTGGCGTTTC"
                     /Hairpin_Tm="46.0"
                     /Tm="64.4"
                     /Self_Dimer_Tm="19.9"
                     /%GC="54.2"
                     /created_by="primer3"
                     /label="A118-P4"
                     /note="Geneious type: primer_bind_reverse"
     primer_bind     922..940
                     /Sequence="CAGTCAACCTGTTGACATG"
                     /Hairpin_Tm="59.3"
                     /Tm="53.9"
                     /Self_Dimer_Tm="32.9"
                     /%GC="47.4"
                     /created_by="primer3"
                     /label="A118_P4_V2"
     misc_feature    926..988
                     /label="BL3attB"
     primer_bind     962..992
                     /Sequence="GAGGTAGTCACGGCTTTTACGTTAGTTGCGG"
                     /Hairpin_Tm="43.6"
```

```
                     /Tm="68.8"
                     /Self_Dimer_Tm="None"
                     /%GC="51.6"
                     /created_by="primer3"
                     /label="BL3 P4 V5"
     misc_feature    970..990
                     /Sequence="CACGGCTTTTACGTTAGTTGC"
                     /Hairpin_Tm="43.6"
                     /Tm="58.4"
                     /Self_Dimer_Tm="None"
                     /%GC="47.6"
                     /created_by="primer3"
                     /label="Wbeta-P4"
                     /note="Geneious type: primer_bind_reverse"
     misc_feature    989..996
                     /label="NotI"
     primer_bind     997..1023
                     /label="CLH3"
     misc_feature    1006..1023
                     /label="A118 R"
     primer_bind     1006..1022
                     /Sequence="GCGGTACCAAGCTTCAA"
                     /Hairpin_Tm="None"
                     /Tm="54.6"
                     /Self_Dimer_Tm="13.5"
                     /%GC="52.9"
                     /created_by="primer3"
                     /label="WB-P4-V2"
     misc_feature    1007..1028
                     /label="RP"
     misc_feature    1029..1083
                     /label="homology"
     misc_feature    complement(<1033..>1059)
                     /Source="referenceToDocument: urn:local:.:4a-
aepbfa5"
                     /label="pGSs009 extraction (concatenated sequence
1)"
                     /note="Geneious type: Concatenated sequence"
     misc_feature    1033..1059
                     /Original_Bases="CTCATGTTTGACAGCTTATCAC"
                     /label="CTCATGTTTGACAGCTTATCAC"
                     /note="Geneious type: Editing History Replacement"
     misc_feature    complement(1033..>1059)
                     /label="Target for MTase"
     primer_bind     1042..1057
                     /Sequence="AGTGAGGTGGAGTACG"
                     /Hairpin_Tm="None"
                     /Tm="51.2"
                     /Self_Dimer_Tm="None"
                     /%GC="56.3"
                     /created_by="primer3"
                     /label="BL3-P2-V3"
     misc_feature    complement(1050..>1059)
                     /label="c31 attB"
```

```
primer_bind     1078..1103
                /Sequence="CGATGACGCATCCTCACGATAATATC"
                /Hairpin_Tm="37.6"
                /Tm="61.7"
                /Self_Dimer_Tm="12.3"
                /%GC="46.2"
                /created_by="primer3"
                /label="BL3-P4-V2"
primer_bind     1140..1161
                /Sequence="CAAAGCGAGGCTGGGTATTTCC"
                /Hairpin_Tm="39.6"
                /Tm="61.8"
                /Self_Dimer_Tm="0.5"
                /%GC="54.5"
                /created_by="primer3"
                /label="BL3-P4-V3"
misc_feature    2047..2066
                /label="polyattB+hk R"
CDS             complement(2077..3087)
                /label="CDS_1"
misc_feature    3135..3154
                /label="polyattB+hk F"
primer_bind     3321..3339
                /Sequence="GAACGTTGCGAAGCAACGG"
                /Hairpin_Tm="84.3"
                /Tm="60.7"
                /Self_Dimer_Tm="42.2"
                /%GC="57.9"
                /created_by="primer3"
                /label="370-P3"
primer_bind     3365..3384
                /Sequence="CGCCATAAACTGCCAGGCAT"
                /Hairpin_Tm="45.5"
                /Tm="61.4"
                /Self_Dimer_Tm="16.6"
                /%GC="55.0"
                /created_by="primer3"
                /label="370-P3-V2"
primer_bind     3390..3408
                /Sequence="TAAGCAGAAGGCCATCCTG"
                /Hairpin_Tm="43.4"
                /Tm="56.8"
                /Self_Dimer_Tm="10.2"
                /%GC="52.6"
                /created_by="primer3"
                /label="370-P3-V3"
misc_feature    3405..3454
                /label="homology"
primer_bind     3434..4
                /Sequence="TGCCAAGCTTGCATGCCTGCACTTT"
                /Hairpin_Tm="57.1"
                /Tm="68.6"
                /Self_Dimer_Tm="26.3"
                /%GC="52.0"
```

```
                     /created_by="primer3"
                     /label="370-P3-V4"
     misc_feature    3435..3
                     /Sequence="GCCAAGCTTGCATGCCTGCACTT"
                     /Hairpin_Tm="57.1"
                     /Tm="67.2"
                     /Self_Dimer_Tm="26.3"
                     /%GC="56.5"
                     /created_by="primer3"
                     /label="R4 P3"
                     /note="Geneious type: primer_bind_reverse"
ORIGIN
        1 cttttataag aagttcctat actttctaga gaataggaac ttcggaatag
gaactaagga
       61 ggatattcat atgcccgcgg ccgctgtaaa ggagactgat aatggcatgt
acaactatac
      121 tcgtcggtaa aaaggcagtc cttgaccagg tttttgacga aagtgatcca
gatgatccag
      181 ctccacaccc cgaacgcgcg cccaagttgc ccatgaccat gccgaagcag
tggtagaagg
      241 gcaccggcag acactcggcc ggcttgtcga cgacggcggt ctccgtcgtc
aggatcatcc
      301 gggcctgata attgccaaca caattaacat ctcaatcaag gtaaatgctt
tttcgttttt
      361 ctcgtggtgg tggaaggtgt tggtgcgggg ttggccgtgg tcgaggtggg
gtggtggtag
      421 ccattcgagt gcagcatgtc attaatatca gtacagataa agctgtatct
cctgtgaaca
      481 caatgggtgc cagatcagct ccgcgggcaa gaccttctcc ttcacggggt
ggaaggtcat
      541 atttaaccgc ttcccgaaaa atttcgcgtg gatgagcaat actttgattc
agtgaacctt
      601 tgaaaatcgt tttctgttgg ataaacaggt caacacatcg cagttatcga
acaatcttcg
      661 aaaatgtatg gaggcacttg tatcaatata ggatgtatac cttcgaagac
acttgtacat
      721 gatggattag aaggcaaatc ctttgagcgc cggatcaggg agtggacggc
ctgggagcgc
      781 tacacgctgt ggctgcggtc tgtaactttt tcggatcaag ctatgaagga
cgcaaagagg
      841 gaactaaaca cttaattaag gtagcgtcaa cgataggtgt aactgtcgtg
tttgtaacgg
      901 tacttccaac agctggcgtt tcagtcaacc tgttgacatg tttccacaga
caactcacgt
      961 ggaggtagtc acggctttta cgttagttgc ggccgcgggg cggccgcggt
accaagcttc
     1021 aattttataa ttgaattctc gagtgaggtg gagtacgcgt gatcagtgaa
ttaatggcga
     1081 tgacgcatcc tcacgataat atccgggtag gcgcaatcac tttcgtctct
actccgttac
     1141 aaagcgaggc tgggtatttc ccggcctttc tgttatccga aatccactga
aagcacagcg
     1201 gctggctgag gagataaata ataaacgagg ggctgtatgc acaaagcatc
ttctgttgag
```

1261 ttaagaacga gtatcgagat ggcacatagc cttgctcaaa ttggaatcag gtttgtgcca
1321 ataccagtag aaacagacga agaagctagc taatgctctg tctcaggtca ctaatactat
1381 ctaagtagtt gattcatagt gactggatat gttgcgtttt gtcgcattat gtagtctatc
1441 atttaaccac agattagtgt aatgcgatga tttttaagtg attaatgtta ttttgtcatc
1501 ctttaggtga ataagttgta tatttaaaat ctctttaatt atcagtaaat taatgtaagt
1561 aggtcattat tagtcaaaat aaaatcattt gtcgatttca attttgtccc atggctaatt
1621 cccatgtcag ccgttaagtg ttcctgtgtc actcaaaatt gctttgagag gctctaaggg
1681 cttctcagtg cgttacatcc ctggcttgtt gtccacaacc gttaaacctt aaaagcttta
1741 aaagccttat atattctttt ttttcttata aaacttaaaa ccttagaggc tatttaagtt
1801 gctgatttat attaatttta ttgttcaaac atgagagctt agtacgtgaa acatgagagc
1861 ttagtacgtt agccatgaga gcttagtacg ttagccatga gggtttagtt cgttaaacat
1921 gagagcttag tacgttaaac atgagagctt agtacgtgaa acatgagagc ttagtacgta
1981 ctatcaacag gttgaactgc tgatcttcag atcctctacg ccggacgcat cgtggccgga
2041 tcttgcggcc gctcggcttg aacgaattgt tagacattat ttgccgacta ccttggtgat
2101 ctcgcctttc acgtagtgga caaattcttc caactgatct gcgcgcgagg ccaagcgatc
2161 ttcttcttgt ccaagataag cctgtctagc ttcaagtatg acgggctgat actgggccgg
2221 caggcgctcc attgcccagt cggcagcgac atccttcggc gcgattttgc cggttactgc
2281 gctgtaccaa atgcgggaca acgtaagcac tacatttcgc tcatcgccag cccagtcggg
2341 cggcgagttc catagcgtta aggtttcatt tagcgcctca aatagatcct gttcaggaac
2401 cggatcaaag agttcctccg ccgctggacc taccaaggca acgctatgtt ctcttgcttt
2461 tgtcagcaag atagccagat caatgtcgat cgtggctggc tcgaagatac ctgcaagaat
2521 gtcattgcgc tgccattctc caaattgcag ttcgcgctta gctggataac gccacggaat
2581 gatgtcgtcg tgcacaacaa tggtgacttc tacagcgcgg agaatctcgc tctctccagg
2641 ggaagccgaa gtttccaaaa ggtcgttgat caaagctcgc cgcgttgttt catcaagcct
2701 tacggtcacc gtaaccagca aatcaatatc actgtgtggc ttcaggccgc catccactgc
2761 ggagccgtac aaatgtacgg ccagcaacgt cggttcgaga tggcgctcga tgacgccaac
2821 tacctctgat agttgagtcg atacttcggc gatcaccgct tccctcatga tgtttaactt

2881 tgttttaggg cgactgccct gctgcgtaac atcgttgctg ctccataaca
tcaaacatcg
2941 acccacggcg taacgcgctt gctgcttgga tgcccgaggc atagactgta
ccccaaaaaa
3001 acagtcataa caagccatga aaaccgccac tgcgccgtta ccaccgctgc
gttcggtcaa
3061 ggttctggac cagttgcgtg agcgcatacg ctacttgcat tacagcttac
gaaccgaaca
3121 ggcttatgtc cactgggttc gtgccttcat ccgtatcgat ggcccccgat
ggtagtgtgg
3181 ggtctcccca tgcgagagta gggaactgcc aggcatcaaa taaaacgaaa
ggctcagtcg
3241 aaagactggg cctttcgttt tatctgttgt ttgtcggtga acgctctcct
gagtaggaca
3301 aatccgccgg gagcggattt gaacgttgcg aagcaacggc ccggagggtg
gcgggcagga
3361 cgcccgccat aaactgccag gcatcaaatt aagcagaagg ccatcctgac
ggatggcctt
3421 tttgcgtggc cagtgccaag cttgcatgcc tgca
//

```
LOCUS       pLAR140                 3079 bp    DNA     circular     26-
SEP-2019
DEFINITION  .
ACCESSION   urn.local...w-exuzpw0
KEYWORDS    .
SOURCE
  ORGANISM  .
FEATURES             Location/Qualifiers
     misc_feature    1..648
                     /label="Target for MTase"
     misc_feature    2..43
                     /label="C31 attp"
     primer_bind     56..209

/Sequence="AATTGTGAGCGGATAACAATTACGAGCTTCATGCACAGTGAAATCAT

GAAAAATTTATTTGCTTTGTGAGCGGATAACAATTATAATATGTGGAATTGTGAGCGC
                     TCACAATTCCACAACGGTTTCCCTCTAGAAATAATTTTGTTTAACTTTT"
                     /created_by="lrf"
                     /label="T5lac"
     misc_feature    206..225
                     /label="Plasmid #1 R"
     misc_feature    512..581
                     /Original_Bases
                     /label
                     /note="Geneious type: Editing History Insertion"
     misc_feature    <512..>517
                     /label="Target for MTase"
     misc_feature    514..>517
                     /label="plasmid#1 F"
     misc_feature    518..575
                     /label="BxB1attP"
     misc_feature    583..602
                     /label="plasmid#1 F"
     misc_feature    587..631
                     /label="c31 attB"
     misc_feature    956..1348
                     /label="lambda attP"
     misc_feature    1349..1785
                     /label="oriR6K"
     misc_feature    1839..2633
                     /label="Kan"
ORIGIN
        1 cgtgccccaa ctggggtaac ctttgagttc tctcagttgg gggcgtaggg
tctagaattg
       61 tgagcggata acaattacga gcttcatgca cagtgaaatc atgaaaaatt
tatttgcttt
      121 gtgagcggat aacaattata atatgtggaa ttgtgagcgc tcacaattcc
acaacggttt
      181 ccctctagaa ataattttgt ttaactttta taggaggtgt tccatatgac
catgattacg
      241 aatagtagct cggtacccgg ggaccctcta gagtcgacct gccgccatgc
aagcttggca
```

301 ctggccgtcg ttttacaacg tcgtgactgg gaaaaccctg gcgttaccca
acttaatcgc
361 cttgcagcac atcccccttt cgccagctgg cgtaatagcg aagaggcccg
caccgatcgc
421 ccttcccaac agttgcgcag cctgaatggc gaatggcgag cttggctgtt
ttggcggatg
481 agagaagatt ttcagcctga tacagattaa gggatccgtc gtggtttgtc
tggtcaacca
541 ccgcggtctc agtggtgtac ggtacaaacc ccgacactag tgatcctgcg
ggtgccaggg
601 cgtgcccttg ggctccccgg gcgcgtactc cacctcactc gagaattctc
atgtttgaca
661 gcttatcact gatcagtgaa ttaatggcga tgacgcatcc tcacgataat
atccgggtag
721 gcgcaatcac tttcgtctct actccgttac aaagcgaggc tgggtatttc
ccggcctttc
781 tgttatccga aatccactga aagcacagcg gctggctgag gagataaata
ataaacgagg
841 ggctgtatgc acaaagcatc ttctgttgag ttaagaacga gtatcgagat
ggcacatagc
901 cttgctcaaa ttggaatcag gtttgtgcca ataccagtag aaacagacga
agaagctagc
961 tttgcactgg attgcgaggc tttgtgcttc tctggagtgc gacaggtttg
atgacaaaaa
1021 attagcgcaa gaagacaaaa atcaccttgc gctaatgctc tgttacaggt
cactaatacc
1081 atctaagtag ttgattcata gtgactgcat atatgttgtg ttttacagta
ttatgtagtc
1141 tgtttttat gcaaaatcta atttaatata ttgatattta tatcatttta
cgtttctcgt
1201 tcagcttttt tatactaagt tggcattata aaaaagcatt gcttatcaat
ttgttgcaac
1261 gaacaggtca ctatcagtca aaataaaatc attatttgat ttcaattttg
tcccactccc
1321 tgcctctgtc atcacgatac tgtgatgcca tggctaattc ccatgtcagc
cgttaagtgt
1381 tcctgtgtca ctcaaaattg ctttgagagg ctctaagggc ttctcagtgc
gttacatccc
1441 tggcttgttg tccacaaccg ttaaacctta aaagctttaa aagccttata
tattcttttt
1501 tttcttataa aacttaaaac cttagaggct atttaagttg ctgatttata
ttaattttat
1561 tgttcaaaca tgagagctta gtacgtgaaa catgagagct tagtacgtta
gccatgagag
1621 cttagtacgt tagccatgag ggtttagttc gttaaacatg agagcttagt
acgttaaaca
1681 tgagagctta gtacgtgaaa catgagagct tagtacgtac tatcaacagg
ttgaactgct
1741 gatcttcaga tcctctacgc cggacgcatc gtggccggat cttgcggccg
caaaaattaa
1801 aaatgaagtt ttgacggtat cgaaccccag agtcccgctc agaagaactc
gtcaagaagg
1861 cgatagaagg cgatgcgctg cgaatcggga gcggcgatac cgtaaagcac
gaggaagcgg

1921 tcagcccatt cgccgccaag ctcttcagca atatcacggg tagccaacgc tatgtcctga
1981 tagcggtccg ccacacccag ccggccacag tcgatgaatc cagaaaagcg gccattttcc
2041 accatgatat tcggcaagca ggcatcgcca tgggtcacga cgagatcctc gccgtcgggc
2101 atccgcgcct tgagcctggc gaacagttcg gctggcgcga gcccctgatg ctcttcgtcc
2161 agatcatcct gatcgacaag accggcttcc atccgagtac gtgctcgctc gatgcgatgt
2221 ttcgcttggt ggtcgaatgg gcaggtagcc ggatcaagcg tatgcagccg ccgcattgca
2281 tcagccatga tggatacttt ctcggcagga gcaaggtgag atgacaggag atcctgcccc
2341 ggcacttcgc ccaatagcag ccagtccctt cccgcttcag tgacaacgtc gagcacagct
2401 gcgcaaggaa cgcccgtcgt ggccagccac gatagccgcg ctgcctcgtc ttggagttca
2461 ttcagggcac cggacaggtc ggtcttgaca aaaagaaccg ggcgcccctg cgctgacagc
2521 cggaacacgg cggcatcaga gcagccgatt gtctgttgtg cccagtcata gccgaatagc
2581 ctctccaccc aagcggccgg agaacctgcg tgcaatccat cttgttcaat catgcgaaac
2641 gatcctcatc ctgtctcttg atccactaga ttattgaagc atttatcagg gttattgtct
2701 catgagcgga tacatatttg aatgtattta gaaaaataaa caaatagggg ttccgcgcac
2761 atttccccga aaagtgccac ctgcatcgat ggcccccgat ggtagtgtgg ggtctcccca
2821 tgcgagagta gggaactgcc aggcatcaaa taaaacgaaa ggctcagtcg aaagactggg
2881 cctttcgttt tatctgttgt ttgtcggtga acgctctcct gagtaggaca aatccgccgg
2941 gagcggattt gaacgttgcg aagcaacggc ccggagggtg gcgggcagga cgcccgccat
3001 aaactgccag gcatcaaatt aagcagaagg ccatcctgac ggatggcctt tttgcgtggc
3061 cagtgccaag cttgcatgc
//

```
LOCUS       pMTL83151              4476 bp    DNA     circular UNA 17-
JAN-2020
DEFINITION  Shuttle vector pMTL83151. clostron.com.
ACCESSION   urn.local...3pr-auy6c07
VERSION     urn.local...3pr-auy6c07
KEYWORDS    .
SOURCE
  ORGANISM  .
FEATURES             Location/Qualifiers
     terminator      9..62
                     /gene="CD0164 terminator"
                     /standard_name="CD0164 terminator"
     primer_bind     63..80
                     /gene="M13R"
                     /standard_name="M13R"
     CDS             98..361
                     /gene="lacZ alpha"
                     /standard_name="lacZ alpha"
     primer_bind     complement(199..216)
                     /gene="M13F"
                     /standard_name="M13F"
     terminator      363..404
                     /gene="Cpa fdx terminator"
                     /standard_name="Cpa fdx terminator"
     CDS             1131..1448
                     /gene="repH"
                     /standard_name="repH"
     CDS             2120..2743
                     /gene="catP"
                     /standard_name="catP"
     rep_origin      2956..3501
     CDS             3902..4342
                     /gene="traJ"
                     /standard_name="traJ"
ORIGIN
        1 cctgcaggat aaaaaaattg tagataaatt ttataaaata gttttatcta
caattttttt
       61 atcaggaaac agctatgacc gcggccgctg tatccatatg accatgatta
cgaattcgag
      121 ctcggtaccc ggggatcctc tagagtcgac gtcacgcgtc catggagatc
tcgaggcctg
      181 cagacatgca agcttggcac tggccgtcgt tttacaacgt cgtgactggg
aaaaccctgg
      241 cgttacccaa cttaatcgcc ttgcagcaca tccccctttc gccagctggc
gtaatagcga
      301 agaggcccgc accgatcgcc cttcccaaca gttgcgcagc ctgaatggcg
aatggcgcta
      361 gcataaaaat aagaagcctg catttgcagg cttcttattt ttatggcgcg
ccgccattat
      421 ttttttgaac aattgacaat tcatttctta ttttttatta agtgatagtc
aaaaggcata
      481 acagtgctga atagaaagaa atttacagaa aagaaaatta tagaatttag
tatgattaat
```

541 tatactcatt tatgaatgtt taattgaata caaaaaaaaa tacttgttat gtattcaatt
601 acgggttaaa atatagacaa gttgaaaaat ttaataaaaa aataagtcct cagctcttat
661 atattaagct accaacttag tatataagcc aaaacttaaa tgtgctacca acacatcaag
721 ccgttagaga actctatcta tagcaatatt tcaaatgtac cgacatacaa gagaaacatt
781 aactatatat attcaattta tgagattatc ttaacagata taaatgtaaa ttgcaataag
841 taagatttag aagtttatag cctttgtgta ttggaagcag tacgcaaagg ctttttttatt
901 tgataaaaat tagaagtata tttatttttt cataattaat ttatgaaaat gaaagggggt
961 gagcaaagtg acagaggaaa gcagtatctt atcaaataac aaggtattag caatatcatt
1021 attgacttta gcagtaaaca ttatgacttt tatagtgctt gtagctaagt agtacgaaag
1081 ggggagcttt aaaaagctcc ttggaataca tagaattcat aaattaattt atgaaaagaa
1141 gggcgtatat gaaaacttgt aaaaattgca aagagtttat taaagatact gaaatatgca
1201 aaatacattc gttgatgatt catgataaaa cagtagcaac ctattgcagt aaatacaatg
1261 agtcaagatg tttacataaa gggaaagtcc aatgtattaa ttgttcaaag atgaaccgat
1321 atggatggtg tgccataaaa atgagatgtt ttacagagga agaacagaaa aaagaacgta
1381 catgcattaa atattatgca aggagcttta aaaaagctca tgtaaagaag agtaaaaaga
1441 aaaaataatt tatttattaa tttaatattg agagtgccga cacagtatgc actaaaaaat
1501 atatctgtgg tgtagtgagc cgatacaaaa ggatagtcac tcgcattttc ataatacatc
1561 ttatgttatg attatgtgtc ggtgggactt cacgacgaaa acccacaata aaaaaagagt
1621 tcggggtagg gttaagcata gttgaggcaa ctaaacaatc aagctaggat atgcagtagc
1681 agaccgtaag gtcgttgttt aggtgtgttg taatacatac gctattaaga tgtaaaaata
1741 cggataccaa tgaagggaaa agtataattt ttggatgtag tttgtttgtt catctatggg
1801 caaactacgt ccaaagccgt ttccaaatct gctaaaaagt atatcctttc taaaatcaaa
1861 gtcaagtatg aaatcataaa taaagtttaa ttttgaagtt attatgatat tatgtttttc
1921 tattaaaata aattaagtat atagaatagt ttaataatag tatatactta atgtgataag
1981 tgtctgacag tgtcacagaa aggatgattg ttatggatta taagcggccg gccagtgggc
2041 aagttgaaaa attcacaaaa atgtggtata atatctttgt tcattagagc gataaacttg
2101 aatttgagag ggaacttaga tggtatttga aaaaattgat aaaaatagtt ggaacagaaa

2161 agagtatttt gaccactact ttgcaagtgt accttgtacc tacagcatga ccgttaaagt
2221 ggatatcaca caaataaagg aaaagggaat gaaactatat cctgcaatgc tttattatat
2281 tgcaatgatt gtaaaccgcc attcagagtt taggacggca atcaatcaag atggtgaatt
2341 ggggatatat gatgagatga taccaagcta tacaatattt cacaatgata ctgaaacatt
2401 ttccagcctt tggactgagt gtaagtctga ctttaaatca tttttagcag attatgaaag
2461 tgatacgcaa cggtatggaa acaatcatag aatggaagga aagccaaatg ctccggaaaa
2521 catttttaat gtatctatga taccgtggtc aaccttcgat ggctttaatc tgaatttgca
2581 gaaaggatat gattatttga ttcctatttt tactatgggg aaatattata aagaagataa
2641 caaaattata cttcctttgg caattcaagt tcatcacgca gtatgtgacg gatttcacat
2701 ttgccgtttt gtaaacgaat tgcaggaatt gataaatagt taacttcagg tttgtctgta
2761 actaaaaaca agtatttaag caaaaacatc gtagaaatac ggtgtttttt gttaccctaa
2821 gtttaaactc ctttttgata atctcatgac caaaatccct taacgtgagt tttcgttcca
2881 ctgagcgtca gaccccgtag aaaagatcaa aggatcttct tgagatcctt tttttctgcg
2941 cgtaatctgc tgcttgcaaa caaaaaaacc accgctacca gcggtggttt gtttgccgga
3001 tcaagagcta ccaactcttt ttccgaaggt aactggcttc agcagagcgc agataccaaa
3061 tactgttctt ctagtgtagc cgtagttagg ccaccacttc aagaactctg tagcaccgcc
3121 tacatacctc gctctgctaa tcctgttacc agtggctgct gccagtggcg ataagtcgtg
3181 tcttaccggg ttggactcaa gacgatagtt accggataag gcgcagcggt cgggctgaac
3241 ggggggttcg tgcacacagc ccagcttgga gcgaacgacc tacaccgaac tgagatacct
3301 acagcgtgag ctatgagaaa gcgccacgct tcccgaaggg agaaaggcgg acaggtatcc
3361 ggtaagcggc agggtcggaa caggagagcg cacgagggag cttccagggg gaaacgcctg
3421 gtatctttat agtcctgtcg ggtttcgcca cctctgactt gagcgtcgat ttttgtgatg
3481 ctcgtcaggg gggcggagcc tatggaaaaa cgccagcaac gcggcctttt tacggttcct
3541 ggccttttgc tggccttttg ctcacatgtt ctttcctgcg ttatcccctg attctgtgga
3601 taaccgtatt accgcctttg agtgagctga taccgctcgc cgcagccgaa cgaccgagcg
3661 cagcgagtca gtgagcgagg aagcggaaga gcgcccaata cgcagggccc cctgcttcgg
3721 ggtcattata gcgatttttt cggtatatcc atcctttttc gcacgatata caggattttg

3781 ccaaagggtt cgtgtagact ttccttggtg tatccaacgg cgtcagccgg gcaggatagg
3841 tgaagtaggc ccacccgcga gcgggtgttc cttcttcact gtcccttatt cgcacctggc
3901 ggtgctcaac gggaatcctg ctctgcgagg ctggccggct accgccggcg taacagatga
3961 gggcaagcgg atggctgatg aaaccaagcc aaccaggaag ggcagcccac ctatcaaggt
4021 gtactgcctt ccagacgaac gaagagcgat tgaggaaaag gcggcggcgg ccggcatgag
4081 cctgtcggcc tacctgctgg ccgtcggcca gggctacaaa atcacgggcg tcgtggacta
4141 tgagcacgtc cgcgagctgg cccgcatcaa tggcgacctg ggccgcctgg gcggcctgct
4201 gaaactctgg ctcaccgacg acccgcgcac ggcgcggttc ggtgatgcca cgatcctcgc
4261 cctgctggcg aagatcgaag agaagcagga cgagcttggc aaggtcatga tgggcgtggt
4321 ccgcccgagg gcagagccat gactttttta gccgctaaaa cggccggggg gtgcgcgtga
4381 ttgccaagca cgtccccatg cgctccatca agaagagcga cttcgcggag ctggtgaagt
4441 acatcaccga cgagcaaggc aagaccgatc gggccc
//

```
LOCUS       pMTV37                  4631 bp    DNA     circular UNA 02-
MAR-2020
DEFINITION  .
ACCESSION   urn.local...5j-exvbs3h
VERSION     urn.local...5j-exvbs3h
KEYWORDS    .
SOURCE
  ORGANISM  .
FEATURES             Location/Qualifiers
     misc_feature    1
                     /label="laczalpha"
     misc_feature    1
                     /label="clone before  this"
     misc_feature    1
                     /label="do not include in cloning"
     primer_bind     1..27
                     /Tm=75
                     /Sequence="AGGATCCTGCGGGTGCCAGGGCGTGCC"
                     /Description="<html><body></body></html>"
                     /label="oMTV206"
     misc_feature    4..23
                     /label="plasmid#1 F"
     misc_feature    8..52
                     /label="c31"
     protein_bind    13..46
                     /bound_moiety="phage œÜC31 integrase"
                     /note="minimal attB site for the œÜC31 integrase (<a
                     href=""http://www.ncbi.nlm.nih.gov/pubmed/10801973""

title=""http://www.ncbi.nlm.nih.gov/pubmed/10801973"">Grot
                     h et al., 2000</a>)"
                     /label="attB"
     terminator      115..361
                     /note="transcription terminator
<i>t</i><sub>L3</sub> from
                     phage Œª"
                     /label="Œª tL3 terminator"
     misc_feature    377..769
                     /label="lambda attP"
     primer_bind     complement(418..438)
                     /Tm=58
                     /Sequence="TTGTCATCAAACCTGTCGCAC"
                     /Description="<html><body></body></html>"
                     /label="oMTV29"
     protein_bind    485..718
                     /bound_moiety="Œª integrase"
                     /gene="<i>att</i>P"
                     /note="integrase from phage Œª"
                     /label="Œª attP"
     misc_feature    770..1206
                     /label="oriR6K"
     rep_origin      785..1173
                     /note="Œ≥ replication origin from <i>E. coli</i>
plasmid
```

```
                     R6K; requires the R6K initiator protein pi for
                     replication"
                     /label="R6K Œ≥ ori"
     primer_bind     complement(848..868)
                     /Tm=60
                     /Sequence="CAAGCCAGGGATGTAACGCAC"
                     /Description="<html><body></body></html>"
                     /label="oMTV28"
     misc_feature    complement(1260..2054)
                     /label="Kan"
     CDS             complement(1260..2054)
                     /codon_start=1
                     /gene="<i>aph(3')-II</i> (or <i>nptII</i>)"
                     /note="confers resistance to neomycin, kanamycin,
and G418
                     (Geneticin¬Æ)"
                     /product="aminoglycoside phosphotransferase from
Tn5"
                     /transl_table=1

/translation="MIEQDGLHAGSPAAWVERLFGYDWAQQTIGCSDAAVFRLSAQGR

PVLFVKTDLSGALNELQDEAARLSWLATTGVPCAAVLDVVTEAGRDWLLLGEVPGQDL

LSSHLAPAEKVSIMADAMRRLHTLDPATCPFDHQAKHRIERARTRMEAGLVDQDDLDE

EHQGLAPAELFARLKARMPDGEDLVVTHGDACLPNIMVENGRFSGFIDCGRLGVADRY
                     QDIALATRDIAEELGGEWADRFLVLYGIAAPDSQRIAFYRLLDEFF*"
                     /label="NeoR/KanR"
     primer_bind     complement(1277..1297)
                     /Tm=60
                     /Sequence="GCATCGCCTTCTATCGCCTTC"
                     /Description="<html><body></body></html>"
                     /label="oMTV26"
     primer_bind     1277..1297
                     /Tm=59
                     /Sequence="GAAGGCGATAGAAGGCGATGC"
                     /Description="<html><body></body></html>"
                     /label="oMTV27"
     primer_bind     complement(1415..1426)
                     /Tm=47
                     /Sequence="GCCGGCGGGGTG"
                     /Mismatch_Positions="======G====="
                     /Mismatches=1

/Extension="AAGTGGTATCGCTTTGGGGAACATGCCTTCAGCGGCGGGCGC"
                     /Description="<html><body></body></html>"
                     /label="oMTV17"
     primer_bind     complement(1733..1754)
                     /Tm=56
                     /Sequence="GATCTCCTGTCATCTCACCTTG"
                     /Description="<html><body></body></html>"
                     /label="oMTV24"
     primer_bind     1733..1754
```

```
                     /Tm=56
                     /Sequence="CAAGGTGAGATGACAGGAGATC"
                     /Description="<html><body></body></html>"
                     /label="oMTV25"
     misc_feature    1948..1965
                     /label="seq1_F"
     misc_feature    2180..2197
                     /label="seq6_F"
     primer_bind     complement(2245..2264)
                     /Tm=57
                     /Sequence="CCTGGCAGTTCCCTACTCTC"
                     /Description="<html><body></body></html>"
                     /label="oMTV22"
     primer_bind     2245..2264
                     /Tm=58
                     /Sequence="GAGAGTAGGGAACTGCCAGG"
                     /Description="<html><body></body></html>"
                     /label="oMTV23"
     terminator      2268..2354
                     /gene="<i>Escherichia coli rrnB</i>"
                     /note="transcription terminator T1 from the <i>E.
coli
                     rrnB</i> gene"
                     /label="rrnB T1 terminator"
     misc_feature    2315..1083
                     /note="prep 1"
                     /label="sequenced"
     misc_feature    2442..2459
                     /label="seq2_F"
     terminator      2446..2473
                     /note="transcription terminator T2 from the <i>E.
coli
                     rrnB</i> gene"
                     /label="rrnB T2 terminator"
     misc_feature    2502..2543
                     /label=31
     misc_feature    2556..2873
                     /note="Geneious type: promoter prokaryotic"
                     /note="/Description=araBp7"
                     /label="Promoter_P_1"
     promoter        2556..2873
                     /note="araBp7"
                     /note="/vntifkey=30"
                     /label="Promoter_P_1"
     promoter        2569..2853
                     /gene="<i>araBAD</i>"
                     /note="promoter of the L-arabinose operon of <i>E.
                     coli</i>; the <i>araC</i> regulatory gene is
transcribed
                     in the opposite direction (<a
                     href=""http://www.ncbi.nlm.nih.gov/pubmed/7608087""

title=""http://www.ncbi.nlm.nih.gov/pubmed/7608087"">Guzma
                     n et al., 1995</a>)"
```

```
                    /label="araBAD promoter"
     primer_bind    2806..2825
                    /Tm=59
                    /Sequence="CGGATCCTACCTGACGCTTT"
                    /Description="<html><body></body></html>"
                    /label="oMTV223"
     primer_bind    complement(2837..2860)
                    /Tm=58
                    /Sequence="AACGGGTATGGAGAAACAGTAGAG"
                    /Description="<html><body></body></html>"
                    /label="oMTV20"
     primer_bind    2837..2860
                    /Tm=59
                    /Sequence="CTCTACTGTTTCTCCATACCCGTT"
                    /Description="<html><body></body></html>"
                    /label="oMTV21"
     primer_bind    complement(2844..2881)
                    /Tm=65
                    /Sequence="ATGAATTCCTCCATCCAAAAAAACGGGTATGGAGAAAC"
                    /Description="<html><body></body></html>"
                    /label="oMTV1"
     primer_bind    complement(2851..2884)
                    /Tm=61
                    /Sequence="CATATGAATTCCTCCATCCAAAAAAACGGGTATG"
                    /Extension="TTGTGGTCGGTGCT"
                    /Description="<html><body></body></html>"
                    /label="oMTV12"
     primer_bind    complement(2851..2881)
                    /Tm=61
                    /Sequence="ATGAATTCCTCCATCCAAAAAAACGGGTATG"
                    /Description="<html><body></body></html>"
                    /label="oMTV205"
     primer_bind    complement(2855..2885)
                    /Tm=62
                    /Sequence="CCAGATGAATTCCTCCATCCAAAAAAACGGG"

/Mismatch_Positions="===G==========================="
                    /Mismatches=1
                    /Extension="TAGGCCTG"
                    /Description="<html><body></body></html>"
                    /label="oMTV3"
     primer_bind    2855..2908
                    /Tm=72

/Sequence="CCCGTTTTTTTGGATGGAGGAATTCATATGGAGGAAATCAGCGCGAA
                    GCCGTAC"
                    /Description="<html><body></body></html>"
                    /label="oMTV207"
     primer_bind    complement(2856..2884)
                    /Tm=59
                    /Sequence="CATATGAATTCCTCCATCCAAAAAAACGG"
                    /Extension="CTTTAATATCTTT"
                    /Description="<html><body></body></html>"
                    /label="oMTV59"
```

```
     primer_bind     complement(2856..2884)
                     /Tm=59
                     /Sequence="CATATGAATTCCTCCATCCAAAAAAACGG"
                     /Extension="CGGAT"
                     /Description="<html><body></body></html>"
                     /label="oMTV49"
     primer_bind     complement(2866..2881)
                     /Tm=47
                     /Sequence="ATGAATTCCTCCATCC"
                     /label="pLAR067+R4 (2950 R) - Paraburkholderia CDS
2912"
     misc_feature    2881
                     /label="clone after this"
     misc_feature    2882..2901
                     /label="Forward"
     CDS             2882..4402
                     /codon_start=1
                     /transl_table=1

/translation="MEEISAKPYDMIQSNIEKISQLFPNVITEIIDKNGKLKKVIDFQ

LLRQELSGEIVDGDFERYQLSWPGKRQAILLANTPTDKVLRVNKKESIDWENTGNLYI

EGDNLDVLKILQKSYMNKIKCIYIDPPYNTGKDFIYKDDFRMETSRYLEKTGRNFSDG

NELDGRFHSNWLTMMYPRLKLARNLLRDDGVIFVSIDNNELYNLQIIMNEIFGESNYV

ETFIWTKTATPPSLSNKSRKAAEYILCYEKNISGMKYFGSKLDNGDAPLLNSGNPIRV

LNFPKGSIRFTFLKEGRFNAGKYDRVELLKDFEVKNGTNNEEVLLKGEFKWTNEFMMN

EIQKGTYFIVKSPKFSVRFQRTDSEERYKPPANLLDIELNKNNGVGTNESAVKELEIL

GMKGYFDYPKPLSLIKKILNMVIKNDKDAIILDFFSGSATTAHAVMELNASDNGRRKY

IMVQKPEPISEDCIAYKAGYRDICQIGKERILRASKIIKEQTGADIDYGVRVYKLE*"
                     /label="Ccla_2133"
     primer_bind     complement(3035..3055)
                     /Tm=60
                     /Sequence="ATCGCCGTCCACAATTTCACC"
                     /Description="<html><body></body></html>"
                     /label="oMTV228"
     primer_bind     3035..3055
                     /Tm=60
                     /Sequence="GGTGAAATTGTGGACGGCGAT"
                     /Description="<html><body></body></html>"
                     /label="oMTV229"
     primer_bind     3511..3534
                     /Tm=59
                     /Sequence="GATCTTCGGCGAAAGCAACTATGT"
                     /Description="<html><body></body></html>"
                     /label="oMTV227"
     primer_bind     complement(3823..3847)
                     /Tm=60
```

```
                     /Sequence="CTTCAGCAGAACTTCCTCGTTGTTG"
                     /Description="<html><body></body></html>"
                     /label="oMTV226"
     primer_bind     4048..4069
                     /Tm=58
                     /Sequence="GGAAATTCTGGGTATGAAGGGC"
                     /Description="<html><body></body></html>"
                     /label="oMTV225"
     primer_bind     complement(4370..4389)
                     /Tm=58
                     /Sequence="TAAACACGCACGCCGTAATC"
                     /Description="<html><body></body></html>"
                     /label="oMTV224"
     misc_feature    4403..4472
                     /label="R4 attP"
     misc_feature    4403
                     /label="clone before this"
     primer_bind     4403..4431
                     /Tm=66
                     /Sequence="AGGCATGTTCCCCAAAGCGATACCACTTG"
                     /Description="<html><body></body></html>"
                     /label="oMTV717"
     primer_bind     complement(4604..27)
                     /Tm=77

/Sequence="GGCACGCCCTGGCACCCGCAGGATCCTAACAGCTATGACCATGATTA
                     CGCCAAGC"
                     /Description="<html><body></body></html>"
                     /label="oMTV208"
     misc_feature    4612..4631
                     /label="Reverse"
ORIGIN
        1 aggatcctgc gggtgccagg gcgtgccctt gggctccccg ggcgcgtact
ccacctcact
       61 cgagaattct catgtttgac agcttatcac tgatcagtga attaatggcg
atgacgcatc
      121 ctcacgataa tatccgggta ggcgcaatca ctttcgtctc tactccgtta
caaagcgagg
      181 ctgggtattt cccggccttt ctgttatccg aaatccactg aaagcacagc
ggctggctga
      241 ggagataaat aataaacgag ggctgtatg cacaaagcat cttctgttga
gttaagaacg
      301 agtatcgaga tggcacatag ccttgctcaa attggaatca ggtttgtgcc
aataccagta
      361 gaaacagacg aagaagctag ctttgcactg gattgcgagg ctttgtgctt
ctctggagtg
      421 cgacaggttt gatgacaaaa aattagcgca agaagacaaa aatcaccttg
cgctaatgct
      481 ctgttacagg tcactaatac catctaagta gttgattcat agtgactgca
tatatgttgt
      541 gttttacagt attatgtagt ctgtttttta tgcaaaatct aatttaatat
attgatattt
      601 atatcatttt acgtttctcg ttcagctttt ttatactaag ttggcattat
aaaaaagcat
```

661 tgcttatcaa tttgttgcaa cgaacaggtc actatcagtc aaaataaaat cattatttga
721 tttcaatttt gtcccactcc ctgcctctgt catcacgata ctgtgatgcc atggctaatt
781 cccatgtcag ccgttaagtg ttcctgtgtc actcaaaatt gctttgagag gctctaaggg
841 cttctcagtg cgttacatcc ctggcttgtt gtccacaacc gttaaacctt aaaagcttta
901 aaagccttat atattctttt ttttcttata aaacttaaaa ccttagaggc tatttaagtt
961 gctgatttat attaatttta ttgttcaaac atgagagctt agtacgtgaa acatgagagc
1021 ttagtacgtt agccatgaga gcttagtacg ttagccatga gggtttagtt cgttaaacat
1081 gagagcttag tacgttaaac atgagagctt agtacgtgaa acatgagagc ttagtacgta
1141 ctatcaacag gttgaactgc tgatcttcag atcctctacg ccggacgcat cgtggccgga
1201 tcttgcggcc gcaaaaatta aaaatgaagt tttgacggta tcgaacccca gagtcccgct
1261 cagaagaact cgtcaagaag gcgatagaag gcgatgcgct gcgaatcggg agcggcgata
1321 ccgtaaagca cgaggaagcg gtcagcccat tcgccgccaa gctcttcagc aatatcacgg
1381 gtagccaacg ctatgtcctg atagcggtcc gccacaccca gccggccaca gtcgatgaat
1441 ccagaaaagc ggccattttc caccatgata ttcggcaagc aggcatcgcc atgggtcacg
1501 acgagatcct cgccgtcggg catccgcgcc ttgagcctgg cgaacagttc ggctggcgcg
1561 agcccctgat gctcttcgtc cagatcatcc tgatcgacaa gaccggcttc catccgagta
1621 cgtgctcgct cgatgcgatg tttcgcttgg tggtcgaatg ggcaggtagc cggatcaagc
1681 gtatgcagcc gccgcattgc atcagccatg atggatactt tctcggcagg agcaaggtga
1741 gatgacagga gatcctgccc cggcacttcg cccaatagca gccagtccct tcccgcttca
1801 gtgacaacgt cgagcacagc tgcgcaagga acgcccgtcg tggccagcca cgatagccgc
1861 gctgcctcgt cttggagttc attcagggca ccggacaggt cggtcttgac aaaaagaacc
1921 gggcgcccct gcgctgacag ccggaacacg gcggcatcag agcagccgat tgtctgttgt
1981 gcccagtcat agccgaatag cctctccacc caagcggccg gagaacctgc gtgcaatcca
2041 tcttgttcaa tcatgcgaaa cgatcctcat cctgtctctt gatccactag attattgaag
2101 catttatcag ggttattgtc tcatgagcgg atacatattt gaatgtattt agaaaaataa
2161 acaaataggg gttccgcgca catttccccg aaaagtgcca cctgcatcga tggcccccga
2221 tggtagtgtg gggtctcccc atgcgagagt agggaactgc caggcatcaa ataaaacgaa

2281 aggctcagtc gaaagactgg gcctttcgtt ttatctgttg tttgtcggtg aacgctctcc
2341 tgagtaggac aaatccgccg ggagcggatt tgaacgttgc gaagcaacgg cccggagggt
2401 ggcgggcagg acgcccgcca taaactgcca ggcatcaaat taagcagaag gccatcctga
2461 cggatggcct ttttgcgtgg ccagtgccaa gcttgcatgc cgtgccccaa ctggggtaac
2521 ctttgagttc tctcagttgg gggcgtaggg tctagccgcc attcagagaa gaaaccaatt
2581 gtccatattg catcagacat tgccgtcact gcgtctttta ctggctcttc tcgctaacca
2641 aaccggtaac cccgcttatt aaaagcattc tgtaacaaag cgggaccaag gccatgacaa
2701 aaacgcgtag caaaagtgtc tataatcacg gcagaaaagt ccacattgat tatttgcacg
2761 gcgtcacact ttgctatgcc atagcatttt tatccataag attagcggat cctacctgac
2821 gctttttatc gcaactctct actgtttctc catacccgtt tttttggatg gaggaattca
2881 tatggaggaa atcagcgcga agccgtacga catgattcag agcaacatcg agaaaattag
2941 ccaactgttc ccgaacgtga tcaccgaaat cattgacaag aacggtaaac tgaagaaagt
3001 tatcgatttc cagctgctgc gtcaagagct gagcggtgaa attgtggacg gcgattttga
3061 gcgttatcag ctgagctggc cgggcaaacg tcaagcgatc ctgctggcga acaccccgac
3121 cgacaaggtg ctgcgtgtta acaagaaaga gagcatcgat tgggaaaaca ccggtaacct
3181 gtacattgaa ggcgacaacc tggatgttct gaagatcctg cagaaaagct acatgaacaa
3241 gatcaagtgc atctacatcg acccgccgta taacaccggc aaggatttca tctacaaaga
3301 cgattttcgt atggagacca gccgttatct ggaaaaaacc ggccgtaact tcagcgacgg
3361 taacgagctg gatggccgtt ttcacagcaa ctggctgacc atgatgtacc cgcgtctgaa
3421 gctggcgcgt aacctgctgc gtgacgatgg tgtgatcttc gttagcattg ataacaacga
3481 gctgtacaac ctgcaaatca ttatgaacga gatcttcggc gaaagcaact atgtggaaac
3541 ctttatttgg accaaaaccg cgaccccgcc gagcctgagc aacaagagcc gtaaagcggc
3601 ggagtacatc ctgtgctatg aaaagaacat tagcggtatg aagtacttcg gcagcaaact
3661 ggacaacggt gatgcgccgc tgctgaacag cggcaacccg atccgtgtgc tgaactttcc
3721 gaaaggtagc attcgtttca cctttctgaa agagggtcgt ttcaacgcgg gcaaatatga
3781 ccgtgtggag ctgctgaagg attttgaagt taaaaacggt accaacaacg aggaagttct
3841 gctgaagggc gaattcaaat ggaccaacga gtttatgatg aacgaaatcc agaagggtac

3901 ctacttcatt gtgaagagcc cgaaattcag cgttcgtttt caacgtaccg acagcgagga
3961 acgttataag ccgccggcga acctgctgga tatcgagctg aacaaaaaca acggtgtggg
4021 caccaacgaa agcgcggtta aggagctgga aattctgggt atgaagggct actttgacta
4081 tccgaaaccg ctgagcctga tcaagaaaat tctgaacatg gttatcaaga acgacaaaga
4141 tgcgatcatt ctggacttct ttagcggtag cgcgaccacc gcgcatgcgg tgatggagct
4201 gaacgcgagc gataacggcc gtcgtaagta catcatggtt cagaaaccgg agccgatcag
4261 cgaagactgc attgcgtaca aggcgggtta tcgtgatatc tgccagattg gcaaagagcg
4321 tatcctgcgt gcgagcaaga tcattaaaga acaaaccggt gcggacattg attacggcgt
4381 gcgtgtttat aaactggaat aaaggcatgt tccccaaagc gataccactt gaagcagtgg
4441 tactgcttgt gggtacactc tgcgggtgat gatcgcgata atgcatgagt cagtactcca
4501 gaagacaatt gatctctgaa agctgacatg tggatcctgc gggtgccagg gcgtgccctt
4561 ggatcggatc ccgggcccgt cgactgcaga ggcctgcatg caagcttggc gtaatcatgg
4621 tcatagctgt t
//

```
LOCUS       pMTV38                  5842 bp    DNA     circular UNA 28-
APR-2020
DEFINITION  .
ACCESSION   urn.local...5m-exvbs3j
VERSION     urn.local...5m-exvbs3j
KEYWORDS    .
SOURCE
  ORGANISM  .
FEATURES             Location/Qualifiers
     misc_feature    1
                     /label="laczalpha"
     misc_feature    1
                     /label="clone before  this"
     misc_feature    1
                     /label="do not include in cloning"
     primer_bind     1..27
                     /Tm=75
                     /Sequence="AGGATCCTGCGGGTGCCAGGGCGTGCC"
                     /Description="<html><body></body></html>"
                     /label="oMTV206"
     misc_feature    4..23
                     /label="plasmid#1 F"
     misc_feature    8..52
                     /label="c31"
     protein_bind    13..46
                     /bound_moiety="phage œÜC31 integrase"
                     /note="minimal attB site for the œÜC31 integrase (<a
                     href=""http://www.ncbi.nlm.nih.gov/pubmed/10801973""

title=""http://www.ncbi.nlm.nih.gov/pubmed/10801973"">Grot
                     h et al., 2000</a>)"
                     /label="attB"
     terminator      115..361
                     /note="transcription terminator
<i>t</i><sub>L3</sub> from
                     phage Œª"
                     /label="Œª tL3 terminator"
     misc_feature    377..769
                     /label="lambda attP"
     primer_bind     complement(418..438)
                     /Tm=58
                     /Sequence="TTGTCATCAAACCTGTCGCAC"
                     /Description="<html><body></body></html>"
                     /label="oMTV29"
     protein_bind    485..718
                     /bound_moiety="Œª integrase"
                     /gene="<i>att</i>P"
                     /note="integrase from phage Œª"
                     /label="Œª attP"
     misc_feature    770..1206
                     /label="oriR6K"
     rep_origin      785..1173
                     /note="Œ≥ replication origin from <i>E. coli</i>
plasmid
```

```
                     R6K; requires the R6K initiator protein pi for
                     replication"
                     /label="R6K Œ≥ ori"
     primer_bind     complement(848..868)
                     /Tm=60
                     /Sequence="CAAGCCAGGGATGTAACGCAC"
                     /Description="<html><body></body></html>"
                     /label="oMTV28"
     misc_feature    complement(1260..2054)
                     /label="Kan"
     CDS             complement(1260..2054)
                     /codon_start=1
                     /gene="<i>aph(3')-II</i> (or <i>nptII</i>)"
                     /note="confers resistance to neomycin, kanamycin,
and G418
                     (Geneticin¬Æ)"
                     /product="aminoglycoside phosphotransferase from
Tn5"
                     /transl_table=1

/translation="MIEQDGLHAGSPAAWVERLFGYDWAQQTIGCSDAAVFRLSAQGR

PVLFVKTDLSGALNELQDEAARLSWLATTGVPCAAVLDVVTEAGRDWLLLGEVPGQDL

LSSHLAPAEKVSIMADAMRRLHTLDPATCPFDHQAKHRIERARTRMEAGLVDQDDLDE

EHQGLAPAELFARLKARMPDGEDLVVTHGDACLPNIMVENGRFSGFIDCGRLGVADRY
                     QDIALATRDIAEELGGEWADRFLVLYGIAAPDSQRIAFYRLLDEFF*"
                     /label="NeoR/KanR"
     primer_bind     complement(1277..1297)
                     /Tm=60
                     /Sequence="GCATCGCCTTCTATCGCCTTC"
                     /Description="<html><body></body></html>"
                     /label="oMTV26"
     primer_bind     1277..1297
                     /Tm=59
                     /Sequence="GAAGGCGATAGAAGGCGATGC"
                     /Description="<html><body></body></html>"
                     /label="oMTV27"
     primer_bind     complement(1415..1426)
                     /Tm=47
                     /Sequence="GCCGGCGGGGTG"
                     /Mismatch_Positions="======G====="
                     /Mismatches=1

/Extension="AAGTGGTATCGCTTTGGGGAACATGCCTTCAGCGGCGGGCGC"
                     /Description="<html><body></body></html>"
                     /label="oMTV17"
     primer_bind     complement(1733..1754)
                     /Tm=56
                     /Sequence="GATCTCCTGTCATCTCACCTTG"
                     /Description="<html><body></body></html>"
                     /label="oMTV24"
     primer_bind     1733..1754
```

```
                     /Tm=56
                     /Sequence="CAAGGTGAGATGACAGGAGATC"
                     /Description="<html><body></body></html>"
                     /label="oMTV25"
     misc_feature    1948..1965
                     /label="seq1_F"
     misc_feature    2180..2197
                     /label="seq6_F"
     primer_bind     complement(2245..2264)
                     /Tm=57
                     /Sequence="CCTGGCAGTTCCCTACTCTC"
                     /Description="<html><body></body></html>"
                     /label="oMTV22"
     primer_bind     2245..2264
                     /Tm=58
                     /Sequence="GAGAGTAGGGAACTGCCAGG"
                     /Description="<html><body></body></html>"
                     /label="oMTV23"
     terminator      2268..2354
                     /gene="<i>Escherichia coli rrnB</i>"
                     /note="transcription terminator T1 from the <i>E.
coli
                     rrnB</i> gene"
                     /label="rrnB T1 terminator"
     misc_feature    2299..24
                     /note="prep 1"
                     /label="sequenced "
     misc_feature    2442..2459
                     /label="seq2_F"
     terminator      2446..2473
                     /note="transcription terminator T2 from the <i>E.
coli
                     rrnB</i> gene"
                     /label="rrnB T2 terminator"
     misc_feature    2502..2543
                     /label=31
     misc_feature    2556..2873
                     /note="Geneious type: promoter prokaryotic"
                     /note="/Description=araBp7"
                     /label="Promoter_P_1"
     promoter        2556..2873
                     /note="araBp7"
                     /note="/vntifkey=30"
                     /label="Promoter_P_1"
     promoter        2569..2853
                     /gene="<i>araBAD</i>"
                     /note="promoter of the L-arabinose operon of <i>E.
                     coli</i>; the <i>araC</i> regulatory gene is
transcribed
                     in the opposite direction (<a
                     href=""http://www.ncbi.nlm.nih.gov/pubmed/7608087""

title=""http://www.ncbi.nlm.nih.gov/pubmed/7608087"">Guzma
                     n et al., 1995</a>)"
```

```
                     /label="araBAD promoter"
     primer_bind     2806..2825
                     /Tm=59
                     /Sequence="CGGATCCTACCTGACGCTTT"
                     /Description="<html><body></body></html>"
                     /label="oMTV223"
     primer_bind     complement(2837..2860)
                     /Tm=58
                     /Sequence="AACGGGTATGGAGAAACAGTAGAG"
                     /Description="<html><body></body></html>"
                     /label="oMTV20"
     primer_bind     2837..2860
                     /Tm=59
                     /Sequence="CTCTACTGTTTCTCCATACCCGTT"
                     /Description="<html><body></body></html>"
                     /label="oMTV21"
     primer_bind     complement(2844..2881)
                     /Tm=65
                     /Sequence="ATGAATTCCTCCATCCAAAAAAACGGGTATGGAGAAAC"
                     /Description="<html><body></body></html>"
                     /label="oMTV1"
     primer_bind     complement(2851..2885)
                     /Tm=62
                     /Sequence="TCATATGAATTCCTCCATCCAAAAAAACGGGTATG"
                     /Extension="TTGTGGTCGGTGC"
                     /Description="<html><body></body></html>"
                     /label="oMTV12"
     primer_bind     complement(2851..2881)
                     /Tm=61
                     /Sequence="ATGAATTCCTCCATCCAAAAAAACGGGTATG"
                     /Description="<html><body></body></html>"
                     /label="oMTV205"
     primer_bind     complement(2855..2884)
                     /Tm=60
                     /Sequence="CAGATGAATTCCTCCATCCAAAAAAACGGG"
                     /Mismatch_Positions="==G==========================="
                     /Mismatches=1
                     /Extension="TAGGCCTGC"
                     /Description="<html><body></body></html>"
                     /label="oMTV3"
     primer_bind     2855..2908
                     /Tm=70

/Sequence="CCCGTTTTTTTGGATGGAGGAATTCATATGACCAAATGGAACAACGT
                     GAGCCTG"
                     /Description="<html><body></body></html>"
                     /label="oMTV209"
     primer_bind     complement(2856..2885)
                     /Tm=60
                     /Sequence="TCATATGAATTCCTCCATCCAAAAAAACGG"
                     /Extension="CTTTAATATCTT"
                     /Description="<html><body></body></html>"
                     /label="oMTV59"
     primer_bind     complement(2856..2885)
```

```
                     /Tm=60
                     /Sequence="TCATATGAATTCCTCCATCCAAAAAAACGG"
                     /Extension="CGGA"
                     /Description="<html><body></body></html>"
                     /label="oMTV49"
     primer_bind     complement(2866..2881)
                     /Tm=47
                     /Sequence="ATGAATTCCTCCATCC"
                     /label="pLAR067+R4 (2950 R) - Paraburkholderia CDS
2912"
     misc_feature    2881
                     /label="clone after this"
     misc_feature    2882..5772
                     /label="Ccla2368-9PhiK38"
     misc_feature    2882..2901
                     /label="Forward"
     CDS             2882..4195
                     /codon_start=1
                     /transl_table=1

/translation="MTKWNNVSLGELLTESNIPSENPDPSKRITVRLNVEGIEKRPFR

EETKGATKYYIRKAGQFIYGKQNLHKGAFGIIPEELDGYESTIDLPAFDIDKSKVLPE

WLMLILTKDNFYKELESIASGSATKRIHPERLFQVKIPLPSISEQKDILKKYDMYKVY

HTAISNIIDDARIYIDKLRQSILQEAVQGKLVPQDPNDEPASVLLERIKEEKERLIKE

GKIKKEKPLPPISEDEIPYELPEGWEWVRLGEIAIINPRNDIDDDVEVSFIPMKLIED

GFSNKHTSEIKKWKDVKNGFTHFQEGDVVIAKITPCFQNRKSAVMRNLKNGYGAGTTE

LYVVRTYANTVLPEYLLALFKTEAFIKGGVATYTGTAGQQRVKKDYIENLLLPLPPLN
                     EQKRIVEKVDQLMALCDELEKNIEQSKKDCELLIQSVLQEAFKEA*"
                     /label="Ccla_2368"
     primer_bind     complement(3281..3303)
                     /Tm=59
                     /Sequence="AGCGGGATCTTCACTTGAAACAG"
                     /Description="<html><body></body></html>"
                     /label="oMTV239"
     primer_bind     3464..3483
                     /Tm=59
                     /Sequence="GATCCGAACGATGAACCGGC"
                     /Description="<html><body></body></html>"
                     /label="oMTV238"
     primer_bind     complement(3694..3713)
                     /Tm=57
                     /Sequence="AACCGTCCTCAATCAGCTTC"
                     /Description="<html><body></body></html>"
                     /label="oMTV237"
     primer_bind     3943..3962
                     /Tm=57
                     /Sequence="CAAGACCGAGGCGTTTATCA"
                     /Description="<html><body></body></html>"
```

```
                     /label="oMTV236"
     CDS             4212..5651
                     /codon_start=1
                     /transl_table=1

/translation="MSISATIKAVQDIMRQDAGVDGDAQRISQLVWMIFLKVFDAKEE

EWELMDDDYTPIIPEGLRWRDWAADDEGITGDELLDFVNNKLFKELKEMKLDENSNPK

AFIVKAVFEDSYNYMKSGTLMRQVINKLNEIDFTTQKDRHLFNDIYESILRDLQSAGN

AGEYYTPRPVTQFMVDMVNPQLGEKVLDFACGTGGFLVCALEHLKKQVKNIEDEKTLQ

ETILGIEKKPLPYMLAVTNLILHDIDVPKIRHDNSLARNVRDYKPVDKVDIIVTNPPF

GGIEEDGILVNFPQQFQTKETADLFMVLLMYLLKDTGRAAIVLPDGFLFGEGVKTTIK

EKLLEEFNLHTIVRLPNGVFAPYTDINTNLLFLEKGKPTKEIWFFEHPLPEGYKKYTK

TKPIRHEEFELEKQWWNNREEKEYAWKVSIEEIKNRNYNLDIKNPNNNRSNELESTEK
                     IIEKLDYNFKKLSLILNEIKDSLFREGVN*"
                     /label="Ccla_2369"
     primer_bind     complement(4355..4374)
                     /Tm=56
                     /Sequence="GGGTGTAGTCATCGTCCATC"
                     /Description="<html><body></body></html>"
                     /label="oMTV235"
     primer_bind     4588..4611
                     /Tm=56
                     /Sequence="TTATCAACAAGCTGAACGAGATTG"
                     /Description="<html><body></body></html>"
                     /label="oMTV234"
     primer_bind     4916..4936
                     /Tm=62
                     /Sequence="GACCAACCTGATCCTGCACGA"
                     /Description="<html><body></body></html>"
                     /label="oMTV233"
     primer_bind     complement(5108..5130)
                     /Tm=60
                     /Sequence="ACATCAGCAGAACCATGAACAGG"
                     /Description="<html><body></body></html>"
                     /label="oMTV232"
     primer_bind     5473..5494
                     /Tm=56
                     /Sequence="GCATCGAAGAGATTAAGAACCG"
                     /Description="<html><body></body></html>"
                     /label="oMTV231"
     primer_bind     complement(5622..5644)
                     /Tm=59
                     /Sequence="ACACCTTCACGAAACAGGCTATC"
                     /Description="<html><body></body></html>"
                     /label="oMTV230"
     primer_bind     complement(5816..27)
                     /Tm=77
```

```
/Sequence="GGCACGCCCTGGCACCCGCAGGATCCTAACAGCTATGACCATGATTA
                     CGCCAAG"
                     /Description="<html><body></body></html>"
                     /label="oMTV210"
     misc_feature    5823..5842
                     /label="Reverse"
ORIGIN
        1 aggatcctgc gggtgccagg gcgtgccctt gggctccccg ggcgcgtact
ccacctcact
       61 cgagaattct catgtttgac agcttatcac tgatcagtga attaatggcg
atgacgcatc
      121 ctcacgataa tatccgggta ggcgcaatca ctttcgtctc tactccgtta
caaagcgagg
      181 ctgggtattt cccggccttt ctgttatccg aaatccactg aaagcacagc
ggctggctga
      241 ggagataaat aataaacgag gggctgtatg cacaaagcat cttctgttga
gttaagaacg
      301 agtatcgaga tggcacatag ccttgctcaa attggaatca ggtttgtgcc
aataccagta
      361 gaaacagacg aagaagctag ctttgcactg gattgcgagg ctttgtgctt
ctctggagtg
      421 cgacaggttt gatgacaaaa aattagcgca agaagacaaa aatcaccttg
cgctaatgct
      481 ctgttacagg tcactaatac catctaagta gttgattcat agtgactgca
tatatgttgt
      541 gttttacagt attatgtagt ctgtttttta tgcaaaatct aatttaatat
attgatattt
      601 atatcatttt acgtttctcg ttcagctttt ttatactaag ttggcattat
aaaaaagcat
      661 tgcttatcaa tttgttgcaa cgaacaggtc actatcagtc aaaataaaat
cattatttga
      721 tttcaatttt gtcccactcc ctgcctctgt catcacgata ctgtgatgcc
atggctaatt
      781 cccatgtcag ccgttaagtg ttcctgtgtc actcaaaatt gctttgagag
gctctaaggg
      841 cttctcagtg cgttacatcc ctggcttgtt gtccacaacc gttaaacctt
aaaagcttta
      901 aaagccttat atattctttt ttttcttata aaacttaaaa ccttagaggc
tatttaagtt
      961 gctgatttat attaatttta ttgttcaaac atgagagctt agtacgtgaa
acatgagagc
     1021 ttagtacgtt agccatgaga gcttagtacg ttagccatga gggtttagtt
cgttaaacat
     1081 gagagcttag tacgttaaac atgagagctt agtacgtgaa acatgagagc
ttagtacgta
     1141 ctatcaacag gttgaactgc tgatcttcag atcctctacg ccggacgcat
cgtggccgga
     1201 tcttgcggcc gcaaaaatta aaaatgaagt tttgacggta tcgaacccca
gagtcccgct
     1261 cagaagaact cgtcaagaag gcgatagaag gcgatgcgct gcgaatcggg
agcggcgata
     1321 ccgtaaagca cgaggaagcg gtcagcccat tcgccgccaa gctcttcagc
aatatcacgg
```

1381 gtagccaacg ctatgtcctg atagcggtcc gccacaccca gccggccaca gtcgatgaat
1441 ccagaaaagc ggccattttc caccatgata ttcggcaagc aggcatcgcc atgggtcacg
1501 acgagatcct cgccgtcggg catccgcgcc ttgagcctgg cgaacagttc ggctggcgcg
1561 agcccctgat gctcttcgtc cagatcatcc tgatcgacaa gaccggcttc catccgagta
1621 cgtgctcgct cgatgcgatg tttcgcttgg tggtcgaatg ggcaggtagc cggatcaagc
1681 gtatgcagcc gccgcattgc atcagccatg atggatactt tctcggcagg agcaaggtga
1741 gatgacagga gatcctgccc cggcacttcg cccaatagca gccagtccct tcccgcttca
1801 gtgacaacgt cgagcacagc tgcgcaagga acgcccgtcg tggccagcca cgatagccgc
1861 gctgcctcgt cttggagttc attcagggca ccggacaggt cggtcttgac aaaaagaacc
1921 gggcgcccct gcgctgacag ccggaacacg gcggcatcag agcagccgat tgtctgttgt
1981 gcccagtcat agccgaatag cctctccacc caagcggccg gagaacctgc gtgcaatcca
2041 tcttgttcaa tcatgcgaaa cgatcctcat cctgtctctt gatccactag attattgaag
2101 catttatcag ggttattgtc tcatgagcgg atacatattt gaatgtattt agaaaaataa
2161 acaaataggg gttccgcgca catttccccg aaaagtgcca cctgcatcga tggcccccga
2221 tggtagtgtg gggtctcccc atgcgagagt agggaactgc caggcatcaa ataaaacgaa
2281 aggctcagtc gaaagactgg gcctttcgtt ttatctgttg tttgtcggtg aacgctctcc
2341 tgagtaggac aaatccgccg ggagcggatt tgaacgttgc gaagcaacgg cccggagggt
2401 ggcgggcagg acgcccgcca taaactgcca ggcatcaaat taagcagaag gccatcctga
2461 cggatggcct ttttgcgtgg ccagtgccaa gcttgcatgc cgtgccccaa ctggggtaac
2521 ctttgagttc tctcagttgg gggcgtaggg tctagccgcc attcagagaa gaaaccaatt
2581 gtccatattg catcagacat tgccgtcact gcgtctttta ctggctcttc tcgctaacca
2641 aaccggtaac cccgcttatt aaaagcattc tgtaacaaag cgggaccaag gccatgacaa
2701 aaacgcgtag caaaagtgtc tataatcacg gcagaaaagt ccacattgat tatttgcacg
2761 gcgtcacact ttgctatgcc atagcatttt tatccataag attagcggat cctacctgac
2821 gctttttatc gcaactctct actgtttctc catacccgtt tttttggatg gaggaattca
2881 tatgaccaaa tggaacaacg tgagcctggg cgaactgctg accgagagca acatcccgag
2941 cgagaacccg gacccgagca aacgtatcac cgtgcgtctg aacgttgagg gcattgaaaa

3001 gcgtccgttc cgtgaggaaa ccaagggtgc gaccaaatac tatatccgta aggcgggcca
3061 attcatttat ggcaagcaga acctgcacaa aggcgcgttt ggtatcattc cggaggaact
3121 ggatggctac gagagcacca tcgacctgcc ggcgttcgat attgacaaga gcaaagttct
3181 gccggaatgg ctgatgctga tcctgaccaa ggacaacttt tataaagagc tggaaagcat
3241 cgcgagcggt agcgcgacca aacgtattca cccggagcgt ctgtttcaag tgaagatccc
3301 gctgccgagc attagcgaac agaaagatat cctgaagaaa tacgacatgt ataaggttta
3361 ccacaccgcg attagcaaca tcattgacga tgcgcgtatc tacattgata aactgcgtca
3421 gagcatcctg caagaagcgg tgcagggcaa gctggttccg caagatccga acgatgaacc
3481 ggcgagcgtg ctgctggagc gtattaaaga ggagaaagaa cgtctgatca aagagggtaa
3541 aattaagaaa gagaagccgc tgccgccgat cagcgaggac gaaattccgt acgagctgcc
3601 ggaaggctgg gagtgggttc gtctgggtga aatcgcgatc attaacccgc gtaacgatat
3661 tgacgatgac gtggaagtta gcttcatccc gatgaagctg attgaggacg gttttagcaa
3721 caaacacacc agcgaaatca agaaatggaa ggatgtgaaa aacggcttca cccactttca
3781 agagggtgac gtggttatcg cgaagattac cccgtgcttc cagaaccgta agagcgcggt
3841 gatgcgtaac ctgaaaaacg gttatggcgc gggtaccacc gaactgtatg tggttcgtac
3901 ctacgcgaac accgttctgc cggaatacct gctggcgctg ttcaagaccg aggcgtttat
3961 caaaggtggc gtggcgacct ataccggtac cgcgggtcag caacgtgtta agaaagatta
4021 cattgagaac ctgctgctgc cgctgccgcc gctgaacgaa caaaagcgta ttgtggagaa
4081 agttgatcag ctgatggcgc tgtgcgacga gctggagaag aacatcgaac agagcaagaa
4141 agactgcgag ctgctgattc aaagcgttct gcaggaagcg tttaaagagg cgtaaatagg
4201 aggtgttcaa tatgagcatc agcgcgacca ttaaggcggt gcaggatatc atgcgtcaag
4261 acgcgggtgt tgatggcgac gcgcagcgta tcagccaact ggtgtggatg attttcctga
4321 aggtttttga cgcgaaagag gaagagtggg agctgatgga cgatgactac accccgatca
4381 ttccggaagg tctgcgttgg cgtgattggg cggcggatga tgagggtatc accggtgatg
4441 aactgctgga cttcgtgaac aacaagctgt ttaaagaact gaaggagatg aaactggacg
4501 agaacagcaa cccgaaggcg ttcattgtga aagcggtttt cgaagatagc tacaactaca
4561 tgaagagcgg caccctgatg cgtcaggtta tcaacaagct gaacgagatt gatttcacca

4621 cccaaaaaga ccgtcacctg tttaacgata tctatgagag cattctgcgt
gacctgcaga
4681 gcgcgggtaa cgcgggcgaa tactataccc cgcgtccggt gacccagttc
atggtggaca
4741 tggttaaccc gcaactgggc gagaaggttc tggatttcgc gtgcggtacc
ggtggctttc
4801 tggtgtgcgc gctggaacac ctgaagaaac aggttaagaa catcgaagat
gagaaaaccc
4861 tgcaagagac catcctgggt attgaaaaga aaccgctgcc gtacatgctg
gcggtgacca
4921 acctgatcct gcacgatatt gacgttccga aaattcgtca cgacaacagc
ctggcgcgta
4981 acgtgcgtga ttataagccg gtggataaag ttgacatcat tgttaccaac
ccgccgttcg
5041 gtggcatcga agaggacggc attctggtga acttcccgca gcaatttcag
accaaggaaa
5101 ccgcggacct gttcatggtt ctgctgatgt acctgctgaa agataccggt
cgtgcggcga
5161 ttgtgctgcc ggatggcttc ctgtttggcg agggcgttaa gaccaccatc
aaggaaaaac
5221 tgctggaaga gttcaacctg cacaccattg tgcgtctgcc gaacggtgtt
ttcgcgccgt
5281 ataccgacat caacaccaac ctgctgtttc tggagaaggg taaaccgacc
aaggaaattt
5341 ggttctttga gcacccgctg ccggaaggct acaagaaata taccaagacc
aaaccgatcc
5401 gtcacgaaga gtttgaactg gagaaacaat ggtggaacaa ccgtgaagag
aaggaatacg
5461 cgtggaaagt gagcatcgaa gagattaaga accgtaacta taacctggac
atcaaaaacc
5521 cgaacaacaa ccgtagcaac gaactggaga gcaccgaaaa gatcattgag
aaactggact
5581 acaacttcaa gaaactgagc ctgatcctga acgagattaa agatagcctg
tttcgtgaag
5641 gtgttaacta accctaatac gcaagtcgat aactctcctg ggagcgttga
caacttgcgc
5701 accctgagac atgttgaaag ctcagaagat cgcgatacaa ttgatctcat
gcatgagtca
5761 gtactcggat cctatcggat cccgggcccg tcgactgcag aggcctgcat
gcaagcttgg
5821 cgtaatcatg gtcatagctg tt
//

```
LOCUS       pMTV39                 5871 bp    DNA     circular UNA 19-
JAN-2020
DEFINITION  .
ACCESSION   urn.local...5p-exvbs3m
VERSION     urn.local...5p-exvbs3m
KEYWORDS    .
SOURCE
  ORGANISM  .
FEATURES             Location/Qualifiers
     misc_feature    1
                     /label="laczalpha"
     misc_feature    1
                     /label="clone before  this"
     misc_feature    1
                     /label="do not include in cloning"
     primer_bind     1..27
                     /Tm=75
                     /Sequence="AGGATCCTGCGGGTGCCAGGGCGTGCC"
                     /Description="<html><body></body></html>"
                     /label="oMTV206"
     misc_feature    4..23
                     /label="plasmid#1 F"
     misc_feature    8..52
                     /label="c31"
     protein_bind    13..46
                     /bound_moiety="phage œÜC31 integrase"
                     /note="minimal attB site for the œÜC31 integrase (<a
                     href=""http://www.ncbi.nlm.nih.gov/pubmed/10801973""

title=""http://www.ncbi.nlm.nih.gov/pubmed/10801973"">Grot
                     h et al., 2000</a>)"
                     /label="attB"
     terminator      115..361
                     /note="transcription terminator
<i>t</i><sub>L3</sub> from
                     phage Œª"
                     /label="Œª tL3 terminator"
     misc_feature    377..769
                     /label="lambda attP"
     primer_bind     complement(418..438)
                     /Tm=58
                     /Sequence="TTGTCATCAAACCTGTCGCAC"
                     /Description="<html><body></body></html>"
                     /label="oMTV29"
     protein_bind    485..718
                     /bound_moiety="Œª integrase"
                     /gene="<i>att</i>P"
                     /note="integrase from phage Œª"
                     /label="Œª attP"
     misc_feature    770..1206
                     /label="oriR6K"
     rep_origin      785..1173
                     /note="Œ≥ replication origin from <i>E. coli</i>
plasmid
```

```
                     R6K; requires the R6K initiator protein pi for
                     replication"
                     /label="R6K Œ≥ ori"
     primer_bind     complement(848..868)
                     /Tm=60
                     /Sequence="CAAGCCAGGGATGTAACGCAC"
                     /Description="<html><body></body></html>"
                     /label="oMTV28"
     misc_feature    complement(1260..2054)
                     /label="Kan"
     CDS             complement(1260..2054)
                     /codon_start=1
                     /gene="<i>aph(3')-II</i> (or <i>nptII</i>)"
                     /note="confers resistance to neomycin, kanamycin,
and G418
                     (Geneticin¬Æ)"
                     /product="aminoglycoside phosphotransferase from
Tn5"
                     /transl_table=1

/translation="MIEQDGLHAGSPAAWVERLFGYDWAQQTIGCSDAAVFRLSAQGR

PVLFVKTDLSGALNELQDEAARLSWLATTGVPCAAVLDVVTEAGRDWLLLGEVPGQDL

LSSHLAPAEKVSIMADAMRRLHTLDPATCPFDHQAKHRIERARTRMEAGLVDQDDLDE

EHQGLAPAELFARLKARMPDGEDLVVTHGDACLPNIMVENGRFSGFIDCGRLGVADRY
                     QDIALATRDIAEELGGEWADRFLVLYGIAAPDSQRIAFYRLLDEFF*"
                     /label="NeoR/KanR"
     primer_bind     complement(1277..1297)
                     /Tm=60
                     /Sequence="GCATCGCCTTCTATCGCCTTC"
                     /Description="<html><body></body></html>"
                     /label="oMTV26"
     primer_bind     1277..1297
                     /Tm=59
                     /Sequence="GAAGGCGATAGAAGGCGATGC"
                     /Description="<html><body></body></html>"
                     /label="oMTV27"
     primer_bind     complement(1415..1426)
                     /Tm=47
                     /Sequence="GCCGGCGGGGTG"
                     /Mismatch_Positions="======G====="
                     /Mismatches=1

/Extension="AAGTGGTATCGCTTTGGGGAACATGCCTTCAGCGGCGGGCGC"
                     /Description="<html><body></body></html>"
                     /label="oMTV17"
     primer_bind     complement(1733..1754)
                     /Tm=56
                     /Sequence="GATCTCCTGTCATCTCACCTTG"
                     /Description="<html><body></body></html>"
                     /label="oMTV24"
     primer_bind     1733..1754
```

```
                     /Tm=56
                     /Sequence="CAAGGTGAGATGACAGGAGATC"
                     /Description="<html><body></body></html>"
                     /label="oMTV25"
     misc_feature    1948..1965
                     /label="seq1_F"
     misc_feature    2180..2197
                     /label="seq6_F"
     primer_bind     complement(2245..2264)
                     /Tm=57
                     /Sequence="CCTGGCAGTTCCCTACTCTC"
                     /Description="<html><body></body></html>"
                     /label="oMTV22"
     primer_bind     2245..2264
                     /Tm=58
                     /Sequence="GAGAGTAGGGAACTGCCAGG"
                     /Description="<html><body></body></html>"
                     /label="oMTV23"
     terminator      2268..2354
                     /gene="<i>Escherichia coli rrnB</i>"
                     /note="transcription terminator T1 from the <i>E.
coli
                     rrnB</i> gene"
                     /label="rrnB T1 terminator"
     misc_feature    2296..1113
                     /note="prep 1"
                     /label="sequenced"
     misc_feature    2442..2459
                     /label="seq2_F"
     terminator      2446..2473
                     /note="transcription terminator T2 from the <i>E.
coli
                     rrnB</i> gene"
                     /label="rrnB T2 terminator"
     misc_feature    2502..2543
                     /label=31
     misc_feature    2556..2873
                     /note="Geneious type: promoter prokaryotic"
                     /note="/Description=araBp7"
                     /label="Promoter_P_1"
     promoter        2556..2873
                     /note="araBp7"
                     /note="/vntifkey=30"
                     /label="Promoter_P_1"
     promoter        2569..2853
                     /gene="<i>araBAD</i>"
                     /note="promoter of the L-arabinose operon of <i>E.
                     coli</i>; the <i>araC</i> regulatory gene is
transcribed
                     in the opposite direction (<a
                     href=""http://www.ncbi.nlm.nih.gov/pubmed/7608087""

title=""http://www.ncbi.nlm.nih.gov/pubmed/7608087"">Guzma
                     n et al., 1995</a>)"
```

```
                     /label="araBAD promoter"
     primer_bind     2806..2825
                     /Tm=59
                     /Sequence="CGGATCCTACCTGACGCTTT"
                     /Description="<html><body></body></html>"
                     /label="oMTV223"
     primer_bind     complement(2837..2860)
                     /Tm=58
                     /Sequence="AACGGGTATGGAGAAACAGTAGAG"
                     /Description="<html><body></body></html>"
                     /label="oMTV20"
     primer_bind     2837..2860
                     /Tm=59
                     /Sequence="CTCTACTGTTTCTCCATACCCGTT"
                     /Description="<html><body></body></html>"
                     /label="oMTV21"
     primer_bind     complement(2844..2881)
                     /Tm=65
                     /Sequence="ATGAATTCCTCCATCCAAAAAAACGGGTATGGAGAAAC"
                     /Description="<html><body></body></html>"
                     /label="oMTV1"
     primer_bind     complement(2851..2885)
                     /Tm=60
                     /Sequence="TCATATGAATTCCTCCATCCAAAAAAACGGGTATG"
                     /Extension="TTGTGGTCGGTGC"
                     /Description="<html><body></body></html>"
                     /label="oMTV12"
     primer_bind     complement(2851..2881)
                     /Tm=61
                     /Sequence="ATGAATTCCTCCATCCAAAAAAACGGGTATG"
                     /Description="<html><body></body></html>"
                     /label="oMTV205"
     primer_bind     complement(2855..2884)
                     /Tm=60
                     /Sequence="CAGATGAATTCCTCCATCCAAAAAAACGGG"
                     /Mismatch_Positions="==G==========================="
                     /Mismatches=1
                     /Extension="TAGGCCTGC"
                     /Description="<html><body></body></html>"
                     /label="oMTV3"
     primer_bind     2855..2908
                     /Tm=72

/Sequence="CCCGTTTTTTTGGATGGAGGAATTCATATGACCGAGTGGCGTGAACA
                     GAAGCTG"
                     /Description="<html><body></body></html>"
                     /label="oMTV211"
     primer_bind     complement(2856..2885)
                     /Tm=60
                     /Sequence="TCATATGAATTCCTCCATCCAAAAAAACGG"
                     /Extension="CTTTAATATCTT"
                     /Description="<html><body></body></html>"
                     /label="oMTV59"
     primer_bind     complement(2856..2885)
```

```
                     /Tm=58
                     /Sequence="TCATATGAATTCCTCCATCCAAAAAAACGG"
                     /Extension="CGGA"
                     /Description="<html><body></body></html>"
                     /label="oMTV49"
     primer_bind     complement(2866..2881)
                     /Tm=47
                     /Sequence="ATGAATTCCTCCATCC"
                     /label="pLAR067+R4 (2950 R) - Paraburkholderia CDS
2912"
     misc_feature    2881
                     /label="clone after this"
     misc_feature    2882..2901
                     /label="Forward"
     CDS             2882..4048
                     /codon_start=1
                     /transl_table=1

/translation="MTEWREQKLSDFMDFNPYTPLSKGIIAKKVTMEKLIPFNRKIQG

YEDAVFSGGTKFKNGDTLVARITPCLENGKTAYVDFLNDEEVAFGSTEFIVLRAKEGI

SDSRFIFYFAISDEFRDTAIQLMSGTSGRQRVDTEALKRKVFTLPPLPEQKAIAEVLS

SLDDKIDLLTKQNKTLEDLAQAYFRKWFIEDASKEWEVVPISEKFDVLLGGTPSRKIE

SYWTNGTIGWINSGKINEFRIIEASEYITEEALNNSSAKLLPAGTTVLAITGATLGKI

SMVLRSFAANQSVIGLVPKAELSNNFIFLWLKENINALISMQTGGAQQHINSNDVKSF

DVIVPDTVALSLFRRKIDPLMLKISQNCFQINTLNKLRDTLLPKLISGEIRVKM*"
                     /label="Ccla_1830"
     primer_bind     complement(3130..3150)
                     /Tm=57
                     /Sequence="CTACCAAACGCAACTTCCTCA"
                     /Description="<html><body></body></html>"
                     /label="oMTV240"
     primer_bind     3445..3468
                     /Tm=59
                     /Sequence="CATTGAAGATGCGAGCAAAGAGTG"
                     /Description="<html><body></body></html>"
                     /label="oMTV241"
     primer_bind     3866..3887
                     /Tm=58
                     /Sequence="AACGACGTGAAGAGCTTTGATG"
                     /Description="<html><body></body></html>"
                     /label="oMTV242"
     primer_bind     complement(4051..4073)
                     /Tm=58
                     /Sequence="CTTCGCCATATTGAACACCTCCT"
                     /Description="<html><body></body></html>"
                     /label="oMTV243"
     CDS             4065..5657
                     /codon_start=1
```

/transl_table=1

/translation="MAKVKKSKNEVKEQPIEQVLWAAADKLRKNMDAAEYKHVVLGLV

FLKYISDSFYDLYYKLKEGKGEYEGADPDDPYEYRAENVFYVPPQARWDYLQSRAKLP

TIGKDIDEAMEAVEKDNPSLKGVLPKEYAKEKLDKQSLGGLIDLIGTIALGDSVSKSS

DILGQVYEYFLGQFALAEGKKGGQFYTPRSVVQLLVEMLEPYEGRVFDPCCGSGGMFV

QSEKFVEAHRDHYNGKSRGIDKLFERVVSIYGQESNQTTWRLCKMNLAIRGIDSTNVL

WNSEGSFLNDAHPDLKADFVIANPPFNDSDWSGELLRNDGRWKYGVPPVSNANYAWIQ

HFLYHLSPKGTAGFVMAKGSLSSKTNGEGEIRKNIIEAPLVDCIVNLPTKLFLNTQIP

ACLWFLSREKETNSNHPRRNKILFIDARNMGTLINRRTRELTDDDIRKIADTYHSWKK

DVGSYEDIPGFCKSATLDEVRELDYVLTPGRYVGLPEEDDDFDFEERVRKLTAELKEY
MEESAKLDERIKENLAKVGIEL*"
/label="Ccla_1831"
primer_bind 4330..4349
/Tm=61
/Sequence="AGGCGCGTTGGGACTATCTG"
/Description="<html><body></body></html>"
/label="oMTV244"
primer_bind complement(4550..4574)
/Tm=59
/Sequence="GAAATACTCGTAAACCTGACCCAGG"
/Description="<html><body></body></html>"
/label="oMTV245"
primer_bind 4812..4831
/Tm=60
/Sequence="GGCCAGGAAAGCAACCAAAC"
/Description="<html><body></body></html>"
/label="oMTV246"
primer_bind complement(5065..5087)
/Tm=60
/Sequence="GCTCAGGTGATACAGGAAGTGCT"
/Description="<html><body></body></html>"
/label="oMTV247"
primer_bind 5260..5281
/Tm=60
/Sequence="GCCGTGAGAAGGAAACCAACAG"
/Description="<html><body></body></html>"
/label="oMTV248"
primer_bind complement(5583..5606)
/Tm=60
/Sequence="CTTCGCGCTTTCCTCCATGTATTC"
/Description="<html><body></body></html>"
/label="oMTV249"
misc_feature 5658..5715
/label="BXB1 attP"
primer_bind 5658..5684

```
                     /Tm=66
                     /Sequence="GTCGTGGTTTGTCTGGTCAACCACCGC"
                     /Description="<html><body></body></html>"
                     /label="oMTV527"
     primer_bind     complement(5844..27)
                     /Tm=77

/Sequence="GGCACGCCCTGGCACCCGCAGGATCCTAACAGCTATGACCATGATTA
                     CGCCAAGC"
                     /Description="<html><body></body></html>"
                     /label="oMTV212"
     misc_feature    5852..5871
                     /label="Reverse"
ORIGIN
        1 aggatcctgc gggtgccagg gcgtgccctt gggctccccg ggcgcgtact
ccacctcact
       61 cgagaattct catgtttgac agcttatcac tgatcagtga attaatggcg
atgacgcatc
      121 ctcacgataa tatccgggta ggcgcaatca ctttcgtctc tactccgtta
caaagcgagg
      181 ctgggtattt cccggccttt ctgttatccg aaatccactg aaagcacagc
ggctggctga
      241 ggagataaat aataaacgag gggctgtatg cacaaagcat cttctgttga
gttaagaacg
      301 agtatcgaga tggcacatag ccttgctcaa attggaatca ggtttgtgcc
aataccagta
      361 gaaacagacg aagaagctag ctttgcactg gattgcgagg ctttgtgctt
ctctggagtg
      421 cgacaggttt gatgacaaaa aattagcgca agaagacaaa aatcaccttg
cgctaatgct
      481 ctgttacagg tcactaatac catctaagta gttgattcat agtgactgca
tatatgttgt
      541 gttttacagt attatgtagt ctgtttttta tgcaaaatct aatttaatat
attgatattt
      601 atatcatttt acgtttctcg ttcagctttt ttatactaag ttggcattat
aaaaaagcat
      661 tgcttatcaa tttgttgcaa cgaacaggtc actatcagtc aaaataaaat
cattatttga
      721 tttcaatttt gtcccactcc ctgcctctgt catcacgata ctgtgatgcc
atggctaatt
      781 cccatgtcag ccgttaagtg ttcctgtgtc actcaaaatt gctttgagag
gctctaaggg
      841 cttctcagtg cgttacatcc ctggcttgtt gtccacaacc gttaaacctt
aaaagcttta
      901 aaagccttat atattctttt ttttcttata aaacttaaaa ccttagaggc
tatttaagtt
      961 gctgatttat attaatttta ttgttcaaac atgagagctt agtacgtgaa
acatgagagc
     1021 ttagtacgtt agccatgaga gcttagtacg ttagccatga gggtttagtt
cgttaaacat
     1081 gagagcttag tacgttaaac atgagagctt agtacgtgaa acatgagagc
ttagtacgta
     1141 ctatcaacag gttgaactgc tgatcttcag atcctctacg ccggacgcat
cgtggccgga
```

1201 tcttgcggcc gcaaaaatta aaaatgaagt tttgacggta tcgaacccca gagtcccgct
1261 cagaagaact cgtcaagaag gcgatagaag gcgatgcgct gcgaatcggg agcggcgata
1321 ccgtaaagca cgaggaagcg gtcagcccat tcgccgccaa gctcttcagc aatatcacgg
1381 gtagccaacg ctatgtcctg atagcggtcc gccacaccca gccggccaca gtcgatgaat
1441 ccagaaaagc ggccattttc caccatgata ttcggcaagc aggcatcgcc atgggtcacg
1501 acgagatcct cgccgtcggg catccgcgcc ttgagcctgg cgaacagttc ggctggcgcg
1561 agcccctgat gctcttcgtc cagatcatcc tgatcgacaa gaccggcttc catccgagta
1621 cgtgctcgct cgatgcgatg tttcgcttgg tggtcgaatg ggcaggtagc cggatcaagc
1681 gtatgcagcc gccgcattgc atcagccatg atggatactt tctcggcagg agcaaggtga
1741 gatgacagga gatcctgccc cggcacttcg cccaatagca gccagtccct tcccgcttca
1801 gtgacaacgt cgagcacagc tgcgcaagga acgcccgtcg tggccagcca cgatagccgc
1861 gctgcctcgt cttggagttc attcagggca ccggacaggt cggtcttgac aaaaagaacc
1921 gggcgcccct gcgctgacag ccggaacacg gcggcatcag agcagccgat tgtctgttgt
1981 gcccagtcat agccgaatag cctctccacc caagcggccg gagaacctgc gtgcaatcca
2041 tcttgttcaa tcatgcgaaa cgatcctcat cctgtctctt gatccactag attattgaag
2101 catttatcag ggttattgtc tcatgagcgg atacatattt gaatgtattt agaaaaataa
2161 acaaataggg gttccgcgca catttccccg aaaagtgcca cctgcatcga tggcccccga
2221 tggtagtgtg gggtctcccc atgcgagagt agggaactgc caggcatcaa ataaaacgaa
2281 aggctcagtc gaaagactgg gcctttcgtt ttatctgttg tttgtcggtg aacgctctcc
2341 tgagtaggac aaatccgccg ggagcggatt tgaacgttgc gaagcaacgg cccggagggt
2401 ggcgggcagg acgcccgcca taaactgcca ggcatcaaat taagcagaag gccatcctga
2461 cggatggcct ttttgcgtgg ccagtgccaa gcttgcatgc cgtgccccaa ctggggtaac
2521 ctttgagttc tctcagttgg gggcgtaggg tctagccgcc attcagagaa gaaaccaatt
2581 gtccatattg catcagacat tgccgtcact gcgtctttta ctggctcttc tcgctaacca
2641 aaccggtaac cccgcttatt aaaagcattc tgtaacaaag cgggaccaag gccatgacaa
2701 aaacgcgtag caaaagtgtc tataatcacg gcagaaaagt ccacattgat tatttgcacg
2761 gcgtcacact ttgctatgcc atagcatttt tatccataag attagcggat cctacctgac

2821 gctttttatc gcaactctct actgtttctc catacccgtt tttttggatg gaggaattca
2881 tatgaccgag tggcgtgaac agaagctgag cgactttatg gatttcaacc cgtacacccc
2941 gctgagcaaa ggtatcattg cgaagaaagt gaccatggag aagctgatcc cgtttaaccg
3001 taaaattcaa ggctatgaag atgcggtgtt tagcggtggc accaagttca aaaacggtga
3061 caccctggtt gcgcgtatta ccccgtgcct ggaaaacggc aagaccgcgt acgtggactt
3121 cctgaacgat gaggaagttg cgtttggtag caccgagttc attgttctgc gtgcgaaaga
3181 aggcatcagc gatagccgtt tcatctttta tttcgcgatt agcgacgaat ttcgtgatac
3241 cgcgatccag ctgatgagcg gtaccagcgg tcgtcaacgt gtggacaccg aggcgctgaa
3301 gcgtaaagtt tttaccctgc cgccgctgcc ggagcagaag gcgattgcgg aagtgctgag
3361 cagcctggac gataaaatcg atctgctgac caagcagaac aaaaccctgg aggacctggc
3421 gcaagcgtac tttcgtaagt ggttcattga agatgcgagc aaagagtggg aagtggttcc
3481 gatcagcgag aagtttgacg ttctgctggg tggcaccccg agccgtaaaa tcgaaagcta
3541 ctggaccaac ggtaccatcg gctggattaa cagcggcaag attaacgagt tccgtatcat
3601 tgaggcgagc gaatatatca ccgaggaagc gctgaacaac agcagcgcga aactgctgcc
3661 ggcgggtacc accgtgctgg cgattaccgg tgcgaccctg ggcaaaatca gcatggttct
3721 gcgtagcttt gcggcgaacc agagcgtgat tggcctggtt ccgaaggcgg agctgagcaa
3781 caactttatc ttcctgtggc tgaaagaaaa catcaacgcg ctgattagca tgcaaaccgg
3841 tggcgcgcag caacacatta acagcaacga cgtgaagagc tttgatgtga tcgttccgga
3901 caccgttgcg ctgagcctgt tccgtcgtaa gatcgatccg ctgatgctga aaattagcca
3961 gaactgcttc caaatcaaca ccctgaacaa gctgcgtgac accctgctgc cgaaactgat
4021 cagcggcgag attcgtgtga aaatgtaaat aggaggtgtt caatatggcg aaggtgaaga
4081 aaagcaagaa cgaggttaaa gaacaaccga ttgagcaagt gctgtgggcg gcggcggata
4141 agctgcgtaa aaacatggac gcggcggaat acaagcacgt ggttctgggt ctggttttcc
4201 tgaaatacat tagcgacagc ttttatgatc tgtactataa gctgaaagag ggtaaaggcg
4261 agtatgaagg cgcggacccg gacgatccgt acgagtatcg tgcggaaaac gtgttctacg
4321 ttccgccgca ggcgcgttgg gactatctgc aaagccgtgc gaagctgccg accattggta
4381 aagacatcga tgaggcgatg gaagcggtgg agaaggataa cccgagcctg aagggcgttc

4441 tgccgaaaga gtacgcgaag gaaaaactgg ataaacagag cctgggtggc ctgatcgacc
4501 tgattggtac catcgcgctg ggcgatagcg tgagcaagag cagcgacatc ctgggtcagg
4561 tttacgagta tttcctgggt caatttgcgc tggcggaagg caagaaaggt ggccagttct
4621 acaccccgcg tagcgtggtt caactgctgg ttgagatgct ggagccgtat gaaggtcgtg
4681 tttttgaccc gtgctgcggc agcggtggca tgttcgtgca aagcgagaaa tttgttgaag
4741 cgcaccgtga tcactacaac ggcaagagcc gtggcattga caaactgttc gagcgtgtgg
4801 ttagcatcta tggccaggaa agcaaccaaa ccacctggcg tctgtgcaag atgaacctgg
4861 cgattcgtgg tatcgatagc accaacgtgc tgtggaacag cgagggcagc tttctgaacg
4921 acgcgcaccc ggatctgaaa gcggacttcg ttatcgcgaa cccgccgttt aacgacagcg
4981 attggagcgg tgaactgctg cgtaacgacg gtcgttggaa gtacggcgtg ccgccggtta
5041 gcaacgcgaa ctacgcgtgg attcagcact tcctgtatca cctgagcccg aaaggtaccg
5101 cgggctttgt gatggcgaag ggcagcctga gcagcaaaac caacggcgag ggcgaaattc
5161 gtaagaacat cattgaggcg ccgctggttg actgcatcgt taacctgccg accaaactgt
5221 tcctgaacac ccaaattccg gcgtgcctgt ggtttctgag ccgtgagaag gaaaccaaca
5281 gcaaccaccc gcgtcgtaac aaaattctgt tcatcgatgc gcgtaacatg ggtaccctga
5341 tcaaccgtcg tacccgtgag ctgaccgacg atgacattcg taagatcgcg gacacctacc
5401 acagctggaa gaaagatgtg ggtagctatg aagacattcc gggcttttgc aaaagcgcga
5461 ccctggatga agtgcgtgaa ctggactacg ttctgacccc gggtcgttat gtgggtctgc
5521 cggaggaaga tgacgatttc gattttgagg aacgtgttcg taagctgacc gcggagctga
5581 aagaatacat ggaggaaagc gcgaagctgg acgagcgtat taaggaaaac ctggcgaaag
5641 ttggtatcga actgtaagtc gtggtttgtc tggtcaacca ccgcggtctc agtggtgtac
5701 ggtacaaacc ccgaccagaa gagacatgtt cgcgatatga aagctgagtc agtactcatg
5761 catcaattga tctcggatcc tgcgggtgcc agggcgtgcc ctatcggatc ccgggcccgt
5821 cgactgcaga ggcctgcatg caagcttggc gtaatcatgg tcatagctgt t
//

```
LOCUS       pMTV40                  6672 bp    DNA     circular UNA 14-
SEP-2020
DEFINITION  .
ACCESSION   urn.local...5s-exvbs3p
VERSION     urn.local...5s-exvbs3p
KEYWORDS    .
SOURCE
  ORGANISM  .
FEATURES             Location/Qualifiers
     misc_feature    1
                     /label="laczalpha"
     misc_feature    1
                     /label="clone before  this"
     misc_feature    1
                     /label="do not include in cloning"
     primer_bind     1..27
                     /Tm=75
                     /Sequence="AGGATCCTGCGGGTGCCAGGGCGTGCC"
                     /Description="<html><body></body></html>"
                     /label="oMTV206"
     misc_feature    4..23
                     /label="plasmid#1 F"
     misc_feature    8..52
                     /label="c31"
     protein_bind    13..46
                     /bound_moiety="phage œÜC31 integrase"
                     /note="minimal attB site for the œÜC31 integrase (<a
                     href=""http://www.ncbi.nlm.nih.gov/pubmed/10801973""

title=""http://www.ncbi.nlm.nih.gov/pubmed/10801973"">Grot
                     h et al., 2000</a>)"
                     /label="attB"
     terminator      115..361
                     /note="transcription terminator
<i>t</i><sub>L3</sub> from
                     phage Œª"
                     /label="Œª tL3 terminator"
     misc_feature    377..769
                     /label="lambda attP"
     primer_bind     complement(418..438)
                     /Tm=58
                     /Sequence="TTGTCATCAAACCTGTCGCAC"
                     /Description="<html><body></body></html>"
                     /label="oMTV29"
     protein_bind    485..718
                     /bound_moiety="Œª integrase"
                     /gene="<i>att</i>P"
                     /note="integrase from phage Œª"
                     /label="Œª attP"
     misc_feature    770..1206
                     /label="oriR6K"
     rep_origin      785..1173
                     /note="Œ≥ replication origin from <i>E. coli</i>
plasmid
```

```
                     R6K; requires the R6K initiator protein pi for
                     replication"
                     /label="R6K Œ≥ ori"
     primer_bind     complement(848..868)
                     /Tm=60
                     /Sequence="CAAGCCAGGGATGTAACGCAC"
                     /Description="<html><body></body></html>"
                     /label="oMTV28"
     misc_feature    complement(1260..2054)
                     /label="Kan"
     CDS             complement(1260..2054)
                     /codon_start=1
                     /gene="<i>aph(3')-II</i> (or <i>nptII</i>)"
                     /note="confers resistance to neomycin, kanamycin,
and G418
                     (Geneticin¬Æ)"
                     /product="aminoglycoside phosphotransferase from
Tn5"
                     /transl_table=1

/translation="MIEQDGLHAGSPAAWVERLFGYDWAQQTIGCSDAAVFRLSAQGR

PVLFVKTDLSGALNELQDEAARLSWLATTGVPCAAVLDVVTEAGRDWLLLGEVPGQDL

LSSHLAPAEKVSIMADAMRRLHTLDPATCPFDHQAKHRIERARTRMEAGLVDQDDLDE

EHQGLAPAELFARLKARMPDGEDLVVTHGDACLPNIMVENGRFSGFIDCGRLGVADRY
                     QDIALATRDIAEELGGEWADRFLVLYGIAAPDSQRIAFYRLLDEFF*"
                     /label="NeoR/KanR"
     primer_bind     complement(1277..1297)
                     /Tm=60
                     /Sequence="GCATCGCCTTCTATCGCCTTC"
                     /Description="<html><body></body></html>"
                     /label="oMTV26"
     primer_bind     1277..1297
                     /Tm=59
                     /Sequence="GAAGGCGATAGAAGGCGATGC"
                     /Description="<html><body></body></html>"
                     /label="oMTV27"
     primer_bind     complement(1415..1426)
                     /Tm=47
                     /Sequence="GCCGGCGGGGTG"
                     /Mismatch_Positions="======G====="
                     /Mismatches=1

/Extension="AAGTGGTATCGCTTTGGGGAACATGCCTTCAGCGGCGGGCGC"
                     /Description="<html><body></body></html>"
                     /label="oMTV17"
     primer_bind     complement(1733..1754)
                     /Tm=56
                     /Sequence="GATCTCCTGTCATCTCACCTTG"
                     /Description="<html><body></body></html>"
                     /label="oMTV24"
     primer_bind     1733..1754
```

```
                     /Tm=56
                     /Sequence="CAAGGTGAGATGACAGGAGATC"
                     /Description="<html><body></body></html>"
                     /label="oMTV25"
     misc_feature    1948..1965
                     /label="seq1_F"
     misc_feature    2180..2197
                     /label="seq6_F"
     primer_bind     complement(2245..2264)
                     /Tm=57
                     /Sequence="CCTGGCAGTTCCCTACTCTC"
                     /Description="<html><body></body></html>"
                     /label="oMTV22"
     primer_bind     2245..2264
                     /Tm=58
                     /Sequence="GAGAGTAGGGAACTGCCAGG"
                     /Description="<html><body></body></html>"
                     /label="oMTV23"
     terminator      2268..2354
                     /gene="<i>Escherichia coli rrnB</i>"
                     /note="transcription terminator T1 from the <i>E.
coli
                     rrnB</i> gene"
                     /label="rrnB T1 terminator"
     misc_feature    2442..2459
                     /label="seq2_F"
     terminator      2446..2473
                     /note="transcription terminator T2 from the <i>E.
coli
                     rrnB</i> gene"
                     /label="rrnB T2 terminator"
     misc_feature    2502..2543
                     /label=31
     misc_feature    2556..2873
                     /note="Geneious type: promoter prokaryotic"
                     /note="/Description=araBp7"
                     /label="Promoter_P_1"
     promoter        2556..2873
                     /note="araBp7"
                     /note="/vntifkey=30"
                     /label="Promoter_P_1"
     promoter        2569..2853
                     /gene="<i>araBAD</i>"
                     /note="promoter of the L-arabinose operon of <i>E.
                     coli</i>; the <i>araC</i> regulatory gene is
transcribed
                     in the opposite direction (<a
                     href=""http://www.ncbi.nlm.nih.gov/pubmed/7608087""

title=""http://www.ncbi.nlm.nih.gov/pubmed/7608087"">Guzma
                     n et al., 1995</a>)"
                     /label="araBAD promoter"
     primer_bind     2806..2825
                     /Tm=59
```

```
                     /Sequence="CGGATCCTACCTGACGCTTT"
                     /Description="<html><body></body></html>"
                     /label="oMTV223"
     primer_bind     complement(2837..2860)
                     /Tm=58
                     /Sequence="AACGGGTATGGAGAAACAGTAGAG"
                     /Description="<html><body></body></html>"
                     /label="oMTV20"
     primer_bind     2837..2860
                     /Tm=59
                     /Sequence="CTCTACTGTTTCTCCATACCCGTT"
                     /Description="<html><body></body></html>"
                     /label="oMTV21"
     primer_bind     complement(2844..2881)
                     /Tm=65
                     /Sequence="ATGAATTCCTCCATCCAAAAAAACGGGTATGGAGAAAC"
                     /Description="<html><body></body></html>"
                     /label="oMTV1"
     primer_bind     complement(2851..2884)
                     /Tm=61
                     /Sequence="CATATGAATTCCTCCATCCAAAAAAACGGGTATG"
                     /Extension="TTGTGGTCGGTGCT"
                     /Description="<html><body></body></html>"
                     /label="oMTV12"
     primer_bind     complement(2851..2881)
                     /Tm=61
                     /Sequence="ATGAATTCCTCCATCCAAAAAAACGGGTATG"
                     /Description="<html><body></body></html>"
                     /label="oMTV205"
     primer_bind     complement(2855..2885)
                     /Tm=62
                     /Sequence="CCAGATGAATTCCTCCATCCAAAAAAACGGG"

/Mismatch_Positions="===G==========================="
                     /Mismatches=1
                     /Extension="TAGGCCTG"
                     /Description="<html><body></body></html>"
                     /label="oMTV3"
     primer_bind     2855..2911
                     /Tm=68

/Sequence="CCCGTTTTTTTGGATGGAGGAATTCATATGGATAAGGTGGTTATCAA
                     GAAATTTGCG"
                     /Description="<html><body></body></html>"
                     /label="oMTV213"
     primer_bind     complement(2856..2884)
                     /Tm=59
                     /Sequence="CATATGAATTCCTCCATCCAAAAAAACGG"
                     /Extension="CTTTAATATCTTT"
                     /Description="<html><body></body></html>"
                     /label="oMTV59"
     primer_bind     complement(2856..2884)
                     /Tm=59
                     /Sequence="CATATGAATTCCTCCATCCAAAAAAACGG"
```

```
                     /Extension="CGGAT"
                     /Description="<html><body></body></html>"
                     /label="oMTV49"
     primer_bind     complement(2866..2881)
                     /Tm=47
                     /Sequence="ATGAATTCCTCCATCC"
                     /label="pLAR067+R4 (2950 R) - Paraburkholderia CDS
2912"
     misc_feature    2881
                     /label="clone after this"
     misc_feature    2882..6648
                     /label="Ccla1051TP901"
     misc_feature    2882..2901
                     /label="Foward"
     CDS             2882..6517
                     /codon_start=1
                     /transl_table=1

/translation="MDKVVIKKFAVWARKKLIEDIKQKAYELGITEKEIKLPGFATSD

TAIIGDRSLSKKEIEQRKSLVSRIEEKGYNNVIEEVAYTWFNRFIALRFMEVNNYLPT

GVRILSSVEPGKKEPDIIKEALNIDLDLDRELVYKLQDENDTESLYRYLLIKQCNALN

KIFPGLFEKIEDYSEILLPSNLLAEGSVIRRLVEDISEEDFKEQVEVIGWMYQYYISE

KKDEVFEGLKKNKKITKENLPAATQLFTPDWIVKYMLENSLGRLWLEGHPDEELKSKW

KYYLEEAEQEPEVQKQLEEIWAKSKNIRPEDIKVLDPAVGSGHILVYAFDLLYDIYRN

AGYSERDIPKLILENNLYGLDIDDRAVQLAYFAVMMKARSKSRRIFKEKVKVNICAIQ

ESNGFPKEAIDYLVNTGETEIEKRLLREDVEYLINVFHDAKEYGSLLEVKPVDFDAIE

SRLEEIKKGEVQDLVEYQYRNIILEKIPPLVKQARIMSQKYDAVCTNPPYLGRRGMNS

KLAKFVEDSYTTGRNDLFAVFIKICLNYSKNYVSMITQHSWMFLSGFEELRKEILNLT

VIKNMLHLGTRTFEEIGGEVVQSVSFTISKIKVRGYNGLYIRLCDYMSSEEKRRNLFN

RNNYYTVNSDEFFKIEGAPIAYWASPKVKNIFSDSIKLGDIAYPRKGNSTSDNDRFLR

LWFEVDIDKVNFNAKKIIKEETIVRRWFPYNKGGGYRKWYGNNYYLIDWKNDAEEIRK

IPTAVIANYHYFMKPGLTWSTVSTGKFSIRIFGYGFIFDNGGCCLFTDEEDRLYYLAL

LNSNIFDYLLGLLNPTVNYQSGEIAKFPVVFAKSEDAGNRINSLAEDNIQIARIDWDS

FETSWDFKRHPLLAHKGDSITVQQAFNNWSAFAEKQFNRLKANEEELNRIFIEIYGLQ

DELSPEVEDRDITVRKAERVRDIKSFISYAVGCMFGRYSIDAEGLIYAGGEFKDKWKN

EDGKWKVRRIVKDDEGKVIEDLWVDAAFIPDMDNIIPVTDDEYFEDDIVKRFIEFLKV
```

```
TFGEETLEENLDYIADTVGKKADETSRQAIRRYFLREFYKDHVQVYQKRPIYWLFDSG

KENGFKALIYMHRYDEFTVARVRTDYLHKLQKSYEAEIKRLDIIIDSDASQREKAGAR

KKKEKILKQMEECRLYDQVIAHAANQRIEIDLDAGIKVNYARFQGIEIPRGNDRKALK
                     TDLLAKI*"
                     /label="Ccla_1051"
     primer_bind     complement(3093..3116)
                     /Tm=60
                     /Sequence="CCTCGATCACGTTGTTGTAACCTT"
                     /Description="<html><body></body></html>"
                     /label="oMTV250"
     primer_bind     3237..3260
                     /Tm=60
                     /Sequence="TCATTAAAGAGGCGCTGAACATCG"
                     /Description="<html><body></body></html>"
                     /label="oMTV251"
     primer_bind     complement(3543..3562)
                     /Tm=61
                     /Sequence="CAGGCCCTCAAACACTTCGT"
                     /Description="<html><body></body></html>"
                     /label="oMTV252"
     primer_bind     3764..3784
                     /Tm=57
                     /Sequence="GAAATCTGGGCGAAGAGCAAA"
                     /Description="<html><body></body></html>"
                     /label="oMTV253"
     primer_bind     complement(4349..4368)
                     /Tm=59
                     /Sequence="GTGCAAACCGCGTCGTATTT"
                     /Description="<html><body></body></html>"
                     /label="oMTV254"
     primer_bind     4450..4474
                     /Tm=60
                     /Sequence="CGACCTGTTCGCGGTGTTTATTAAG"
                     /Description="<html><body></body></html>"
                     /label="oMTV255"
     primer_bind     complement(4913..4932)
                     /Tm=59
                     /Sequence="CACAGACGCAGGAAACGGTC"
                     /Description="<html><body></body></html>"
                     /label="oMTV256"
     primer_bind     5201..5222
                     /Tm=53
                     /Sequence="TACGGTTTCATCTTTGATAACG"
                     /Description="<html><body></body></html>"
                     /label="oMTV257"
     primer_bind     complement(5393..5414)
                     /Tm=61
                     /Sequence="CTTCCGCCAGGCTGTTAATACG"
                     /Description="<html><body></body></html>"
                     /label="oMTV258"
     primer_bind     5768..5788
```

```
                     /Tm=58
                     /Sequence="GGCGGTGAGTTCAAGGATAAG"
                     /Description="<html><body></body></html>"
                     /label="oMTV259"
     primer_bind     complement(6119..6139)
                     /Tm=57
                     /Sequence="GTCGAACAGCCAATAAATCGG"
                     /Description="<html><body></body></html>"
                     /label="oMTV260"
     primer_bind     complement(6349..6368)
                     /Tm=58
                     /Sequence="CATACAGACGGCACTCTTCC"
                     /Description="<html><body></body></html>"
                     /label="oMTV261"
     misc_feature    6518..6583
                     /label="tp901"
     primer_bind     6518..6587
                     /Tm=67

/Sequence="TCCAACTCGCTTAATTGCGAGTTTTTATTTCGTTTATTTCAATTAAG
                     GTAACTAAAAAACTCCTTTATGC"
                     /Description="<html><body></body></html>"
                     /label="oMTV443"
     misc_feature    6519..6533
                     /label="Primer"
     primer_bind     complement(6646..27)
                     /Tm=78

/Sequence="GGCACGCCCTGGCACCCGCAGGATCCTAACAGCTATGACCATGATTA
                     CGCCGGA"
                     /Description="<html><body></body></html>"
                     /label="oMTV214"
     misc_feature    6653..6672
                     /label="Reverse"
ORIGIN
        1 aggatcctgc gggtgccagg gcgtgccctt gggctccccg ggcgcgtact
ccacctcact
       61 cgagaattct catgtttgac agcttatcac tgatcagtga attaatggcg
atgacgcatc
      121 ctcacgataa tatccgggta ggcgcaatca ctttcgtctc tactccgtta
caaagcgagg
      181 ctgggtattt cccggccttt ctgttatccg aaatccactg aaagcacagc
ggctggctga
      241 ggagataaat aataaacgag ggctgtatg cacaaagcat cttctgttga
gttaagaacg
      301 agtatcgaga tggcacatag ccttgctcaa attggaatca ggtttgtgcc
aataccagta
      361 gaaacagacg aagaagctag ctttgcactg gattgcgagg ctttgtgctt
ctctggagtg
      421 cgacaggttt gatgacaaaa aattagcgca agaagacaaa aatcaccttg
cgctaatgct
      481 ctgttacagg tcactaatac catctaagta gttgattcat agtgactgca
tatatgttgt
```

541 gttttacagt attatgtagt ctgtttttta tgcaaaatct aatttaatat attgatattt
601 atatcatttt acgtttctcg ttcagctttt ttatactaag ttggcattat aaaaaagcat
661 tgcttatcaa tttgttgcaa cgaacaggtc actatcagtc aaaataaaat cattatttga
721 tttcaatttt gtcccactcc ctgcctctgt catcacgata ctgtgatgcc atggctaatt
781 cccatgtcag ccgttaagtg ttcctgtgtc actcaaaatt gctttgagag gctctaaggg
841 cttctcagtg cgttacatcc ctggcttgtt gtccacaacc gttaaacctt aaaagcttta
901 aaagccttat atattctttt ttttcttata aaacttaaaa ccttagaggc tatttaagtt
961 gctgatttat attaatttta ttgttcaaac atgagagctt agtacgtgaa acatgagagc
1021 ttagtacgtt agccatgaga gcttagtacg ttagccatga gggtttagtt cgttaaacat
1081 gagagcttag tacgttaaac atgagagctt agtacgtgaa acatgagagc ttagtacgta
1141 ctatcaacag gttgaactgc tgatcttcag atcctctacg ccggacgcat cgtggccgga
1201 tcttgcggcc gcaaaaatta aaaatgaagt tttgacggta tcgaacccca gagtcccgct
1261 cagaagaact cgtcaagaag gcgatagaag gcgatgcgct gcgaatcggg agcggcgata
1321 ccgtaaagca cgaggaagcg gtcagcccat tcgccgccaa gctcttcagc aatatcacgg
1381 gtagccaacg ctatgtcctg atagcggtcc gccacaccca gccggccaca gtcgatgaat
1441 ccagaaaagc ggccattttc caccatgata ttcggcaagc aggcatcgcc atgggtcacg
1501 acgagatcct cgccgtcggg catccgcgcc ttgagcctgg cgaacagttc ggctggcgcg
1561 agcccctgat gctcttcgtc cagatcatcc tgatcgacaa gaccggcttc catccgagta
1621 cgtgctcgct cgatgcgatg tttcgcttgg tggtcgaatg ggcaggtagc cggatcaagc
1681 gtatgcagcc gccgcattgc atcagccatg atggatactt tctcggcagg agcaaggtga
1741 gatgacagga gatcctgccc cggcacttcg cccaatagca gccagtccct tcccgcttca
1801 gtgacaacgt cgagcacagc tgcgcaagga acgcccgtcg tggccagcca cgatagccgc
1861 gctgcctcgt cttggagttc attcagggca ccggacaggt cggtcttgac aaaaagaacc
1921 gggcgcccct gcgctgacag ccggaacacg gcggcatcag agcagccgat tgtctgttgt
1981 gcccagtcat agccgaatag cctctccacc caagcggccg gagaacctgc gtgcaatcca
2041 tcttgttcaa tcatgcgaaa cgatcctcat cctgtctctt gatccactag attattgaag
2101 catttatcag ggttattgtc tcatgagcgg atacatattt gaatgtattt agaaaaataa

2161 acaaataggg gttccgcgca catttccccg aaaagtgcca cctgcatcga tggcccccga
2221 tggtagtgtg gggtctcccc atgcgagagt agggaactgc caggcatcaa ataaaacgaa
2281 aggctcagtc gaaagactgg gcctttcgtt ttatctgttg tttgtcggtg aacgctctcc
2341 tgagtaggac aaatccgccg ggagcggatt tgaacgttgc gaagcaacgg cccggagggt
2401 ggcgggcagg acgcccgcca taaactgcca ggcatcaaat taagcagaag gccatcctga
2461 cggatggcct ttttgcgtgg ccagtgccaa gcttgcatgc cgtgccccaa ctggggtaac
2521 ctttgagttc tctcagttgg gggcgtaggg tctagccgcc attcagagaa gaaaccaatt
2581 gtccatattg catcagacat tgccgtcact gcgtctttta ctggctcttc tcgctaacca
2641 aaccggtaac cccgcttatt aaaagcattc tgtaacaaag cgggaccaag gccatgacaa
2701 aaacgcgtag caaaagtgtc tataatcacg gcagaaaagt ccacattgat tatttgcacg
2761 gcgtcacact ttgctatgcc atagcatttt tatccataag attagcggat cctacctgac
2821 gctttttatc gcaactctct actgtttctc catacccgtt tttttggatg gaggaattca
2881 tatggataag gtggttatca agaaatttgc ggtgtgggcg cgtaagaaac tgatcgaaga
2941 cattaaacag aaggcgtacg agctgggcat caccgaaaag gagattaaac tgccgggttt
3001 cgcgaccagc gataccgcga tcattggcga ccgtagcctg agcaagaaag aaatcgagca
3061 gcgtaagagc ctggttagcc gtattgagga aaaaggttac aacaacgtga tcgaggaagt
3121 tgcgtatacc tggttcaacc gttttattgc gctgcgtttc atggaagtga acaactatct
3181 gccgaccggt gtgcgtatcc tgagcagcgt tgaaccgggc aagaaagagc cggacatcat
3241 taaagaggcg ctgaacatcg acctggatct ggaccgtgaa ctggtttaca aactgcagga
3301 tgaaaacgac accgagagcc tgtaccgtta tctgctgatt aagcaatgca acgcgctgaa
3361 caaaatcttc ccgggtctgt ttgaaaagat tgaggattat agcgaaattc tgctgccgag
3421 caacctgctg gcggagggca gcgtgattcg tcgtctggtt gaagatatca gcgaggaaga
3481 cttcaaagaa caggttgagg tgatcggttg gatgtaccaa tactatatca gcgagaagaa
3541 agacgaagtg tttgagggcc tgaagaaaaa caagaaaatt accaaggaaa acctgccggc
3601 ggcgacccag ctgttcaccc cggattggat cgttaaatat atgctggaga acagcctggg
3661 ccgtctgtgg ctggaaggtc acccggacga ggaactgaaa agcaagtgga aatactatct
3721 ggaggaagcg gaacaagagc cggaagtgca gaagcaactg gaggaaatct gggcgaagag

3781 caaaaacatc cgtccggagg atattaaagt tctggacccg gcggttggca gcggtcacat
3841 tctggtttat gcgtttgatc tgctgtacga catctatcgt aacgcgggtt acagcgagcg
3901 tgatatcccg aagctgattc tggaaaacaa cctgtatggc ctggacattg acgatcgtgc
3961 ggtgcagctg gcgtacttcg cggttatgat gaaggcgcgt agcaaaagcc gtcgtatctt
4021 caaggaaaag gtgaaggtta acatctgcgc gattcaagaa agcaacggtt tcccgaaaga
4081 ggcgattgac tacctggtga acaccggcga gaccgaaatc gagaaacgtc tgctgcgtga
4141 agatgtggag tatctgatca acgtttttca cgacgcgaag gaatacggta gcctgctgga
4201 agtgaaaccg gttgatttcg acgcgattga gagccgtctg gaggaaatca agaaaggcga
4261 agtgcaggat ctggttgagt accaatatcg taacatcatt ctggaaaaga ttccgccgct
4321 ggtgaaacag gcgcgtatca tgagccaaaa atacgacgcg gtttgcacca acccgccgta
4381 tctgggccgt cgtggtatga acagcaagct ggcgaaattc gtggaggata gctacaccac
4441 cggtcgtaac gacctgttcg cggtgtttat taagatctgc ctgaactaca gcaaaaacta
4501 tgttagcatg atcacccagc acagctggat gttcctgagc ggttttgagg aactgcgtaa
4561 ggagattctg aacctgaccg ttatcaaaaa catgctgcac ctgggcaccc gtaccttcga
4621 ggaaattggt ggcgaagtgg ttcaaagcgt gagctttacc atcagcaaaa ttaaggttcg
4681 tggctacaac ggtctgtata tccgtctgtg cgattacatg agcagcgagg aaaagcgtcg
4741 taacctgttt aaccgtaaca actactatac cgtgaacagc gacgaattct ttaaaatcga
4801 gggtgcgccg attgcgtatt gggcgagccc gaaagttaag aacatcttca gcgatagcat
4861 taagctgggc gacatcgcgt acccgcgtaa gggtaacagc accagcgata acgaccgttt
4921 cctgcgtctg tggtttgagg tggatattga caaggttaac ttcaacgcga agaaaatcat
4981 taaagaggaa accatcgttc gtcgttggtt tccgtacaac aagggtggcg gttaccgtaa
5041 atggtatggt aacaactact atctgattga ttggaagaac gacgcggagg aaatccgtaa
5101 aattccgacc gcggtgatcg cgaactacca ctatttcatg aagccgggcc tgacctggag
5161 caccgttagc accggtaaat tcagcattcg tatctttggc tacggtttca tctttgataa
5221 cggcggttgc tgcctgttta ccgatgagga agaccgtctg tactatctgg cgctgctgaa
5281 cagcaacatt ttcgactatc tgctgggtct gctgaacccg accgtgaact accagagcgg
5341 cgagatcgcg aagttcccgg tggtttttgc gaaaagcgag gatgcgggta accgtattaa

5401 cagcctggcg gaagacaaca ttcaaatcgc gcgtatcgat tgggacagct
tcgagaccag
5461 ctgggatttt aagcgtcacc cgctgctggc gcacaaaggc gacagcatca
ccgttcagca
5521 agcgtttaac aactggagcg cgttcgcgga aaagcagttt aaccgtctga
aagcgaacga
5581 ggaagagctg aaccgtattt tcatcgaaat ttacggtctg caagacgagc
tgagcccgga
5641 agtggaggat cgtgacatca ccgtgcgtaa ggcggagcgt gttcgtgata
tcaaaagctt
5701 cattagctac gcggttggct gcatgtttgg tcgttatagc attgacgcgg
aaggtctgat
5761 ctacgcgggc ggtgagttca aggataagtg gaaaaacgaa gacggcaagt
ggaaagtgcg
5821 tcgtattgtt aaggacgatg aaggcaaagt gatcgaggat ctgtgggttg
acgcggcgtt
5881 catcccggat atggacaaca tcattccggt gaccgacgat gaatattttg
aggacgatat
5941 tgtgaagcgt ttcatcgaat ttctgaaagt taccttcggt gaagagaccc
tggaagagaa
6001 cctggattac attgcggaca ccgttggcaa gaaagcggac gagaccagcc
gtcaggcgat
6061 ccgtcgttac ttcctgcgtg aattttataa ggatcacgtg caggtttacc
aaaaacgtcc
6121 gatttattgg ctgttcgaca gcggcaagga gaacggtttt aaagcgctga
tctacatgca
6181 ccgttatgat gaatttaccg tggcgcgtgt tcgtaccgac tacctgcaca
agctgcagaa
6241 aagctatgaa gcggagatca agcgtctgga tatcattatc gatagcgatg
cgagccagcg
6301 tgagaaagcg ggtgcgcgta agaaaaagga aaagatcctg aagcaaatgg
aagagtgccg
6361 tctgtatgac caggtgatcg cgcacgcggc gaaccaacgt atcgagattg
atctggacgc
6421 gggcattaag gttaactacg cgcgtttcca gggcatcgaa attccgcgtg
gtaacgatcg
6481 taaggcgctg aaaaccgacc tgctggcgaa aatctaatcc aactcgctta
attgcgagtt
6541 tttatttcgt ttatttcaat taaggtaact aaaaaactcc tttatgcatg
agtcagtact
6601 ccaattgatc tctcgcgata gacatgttga aagctcagaa gaggatccgg
cgtaatcatg
6661 gtcatagctg tt
//

```
LOCUS       pMTV41                  5690 bp    DNA     circular UNA 24-
MAR-2020
DEFINITION  .
ACCESSION   urn.local...5v-exvbs3r
VERSION     urn.local...5v-exvbs3r
KEYWORDS    .
SOURCE
  ORGANISM  .
FEATURES             Location/Qualifiers
     misc_feature    1
                     /label="laczalpha"
     misc_feature    1
                     /label="clone before  this"
     misc_feature    1
                     /label="do not include in cloning"
     primer_bind     1..27
                     /Tm=75
                     /Sequence="AGGATCCTGCGGGTGCCAGGGCGTGCC"
                     /Description="<html><body></body></html>"
                     /label="oMTV206"
     misc_feature    4..23
                     /label="plasmid#1 F"
     misc_feature    8..52
                     /label="c31"
     protein_bind    13..46
                     /bound_moiety="phage œÜC31 integrase"
                     /note="minimal attB site for the œÜC31 integrase (<a
                     href=""http://www.ncbi.nlm.nih.gov/pubmed/10801973""

title=""http://www.ncbi.nlm.nih.gov/pubmed/10801973"">Grot
                     h et al., 2000</a>)"
                     /label="attB"
     terminator      115..361
                     /note="transcription terminator
<i>t</i><sub>L3</sub> from
                     phage Œª"
                     /label="Œª tL3 terminator"
     misc_feature    377..769
                     /label="lambda attP"
     primer_bind     complement(418..438)
                     /Tm=58
                     /Sequence="TTGTCATCAAACCTGTCGCAC"
                     /Description="<html><body></body></html>"
                     /label="oMTV29"
     protein_bind    485..718
                     /bound_moiety="Œª integrase"
                     /gene="<i>att</i>P"
                     /note="integrase from phage Œª"
                     /label="Œª attP"
     misc_feature    770..1206
                     /label="oriR6K"
     rep_origin      785..1173
                     /note="Œ≥ replication origin from <i>E. coli</i>
plasmid
```

```
                     R6K; requires the R6K initiator protein pi for
                     replication"
                     /label="R6K Œ≥ ori"
     primer_bind     complement(848..868)
                     /Tm=60
                     /Sequence="CAAGCCAGGGATGTAACGCAC"
                     /Description="<html><body></body></html>"
                     /label="oMTV28"
     misc_feature    complement(1260..2054)
                     /label="Kan"
     CDS             complement(1260..2054)
                     /codon_start=1
                     /gene="<i>aph(3')-II</i> (or <i>nptII</i>)"
                     /note="confers resistance to neomycin, kanamycin,
and G418
                     (Geneticin¬Æ)"
                     /product="aminoglycoside phosphotransferase from
Tn5"
                     /transl_table=1

/translation="MIEQDGLHAGSPAAWVERLFGYDWAQQTIGCSDAAVFRLSAQGR

PVLFVKTDLSGALNELQDEAARLSWLATTGVPCAAVLDVVTEAGRDWLLLGEVPGQDL

LSSHLAPAEKVSIMADAMRRLHTLDPATCPFDHQAKHRIERARTRMEAGLVDQDDLDE

EHQGLAPAELFARLKARMPDGEDLVVTHGDACLPNIMVENGRFSGFIDCGRLGVADRY
                     QDIALATRDIAEELGGEWADRFLVLYGIAAPDSQRIAFYRLLDEFF*"
                     /label="NeoR/KanR"
     primer_bind     complement(1277..1297)
                     /Tm=60
                     /Sequence="GCATCGCCTTCTATCGCCTTC"
                     /Description="<html><body></body></html>"
                     /label="oMTV26"
     primer_bind     1277..1297
                     /Tm=59
                     /Sequence="GAAGGCGATAGAAGGCGATGC"
                     /Description="<html><body></body></html>"
                     /label="oMTV27"
     primer_bind     complement(1415..1426)
                     /Tm=47
                     /Sequence="GCCGGCGGGGTG"
                     /Mismatch_Positions="======G====="
                     /Mismatches=1

/Extension="AAGTGGTATCGCTTTGGGGAACATGCCTTCAGCGGCGGGCGC"
                     /Description="<html><body></body></html>"
                     /label="oMTV17"
     primer_bind     complement(1733..1754)
                     /Tm=56
                     /Sequence="GATCTCCTGTCATCTCACCTTG"
                     /Description="<html><body></body></html>"
                     /label="oMTV24"
     primer_bind     1733..1754
```

```
                     /Tm=56
                     /Sequence="CAAGGTGAGATGACAGGAGATC"
                     /Description="<html><body></body></html>"
                     /label="oMTV25"
     misc_feature    1948..1965
                     /label="seq1_F"
     misc_feature    2011..1067
                     /note="prep 1 and prep 2"
                     /label="sequenced"
     misc_feature    2180..2197
                     /label="seq6_F"
     primer_bind     complement(2245..2264)
                     /Tm=57
                     /Sequence="CCTGGCAGTTCCCTACTCTC"
                     /Description="<html><body></body></html>"
                     /label="oMTV22"
     primer_bind     2245..2264
                     /Tm=58
                     /Sequence="GAGAGTAGGGAACTGCCAGG"
                     /Description="<html><body></body></html>"
                     /label="oMTV23"
     terminator      2268..2354
                     /gene="<i>Escherichia coli rrnB</i>"
                     /note="transcription terminator T1 from the <i>E.
coli
                     rrnB</i> gene"
                     /label="rrnB T1 terminator"
     misc_feature    2442..2459
                     /label="seq2_F"
     terminator      2446..2473
                     /note="transcription terminator T2 from the <i>E.
coli
                     rrnB</i> gene"
                     /label="rrnB T2 terminator"
     misc_feature    2502..2543
                     /label=31
     misc_feature    2556..2873
                     /note="Geneious type: promoter prokaryotic"
                     /note="/Description=araBp7"
                     /label="Promoter_P_1"
     promoter        2556..2873
                     /note="araBp7"
                     /note="/vntifkey=30"
                     /label="Promoter_P_1"
     promoter        2569..2853
                     /gene="<i>araBAD</i>"
                     /note="promoter of the L-arabinose operon of <i>E.
                     coli</i>; the <i>araC</i> regulatory gene is
transcribed
                     in the opposite direction (<a
                     href=""http://www.ncbi.nlm.nih.gov/pubmed/7608087""

title=""http://www.ncbi.nlm.nih.gov/pubmed/7608087"">Guzma
                     n et al., 1995</a>)"
```

```
                     /label="araBAD promoter"
     primer_bind     2806..2825
                     /Tm=59
                     /Sequence="CGGATCCTACCTGACGCTTT"
                     /Description="<html><body></body></html>"
                     /label="oMTV223"
     primer_bind     complement(2837..2860)
                     /Tm=58
                     /Sequence="AACGGGTATGGAGAAACAGTAGAG"
                     /Description="<html><body></body></html>"
                     /label="oMTV20"
     primer_bind     2837..2860
                     /Tm=59
                     /Sequence="CTCTACTGTTTCTCCATACCCGTT"
                     /Description="<html><body></body></html>"
                     /label="oMTV21"
     primer_bind     complement(2844..2881)
                     /Tm=65
                     /Sequence="ATGAATTCCTCCATCCAAAAAAACGGGTATGGAGAAAC"
                     /Description="<html><body></body></html>"
                     /label="oMTV1"
     primer_bind     complement(2851..2885)
                     /Tm=61
                     /Sequence="TCATATGAATTCCTCCATCCAAAAAAACGGGTATG"
                     /Extension="TTGTGGTCGGTGC"
                     /Description="<html><body></body></html>"
                     /label="oMTV12"
     primer_bind     complement(2851..2881)
                     /Tm=61
                     /Sequence="ATGAATTCCTCCATCCAAAAAAACGGGTATG"
                     /Description="<html><body></body></html>"
                     /label="oMTV205"
     primer_bind     complement(2855..2884)
                     /Tm=60
                     /Sequence="CAGATGAATTCCTCCATCCAAAAAAACGGG"
                     /Mismatch_Positions="==G==========================="
                     /Mismatches=1
                     /Extension="TAGGCCTGC"
                     /Description="<html><body></body></html>"
                     /label="oMTV3"
     primer_bind     2855..2908
                     /Tm=69

/Sequence="CCCGTTTTTTTGGATGGAGGAATTCATATGAACATTCAGAAGATCAG
                     CGTGGAG"
                     /Description="<html><body></body></html>"
                     /label="oMTV215"
     primer_bind     complement(2856..2886)
                     /Tm=60
                     /Sequence="TTCATATGAATTCCTCCATCCAAAAAAACGG"
                     /Extension="CTTTAATATCT"
                     /Description="<html><body></body></html>"
                     /label="oMTV59"
     primer_bind     complement(2856..2885)
```

/Tm=60
/Sequence="TCATATGAATTCCTCCATCCAAAAAAACGG"
/Extension="CGGA"
/Description="<html><body></body></html>"
/label="oMTV49"
primer_bind complement(2866..2881)
/Tm=47
/Sequence="ATGAATTCCTCCATCC"
/label="pLAR067+R4 (2950 R) - Paraburkholderia CDS
2912"
misc_feature 2881
/label="clone after this"
misc_feature 2882..5621
/label="Ccla1868-9TG1"
misc_feature 2882..2901
/label="Forward"
CDS 2882..4219
/codon_start=1
/transl_table=1

/translation="MNIQKISVEKLNPAAYNPRKDLKPGDKEYEKLKRSIEEFGYVEP

VIWNQKTGNVVGGHQRLKVLLDLGQTEIDCVVVDLDPQREKALNLALNKIQGEWDENK

LAELMAELDAGAFDVSLTGFDASEIDELLNRWYSKEAVQDSFDIDKAHEEIVQREPVT

KRGDIWLLGNHRLMCGDSTKDEDFEKLMEGCHAQMAVTSPPYGVGKEYEKAGIEPWFE

TVRPVIRNLCRYADIVCWNLGDLYATGSQFIEPTSVYSVNMFLENGYRPIWIRIWKKQ

GQNFGVGPYHLVSNKPVQQYEYISAFSNKGEVEEYNDQEYVWLSAFAGHSYKFVKRLT

KEERKKWGYAGIWEMTTVRANKEHPAMFPVELPWRCIKMHSDKGGIVLEPFSGSGTTI

IAAEQTERRCYAMELSPVYCDLAVKRWEDFTGEKAVKLLPHEAGFMGAPGLED*"
/label="Ccla_1868"
primer_bind complement(3088..3110)
/Tm=60
/Sequence="CAACCACGCAATCAATCTCGGTC"
/Description="<html><body></body></html>"
/label="oMTV262"
primer_bind 3314..3335
/Tm=60
/Sequence="GACATTGATAAAGCGCACGAGG"
/Description="<html><body></body></html>"
/label="oMTV263"
primer_bind complement(3667..3687)
/Tm=59
/Sequence="CAGATCGGACGATAGCCGTTT"
/Description="<html><body></body></html>"
/label="oMTV264"
primer_bind 3923..3942
/Tm=58
/Sequence="GAAATGACCACCGTTCGTGC"

```
                     /Description="<html><body></body></html>"
                     /label="oMTV265"
     CDS             4236..5489
                     /codon_start=1
                     /transl_table=1

/translation="MEIQKIPAEKLKAAKYNPRKDLKPGDPEYEKLRRSIEEFGYVEP

VIWNRRTGNIVGGHQRYKVLTALGYKEIDCVVVDLDEQREKALNVALNKISGEFDIPL

LTDLLKGLNEDGFDVSLTGFDAAEIDELFRDKTAANVKEDNFDAEKAVSEIKMPVTQR

GDIWLLGRHRLMCGDSALLSDVQKLMAGQKARFVFTDPPWNVDYGSDARHPSWKPRQI

LNDRMSTEEFGAFLLSAFNCMREVSEPGCMTYVVMSAQEWGNVMNALREAGYHWSSTI

IWKKDSLVLSRKDYHTQYEPIWYGWLEGTRLCPLKDRKQSDVWEIPRPKVSEEHPTMK

PVSLVAKAMLNSSHTGDLALDLFGGSGTTMIAAEQTGRVCFMMELDPKYCDVIAKRYV
                     SQFGDNAAFLLRGDEKIPYAETQIA*"
                     /label="Ccla_1869"
     primer_bind     complement(4276..4295)
                     /Tm=56
                     /Sequence="CTTACGCGGGTTGTATTTCG"
                     /Description="<html><body></body></html>"
                     /label="oMTV266"
     primer_bind     4524..4547
                     /Tm=59
                     /Sequence="GAGTTTGATATTCCGCTGCTGACC"
                     /Description="<html><body></body></html>"
                     /label="oMTV267"
     primer_bind     complement(4880..4899)
                     /Tm=59
                     /Sequence="GGTCGTTCAGGATCTGACGC"
                     /Description="<html><body></body></html>"
                     /label="oMTV268"
     primer_bind     5124..5146
                     /Tm=61
                     /Sequence="ATCTGGTATGGTTGGCTGGAAGG"
                     /Description="<html><body></body></html>"
                     /label="oMTV269"
     primer_bind     complement(5409..5430)
                     /Tm=60
                     /Sequence="CGTTATCACCGAACTGGCTCAC"
                     /Description="<html><body></body></html>"
                     /label="oMTV270"
     misc_feature    5490..5556
                     /label="TG1 attP"
     misc_feature    5490
                     /label="Clone before this"
     primer_bind     5490..5516
                     /Tm=68
                     /Sequence="TCAACCCCGTTCCAGCCCAACAGTGTT"
                     /Description="<html><body></body></html>"
```

```
                     /label="oMTV894"
     primer_bind     complement(5664..27)
                     /Tm=77

/Sequence="GGCACGCCCTGGCACCCGCAGGATCCTAACAGCTATGACCATGATTA
                     CGCCAAG"
                     /Description="<html><body></body></html>"
                     /label="oMTV216"
     misc_feature    5671..5690
                     /label="Reverse"
ORIGIN
        1 aggatcctgc gggtgccagg gcgtgccctt gggctccccg ggcgcgtact
ccacctcact
       61 cgagaattct catgtttgac agcttatcac tgatcagtga attaatggcg
atgacgcatc
      121 ctcacgataa tatccgggta ggcgcaatca ctttcgtctc tactccgtta
caaagcgagg
      181 ctgggtattt cccggccttt ctgttatccg aaatccactg aaagcacagc
ggctggctga
      241 ggagataaat aataaacgag gggctgtatg cacaaagcat cttctgttga
gttaagaacg
      301 agtatcgaga tggcacatag ccttgctcaa attggaatca ggtttgtgcc
aataccagta
      361 gaaacagacg aagaagctag ctttgcactg gattgcgagg ctttgtgctt
ctctggagtg
      421 cgacaggttt gatgacaaaa aattagcgca agaagacaaa aatcaccttg
cgctaatgct
      481 ctgttacagg tcactaatac catctaagta gttgattcat agtgactgca
tatatgttgt
      541 gttttacagt attatgtagt ctgtttttta tgcaaaatct aatttaatat
attgatattt
      601 atatcatttt acgtttctcg ttcagctttt ttatactaag ttggcattat
aaaaaagcat
      661 tgcttatcaa tttgttgcaa cgaacaggtc actatcagtc aaaataaaat
cattatttga
      721 tttcaatttt gtcccactcc ctgcctctgt catcacgata ctgtgatgcc
atggctaatt
      781 cccatgtcag ccgttaagtg ttcctgtgtc actcaaaatt gctttgagag
gctctaaggg
      841 cttctcagtg cgttacatcc ctggcttgtt gtccacaacc gttaaacctt
aaaagcttta
      901 aaagccttat atattctttt ttttcttata aaacttaaaa ccttagaggc
tatttaagtt
      961 gctgatttat attaatttta ttgttcaaac atgagagctt agtacgtgaa
acatgagagc
     1021 ttagtacgtt agccatgaga gcttagtacg ttagccatga gggtttagtt
cgttaaacat
     1081 gagagcttag tacgttaaac atgagagctt agtacgtgaa acatgagagc
ttagtacgta
     1141 ctatcaacag gttgaactgc tgatcttcag atcctctacg ccggacgcat
cgtggccgga
     1201 tcttgcggcc gcaaaaatta aaaatgaagt tttgacggta tcgaacccca
gagtcccgct
```

1261 cagaagaact cgtcaagaag gcgatagaag gcgatgcgct gcgaatcggg agcggcgata
1321 ccgtaaagca cgaggaagcg gtcagcccat tcgccgccaa gctcttcagc aatatcacgg
1381 gtagccaacg ctatgtcctg atagcggtcc gccacaccca gccggccaca gtcgatgaat
1441 ccagaaaagc ggccattttc caccatgata ttcggcaagc aggcatcgcc atgggtcacg
1501 acgagatcct cgccgtcggg catccgcgcc ttgagcctgg cgaacagttc ggctggcgcg
1561 agcccctgat gctcttcgtc cagatcatcc tgatcgacaa gaccggcttc catccgagta
1621 cgtgctcgct cgatgcgatg tttcgcttgg tggtcgaatg ggcaggtagc cggatcaagc
1681 gtatgcagcc gccgcattgc atcagccatg atggatactt tctcggcagg agcaaggtga
1741 gatgacagga gatcctgccc cggcacttcg cccaatagca gccagtccct tcccgcttca
1801 gtgacaacgt cgagcacagc tgcgcaagga acgcccgtcg tggccagcca cgatagccgc
1861 gctgcctcgt cttggagttc attcagggca ccggacaggt cggtcttgac aaaaagaacc
1921 gggcgcccct gcgctgacag ccggaacacg gcggcatcag agcagccgat tgtctgttgt
1981 gcccagtcat agccgaatag cctctccacc caagcggccg gagaacctgc gtgcaatcca
2041 tcttgttcaa tcatgcgaaa cgatcctcat cctgtctctt gatccactag attattgaag
2101 catttatcag ggttattgtc tcatgagcgg atacatattt gaatgtattt agaaaaataa
2161 acaaataggg gttccgcgca catttccccg aaaagtgcca cctgcatcga tggcccccga
2221 tggtagtgtg gggtctcccc atgcgagagt agggaactgc caggcatcaa ataaaacgaa
2281 aggctcagtc gaaagactgg gcctttcgtt ttatctgttg tttgtcggtg aacgctctcc
2341 tgagtaggac aaatccgccg ggagcggatt tgaacgttgc gaagcaacgg cccggagggt
2401 ggcgggcagg acgcccgcca taaactgcca ggcatcaaat taagcagaag gccatcctga
2461 cggatggcct ttttgcgtgg ccagtgccaa gcttgcatgc cgtgccccaa ctggggtaac
2521 ctttgagttc tctcagttgg gggcgtaggg tctagccgcc attcagagaa gaaaccaatt
2581 gtccatattg catcagacat tgccgtcact gcgtctttta ctggctcttc tcgctaacca
2641 aaccggtaac cccgcttatt aaaagcattc tgtaacaaag cgggaccaag gccatgacaa
2701 aaacgcgtag caaaagtgtc tataatcacg gcagaaaagt ccacattgat tatttgcacg
2761 gcgtcacact ttgctatgcc atagcatttt tatccataag attagcggat cctacctgac
2821 gctttttatc gcaactctct actgtttctc catacccgtt tttttggatg gaggaattca

2881 tatgaacatt cagaagatca gcgtggagaa actgaacccg gcggcgtaca acccgcgtaa
2941 ggacctgaaa ccgggcgata aggagtacga aaagctgaaa cgtagcattg aggaattcgg
3001 ctatgttgaa ccggtgatct ggaaccagaa gaccggtaac gtggttggtg gccaccaacg
3061 tctgaaagtt ctgctggacc tgggccagac cgagattgat tgcgtggttg tggacctgga
3121 cccgcagcgt gaaaaggcgc tgaacctggc gctgaacaaa atccagggcg agtgggacga
3181 aaacaagctg gcggagctga tggcggaact ggatgcgggc gcgttcgatg ttagcctgac
3241 cggttttgac gcgagcgaga ttgatgaact gctgaaccgt tggtatagca aagaggcggt
3301 gcaagatagc tttgacattg ataaagcgca cgaggaaatc gttcagcgtg aaccggtgac
3361 caaacgtggc gacatctggc tgctgggtaa ccaccgtctg atgtgcggcg atagcaccaa
3421 ggacgaggat ttcgaaaaac tgatggaggg ttgccacgcg caaatggcgg ttaccagccc
3481 gccgtacggt gtgggcaagg agtatgaaaa agcgggtatt gagccgtggt ttgaaaccgt
3541 tcgtccggtg attcgtaacc tgtgccgtta cgcggacatc gtttgctgga acctgggcga
3601 tctgtatgcg accggtagcc agttcatcga gccgaccagc gtttacagcg tgaacatgtt
3661 tctggaaaac ggctatcgtc cgatctggat tcgtatctgg aagaaacagg gtcaaaactt
3721 cggtgtgggc ccgtaccacc tggttagcaa caagccggtg cagcaatacg agtatattag
3781 cgcgtttagc aacaaaggcg aggttgagga atacaacgac caagaatatg tgtggctgag
3841 cgcgttcgcg ggtcacagct acaagtttgt taaacgtctg accaaagagg agcgtaagaa
3901 atggggttat gcgggcatct gggaaatgac caccgttcgt gcgaacaaag agcacccggc
3961 gatgttcccg gtggaactgc cgtggcgttg cattaagatg cacagcgata aaggtggcat
4021 cgttctggag ccgtttagcg gtagcggcac caccatcatt gcggcggagc agaccgaacg
4081 tcgttgctac gcgatggaac tgagcccggt ttattgcgac ctggcggtga aacgttggga
4141 ggatttcacc ggtgaaaaag cggtgaaact gctgccgcat gaggcgggtt ttatgggtgc
4201 gccgggtctg gaagactaaa taggaggtgt tcaatatgga gatccagaag attccggcgg
4261 aaaagctgaa agcggcgaaa tacaacccgc gtaaggacct gaaaccgggt gatccggagt
4321 acgaaaagct gcgtcgtagc atcgaggaat tcggttatgt tgagccggtg atctggaacc
4381 gtcgtaccgg caacattgtt ggtggccacc agcgttacaa ggtgctgacc gcgctgggct
4441 ataaagaaat tgattgcgtg gttgtggacc tggatgagca acgtgaaaag gcgctgaacg

4501 ttgcgctgaa caaaatcagc ggcgagtttg atattccgct gctgaccgac ctgctgaaag
4561 gtctgaacga agacggcttc gatgttagcc tgaccggctt tgacgcggcg gagatcgatg
4621 aactgttccg tgacaagacc gcggcgaacg tgaaagagga caactttgat gcggagaagg
4681 cggttagcga aatcaaaatg ccggtgaccc aacgtggtga catttggctg ctgggccgtc
4741 accgtctgat gtgcggtgat agcgcgctgc tgagcgacgt gcagaagctg atggcgggcc
4801 aaaaagcgcg tttcgttttt accgatccgc cgtggaacgt ggactacggt agcgatgcgc
4861 gtcacccgag ctggaagccg cgtcagatcc tgaacgaccg tatgagcacc gaggaattcg
4921 gtgcgtttct gctgagcgcg ttcaactgca tgcgtgaggt ttctgaaccg ggctgcatga
4981 cctacgttgt gatgagcgcg caagagtggg gtaacgtgat gaacgcgctg cgtgaagcgg
5041 gctatcactg gagcagcacc atcatttgga agaaagatag cctggttctg agccgtaagg
5101 actaccacac ccagtatgag ccgatctggt atggttggct ggaaggcacc cgtctgtgcc
5161 cgctgaagga tcgtaaacaa agcgacgttt gggaaattcc gcgtccgaaa gtgagcgagg
5221 aacacccgac catgaagccg gttagcctgg tggcgaaagc gatgctgaac agcagccaca
5281 ccggtgatct ggcgctggac ctgttcggtg gcagcggtac caccatgatt gcggcggagc
5341 agaccggtcg tgtgtgcttt atgatggaac tggacccgaa gtactgcgac gttattgcga
5401 aacgttatgt gagccagttc ggtgataacg cggcgtttct gctgcgtggc gacgagaaaa
5461 tcccgtatgc ggaaacccaa attgcgtaat caaccccgtt ccagcccaac agtgttagtc
5521 tttgctctta cccagttggg cgggatagcc tgcccgatgc atcagaagac aattgatctc
5581 gacatgttga aagcttcgcg atagagtcag tactcggatc catcggatcc cgggcccgtc
5641 gactgcagag gcctgcatgc aagcttggcg taatcatggt catagctgtt
//

```
LOCUS       pMTV42                  4603 bp    DNA     circular UNA 16-
OCT-2019
DEFINITION  .
ACCESSION   urn.local...5y-exvbs3t
VERSION     urn.local...5y-exvbs3t
KEYWORDS    .
SOURCE
  ORGANISM  .
FEATURES             Location/Qualifiers
     misc_feature    5..24
                     /standard_name="plasmid#1 F"
                     /label="plasmid#1 F"
     misc_feature    9..53
                     /standard_name="c31"
                     /label="c31"
     protein_bind    14..47
                     /bound_moiety="phage œÜC31 integrase"
                     /note="minimal attB site for the œÜC31 integrase (<a
                     href=""http://www.ncbi.nlm.nih.gov/pubmed/10801973""

title=""http://www.ncbi.nlm.nih.gov/pubmed/10801973"">Grot
                     h et al., 2000</a>)"
                     /label="attB"
     terminator      116..362
                     /note="transcription terminator
<i>t</i><sub>L3</sub> from
                     phage Œª"
                     /label="Œª tL3 terminator"
     misc_feature    378..770
                     /standard_name="lambda attP"
                     /label="lambda attP"
     primer_bind     complement(419..439)
                     /Tm=58
                     /Sequence="TTGTCATCAAACCTGTCGCAC"
                     /Description="<html><body></body></html>"
                     /label="oMTV29"
     protein_bind    486..719
                     /bound_moiety="Œª integrase"
                     /gene="<i>att</i>P"
                     /note="integrase from phage Œª"
                     /label="Œª attP"
     misc_feature    771..1207
                     /standard_name="oriR6K"
                     /label="oriR6K"
     rep_origin      786..1174
                     /note="Œ≥ replication origin from <i>E. coli</i>
plasmid
                     R6K; requires the R6K initiator protein pi for
                     replication"
                     /label="R6K Œ≥ ori"
     primer_bind     complement(849..869)
                     /Tm=60
                     /Sequence="CAAGCCAGGGATGTAACGCAC"
                     /Description="<html><body></body></html>"
```

```
                     /label="oMTV28"
     misc_feature    1261..2055
                     /standard_name="Kan"
                     /label="Kan"
     CDS             complement(1261..2055)
                     /codon_start=1
                     /gene="<i>aph(3')-II</i> (or <i>nptII</i>)"
                     /note="confers resistance to neomycin, kanamycin,
and G418
                     (Geneticin¬Æ)"
                     /product="aminoglycoside phosphotransferase from
Tn5"
                     /transl_table=1

/translation="MIEQDGLHAGSPAAWVERLFGYDWAQQTIGCSDAAVFRLSAQGR

PVLFVKTDLSGALNELQDEAARLSWLATTGVPCAAVLDVVTEAGRDWLLLGEVPGQDL

LSSHLAPAEKVSIMADAMRRLHTLDPATCPFDHQAKHRIERARTRMEAGLVDQDDLDE

EHQGLAPAELFARLKARMPDGEDLVVTHGDACLPNIMVENGRFSGFIDCGRLGVADRY
                     QDIALATRDIAEELGGEWADRFLVLYGIAAPDSQRIAFYRLLDEFF*"
                     /label="NeoR/KanR"
     primer_bind     1278..1298
                     /Tm=59
                     /Sequence="GAAGGCGATAGAAGGCGATGC"
                     /Description="<html><body></body></html>"
                     /label="oMTV27"
     primer_bind     complement(1278..1298)
                     /Tm=60
                     /Sequence="GCATCGCCTTCTATCGCCTTC"
                     /Description="<html><body></body></html>"
                     /label="oMTV26"
     primer_bind     1734..1755
                     /Tm=56
                     /Sequence="CAAGGTGAGATGACAGGAGATC"
                     /Description="<html><body></body></html>"
                     /label="oMTV25"
     primer_bind     complement(1734..1755)
                     /Tm=56
                     /Sequence="GATCTCCTGTCATCTCACCTTG"
                     /Description="<html><body></body></html>"
                     /label="oMTV24"
     misc_feature    1894..2882
                     /label="sequenced"
     misc_feature    1949..1966
                     /standard_name="seq1_F"
                     /label="seq1_F"
     misc_feature    2180..418
                     /note="prep 2"
                     /label="sequenced "
     misc_feature    2181..2198
                     /standard_name="seq6_F"
                     /label="seq6_F"
```

```
     primer_bind     2246..2265
                     /Tm=58
                     /Sequence="GAGAGTAGGGAACTGCCAGG"
                     /Description="<html><body></body></html>"
                     /label="oMTV23"
     primer_bind     complement(2246..2265)
                     /Tm=57
                     /Sequence="CCTGGCAGTTCCCTACTCTC"
                     /Description="<html><body></body></html>"
                     /label="oMTV22"
     terminator      2269..2355
                     /gene="<i>Escherichia coli rrnB</i>"
                     /note="transcription terminator T1 from the <i>E.
coli
                     rrnB</i> gene"
                     /label="rrnB T1 terminator"
     misc_feature    2443..2460
                     /standard_name="seq2_F"
                     /label="seq2_F"
     terminator      2447..2474
                     /note="transcription terminator T2 from the <i>E.
coli
                     rrnB</i> gene"
                     /label="rrnB T2 terminator"
     misc_feature    2503..2544
                     /standard_name=31
                     /label=31
     promoter        2557..2874
                     /note="araBp7"
                     /note="/vntifkey=30"
                     /label="Promoter_P_1"
     promoter        2570..2854
                     /gene="<i>araBAD</i>"
                     /note="promoter of the L-arabinose operon of <i>E.
                     coli</i>; the <i>araC</i> regulatory gene is
transcribed
                     in the opposite direction (<a
                     href=""http://www.ncbi.nlm.nih.gov/pubmed/7608087""

title=""http://www.ncbi.nlm.nih.gov/pubmed/7608087"">Guzma
                     n et al., 1995</a>)"
                     /label="araBAD promoter"
     primer_bind     2807..2826
                     /Tm=59
                     /Sequence="CGGATCCTACCTGACGCTTT"
                     /Description="<html><body></body></html>"
                     /label="oMTV223"
     primer_bind     2838..2861
                     /Tm=59
                     /Sequence="CTCTACTGTTTCTCCATACCCGTT"
                     /Description="<html><body></body></html>"
                     /label="oMTV21"
     primer_bind     complement(2838..2861)
                     /Tm=58
```

```
                     /Sequence="AACGGGTATGGAGAAACAGTAGAG"
                     /Description="<html><body></body></html>"
                     /label="oMTV20"
     primer_bind     complement(2845..2882)
                     /Tm=65
                     /Sequence="ATGAATTCCTCCATCCAAAAAAACGGGTATGGAGAAAC"
                     /Description="<html><body></body></html>"
                     /label="oMTV1"
     primer_bind     complement(2852..2885)
                     /Tm=61
                     /Sequence="CATATGAATTCCTCCATCCAAAAAAACGGGTATG"
                     /Extension="TTGTGGTCGGTGCT"
                     /Description="<html><body></body></html>"
                     /label="oMTV12"
     primer_bind     complement(2856..2886)
                     /Tm=62
                     /Sequence="CCAGATGAATTCCTCCATCCAAAAAAACGGG"

/Mismatch_Positions="===G==========================="
                     /Mismatches=1
                     /Extension="TAGGCCTG"
                     /Description="<html><body></body></html>"
                     /label="oMTV3"
     primer_bind     complement(2856..2882)
                     /Tm=61
                     /Sequence="ATGAATTCCTCCATCCAAAAAAACGGG"
                     /Description="<html><body></body></html>"
                     /label="oMTV217"
     primer_bind     2856..2909
                     /Tm=69

/Sequence="CCCGTTTTTTTGGATGGAGGAATTCATATGGATCGTAAGCAGAAACT
                     GGAACTG"
                     /Description="<html><body></body></html>"
                     /label="oMTV219"
     primer_bind     complement(2857..2885)
                     /Tm=59
                     /Sequence="CATATGAATTCCTCCATCCAAAAAAACGG"
                     /Extension="CGGAT"
                     /Description="<html><body></body></html>"
                     /label="oMTV49"
     misc_feature    2883..4463
                     /label="Ccla1961PhiFC1"
     misc_feature    2883..2902
                     /label="Forward"
     CDS             2883..4463
                     /codon_start=1
                     /transl_table=1

/translation="MDRKQKLELTWIGKYDEIKVEPRILLEDKSKGYGDPNTENMLIH

GDNLLALKALEQDFAGKIKCIYIDPPYNTGNAFEHYDDNLEHSIWLNLMKPRLEILKN

LLSNDGSIWISIDADESHYLKVLCDEIFGRKNFIDEIIWQRAFAPVNLKKTLSRSHDC
```

ILVYAKNYDNEFQLNKLPRSETQLSNYKNPDNDPRGLWTSSDCTVGPVVPEKLYEITL

PSGRKVTPPDGRCWLYTKERFDELVRDNRIWFGENGNNVPRVKKFLTEVKGGVVAITL

WLRDEVGDNQEAKREVKAFNNDDVFTTPKPERLIQRILTLATNPGDWVLDSFLGSGTT

AAVAHKMGRRWIGIELGDHCYTHCLPRLKAVVDGEQGGISKTVNWQGGGGFRFFELAP

SLLKKDKYGNWVIDERYNADMLAAAMAKHQGFTYAPDSEVFWKQGYSYEKDYIFTTTQ

FVTLEYLDLIHQEMAEDESLLICCKAFQDGADQRYENINVKKIPQVLLNKCDFGVESY
SLNIVGLPEIDEEVDDNE*"
/label="Ccla_1961"
primer_bind complement(3198..3221)
/Tm=62
/Sequence="GCTAATCCAGATGCTACCGTCGTT"
/Description="<html><body></body></html>"
/label="oMTV271"
primer_bind 3361..3380
/Tm=59
/Sequence="GCATCCTGGTTTACGCGAAG"
/Description="<html><body></body></html>"
/label="oMTV272"
primer_bind complement(3530..3563)
/Tm=50
/Sequence="ACATGCCTTCAGCGGCGGGCGCGCCGGCGGGGTG"

/Mismatch_Positions="ACAT=CCT===GCGGCG=G=GCGC====G====="
/Mismatches=19
/Extension="AAGTGGTATCGCTTTGGGGA"
/Description="<html><body></body></html>"
/label="oMTV17"
primer_bind complement(3583..3602)
/Tm=56
/Sequence="GTCGAAACGCTCCTTGGTAT"
/Description="<html><body></body></html>"
/label="oMTV273"
primer_bind 3941..3960
/Tm=60
/Sequence="CTGCTACACCCATTGCCTGC"
/Description="<html><body></body></html>"
/label="oMTV274"
primer_bind complement(4165..4187)
/Tm=61
/Sequence="CTTCCAGAACACCTCGCTATCCG"
/Description="<html><body></body></html>"
/label="oMTV275"
primer_bind 4269..4288
/Tm=55
/Sequence="GAAATGGCGGAGGATGAAAG"
/Description="<html><body></body></html>"
/label="oMTV276"
primer_bind complement(4437..4490)

```
                     /Tm=66

/Sequence="AGTTTTTCATTGTTCAACAGTATTCTCTTATTCGTTATCGTCAACTT
                     CCTCGTC"
                     /Description="<html><body></body></html>"
                     /label="oMTV220"
     primer_bind     4463..4491
                     /Tm=55
                     /Sequence="AGAGAATACTGTTGAACAATGAAAAACTA"
                     /Extension="ATGGCGGCTTG"
                     /Description="<html><body></body></html>"
                     /label="oMTV50"
     misc_feature    4464..723
                     /label="sequenced"
     misc_feature    4464..4524
                     /standard_name="BL3 attP"
                     /label="BL3 attP"
     misc_feature    4464..2882
                     /note="for cloning to include BLE att site"
                     /label="pMMW003-backbone"
     primer_bind     4464..4513
                     /Tm=65

/Sequence="GAGAATACTGTTGAACAATGAAAAACTAGGCATGTAGAAGTTGTTTG
                     TGC"
                     /Description="<html><body></body></html>"
                     /label="oMTV218"
     misc_feature    4564..4583
                     /standard_name="Reverse"
                     /label="Reverse"
     misc_feature    complement(4564..4583)
                     /note="Geneious type: primer_bind_reverse"
                     /standard_name="pGSs060 extraction (2091 R) -
pLAR067
                     extraction"
                     /label="pGSs060 extraction (2091 R) - pLAR067
extraction"
     misc_feature    4584..4603
                     /note="Geneious type: ligation"
                     /standard_name="Ligation"
                     /label="Ligation"
     misc_feature    4584..2
                     /standard_name="laczalpha"
                     /label="laczalpha"
ORIGIN
        1 aaggatcctg cgggtgccag ggcgtgccct tgggctcccc gggcgcgtac
tccacctcac
       61 tcgagaattc tcatgtttga cagcttatca ctgatcagtg aattaatggc
gatgacgcat
      121 cctcacgata atatccgggt aggcgcaatc actttcgtct ctactccgtt
acaaagcgag
      181 gctgggtatt tcccggcctt tctgttatcc gaaatccact gaaagcacag
cggctggctg
```

241 aggagataaa taataaacga ggggctgtat gcacaaagca tcttctgttg agttaagaac
301 gagtatcgag atggcacata gccttgctca aattggaatc aggtttgtgc caataccagt
361 agaaacagac gaagaagcta gctttgcact ggattgcgag gctttgtgct tctctggagt
421 gcgacaggtt tgatgacaaa aaattagcgc aagaagacaa aaatcacctt gcgctaatgc
481 tctgttacag gtcactaata ccatctaagt agttgattca tagtgactgc atatatgttg
541 tgttttacag tattatgtag tctgtttttt atgcaaaatc taatttaata tattgatatt
601 tatatcattt tacgtttctc gttcagcttt tttatactaa gttggcatta taaaaaagca
661 ttgcttatca atttgttgca acgaacaggt cactatcagt caaaataaaa tcattatttg
721 atttcaattt tgtcccactc cctgcctctg tcatcacgat actgtgatgc catggctaat
781 tcccatgtca gccgttaagt gttcctgtgt cactcaaaat tgctttgaga ggctctaagg
841 gcttctcagt gcgttacatc cctggcttgt tgtccacaac cgttaaacct taaaagcttt
901 aaaagcctta tatattcttt tttttcttat aaaacttaaa accttagagg ctatttaagt
961 tgctgattta tattaatttt attgttcaaa catgagagct tagtacgtga aacatgagag
1021 cttagtacgt tagccatgag agcttagtac gttagccatg agggtttagt tcgttaaaca
1081 tgagagctta gtacgttaaa catgagagct tagtacgtga aacatgagag cttagtacgt
1141 actatcaaca ggttgaactg ctgatcttca gatcctctac gccggacgca tcgtggccgg
1201 atcttgcggc cgcaaaaatt aaaaatgaag ttttgacggt atcgaacccc agagtcccgc
1261 tcagaagaac tcgtcaagaa ggcgatagaa ggcgatgcgc tgcgaatcgg gagcggcgat
1321 accgtaaagc acgaggaagc ggtcagccca ttcgccgcca agctcttcag caatatcacg
1381 ggtagccaac gctatgtcct gatagcggtc cgccacaccc agccggccac agtcgatgaa
1441 tccagaaaag cggccatttt ccaccatgat attcggcaag caggcatcgc catgggtcac
1501 gacgagatcc tcgccgtcgg gcatccgcgc cttgagcctg gcgaacagtt cggctggcgc
1561 gagcccctga tgctcttcgt ccagatcatc ctgatcgaca agaccggctt ccatccgagt
1621 acgtgctcgc tcgatgcgat gtttcgcttg gtggtcgaat gggcaggtag ccggatcaag
1681 cgtatgcagc cgccgcattg catcagccat gatggatact ttctcggcag gagcaaggtg
1741 agatgacagg agatcctgcc ccggcacttc gcccaatagc agccagtccc ttcccgcttc
1801 agtgacaacg tcgagcacag ctgcgcaagg aacgcccgtc gtggccagcc acgatagccg

1861 cgctgcctcg tcttggagtt cattcagggc accggacagg tcggtcttga caaaaagaac
1921 cgggcgcccc tgcgctgaca gccggaacac ggcggcatca gagcagccga ttgtctgttg
1981 tgcccagtca tagccgaata gcctctccac ccaagcggcc ggagaacctg cgtgcaatcc
2041 atcttgttca atcatgcgaa acgatcctca tcctgtctct tgatccacta gattattgaa
2101 gcatttatca gggttattgt ctcatgagcg gatacatatt tgaatgtatt tagaaaaata
2161 aacaaatagg ggttccgcgc acatttcccc gaaaagtgcc acctgcatcg atggcccccg
2221 atggtagtgt ggggtctccc catgcgagag tagggaactg ccaggcatca aataaaacga
2281 aaggctcagt cgaaagactg ggcctttcgt tttatctgtt gtttgtcggt gaacgctctc
2341 ctgagtagga caaatccgcc gggagcggat ttgaacgttg cgaagcaacg gcccggaggg
2401 tggcgggcag gacgcccgcc ataaactgcc aggcatcaaa ttaagcagaa ggccatcctg
2461 acggatggcc tttttgcgtg gccagtgcca agcttgcatg ccgtgcccca actggggtaa
2521 cctttgagtt ctctcagttg ggggcgtagg gtctagccgc cattcagaga agaaaccaat
2581 tgtccatatt gcatcagaca ttgccgtcac tgcgtctttt actggctctt ctcgctaacc
2641 aaaccggtaa ccccgcttat taaaagcatt ctgtaacaaa gcgggaccaa ggccatgaca
2701 aaaacgcgta gcaaaagtgt ctataatcac ggcagaaaag tccacattga ttatttgcac
2761 ggcgtcacac tttgctatgc catagcattt ttatccataa gattagcgga tcctacctga
2821 cgctttttat cgcaactctc tactgtttct ccatacccgt ttttttggat ggaggaattc
2881 atatggatcg taagcagaaa ctggaactga cctggattgg caagtacgac gagatcaaag
2941 ttgaaccgcg tattctgctg gaagacaaga gcaaaggtta tggcgatccg aacaccgaga
3001 acatgctgat ccacggcgac aacctgctgg cgctgaaggc gctggaacaa gatttcgcgg
3061 gcaagattaa atgcatctac attgacccgc cgtataacac cggcaacgcg tttgagcact
3121 acgacgataa cctggaacac agcatctggc tgaacctgat gaagccgcgt ctggagattc
3181 tgaaaaacct gctgagcaac gacggtagca tctggattag catcgacgcg gatgagagcc
3241 actatctgaa ggtgctgtgc gacgaaatct tcggccgtaa gaacttcatc gatgagatca
3301 tttggcagcg tgcgttcgcg ccggtgaacc tgaagaaaac cctgagccgt agccacgact
3361 gcatcctggt ttacgcgaag aactatgata acgaatttca gctgaacaaa ctgccgcgta
3421 gcgagaccca actgagcaac tacaaaaacc cggacaacga tccgcgtggt ctgtggacca

3481 gcagcgattg caccgtgggt ccggtggttc cggagaagct gtacgaaatc
accctgccga
3541 gcggtcgtaa agtgaccccg ccggatggcc gttgctggct gtataccaag
gagcgtttcg
3601 acgaactggt gcgtgataac cgtatctggt ttggtgaaaa cggcaacaac
gtgccgcgtg
3661 ttaagaaatt cctgaccgag gtgaaaggtg gcgtggttgc gattaccctg
tggctgcgtg
3721 acgaagttgg tgataaccag gaagcgaagc gtgaagtgaa agcgttcaac
aacgacgatg
3781 tttttaccac cccgaagccg gagcgtctga ttcaacgtat cctgaccctg
gcgaccaacc
3841 cgggtgactg ggtgctggat agcttcctgg gtagcggtac caccgcggcg
gttgcgcaca
3901 aaatgggccg tcgttggatt ggtatcgaac tgggcgatca ctgctacacc
cattgcctgc
3961 cgcgtctgaa ggcggtggtt gacggcgagc agggtggcat cagcaaaacc
gttaactggc
4021 aaggtggcgg tggctttcgt ttctttgaac tggcgccgag cctgctgaag
aaagacaagt
4081 acggcaactg ggtgattgac gagcgttata acgcggatat gctggcggcg
gcgatggcga
4141 aacaccaggg tttcacctat gcgccggata gcgaggtgtt ctggaagcaa
ggctacagct
4201 atgagaaaga ctacatcttc accaccaccc agtttgtgac cctggagtat
ctggacctga
4261 tccaccaaga aatggcggag gatgaaagcc tgctgatttg ctgcaaggcg
ttccaggacg
4321 gtgcggatca acgttacgaa aacattaacg tgaagaaaat cccgcaggtt
ctgctgaaca
4381 aatgcgattt tggtgtggag agctatagcc tgaacatcgt tggcctgccg
gagattgacg
4441 aggaagttga cgataacgaa taagagaata ctgttgaaca atgaaaaact
aggcatgtag
4501 aagttgtttg tgcactaact ttaaccaagc ttgttaatga caaaaagtcc
cggggatccg
4561 gcgtaatcat ggtcatagct gtttttcag cctgatacag att
//

```
LOCUS       pMTV43                  6690 bp    DNA     circular UNA 24-
MAR-2020
DEFINITION  .
ACCESSION   urn.local...61-exvbs3v
VERSION     urn.local...61-exvbs3v
KEYWORDS    .
SOURCE
  ORGANISM  .
FEATURES             Location/Qualifiers
     misc_feature    1
                     /label="laczalpha"
     misc_feature    1
                     /label="clone before  this"
     misc_feature    1
                     /label="do not include in cloning"
     primer_bind     1..27
                     /Tm=75
                     /Sequence="AGGATCCTGCGGGTGCCAGGGCGTGCC"
                     /Description="<html><body></body></html>"
                     /label="oMTV206"
     misc_feature    4..23
                     /label="plasmid#1 F"
     misc_feature    8..52
                     /label="c31"
     protein_bind    13..46
                     /bound_moiety="phage œÜC31 integrase"
                     /note="minimal attB site for the œÜC31 integrase (<a
                     href=""http://www.ncbi.nlm.nih.gov/pubmed/10801973""

title=""http://www.ncbi.nlm.nih.gov/pubmed/10801973"">Grot
                     h et al., 2000</a>)"
                     /label="attB"
     terminator      115..361
                     /note="transcription terminator
<i>t</i><sub>L3</sub> from
                     phage Œª"
                     /label="Œª tL3 terminator"
     misc_feature    377..769
                     /label="lambda attP"
     primer_bind     complement(418..438)
                     /Tm=58
                     /Sequence="TTGTCATCAAACCTGTCGCAC"
                     /Description="<html><body></body></html>"
                     /label="oMTV29"
     protein_bind    485..718
                     /bound_moiety="Œª integrase"
                     /gene="<i>att</i>P"
                     /note="integrase from phage Œª"
                     /label="Œª attP"
     misc_feature    770..1206
                     /label="oriR6K"
     rep_origin      785..1173
                     /note="Œ≥ replication origin from <i>E. coli</i>
plasmid
```

```
                     R6K; requires the R6K initiator protein pi for
                     replication"
                     /label="R6K Œ≥ ori"
     primer_bind     complement(848..868)
                     /Tm=60
                     /Sequence="CAAGCCAGGGATGTAACGCAC"
                     /Description="<html><body></body></html>"
                     /label="oMTV28"
     misc_feature    complement(1260..2054)
                     /label="Kan"
     CDS             complement(1260..2054)
                     /codon_start=1
                     /gene="<i>aph(3')-II</i> (or <i>nptII</i>)"
                     /note="confers resistance to neomycin, kanamycin,
and G418
                     (Geneticin¬Æ)"
                     /product="aminoglycoside phosphotransferase from
Tn5"
                     /transl_table=1

/translation="MIEQDGLHAGSPAAWVERLFGYDWAQQTIGCSDAAVFRLSAQGR

PVLFVKTDLSGALNELQDEAARLSWLATTGVPCAAVLDVVTEAGRDWLLLGEVPGQDL

LSSHLAPAEKVSIMADAMRRLHTLDPATCPFDHQAKHRIERARTRMEAGLVDQDDLDE

EHQGLAPAELFARLKARMPDGEDLVVTHGDACLPNIMVENGRFSGFIDCGRLGVADRY
                     QDIALATRDIAEELGGEWADRFLVLYGIAAPDSQRIAFYRLLDEFF*"
                     /label="NeoR/KanR"
     primer_bind     complement(1277..1297)
                     /Tm=60
                     /Sequence="GCATCGCCTTCTATCGCCTTC"
                     /Description="<html><body></body></html>"
                     /label="oMTV26"
     primer_bind     1277..1297
                     /Tm=59
                     /Sequence="GAAGGCGATAGAAGGCGATGC"
                     /Description="<html><body></body></html>"
                     /label="oMTV27"
     primer_bind     complement(1415..1426)
                     /Tm=47
                     /Sequence="GCCGGCGGGGTG"
                     /Mismatch_Positions="======G====="
                     /Mismatches=1

/Extension="AAGTGGTATCGCTTTGGGGAACATGCCTTCAGCGGCGGGCGC"
                     /Description="<html><body></body></html>"
                     /label="oMTV17"
     primer_bind     complement(1733..1754)
                     /Tm=56
                     /Sequence="GATCTCCTGTCATCTCACCTTG"
                     /Description="<html><body></body></html>"
                     /label="oMTV24"
     primer_bind     1733..1754
```

```
                    /Tm=56
                    /Sequence="CAAGGTGAGATGACAGGAGATC"
                    /Description="<html><body></body></html>"
                    /label="oMTV25"
     misc_feature   1948..1965
                    /label="seq1_F"
     misc_feature   2140..383
                    /note="prep 6"
                    /label="sequenced pMTV43 prep 6 and prep 7"
     misc_feature   2180..2197
                    /label="seq6_F"
     primer_bind    complement(2245..2264)
                    /Tm=57
                    /Sequence="CCTGGCAGTTCCCTACTCTC"
                    /Description="<html><body></body></html>"
                    /label="oMTV22"
     primer_bind    2245..2264
                    /Tm=58
                    /Sequence="GAGAGTAGGGAACTGCCAGG"
                    /Description="<html><body></body></html>"
                    /label="oMTV23"
     terminator     2268..2354
                    /gene="<i>Escherichia coli rrnB</i>"
                    /note="transcription terminator T1 from the <i>E.
coli
                    rrnB</i> gene"
                    /label="rrnB T1 terminator"
     misc_feature   2442..2459
                    /label="seq2_F"
     terminator     2446..2473
                    /note="transcription terminator T2 from the <i>E.
coli
                    rrnB</i> gene"
                    /label="rrnB T2 terminator"
     misc_feature   2502..2543
                    /label=31
     misc_feature   2556..2873
                    /note="Geneious type: promoter prokaryotic"
                    /note="/Description=araBp7"
                    /label="Promoter_P_1"
     promoter       2556..2873
                    /note="araBp7"
                    /note="/vntifkey=30"
                    /label="Promoter_P_1"
     promoter       2569..2853
                    /gene="<i>araBAD</i>"
                    /note="promoter of the L-arabinose operon of <i>E.
                    coli</i>; the <i>araC</i> regulatory gene is
transcribed
                    in the opposite direction (<a
                    href=""http://www.ncbi.nlm.nih.gov/pubmed/7608087""

title=""http://www.ncbi.nlm.nih.gov/pubmed/7608087"">Guzma
                    n et al., 1995</a>)"
```

```
                     /label="araBAD promoter"
     primer_bind     2806..2825
                     /Tm=59
                     /Sequence="CGGATCCTACCTGACGCTTT"
                     /label="3,770 F"
     primer_bind     2806..2825
                     /Tm=59
                     /Sequence="CGGATCCTACCTGACGCTTT"
                     /Description="<html><body></body></html>"
                     /label="oMTV223"
     primer_bind     complement(2837..2860)
                     /Tm=58
                     /Sequence="AACGGGTATGGAGAAACAGTAGAG"
                     /Description="<html><body></body></html>"
                     /label="oMTV20"
     primer_bind     2837..2860
                     /Tm=59
                     /Sequence="CTCTACTGTTTCTCCATACCCGTT"
                     /Description="<html><body></body></html>"
                     /label="oMTV21"
     primer_bind     complement(2844..2881)
                     /Tm=65
                     /Sequence="ATGAATTCCTCCATCCAAAAAAACGGGTATGGAGAAAC"
                     /Description="<html><body></body></html>"
                     /label="oMTV1"
     primer_bind     complement(2851..2884)
                     /Tm=61
                     /Sequence="CATATGAATTCCTCCATCCAAAAAAACGGGTATG"
                     /Extension="TTGTGGTCGGTGCT"
                     /Description="<html><body></body></html>"
                     /label="oMTV12"
     primer_bind     complement(2851..2881)
                     /Tm=61
                     /Sequence="ATGAATTCCTCCATCCAAAAAAACGGGTATG"
                     /Description="<html><body></body></html>"
                     /label="oMTV205"
     primer_bind     complement(2855..2885)
                     /Tm=59
                     /Sequence="CCAGATGAATTCCTCCATCCAAAAAAACGGG"

/Mismatch_Positions="===G==========================="
                     /Mismatches=1
                     /Extension="TAGGCCTG"
                     /Description="<html><body></body></html>"
                     /label="oMTV3"
     primer_bind     2855..2908
                     /Tm=69

/Sequence="CCCGTTTTTTTGGATGGAGGAATTCATATGGATAAGGCGGCGATCAA
                     GAAATTC"
                     /Description="<html><body></body></html>"
                     /label="oMTV221"
     primer_bind     complement(2856..2884)
                     /Tm=59
```

```
                     /Sequence="CATATGAATTCCTCCATCCAAAAAAACGG"
                     /Extension="CTTTAATATCTTT"
                     /Description="<html><body></body></html>"
                     /label="oMTV59"
     primer_bind     complement(2856..2884)
                     /Tm=59
                     /Sequence="CATATGAATTCCTCCATCCAAAAAAACGG"
                     /Extension="CGGAT"
                     /Description="<html><body></body></html>"
                     /label="oMTV49"
     primer_bind     complement(2866..2881)
                     /Tm=47
                     /Sequence="ATGAATTCCTCCATCC"
                     /label="pLAR067+R4 (2950 R) - Paraburkholderia CDS
2912"
     misc_feature    2881
                     /label="clone after this"
     misc_feature    2882..6666
                     /label="Ccla1058RV"
     misc_feature    2882..2901
                     /label="F1"
     CDS             2882..6544
                     /codon_start=1
                     /transl_table=1

/translation="MDKAAIKKFAVWARKKLIEDIKQKAYELGITEKEIKKPDVSTSD

TVIIGDRSLNRKEIEQRNSLVSRIEEKGFNNVIEEVAYTWFNRFIALRFMEVNGYLPT

GIRVLSSIEHDRKEPDIIREALNIDLDLERELVYKLQDDNDTESLYRYLLVKQCNALN

EILPGLFEKIDDYTEILLPSNLLAEGSVIRHLVEDISEDDFREQVEIIGWMYQYYISE

KKDEVFEGLKKNIKITKENIPAATQLFTPDWIVKYMVENSLGRLWLEGHPDEELKSKW

KYYLDEAEQEPEVQKQLEEIRAKSKDIRPEDIKVLDPAMGSGHILVYAFDVLYDIYKS

AGYSERDIPKLILENNLYGLDIDDRAAQLAYFAVMMKARSKSRRIFREKINVNVCSIQ

ESNGFPKEAMDYLVNPEETEIEKRLHREDVEYLINVFQDAKEYGSILEVKPIDFDAIE

RRLEEIRDGEIQDLIEYQFKNIIIERIPPLIKQAKIMSQKYNVVCTNPPYMGDDGINL

KLSKFLKENYKDTRSDLFAVFIEKTIKFSTKSSYISMITQNSWMFLRSYEKLRGMILN

GATINSMVHLGPRAFEEIDGEVVRTTAFVLNNNVLNGYKGIYIRLVDIYKSLDKEKEY

FVLKNRYNGVKQNSFKRIPGSPIAYWCSEMVANSFIDGIILKNISEPKQGLITGNSSA

FLRIWYEVNIENIGFKMKDREEAIVSKKKWFPINKGGEYRKWYGNNEYIVNWQNDGIE

IKNYKDEKGKLKSRPQNIEYYFKKGLTWTKVTSAKFSVRFTEDGFIFSEAGMKIFVHD

NLLDYIGSFLNSNLVNVLLGALSETINYEQGNVARLPLKICNDININEKISHLFKTNV
```

```
DISRVDWDSFENSWDFRKHPLLNYKFNGRTIEQAFNNWSAFAEEQFNQLKTNEEELNR

IFIQIYGLQDELTPEVEDKDITIRKADRERDIKSFISYAVGCMFGRYSIDAEGLIYAG

GDFKDKWKNEDGQWKVRKTVKDDESKIIEDTWVNATFVPDMDNIIPITDDEYFEDDIV

SRFVEFLKVTFGEETLEENLDYIADTIGRRTTETARQTIRRYFLKEFYKDHVQVYQKR

PIYWLFDSGKEDGFKALIYMHRYDELAVARVRTDYLHKLQKSYEAEVKRLDIIIDSNA

SQREKANARKKREKILKQMEECLQYDQVIAHVANQRIKIDLDDGVKVNYAKFQGIEVP
                     QGEGRKPLKADLLAKI*"
                     /label="Ccla_1058"
     misc_feature    2882..6001
                     /label="TO CLONE"
     primer_bind     complement(3184..3204)
                     /Tm=59
                     /Sequence="CTCAGAACACGAATGCCGGTC"
                     /Description="<html><body></body></html>"
                     /label="oMTV277"
     primer_bind     3350..3370
                     /Tm=61
                     /Sequence="AACGCGCTGAACGAAATCCTG"
                     /Description="<html><body></body></html>"
                     /label="oMTV278"
     primer_bind     complement(3709..3733)
                     /Tm=59
                     /Sequence="CTCCGCTTCGTCCAGATAGTATTTC"
                     /Description="<html><body></body></html>"
                     /label="oMTV279"
     primer_bind     3871..3892
                     /Tm=56
                     /Sequence="TATCTATAAGAGCGCGGGTTAC"
                     /Description="<html><body></body></html>"
                     /label="oMTV280"
     primer_bind     complement(4420..4444)
                     /Tm=58
                     /Sequence="GGTGTCTTTGTAGTTCTCCTTCAGG"
                     /Description="<html><body></body></html>"
                     /label="oMTV281"
     primer_bind     4477..4500
                     /Tm=59
                     /Sequence="GACCATCAAGTTCAGCACCAAGAG"
                     /Description="<html><body></body></html>"
                     /label="oMTV282"
     primer_bind     complement(4844..4865)
                     /Tm=61
                     /Sequence="CGTCAATGAAGCTGTTCGCCAC"
                     /Description="<html><body></body></html>"
                     /label="oMTV283"
     primer_bind     5023..5044
                     /Tm=57
                     /Sequence="GATTAACAAGGGTGGCGAGTAC"
```

```
                     /Description="<html><body></body></html>"
                     /label="oMTV284"
     primer_bind     complement(5550..5572)
                     /Tm=61
                     /Sequence="GAACGCGCTCCAGTTGTTAAACG"
                     /Description="<html><body></body></html>"
                     /label="oMTV285"
     primer_bind     5550..5572
                     /Tm=61
                     /Sequence="CGTTTAACAACTGGAGCGCGTTC"
                     /Description="<html><body></body></html>"
                     /label="oMTV286"
     primer_bind     complement(5978..6027)
                     /Tm=71

/Sequence="TTCTCTTCCAGGGTCTCTTCACCGAAGGTCACTTTCAGAAACTCAAC
                     GAA"
                     /Description="<html><body></body></html>"
                     /label="oMTV222"
     misc_feature    6002..26
                     /label="GBLOCK"
     primer_bind     complement(6078..6101)
                     /Tm=60
                     /Sequence="GGAAGTAACGACGGATGGTCTGAC"
                     /Description="<html><body></body></html>"
                     /label="oMTV287"
     primer_bind     6081..6103
                     /Tm=60
                     /Sequence="AGACCATCCGTCGTTACTTCCTG"
                     /Description="<html><body></body></html>"
                     /label="oMTV288"
     misc_feature    6545..6601
                     /label="A118 attP"
     misc_feature    6545
                     /label="Clone after this"
     primer_bind     6545..6587
                     /Tm=66

/Sequence="TTGTTTAGTTCCTCGTTTTCTCTCGTTGGAAGAAGAAGAAACG"
                     /Description="<html><body></body></html>"
                     /label="oMTV903"
     misc_feature    6671..6690
                     /label="R1"
ORIGIN
        1 aggatcctgc gggtgccagg gcgtgccctt gggctccccg ggcgcgtact
ccacctcact
       61 cgagaattct catgtttgac agcttatcac tgatcagtga attaatggcg
atgacgcatc
      121 ctcacgataa tatccgggta ggcgcaatca ctttcgtctc tactccgtta
caaagcgagg
      181 ctgggtattt cccggccttt ctgttatccg aaatccactg aaagcacagc
ggctggctga
      241 ggagataaat aataaacgag ggctgtatg cacaaagcat cttctgttga
gttaagaacg
```

301 agtatcgaga tggcacatag ccttgctcaa attggaatca ggtttgtgcc aataccagta
361 gaaacagacg aagaagctag ctttgcactg gattgcgagg ctttgtgctt ctctggagtg
421 cgacaggttt gatgacaaaa aattagcgca agaagacaaa aatcaccttg cgctaatgct
481 ctgttacagg tcactaatac catctaagta gttgattcat agtgactgca tatatgttgt
541 gttttacagt attatgtagt ctgtttttta tgcaaaatct aatttaatat attgatattt
601 atatcatttt acgtttctcg ttcagctttt ttatactaag ttggcattat aaaaaagcat
661 tgcttatcaa tttgttgcaa cgaacaggtc actatcagtc aaaataaaat cattatttga
721 tttcaatttt gtcccactcc ctgcctctgt catcacgata ctgtgatgcc atggctaatt
781 cccatgtcag ccgttaagtg ttcctgtgtc actcaaaatt gctttgagag gctctaaggg
841 cttctcagtg cgttacatcc ctggcttgtt gtccacaacc gttaaacctt aaaagcttta
901 aaagccttat atattctttt ttttcttata aaacttaaaa ccttagaggc tatttaagtt
961 gctgatttat attaatttta ttgttcaaac atgagagctt agtacgtgaa acatgagagc
1021 ttagtacgtt agccatgaga gcttagtacg ttagccatga gggtttagtt cgttaaacat
1081 gagagcttag tacgttaaac atgagagctt agtacgtgaa acatgagagc ttagtacgta
1141 ctatcaacag gttgaactgc tgatcttcag atcctctacg ccggacgcat cgtggccgga
1201 tcttgcggcc gcaaaaatta aaaatgaagt tttgacggta tcgaacccca gagtcccgct
1261 cagaagaact cgtcaagaag gcgatagaag gcgatgcgct gcgaatcggg agcggcgata
1321 ccgtaaagca cgaggaagcg gtcagcccat tcgccgccaa gctcttcagc aatatcacgg
1381 gtagccaacg ctatgtcctg atagcggtcc gccacaccca gccggccaca gtcgatgaat
1441 ccagaaaagc ggccattttc caccatgata ttcggcaagc aggcatcgcc atgggtcacg
1501 acgagatcct cgccgtcggg catccgcgcc ttgagcctgg cgaacagttc ggctggcgcg
1561 agcccctgat gctcttcgtc cagatcatcc tgatcgacaa gaccggcttc catccgagta
1621 cgtgctcgct cgatgcgatg tttcgcttgg tggtcgaatg ggcaggtagc cggatcaagc
1681 gtatgcagcc gccgcattgc atcagccatg atggatactt tctcggcagg agcaaggtga
1741 gatgacagga gatcctgccc cggcacttcg cccaatagca gccagtccct tcccgcttca
1801 gtgacaacgt cgagcacagc tgcgcaagga acgcccgtcg tggccagcca cgatagccgc
1861 gctgcctcgt cttggagttc attcagggca ccggacaggt cggtcttgac aaaaagaacc

1921 gggcgcccct gcgctgacag ccggaacacg gcggcatcag agcagccgat
tgtctgttgt
1981 gcccagtcat agccgaatag cctctccacc caagcggccg gagaacctgc
gtgcaatcca
2041 tcttgttcaa tcatgcgaaa cgatcctcat cctgtctctt gatccactag
attattgaag
2101 catttatcag ggttattgtc tcatgagcgg atacatattt gaatgtattt
agaaaaataa
2161 acaaataggg gttccgcgca catttccccg aaaagtgcca cctgcatcga
tggcccccga
2221 tggtagtgtg gggtctcccc atgcgagagt agggaactgc caggcatcaa
ataaaacgaa
2281 aggctcagtc gaaagactgg gcctttcgtt ttatctgttg tttgtcggtg
aacgctctcc
2341 tgagtaggac aaatccgccg ggagcggatt tgaacgttgc gaagcaacgg
cccggagggt
2401 ggcgggcagg acgcccgcca taaactgcca ggcatcaaat taagcagaag
gccatcctga
2461 cggatggcct ttttgcgtgg ccagtgccaa gcttgcatgc cgtgccccaa
ctggggtaac
2521 ctttgagttc tctcagttgg gggcgtaggg tctagccgcc attcagagaa
gaaaccaatt
2581 gtccatattg catcagacat tgccgtcact gcgtctttta ctggctcttc
tcgctaacca
2641 aaccggtaac cccgcttatt aaaagcattc tgtaacaaag cgggaccaag
gccatgacaa
2701 aaacgcgtag caaaagtgtc tataatcacg gcagaaaagt ccacattgat
tatttgcacg
2761 gcgtcacact ttgctatgcc atagcatttt tatccataag attagcggat
cctacctgac
2821 gctttttatc gcaactctct actgtttctc catacccgtt tttttggatg
gaggaattca
2881 tatggataag gcggcgatca agaaattcgc ggtgtgggcg cgtaagaaac
tgattgagga
2941 catcaaacag aaggcgtacg aactgggtat taccgaaaaa gagatcaaga
aaccggacgt
3001 gagcaccagc gataccgtta tcattggcga ccgtagcctg aaccgtaagg
aaatcgagca
3061 acgtaacagc ctggttagcc gtatcgagga aaaaggtttt aacaacgtga
ttgaggaagt
3121 tgcgtatacc tggttcaacc gttttatcgc gctgcgtttc atggaagtga
acggttacct
3181 gccgaccggc attcgtgttc tgagcagcat cgaacacgac cgtaaggagc
cggatatcat
3241 tcgtgaagcg ctgaacatcg acctggatct ggaacgtgag ctggtgtata
aactgcagga
3301 cgataacgat accgagagcc tgtaccgtta tctgctggtt aagcaatgca
acgcgctgaa
3361 cgaaatcctg ccgggtctgt ttgagaaaat tgacgattac accgaaattc
tgctgccgag
3421 caacctgctg gcggagggca gcgtgattcg tcacctggtt gaagacatca
gcgaggacga
3481 tttccgtgaa caggtggaga tcattggttg gatgtaccaa tactatatca
gcgagaagaa

3541 agatgaagtt tttgagggcc tgaagaaaaa catcaagatc accaaggaaa
acatcccggc
3601 ggcgacccag ctgttcaccc cggactggat cgtgaagtac atggttgaga
acagcctggg
3661 tcgtctgtgg ctggaaggcc acccggatga ggaactgaaa agcaagtgga
aatactatct
3721 ggacgaagcg gagcaagaac cggaagtgca gaaacaactg gaggaaatcc
gtgcgaagag
3781 caaagacatt cgtccggaag atatcaaggt tctggacccg gcgatgggta
gcggccacat
3841 tctggtgtat gcgtttgacg ttctgtacga tatctataag agcgcgggtt
acagcgagcg
3901 tgatattccg aaactgatcc tggaaaacaa cctgtatggc ctggacatcg
acgatcgtgc
3961 ggcgcagctg gcgtactttg cggtgatgat gaaggcgcgt agcaaaagcc
gtcgtatttt
4021 tcgtgagaag atcaacgtga acgtttgcag cattcaagag agcaacggtt
tcccgaaaga
4081 agcgatggac tacctggtga acccggagga aaccgaaatc gagaagcgtc
tgcaccgtga
4141 agatgtggag tatctgatta acgtttttca ggacgcgaag gaatacggta
gcatcctgga
4201 agtgaaaccg attgacttcg atgcgatcga acgtcgtctg gaggaaatcc
gtgatggcga
4261 aattcaggac ctgatcgagt accaattcaa gaacatcatc atcgagcgta
ttccgccgct
4321 gatcaagcag gcgaaaatta tgagccaaaa gtacaacgtg gtttgcacca
acccgccgta
4381 tatgggtgac gatggcatca acctgaagct gagcaaattc ctgaaggaga
actacaaaga
4441 cacccgtagc gacctgttcg cggttttcat cgaaaagacc atcaagttca
gcaccaagag
4501 cagctatatt agcatgatca cccagaacag ctggatgttc ctgcgtagct
acgaaaaact
4561 gcgtggtatg attctgaacg gcgcgaccat caacagcatg gtgcacctgg
gtccgcgtgc
4621 gttcgaggaa attgacggcg aggtggttcg taccaccgcg tttgtgctga
acaacaacgt
4681 tctgaacggt tacaagggca tctacatccg tctggttgat atctacaaga
gcctggacaa
4741 ggaaaaagag tacttcgtgc tgaaaaaccg ttataacggt gttaagcaga
acagctttaa
4801 acgtattccg ggcagcccga ttgcgtactg gtgcagcgag atggtggcga
acagcttcat
4861 tgacggtatt atcctgaaga acatcagcga accgaaacaa ggtctgatta
ccggcaacag
4921 cagcgcgttc ctgcgtatct ggtacgaagt gaacattgag aacatcggtt
ttaagatgaa
4981 agatcgtgag gaagcgatcg ttagcaagaa aaagtggttt ccgattaaca
agggtggcga
5041 gtaccgtaaa tggtatggca acaacgaata tattgttaac tggcagaacg
atggtatcga
5101 gattaaaaac tacaaggacg aaaagggcaa actgaagagc cgtccgcaaa
acatcgagta

5161 ctatttcaaa aagggtctga cctggaccaa ggtgaccagc gcgaaattca gcgttcgttt
5221 taccgaggac ggtttcatct ttagcgaagc gggcatgaag attttcgtgc acgacaacct
5281 gctggattat attggtagct ttctgaacag caacctggtg aacgttctgc tgggtgcgct
5341 gagcgaaacc atcaactacg agcagggcaa cgttgcgcgt ctgccgctga aaatctgcaa
5401 cgatatcaac attaacgaga agattagcca cctgttcaaa accaacgtgg atatcagccg
5461 tgttgactgg gatagcttcg aaaacagctg ggactttcgt aagcacccgc tgctgaacta
5521 taaattcaac ggtcgtacca tcgagcaagc gtttaacaac tggagcgcgt tcgcggagga
5581 acagtttaac caactgaaga ccaacgagga agagctgaac cgtatcttta ttcagatcta
5641 cggcctgcaa gacgagctga ccccggaagt ggaggacaag gatattacca tccgtaaagc
5701 ggaccgtgaa cgtgatatta aaagcttcat cagctacgcg gttggttgca tgtttggccg
5761 ttatagcatt gatgcggagg gtctgatcta cgcgggtggc gacttcaagg ataagtggaa
5821 aaacgaagac ggccagtgga aagtgcgtaa gaccgttaaa gacgatgaaa gcaagattat
5881 cgaggatacc tgggtgaacg cgaccttcgt tccggacatg gataacatta tcccgattac
5941 cgacgatgaa tattttgagg acgatatcgt gagccgtttc gttgagtttc tgaaagtgac
6001 cttcggtgaa gagaccctgg aagagaacct ggactacatt gcggatacca ttggtcgtcg
6061 taccaccgag accgcgcgtc agaccatccg tcgttacttc ctgaaggaat tttataaaga
6121 ccacgtgcag gtttaccaaa agcgtccgat ttattggctg ttcgatagcg gtaaagagga
6181 cggctttaaa gcgctgatct acatgcaccg ttatgatgaa ctggcggtgg cgcgtgttcg
6241 taccgactac ctgcacaagc tgcagaaaag ctatgaagcg gaagtgaagc gtctggacat
6301 tatcattgat agcaacgcga gccagcgtga gaaagcgaac gcgcgtaaaa agcgtgaaaa
6361 gatcctgaag caaatggaag agtgcctgca gtatgatcaa gtgatcgcgc acgttgcgaa
6421 ccaacgtatt aagatcgacc tggacgatgg tgtgaaggtt aactacgcga aattccaggg
6481 cattgaagtt ccgcaaggcg agggccgtaa gccgctgaaa gcggacctgc tggcgaaaat
6541 ctaattgttt agttcctcgt ttctctcgt tggaagaaga agaaacgaga aactaaaatt
6601 atcgcgatag agtcagtact cgacatgttg aaagctatgc atcagaagac aattgatctc
6661 ggatccggcg taatcatggt catagctgtt
//

```
LOCUS       pMTV44                  4080 bp    DNA     circular UNA 02-
MAR-2020
DEFINITION  .
ACCESSION   urn.local...64-exvbs3y
VERSION     urn.local...64-exvbs3y
KEYWORDS    .
SOURCE
  ORGANISM  .
FEATURES             Location/Qualifiers
     misc_feature    1
                     /label="laczalpha"
     misc_feature    1
                     /label="clone before  this"
     misc_feature    1
                     /label="do not include in cloning"
     primer_bind     1..27
                     /Tm=75
                     /Sequence="AGGATCCTGCGGGTGCCAGGGCGTGCC"
                     /Description="<html><body></body></html>"
                     /label="oMTV206"
     misc_feature    4..23
                     /label="plasmid#1 F"
     misc_feature    8..52
                     /label="c31"
     protein_bind    13..46
                     /bound_moiety="phage œÜC31 integrase"
                     /note="minimal attB site for the œÜC31 integrase (<a
                     href=""http://www.ncbi.nlm.nih.gov/pubmed/10801973""

title=""http://www.ncbi.nlm.nih.gov/pubmed/10801973"">Grot
                     h et al., 2000</a>)"
                     /label="attB"
     terminator      115..361
                     /note="transcription terminator
<i>t</i><sub>L3</sub> from
                     phage Œª"
                     /label="Œª tL3 terminator"
     misc_feature    377..769
                     /label="lambda attP"
     primer_bind     complement(418..438)
                     /Tm=58
                     /Sequence="TTGTCATCAAACCTGTCGCAC"
                     /Description="<html><body></body></html>"
                     /label="oMTV29"
     protein_bind    485..718
                     /bound_moiety="Œª integrase"
                     /gene="<i>att</i>P"
                     /note="integrase from phage Œª"
                     /label="Œª attP"
     misc_feature    770..1206
                     /label="oriR6K"
     rep_origin      785..1173
                     /note="Œ≥ replication origin from <i>E. coli</i>
plasmid
```

```
                     R6K; requires the R6K initiator protein pi for
                     replication"
                     /label="R6K Œ≥ ori"
     primer_bind     complement(848..868)
                     /Tm=60
                     /Sequence="CAAGCCAGGGATGTAACGCAC"
                     /Description="<html><body></body></html>"
                     /label="oMTV28"
     misc_feature    complement(1260..2054)
                     /label="Kan"
     CDS             complement(1260..2054)
                     /codon_start=1
                     /gene="<i>aph(3')-II</i> (or <i>nptII</i>)"
                     /note="confers resistance to neomycin, kanamycin,
and G418
                     (Geneticin¬Æ)"
                     /product="aminoglycoside phosphotransferase from
Tn5"
                     /transl_table=1

/translation="MIEQDGLHAGSPAAWVERLFGYDWAQQTIGCSDAAVFRLSAQGR

PVLFVKTDLSGALNELQDEAARLSWLATTGVPCAAVLDVVTEAGRDWLLLGEVPGQDL

LSSHLAPAEKVSIMADAMRRLHTLDPATCPFDHQAKHRIERARTRMEAGLVDQDDLDE

EHQGLAPAELFARLKARMPDGEDLVVTHGDACLPNIMVENGRFSGFIDCGRLGVADRY
                     QDIALATRDIAEELGGEWADRFLVLYGIAAPDSQRIAFYRLLDEFF*"
                     /label="NeoR/KanR"
     primer_bind     complement(1277..1297)
                     /Tm=60
                     /Sequence="GCATCGCCTTCTATCGCCTTC"
                     /Description="<html><body></body></html>"
                     /label="oMTV26"
     primer_bind     1277..1297
                     /Tm=59
                     /Sequence="GAAGGCGATAGAAGGCGATGC"
                     /Description="<html><body></body></html>"
                     /label="oMTV27"
     primer_bind     complement(1415..1426)
                     /Tm=47
                     /Sequence="GCCGGCGGGGTG"
                     /Mismatch_Positions="======G====="
                     /Mismatches=1

/Extension="AAGTGGTATCGCTTTGGGGAACATGCCTTCAGCGGCGGGCGC"
                     /Description="<html><body></body></html>"
                     /label="oMTV17"
     primer_bind     complement(1733..1754)
                     /Tm=56
                     /Sequence="GATCTCCTGTCATCTCACCTTG"
                     /Description="<html><body></body></html>"
                     /label="oMTV24"
     primer_bind     1733..1754
```

```
                     /Tm=56
                     /Sequence="CAAGGTGAGATGACAGGAGATC"
                     /Description="<html><body></body></html>"
                     /label="oMTV25"
     misc_feature    1948..1965
                     /label="seq1_F"
     misc_feature    2180..2197
                     /label="seq6_F"
     primer_bind     complement(2245..2264)
                     /Tm=57
                     /Sequence="CCTGGCAGTTCCCTACTCTC"
                     /Description="<html><body></body></html>"
                     /label="oMTV22"
     primer_bind     2245..2264
                     /Tm=58
                     /Sequence="GAGAGTAGGGAACTGCCAGG"
                     /Description="<html><body></body></html>"
                     /label="oMTV23"
     terminator      2268..2354
                     /gene="<i>Escherichia coli rrnB</i>"
                     /note="transcription terminator T1 from the <i>E.
coli
                     rrnB</i> gene"
                     /label="rrnB T1 terminator"
     misc_feature    2284..382
                     /note="prep 3 "
                     /label="sequenced"
     misc_feature    2442..2459
                     /label="seq2_F"
     terminator      2446..2473
                     /note="transcription terminator T2 from the <i>E.
coli
                     rrnB</i> gene"
                     /label="rrnB T2 terminator"
     misc_feature    2502..2543
                     /label=31
     misc_feature    2556..2873
                     /note="Geneious type: promoter prokaryotic"
                     /note="/Description=araBp7"
                     /label="Promoter_P_1"
     promoter        2556..2873
                     /note="araBp7"
                     /note="/vntifkey=30"
                     /label="Promoter_P_1"
     promoter        2569..2853
                     /gene="<i>araBAD</i>"
                     /note="promoter of the L-arabinose operon of <i>E.
                     coli</i>; the <i>araC</i> regulatory gene is
transcribed
                     in the opposite direction (<a
                     href=""http://www.ncbi.nlm.nih.gov/pubmed/7608087""

title=""http://www.ncbi.nlm.nih.gov/pubmed/7608087"">Guzma
                     n et al., 1995</a>)"
```

```
                     /label="araBAD promoter"
     primer_bind     2806..2825
                     /Tm=59
                     /Sequence="CGGATCCTACCTGACGCTTT"
                     /label="3,770 F"
     primer_bind     complement(2837..2860)
                     /Tm=58
                     /Sequence="AACGGGTATGGAGAAACAGTAGAG"
                     /Description="<html><body></body></html>"
                     /label="oMTV20"
     primer_bind     2837..2860
                     /Tm=59
                     /Sequence="CTCTACTGTTTCTCCATACCCGTT"
                     /Description="<html><body></body></html>"
                     /label="oMTV21"
     primer_bind     complement(2844..2881)
                     /Tm=65
                     /Sequence="ATGAATTCCTCCATCCAAAAAAACGGGTATGGAGAAAC"
                     /Description="<html><body></body></html>"
                     /label="oMTV1"
     primer_bind     complement(2851..2884)
                     /Tm=61
                     /Sequence="CATATGAATTCCTCCATCCAAAAAAACGGGTATG"
                     /Extension="TTGTGGTCGGTGCT"
                     /Description="<html><body></body></html>"
                     /label="oMTV12"
     primer_bind     complement(2851..2881)
                     /Tm=61
                     /Sequence="ATGAATTCCTCCATCCAAAAAAACGGGTATG"
                     /Description="<html><body></body></html>"
                     /label="oMTV205"
     primer_bind     complement(2855..2886)
                     /Tm=62
                     /Sequence="GCCAGATGAATTCCTCCATCCAAAAAAACGGG"

/Mismatch_Positions="====G==========================="
                     /Mismatches=1
                     /Extension="TAGGCCT"
                     /Description="<html><body></body></html>"
                     /label="oMTV3"
     primer_bind     complement(2856..2884)
                     /Tm=59
                     /Sequence="CATATGAATTCCTCCATCCAAAAAAACGG"
                     /Extension="CTTTAATATCTTT"
                     /Description="<html><body></body></html>"
                     /label="oMTV59"
     primer_bind     complement(2856..2884)
                     /Tm=59
                     /Sequence="CATATGAATTCCTCCATCCAAAAAAACGG"
                     /Extension="CGGAT"
                     /Description="<html><body></body></html>"
                     /label="oMTV49"
     misc_feature    2881
                     /label="clone after this"
```

```
     CDS             2882..3871
                     /codon_start=1
                     /transl_table=1

/translation="MAKKVKCLYCGEKYNRTQYPEHLESSHKDGYLRLIENIKKDIQN

DFSIKETAYRNDVTQLFVKKIMKESMIYIKEKESDYYVNKLNIKSWEPENFSLETTTI

WGFPDRGSWATHSGKYRGNWSPFIPRNVILRYSTEGEIVLDQFVGSGTTLVEAKLLNR

KSIGIDINPEAVNIARHNTNFERDGSGEVEVHVGDARHLEFIDDESIDLICTHPPYSN

IIRYSENIQGDLSHCDIKEFYKEMEKVAIECYRVLKKNKFCAILIGDTRKKGHMIPIG

FNVMEIFLRTGFKLKEIVIKEQHNCSSTGYWRNQSIKYNFLLIAHEYLFIFRK*"
                     /label="Ccla_1994"
     misc_feature    2882..2901
                     /label="Forward"
     misc_feature    2882..3988
                     /label="Ccla1994MR11"
     primer_bind     complement(3135..3154)
                     /Tm=55
                     /Sequence="CTCCCAGCTCTTGATGTTCA"
                     /Description="<html><body></body></html>"
                     /label="oMTV289"
     primer_bind     3302..3324
                     /Tm=59
                     /Sequence="GTTCTGGACCAGTTTGTGGGTAG"
                     /Description="<html><body></body></html>"
                     /label="oMTV290"
     primer_bind     complement(3622..3643)
                     /Tm=61
                     /Sequence="CTTCAGCACACGGTAGCATTCG"
                     /Description="<html><body></body></html>"
                     /label="oMTV291"
     primer_bind     3674..3694
                     /Tm=58
                     /Sequence="GATACCCGTAAGAAAGGCCAC"
                     /Description="<html><body></body></html>"
                     /label="oMTV292"
     misc_feature    3872..3923
                     /label="Œ¶370 attP"
     misc_feature    3872
                     /label="clone before this"
     primer_bind     3872..3919
                     /Tm=63

/Sequence="TAAAAAAATACAGCGTTTTTCATGTACAACTATACTAGTTGTAGTGC
                     C"
                     /Description="<html><body></body></html>"
                     /label="oMTV714"
     misc_feature    4061..4080
                     /label="Reverse"
ORIGIN
```

1 aggatcctgc gggtgccagg gcgtgccctt gggctccccg ggcgcgtact ccacctcact
61 cgagaattct catgtttgac agcttatcac tgatcagtga attaatggcg atgacgcatc
121 ctcacgataa tatccgggta ggcgcaatca ctttcgtctc tactccgtta caaagcgagg
181 ctgggtattt cccggccttt ctgttatccg aaatccactg aaagcacagc ggctggctga
241 ggagataaat aataaacgag ggctgtatg cacaaagcat cttctgttga gttaagaacg
301 agtatcgaga tggcacatag ccttgctcaa attggaatca ggtttgtgcc aataccagta
361 gaaacagacg aagaagctag ctttgcactg gattgcgagg ctttgtgctt ctctggagtg
421 cgacaggttt gatgacaaaa aattagcgca agaagacaaa aatcaccttg cgctaatgct
481 ctgttacagg tcactaatac catctaagta gttgattcat agtgactgca tatatgttgt
541 gttttacagt attatgtagt ctgtttttta tgcaaaatct aatttaatat attgatattt
601 atatcatttt acgtttctcg ttcagctttt ttatactaag ttggcattat aaaaaagcat
661 tgcttatcaa tttgttgcaa cgaacaggtc actatcagtc aaaataaaat cattatttga
721 tttcaatttt gtcccactcc ctgcctctgt catcacgata ctgtgatgcc atggctaatt
781 cccatgtcag ccgttaagtg ttcctgtgtc actcaaaatt gctttgagag gctctaaggg
841 cttctcagtg cgttacatcc ctggcttgtt gtccacaacc gttaaacctt aaaagcttta
901 aaagccttat atattctttt ttttcttata aaacttaaaa ccttagaggc tatttaagtt
961 gctgatttat attaatttta ttgttcaaac atgagagctt agtacgtgaa acatgagagc
1021 ttagtacgtt agccatgaga gcttagtacg ttagccatga gggtttagtt cgttaaacat
1081 gagagcttag tacgttaaac atgagagctt agtacgtgaa acatgagagc ttagtacgta
1141 ctatcaacag gttgaactgc tgatcttcag atcctctacg ccggacgcat cgtggccgga
1201 tcttgcggcc gcaaaaatta aaaatgaagt tttgacggta tcgaacccca gagtcccgct
1261 cagaagaact cgtcaagaag gcgatagaag gcgatgcgct gcgaatcggg agcggcgata
1321 ccgtaaagca cgaggaagcg gtcagcccat tcgccgccaa gctcttcagc aatatcacgg
1381 gtagccaacg ctatgtcctg atagcggtcc gccacaccca gccggccaca gtcgatgaat
1441 ccagaaaagc ggccattttc caccatgata ttcggcaagc aggcatcgcc atgggtcacg
1501 acgagatcct cgccgtcggg catccgcgcc ttgagcctgg cgaacagttc ggctggcgcg
1561 agcccctgat gctcttcgtc cagatcatcc tgatcgacaa gaccggcttc catccgagta

1621 cgtgctcgct cgatgcgatg tttcgcttgg tggtcgaatg ggcaggtagc cggatcaagc
1681 gtatgcagcc gccgcattgc atcagccatg atggatactt tctcggcagg agcaaggtga
1741 gatgacagga gatcctgccc cggcacttcg cccaatagca gccagtccct tcccgcttca
1801 gtgacaacgt cgagcacagc tgcgcaagga acgcccgtcg tggccagcca cgatagccgc
1861 gctgcctcgt cttggagttc attcagggca ccggacaggt cggtcttgac aaaaagaacc
1921 gggcgcccct gcgctgacag ccggaacacg gcggcatcag agcagccgat tgtctgttgt
1981 gcccagtcat agccgaatag cctctccacc caagcggccg gagaacctgc gtgcaatcca
2041 tcttgttcaa tcatgcgaaa cgatcctcat cctgtctctt gatccactag attattgaag
2101 catttatcag ggttattgtc tcatgagcgg atacatattt gaatgtattt agaaaaataa
2161 acaaataggg gttccgcgca catttccccg aaaagtgcca cctgcatcga tggcccccga
2221 tggtagtgtg gggtctcccc atgcgagagt agggaactgc caggcatcaa ataaaacgaa
2281 aggctcagtc gaaagactgg gcctttcgtt ttatctgttg tttgtcggtg aacgctctcc
2341 tgagtaggac aaatccgccg ggagcggatt tgaacgttgc gaagcaacgg cccggagggt
2401 ggcgggcagg acgcccgcca taaactgcca ggcatcaaat taagcagaag gccatcctga
2461 cggatggcct ttttgcgtgg ccagtgccaa gcttgcatgc cgtgccccaa ctggggtaac
2521 ctttgagttc tctcagttgg gggcgtaggg tctagccgcc attcagagaa gaaaccaatt
2581 gtccatattg catcagacat tgccgtcact gcgtctttta ctggctcttc tcgctaacca
2641 aaccggtaac cccgcttatt aaaagcattc tgtaacaaag cgggaccaag gccatgacaa
2701 aaacgcgtag caaaagtgtc tataatcacg gcagaaaagt ccacattgat tatttgcacg
2761 gcgtcacact ttgctatgcc atagcatttt tatccataag attagcggat cctacctgac
2821 gctttttatc gcaactctct actgtttctc catacccgtt tttttggatg gaggaattca
2881 tatggcgaag aaagttaaat gcctgtactg cggcgagaag tataaccgta cccagtaccc
2941 ggagcacctg gaaagcagcc acaaagacgg ttatctgcgt ctgattgaga acatcaagaa
3001 agacattcag aacgatttca gcatcaaaga aaccgcgtat cgtaacgacg ttacccaact
3061 gtttgtgaag aaaatcatga aggagagcat gatctacatt aaggagaaag aaagcgatta
3121 ctacgttaac aagctgaaca tcaagagctg ggagccggaa aacttcagcc tggaaaccac
3181 caccatttgg ggttttccgg atcgtggcag ctgggcgacc cacagcggca agtatcgtgg

3241 caactggagc ccgttcattc cgcgtaacgt gatcctgcgt tacagcaccg
agggcgaaat
3301 cgttctggac cagtttgtgg gtagcggcac caccctggtt gaggcgaaac
tgctgaaccg
3361 taagagcatc ggtattgata tcaacccgga ggcggtgaac atcgcgcgtc
acaacaccaa
3421 cttcgaacgt gacggtagcg gcgaggtgga agttcacgtg ggcgatgcgc
gtcacctgga
3481 gtttattgac gatgaaagca ttgacctgat ctgcacccac ccgccgtata
gcaacatcat
3541 tcgttacagc gaaaacatcc aaggtgacct gagccactgc gatatcaagg
agttctacaa
3601 ggagatggaa aaggttgcga tcgaatgcta ccgtgtgctg aagaaaaaca
aattctgcgc
3661 gattctgatc ggtgataccc gtaagaaagg ccacatgatt ccgatcggtt
ttaacgttat
3721 ggagattttc ctgcgtaccg gctttaagct gaaagagatt gtgatcaagg
aacagcacaa
3781 ctgcagcagc accggttatt ggcgtaacca aagcattaaa tacaactttc
tgctgatcgc
3841 gcacgaatac ctgttcattt ttcgtaagta ataaaaaaat acagcgtttt
tcatgtacaa
3901 ctatactagt tgtagtgcct aaagacatgt gagtcagtac tctgaaagct
atgcatcaga
3961 agacaattga tctctcgcga taggatccgg cgtaatcatg gtcatagctg
tatcggatcc
4021 cgggcccgtc gactgcagag gcctgcatgc aagcttggcg taatcatggt
catagctgtt
//
